# Sterically Controlled Late‐Stage Functionalization of Bulky Phosphines

**DOI:** 10.1002/chem.202202074

**Published:** 2022-08-01

**Authors:** Hao Deng, Marco Bengsch, Nico Tchorz, Constanze N. Neumann

**Affiliations:** ^1^ Department of Heterogeneous Catalysis Max-Planck-Institut für Kohlenforschung Kaiser-Wilhelm-Platz 1 45470 Mülheim an der Ruhr Germany

**Keywords:** borylation, iridium catalysis, late-stage functionalization, ligand libraries, phosphines

## Abstract

The fine‐tuning of metal‐phosphine‐catalyzed reactions relies largely on accessing ever more precisely tuned phosphine ligands by de‐novo synthesis. Late‐stage C−H functionalization and diversification of commercial phosphines offers rapid access to entire libraries of derivatives based on privileged scaffolds. But existing routes, relying on phosphorus‐directed transformations, only yield functionalization of C${}_{\mathrm{sp}{}^{2}}$ −H bonds in a specific position relative to phosphorus. In contrast to phosphorus‐directed strategies, herein we disclose an orthogonal functionalization strategy capable of introducing a range of substituents into previously inaccessible positions on arylphosphines. The strongly coordinating phosphine group acts solely as a bystander in the sterically controlled borylation of bulky phosphines, and the resulting borylated phosphines serve as the supporting ligands for palladium during diversification through phosphine self‐assisted Suzuki‐Miyaura reactions.

## Graphical abstract

**Due to the high affinity** of phosphines for transition metals, C−H functionalization of aryl phosphines is dominated by phosphorus‐directed reactions. Here, we pair a sterically controlled borylation reaction of unprotected phosphines with phosphine self‐assisted Suzuki–Miyaura reactions to grant ready access to libraries of functionalized phosphines with a positional selectivity that complements existing methods.

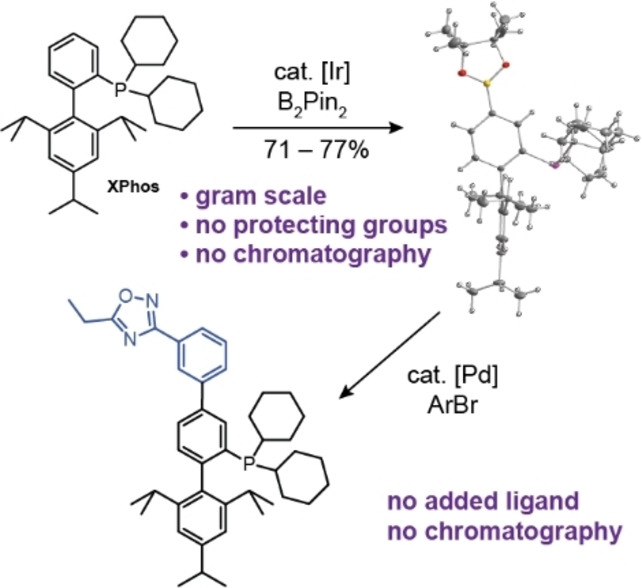

## Introduction

The development of an optimized supporting ligand has frequently led to considerable improvements in transition‐metal‐catalyzed transformations, and even enabled the discovery of previously unknown transformations.^[1]^ Rapid optimization of catalytic processes through high‐throughput experimentation (HTE), design of experiments (DoE), or autonomous process optimization, for which a set of commercially available ligands is generally selected for testing, is becoming increasingly common.^[2]^ Furthermore, ligand parameterization in combination with machine learning can highlight which modifications to the phosphine ligand are likely to improve catalytic performance.^[3]^ Synthetic methods that permit late‐stage elaboration of the ligand scaffold of commercially available phosphine ligands facilitate rapid exploration of a targeted area of chemical space without the need for de‐novo synthesis of each separate ligand.

**Scheme 1:**
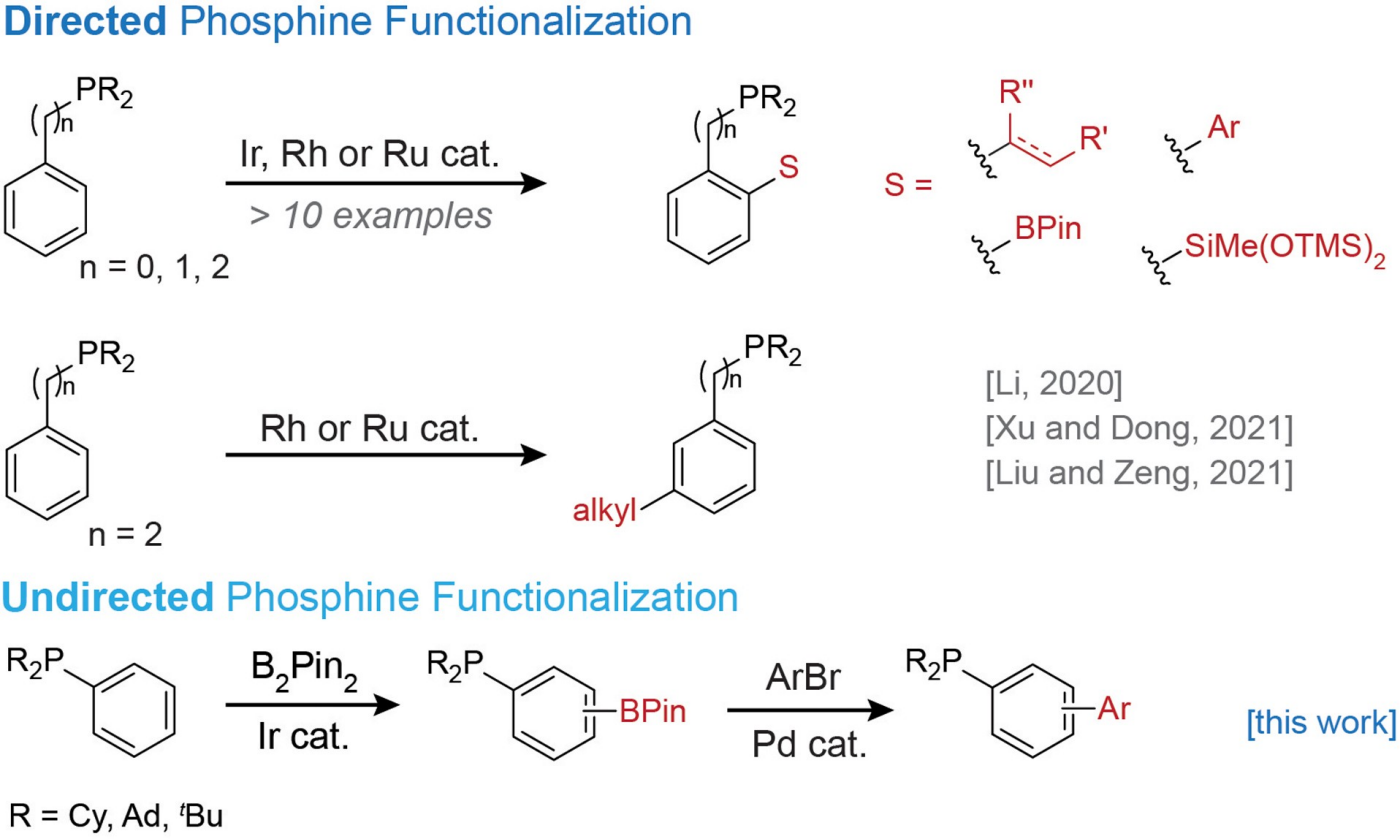
Directed and undirected metal‐catalyzed C−H functionalization of aryl phosphines.[^6c^, ^7^, ^8^, ^9^, ^10^, ^11^]

Substantial alterations to the phosphine structure can be made by replacing rather than modifying one of the phosphine substituents: Morandi et al. disclosed carbon–phosphorus bond metathesis that enables the scrambling of substituents between different triarylphosphines,^[4]^ and more recently, the replacement of aryl for alkyl substituent in (alkyl)arylphosphines by alkylation followed by nickel‐catalyzed dearylation.^[5]^ For the generation of catalyst libraries with more fine‐grained structural modifications, an attractive option consists in regioselective C−H functionalization, ideally with a substituent capable of further differentiation, so that the initial functionalization serves as a branching point for a range of ligands. The presence of a strongly coordinating phosphine group, however, is a double‐edged sword: enforced proximity of the catalyst to a particular C−H bond can enhance both regioselectivity and overall reactivity, but the high affinity of phosphines to transition metals can likewise prevent catalyst turnover.^[6]^ Previously reported arylphosphine C−H borylation reactions, which are catalyzed by rhodium, ruthenium or iridium, or mediated by boron bromide reagents, all rely on the directing effect of the phosphine substituent to achieve high regioselectivity (Scheme 1).[^6c^, ^7^] Directed arylation,^[8]^ alkylation,^[9]^ alkenylation[^9b^, ^10^] and silylation^[11]^ provide efficient access to additional substituents, but the use of a directing group strategy still limits functionalization to the C−H bond that lies closest to a catalyst engaged in dative bonding with the phosphorus lone pair,[^8^, ^9b^, ^9e^, ^9f^, ^10^, ^11^, ^12^] or the C−H bond para to this position.[^9a^, ^9c^, ^9d^] Here we report an *undirected* C−H borylation reaction of bulky phosphines, where the most sterically accessible position(s) of the scaffold undergo functionalization, so that a distinct regioisomer is obtained compared with phosphorus directed C−H borylation reactions. Many of the commonly employed phosphine ligands, for which rapid diversification would be enabling, carry bulky substituents such as cylcohexyl, *tert*‐butyl, or adamantyl on phosphorus, which makes it possible to disfavor phosphine‐directed borylation to access a sterically controlled pathway.

Diversification of C−H borylated arylphosphines has successfully been shown by functional group interconversion, and, in isolated cases, by Suzuki reactions, which show moderate yield,^[7c]^ or require protection of the phosphine group.^[6c]^ A broadly applicable and operationally facile protocol for the Suzuki reaction of substrates containing an unprotected phosphine substituent would elevate borylated phosphines to a branching point from which a multitude of ligand derivatives are accessible. Inspired by two prior reports of phosphine self‐assisted reactions involving phosphine substituted aryl chlorides^[13]^ and alkenyl iodides,^[14]^ we developed a palladium‐catalyzed reaction in which the borylated phosphines act as supporting ligands in their own further functionalization. By targeting different positions on the phosphine skeleton, our two‐step borylation–arylation sequence complements existing methods for the synthesis of arylated phosphine ligand libraries (Scheme 1).^[8c]^ In an effort to render phosphine diversification operationally facile, we relied on commercially available ligands and [Ir(COD)OMe]_2_ (Table S1 in the Supporting Information), and developed purification protocols that require neither protecting groups nor chromatography.

## Results and Discussion

**Scheme 2:**
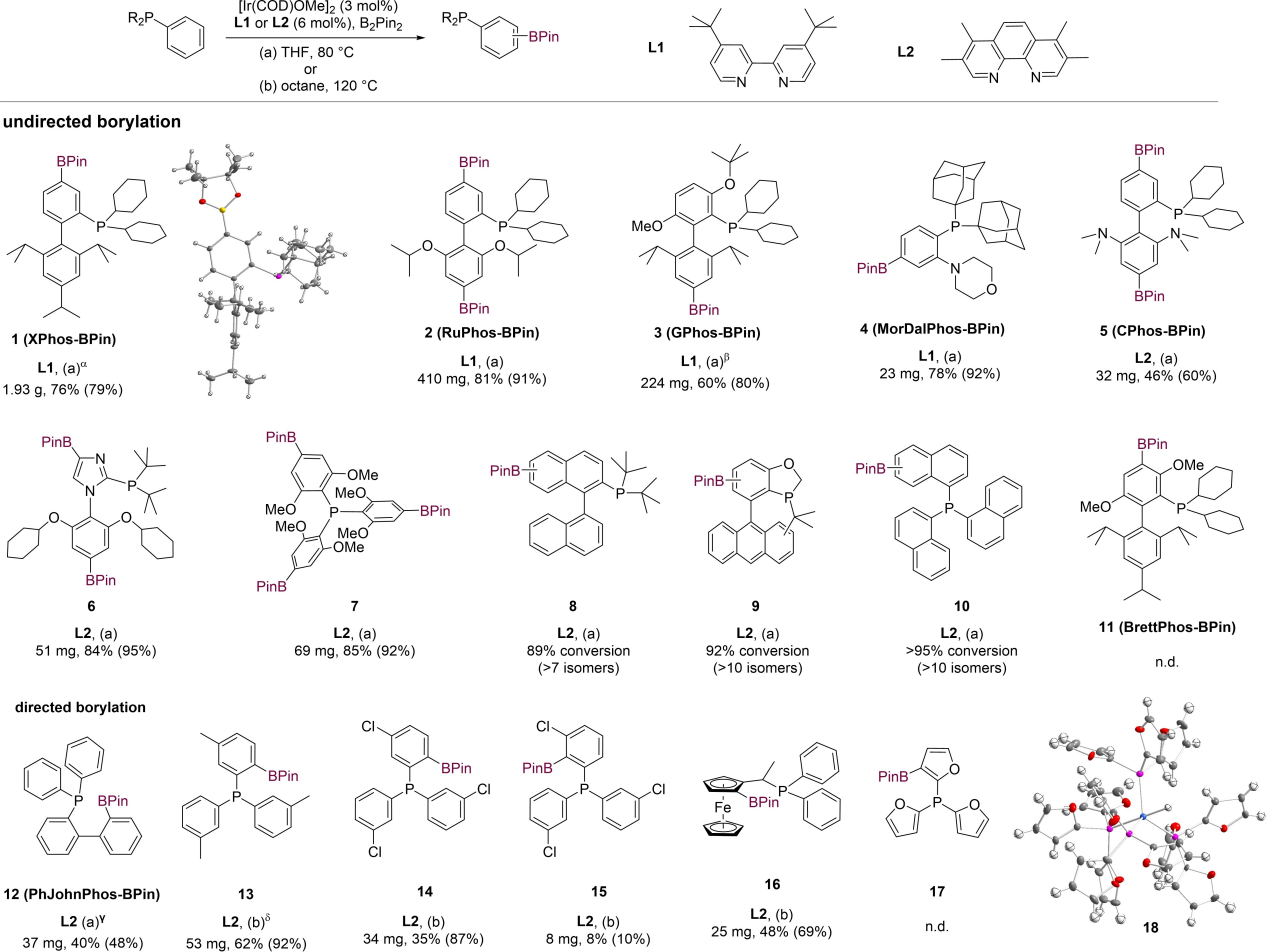
Substrate scope of iridium‐catalyzed borylation showing the isolated amount of product and isolated yields obtained for the regioisomer depicted, along with NMR yields for the depicted regioisomer in brackets. [α] 1 mol % [Ir(COD)OMe]_2_ and 2 mol % **L1** used; [β] 5 mol % [Ir(COD)OMe]_2_ and 10 mol % **L1** used; [γ] HBPin was used in place of B_2_Pin_2_; [δ] isolated yield corresponds to a 7 : 1 mixture of two regioisomers; n. d.=not detected.

We began our work by testing if commercially available alkylarylphosphines could successfully undergo iridium‐catalyzed borylation despite the presence of a strongly coordinating phosphine moiety, by exploring different iridium precursors, supporting ligands, and solvents (Table S2). Xphos,^[15]^ RuPhos,^[16]^ MorDalPhos^[17]^ and CPhos^[18]^ could be efficiently transformed into phosphine borates **1**, **2**, **4** and **5** using [Ir(COD)OMe]_2_ and **L1** or **L2** in THF at 80 °C (Scheme 2). Structurally related BrettPhos^[19]^ proved unreactive however, which we attributed to the steric hindrance surrounding all available C${}_{\mathrm{sp}{}^{2}}$ −H bonds. In the case of RuPhos, on the other hand, the dominant product observed after 12 h is diborylated, with functionalization occurring on both the top and bottom aryl ring. Based on the results of BrettPhos and RuPhos borylation, we predicted that GPhos^[20]^ would undergo highly regioselective monofunctionalization, and indeed only a single new resonance corresponding to **3** was visible in the ^31^P NMR spectrum of the concentrated reaction mixture when GPhos was subjected to borylation. Likewise, a bulky imidazole‐based phosphine developed for the Barbier–Negishi coupling of secondary alkyl bromides with aryl triflates,^[21]^ gave rise to a single regioisomer during C−H borylation. Notably, borylation on the heteroarene was observed in addition to functionalization of the carboarene for **6**, rendering the imidazole ring accessible to further functionalization.

For tris(2,6‐dimethoxylphenyl)phosphine, the use of 3.0 equiv. B_2_Pin_2_ led to efficient formation of the triborylated product (**7**), while triarylphosphines devoid of *ortho* substituents proved to be unreactive in the presence of either **L1** or **L2**. We found however, that the use of octane instead of THF as a solvent, and an increase in the reaction temperature from 80 to 120 °C led to substantial yields of the monoborylated triarylphosphines **13**, **14** and **15**. A single regioisomer of PhJohnPhos‐BPin (**12**) was formed featuring borylation in the δ‐position with respect to the phosphorus atom, which suggests a phosphine‐directed reaction, in which the substrate is serving as a ligand for iridium. In the case of **16**, borylation of ferrocene occurred *ortho* to the alkyldiphenylphosphine substituent, which also suggests a directed reaction pathway. In the case of tris(3‐chlorophenyl)phosphine, two different β‐borylated regioisomers (**14** and **15**) could be isolated from the same borylation reaction, with minor isomer **15** featuring BPin in a crowded *ortho*, *ortho’*‐disubstituted position. Unlike **1**–**10**, phosphine borates **13**, **14** and **15** were formed in a phosphorus‐directed reaction because only borylation of the β‐position with respect to the phosphine substituent was observed, while for a sterically controlled pathway highly selective functionalization of the γ‐position would be expected.^[22]^ Undirected borylation reactions such as those yielding **1** or **7** require the presence of a diamine ligand (Scheme S1), while the rate of the directed reaction forming **13**, **14** and **15** was decreased, and the formation of **12** unaffected (Scheme S2), by the presence of **L2**.^[7a]^ However, optimized reaction conditions for directed β‐borylation include **L2**, because the slower rate of reaction facilitated the isolation of mono‐ rather than diborylated products in the synthesis of **13**, **14** and **15**.

Directed borylation was only observed for phosphines lacking bulky substituents, while sterically directed borylation was observed for all cylohexyl‐, *tert*‐butyl‐, and adamantyl‐substituted phosphines we tested. XPhos, RuPhos, MorDalPhos and CPhos did not give rise to phosphine‐directed β‐borylation. Furthermore, for substrates where the γ (**10**), δ (**8**) or ϵ (**9**) positions relative to phosphorus are unsubstituted, multiple C−H bonds underwent borylation at comparable rates, ruling out a substantial rate enhancement for the borylation of a particular position due to coordination of iridium to the phosphorus center (Scheme S3). Neither iridium‐catalyzed phosphine‐directed β‐borylation in more than 16 % yield,^[6c]^ nor undirected borylation of phosphines has previously been reported (Schemes 1 and S4).

For some phosphines, an isomer distinct from that accessible by directed phosphine borylation (Scheme 1) can also be accessed by *meta*‐directed borylation of the corresponding phosphine oxide^[23]^ or phosphonium salt^[24]^ using [Ir(COD)OMe]_2_ and ligands requiring two‐step synthesis. In the context of phosphine library synthesis, a suitable phosphine scaffold for elaboration may be commercially available, whereas the corresponding phosphine oxide commonly has to be synthesized. Following borylation, reduction of the phosphine oxide, which can be a challenging transformation (Scheme S5, Figure S1) is required. The products shown in Scheme 2, on the other hand, were all obtained in one step from commercially available starting materials.

Analogously to what had been previously observed in rhodium‐catalyzed directed borylation,^[6c]^ trifurylphosphine failed to yield the desired borylated product **17**. X‐ray analysis of crystals retrieved from attempted C−H borylation of trifurylphosphine revealed pentacoordinate iridium complex **18** featuring four trifurylphosphine and one hydride ligand in a distorted trigonal bipyramidal arrangement, the composition of which was confirmed by IR spectroscopy and high‐resolution mass spectrometry (Figure S2). Sequestration of the catalyst from the reaction mixture through the formation of a thermally stable iridium complex that is poorly soluble in octane likely explains why no C${}_{\mathrm{sp}{}^{2}}$ −H borylation was observed for trifurylphosphine. Likewise, only low conversions (<25 %) could be achieved for all diphosphine substrates tested (Scheme S6). We suspect that binding of the chelating diphosphine in preference to **L1** or **L2** yields an iridium complex that fails to catalyze the aromatic C−H borylation reaction. When we tested if site‐selective monoborylation of **6** or **7** could be achieved at lower reaction temperatures (Schemes S7 and S8, Figures S3 and S4), we observed that borylation of the heterocycle‐containing substrate stalled at 30 % conversion at 60 °C and 15 % at room temperature, while full conversion was achieved at 80 °C within 3 h (Figure S4), suggesting that the phosphine containing substrate irreversibly (Figure S5) deactivates the iridium catalyst at lower reaction temperatures.

**Scheme 3:**
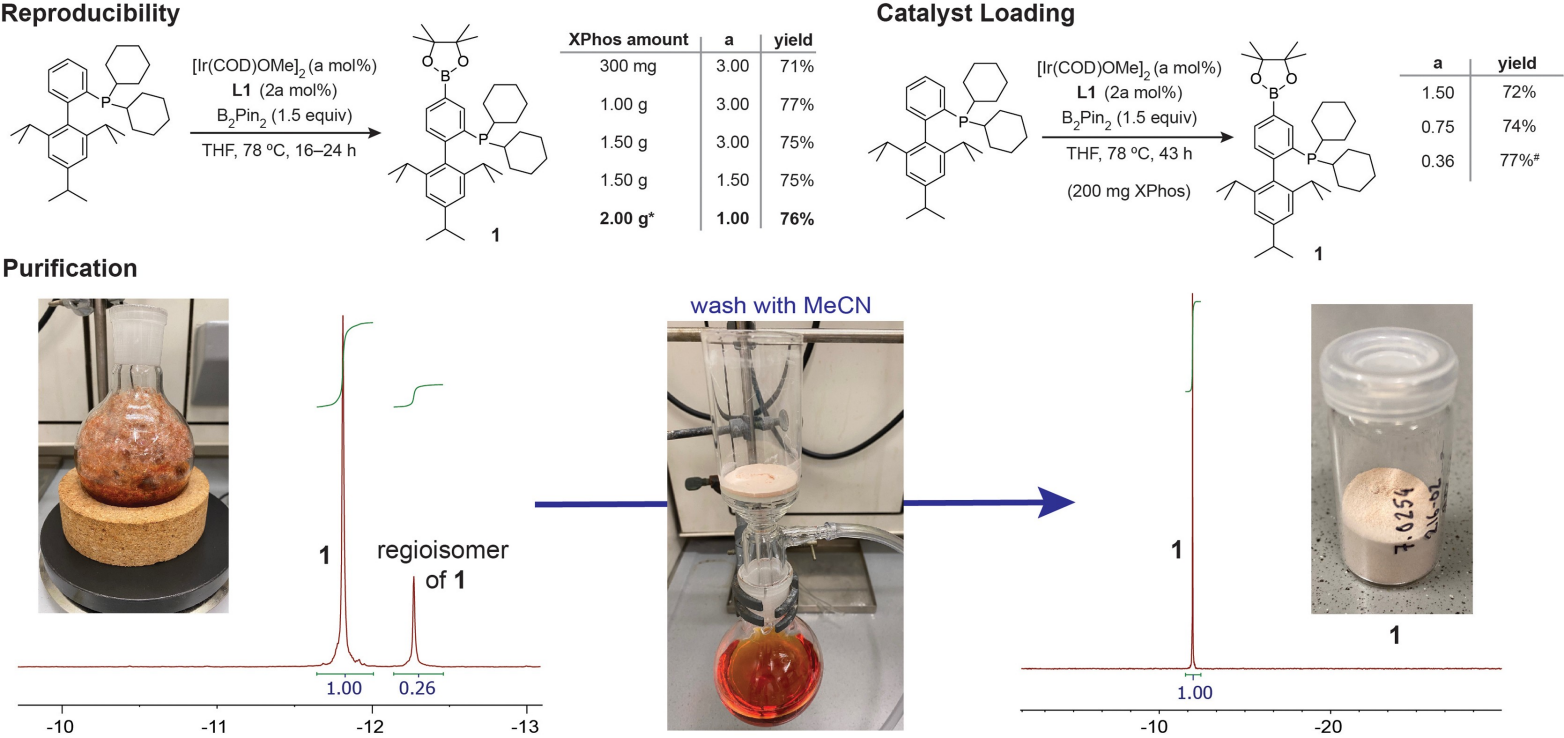
Borylation of XPhos proceeds efficiently on the gram scale, and a single regioisomer of **1** is obtained after simple washing of the concentrated reaction mixture. Full conversion was achieved on large and small reaction scales even with reduced catalyst loading. * 1.2 equiv. B_2_Pin_2_ was used. ^#^ **1** was isolated in 97 % purity.

Because several C−H bonds are commonly available for functionalization, the utility of late‐stage C−H functionalization largely rests on the ease with which a single regioisomer can be isolated, which can be very challenging even for air‐stable substrates.[^23^, ^25^] For alkylarylphosphines and some arylphosphines, a further complication arises in that temporary protection of the phosphine moiety is required to ensure product stability during column chromatography. In directed C−H borylation reactions with iridium, rhodium or ruthenium, borylated phosphines were purified by chromatography after formation of a BH_3_ adduct, from which the free phosphine can be recovered by treatment with DABCO or diethylamine.[^6c^, ^7a^, ^7b^] Here, we employ a simple washing procedure, which obviates the need for BH_3_ protection and deprotection, even for sensitive substrates. Due to the limited number of C−H bonds available in **3**, **6**, **7** and **12**, the reaction conditions could be tuned so that only a single borylated isomer was formed. For substrates where mixtures of isomers were obtained, we observed that the different regioisomers displayed stark solubility differences in polar solvents, which allowed us to isolate a single regioisomer of the phosphine borate in analytically pure fashion after simple washing of the concentrated reaction mixture with acetonitrile or methanol for **1**, **2**, **4**, **5** and **16** (Scheme 3). Borylation of XPhos on scales between 200 mg and 2.00 g afforded **1** in consistently high yields (71–77 %, *n*=7, Scheme 3) and purity. The iridium catalyst loading could be reduced below 1 mol % without affecting the isolated yield of **1**, and only the lowest catalyst loading tested (0.36 mol % Ir) failed to reach full conversion after 43 h (Scheme 3B). While the δ‐borylated regioisomer of **1**, phosphine oxides, catalyst and **L1** were efficiently removed by washing with MeCN, residual XPhos was retained, leading to isolation of **1** in undiminished yield but only 97 % purity when 0.36 mol % [Ir(COD)OMe]_2_ was employed.

**Scheme 4:**
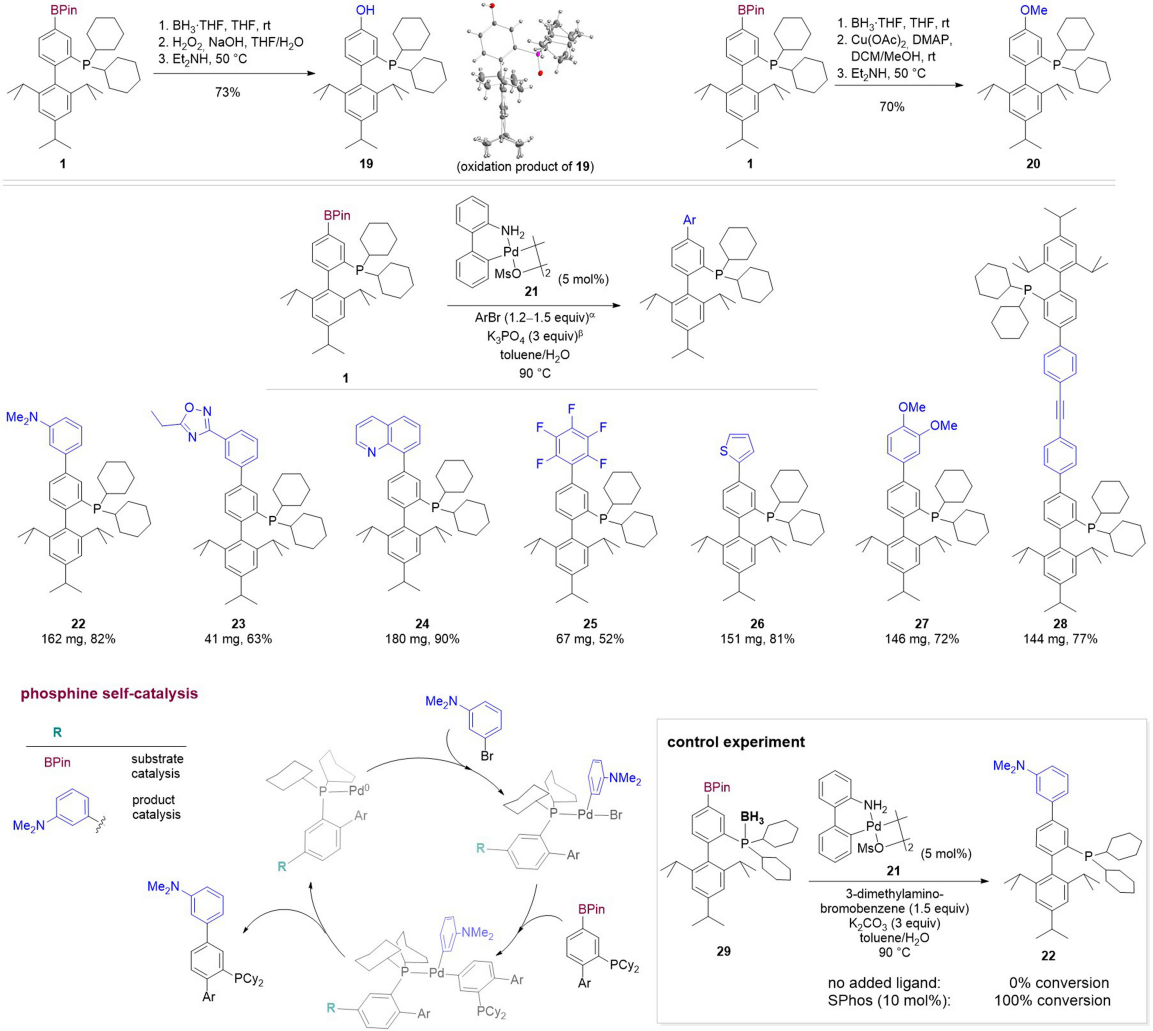
Synthesis of a library of XPhos ligand derivatives by functional group interconversion and arylation by phosphine self‐catalysis. [α] for **25**, iodopentafluorobenzene was used and the reaction was carried out at 100 °C; for **28** only 0.5 equiv. ArBr was used. [β] for **22**, K_2_CO_3_ (3 equiv) was used; Ar=2,6‐(diisopropyl)phenyl.

Previously reported methods of functional group interconversion and Suzuki coupling make it possible to generate additional phosphine ligand derivatives from borane protected phosphine borates, which we illustrate here by the synthesis of XPhos‐OH (**19**) and XPhos‐OMe (**20**) from **1**.^[7a]^ The use of a borane protecting group, which is routinely employed even for functionalization reactions that do not employ an oxidant, such as the Suzuki reactions with aryl halides, result in a three step sequence for the further elaboration of phosphine borates. To expedite the generation of libraries of phosphine catalysts, we tested if instead of protecting phosphine centers from coordination with palladium, phosphine borates such as **1** could serve as the supporting ligand in their own further elaboration. Despite the significant deviation from the optimal ligand : Pd ratio of around 1–3 : 1,^[26]^ subjecting **1** to 3‐(dimethylamino)bromobenzene in presence of catalytic amounts of palladium dimer **21** and K_2_CO_3_, resulted in complete conversion to arylated XPhos **22**. Simple filtration of the reaction mixture of the Suzuki reaction over celite followed by trituration with acetonitrile furnished analytically pure XPhos derivatives **22**–**24** and **26**–**28**. Different substituents including heteroaromatic moieties could thus be installed on the phosphine scaffold without the need for additional supporting ligands for palladium, protecting groups or purification by column chromatography. The use of 0.5 equivalents of bis(4‐bromophenyl)acetylene in phosphine self‐assisted arylation of **1** gave access to **28**, which contains two XPhos moieties linked via a rigid tether. In the synthesis of arylated XPhos derivative **22**, full conversion could also be achieved in the presence of only 1 mol % **21**, which corresponds to a Pd/ligand ratio of 1: 50. For polyfluorinated XPhos derivative **25**, which was the material most prone to air oxidation among all phosphine derivatives investigated, the isolated product contained minor impurities which we attribute to oxidative decomposition. Derivatized phosphines could often be stored under air in the solid state without undergoing oxidation (**1** and **2** were stable under air for more than 3 months and **19** for more than a week), but showed limited solution stability, so that attempted crystallization of **19** under air yielded a single crystal of the corresponding phosphine oxide (Scheme 4).

**Scheme 5:**
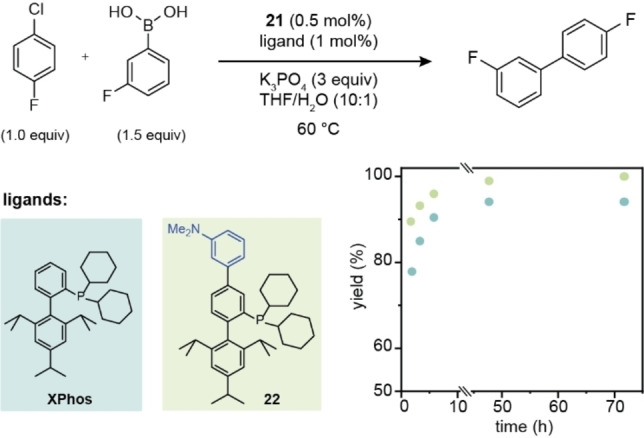
Comparative ability of XPhos and arylated XPhos derivative **22** to promote the palladium‐catalyzed Suzuki reaction of an aryl chloride (yield obtained after stated intervals determined by ^19^F NMR).

While directing groups are commonly employed in transition metal catalysis to enhance reactivity or alter site selectivity, coordination of **1** to the palladium center does not position the C−B bond in a suitable arrangement to undergo transmetalation (Scheme 4). We thus surmised that one molecule of XPhos‐BPin (**1**) or XPhos‐Ar (**22**) acts as the supporting ligand for palladium while a second molecule of **1** undergoes transmetalation to the palladium center (Scheme 4), which requires that both **1** and **22** are competent ligands for Suzuki–Miyaura reactions. We verified that a supporting ligand is required to achieve the observed catalyst activity by submitting **29**, the BH_3_ adduct of **1**, to palladium‐catalyzed cross coupling with 3‐(dimethylamino)bromobenzene, where no C−C bond formation could be detected. Repeating the reaction in the presence of 10 mol % SPhos,^[26a]^ however, resulted in complete conversion of **29** to arylated phosphine **22** (Scheme 4). To verify that not only **1** but also **22** is a competent ligand in Suzuki–Miyaura couplings, as would be required for phosphine self‐assisted arylation to take place, we compared the performance of **22** with the well‐studied XPhos ligand. Replacement of XPhos with arylated XPhos derivative **22** in the Suzuki–Miyaura reaction of 1‐chloro‐4‐fluorobenzene increased the rate of formation of biaryl product (Scheme 5 and Figure S6).^[26a]^ Even at room temperature and using only 0.25 mol % palladium dimer **21**, a 1.00:0.62 ratio of 1‐chloro‐4‐fluorobenzene and the biaryl product was formed after 5 h (Table S3).

## Conclusion

In summary, sterically controlled iridium‐catalyzed C−H borylation is a facile entry point for the creation of libraries of phosphines with bulky substituents. Borylation reactions can be carried out on a gram scale, and the resulting phosphine borates can be isolated as single regioisomers without the need for protecting groups or chromatography. Our two‐step approach involving C−H borylation and phosphine self‐assisted Suzuki–Miyaura reactions complements ruthenium‐ and rhodium‐catalyzed phosphine‐directed arylation reactions by granting access to different regioisomers.

## Experimental Section

**General experimental procedures**: Unless otherwise specified, chemicals were obtained from commercial suppliers and used as received. For NMR analysis of air‐sensitive samples, [D_6_]benzene was stored over 4 Å molecular in an argon glovebox. The 4 Å molecular sieves were activated at 300 °C under dynamic vacuum (5×10^−6^ mbar) for 3 days prior to use. Pd catalyst **21**,^[27]^ Gphos,^[20]^ and [Ir(COD)OH]_2_ ^[28]^ were synthesized according to literature procedures.

**Mass spectrometry**: A QExactive instrument from Thermo Fisher Scientific with direct injection to the sprayer was used to collect ESI measurements. Nuclear Magnetic Resonance (NMR) Spectroscopy: NMR data were recorded using a Bruker AVIII HD 300 MHz, Bruker AVIII HD 400 MHz, or Bruker AVNeo 600 MHz NMR spectrometer. ^1^H and ^13^C chemical shifts are referenced to the deuterated solvent as internal standard. Determination of which position underwent borylation was made based on ^1^H, ^13^C, [^1^H, ^1^H] COSY, [^13^C, ^1^H] HSQC, [^13^C, ^1^H] HMBC, [^31^P, ^1^H] HMBC, [^15^N, ^1^H] HMBC and [^1^H, ^1^H] NOESY NMR spectra of isolated compounds (and confirmed by single crystal X‐ray crystallography in the case of **1**). Single crystal X‐ray diffraction (SC‐XRD): SC‐XRD data were recorded on Bruker AXS Enraf‐Nonius KappaCCD diffractometer with a FR591 rotating Mo‐anode X‐ray source and a Bruker‐AXS Kappa Mach3 with APEX‐II detector and IμS microfocus Mo‐anode X‐ray source. Flash column chromatography: VWR silica gel (40‐63 μm) was used.

**General procedure for phosphine C−H borylation**: In an argon glove box, phosphine (1.00 equiv.), [Ir(COD)OMe]_2_ (*a* mol %), diamine ligand (**L1** or **L2**, 2 a mol %), B_2_Pin_2_ (*b* equiv) and solvent (THF or *n*‐octane) were added to a borosilicate vial or Schlenk flask. The resulting suspension was heated at 80 °C (for reactions in THF) or 120 °C (for reactions in *n*‐octane) outside the glovebox. After *c* hours, the reaction mixture was left to cool to room temperature and concentrated. Borylated phosphines were isolated either by washing with acetonitrile or methanol (alkylarylphosphines) or column chromatography (triarylphosphines).

**1**: *a*=1, **L1**, THF, *b*=1.20, *c*=24, isolated by washing with MeCN. ^1^H NMR (600 MHz, C_6_D_6_): *δ*=8.35 (s, 1H), 7.95 (d, *J*=7.6 Hz, 1H), 7.16 (dd, *J*=7.6, 3.7 Hz, 1H), 7.04 (s, 2H), 2.69 (hept, *J*=6.9 Hz, 1H), 2.58 (hept, *J*=6.9 Hz, 2H), 1.80–1.69 (m, 4H), 1.67–1.58 (m, 2H), 1.46–1.34 (m, 6H), 1.24 (d, *J*=6.8 Hz, 6H), 1.12–1.00 (m, 12H), 0.96–0.81 (m, 22H). ^13^C NMR (151 MHz, C_6_D_6_): *δ*=151.8, 151.6, 148.5, 146.5, 139.3 (139.32), 139.3 (139.30), 137.5, 137.4, 137.1, 137.0, 135.0, 131.7 (131.73), 131.7 (131.69), 128.6, 120.7, 83.9, 34.8, 34.7, 34.6, 31.7, 31.6, 31.2 (31.22), 31.2 (31.21), 29.9, 29.8, 27.9, 27.8, 27.5, 27.4, 26.8, 26.3, 25.0, 24.4, 23.4, 23.3. ^31^P NMR (122 MHz, C_6_D_6_): *δ*=−11.5. HRMS‐ESI (*m*/*z*): calcd for C_39_H_60_BO_2_P: 603.4497 [*M*+H]^+^; found: 603.4499.

**2**: *a*=3, **L1**, THF, *b*=2.2, *c*=24, isolated by recrystallization from methanol. ^1^H NMR (300 MHz, C_6_D_6_): *δ*=8.53 (t, *J*=1.7 Hz, 1H), 8.20 (dt, *J*=7.6, 1.1 Hz, 1H), 7.44 (s, 2H), 7.35 (dd, *J*=7.6, 3.4 Hz, 1H), 4.28 (hept, *J*=6.0 Hz, 2H), 2.08–1.79 (m, 6H), 1.70–1.56 (m, 6H), 1.34–1.10 (m, 21H), 1.08–0.98 (m, 19H), 0.87 (d, *J*=6.0 Hz, 6H). ^13^C NMR (151 MHz, C_6_D_6_): *δ*=156.5 (156.50), 156.5 (156.49), 148.5, 148.2, 139.1 (139.10), 139.1 (139.08), 137.3, 137.1, 134.8, 131.2, 131.1, 127.5, 127.4, 112.7, 83.8, 83.6, 70.2, 35.1, 35.0, 31.0, 30.9, 30.6, 30.5, 27.7, 27.6 (27.64), 27.6 (27.56), 27.1, 25.1 (25.10), 25.1 (25.05), 22.5, 22.2. ^31^P NMR (122 MHz, C_6_D_6_): *δ*=−8.8. HRMS‐ESI (*m*/*z*): calcd for C_42_H_65_B_2_O_6_P: 719.4778 [*M*+H]^+^; found: 719.4781.

**3**: *a*=5, **L1**, THF, *b*=2, *c*=72, isolated by washing with methanol. ^1^H NMR (600 MHz, C_6_D_6_): *δ*=8.23 (s, 2H), 6.70 (d, *J*=9.0 Hz, 1H), 6.46 (d, *J*=9.0 Hz, 1H), 3.16 (s, 3H), 2.83 (hept, *J*=6.8 Hz, 2H), 2.45 (tt, *J*=12.1, 3.2 Hz, 2H), 2.02–1.92 (m, 2H), 1.79–1.67 (m, 6H), 1.66–1.60 (m, 2H), 1.44 (d, *J*=6.8 Hz, 6H), 1.41 (s, 9H), 1.38–1.17 (m, 10H), 1.15 (d, *J*=6.7 Hz, 6H), 1.13 (s, 12H). ^13^C NMR (151 MHz, C_6_D_6_): *δ*=153.1 (153.14), 153.1 (153.12), 151.5, 151.4, 146.5, 146.4, 140.4, 140.2, 139.9 (139.93), 139.9 (139.87), 129.6, 127.3, 127.1, 122.6, 112.7, 110.6, 83.4, 77.1, 54.1, 38.3, 38.2, 34.0, 33.9, 30.9, 30.8 (30.84), 30.8 (30.80), 29.0, 28.5 (28.53), 28.5 (28.49), 28.1, 28.0, 26.9, 25.6, 25.1, 24.1. ^31^P NMR (243 MHz, C_6_D_6_): *δ*=−4.5. HRMS‐ESI (*m*/*z*): calcd for C_41_H_64_BO_4_P: 663.4708 [*M*+H]^+^; found: 663.4713.

**4**: *a*=3, **L1**, THF, *b*=2.2, *c*=24, isolated by washing with methanol. ^1^H NMR (600 MHz, C_6_D_6_): *δ*=7.97 (dd, *J*=7.5, 1.2 Hz, 1H), 7.94 (dd, *J*=4.6, 1.2 Hz, 1H), 7.86 (dd, *J*=7.5, 1.4 Hz, 1H), 3.82 (t, *J*=4.4 Hz, 4H), 3.01 (t, *J*=4.5 Hz, 4H), 2.16–2.02 (m, 12H), 1.86 (t, *J*=3.1 Hz, 6H), 1.64 (d, *J*=3.3 Hz, 12H), 1.16 (s, 12H). ^13^C NMR (151 MHz, C_6_D_6_): *δ*=159.7, 159.6, 137.3, 129.2, 127.1, 83.9, 67.3, 54.1, 54.0, 42.4, 42.3, 37.4, 37.3, 37.2, 29.4 (29.41), 29.4 (29.36), 25.0. ^31^P NMR (243 MHz, C_6_D_6_): *δ*=20.5. HRMS‐ESI (*m*/*z*): calcd for C_36_H_53_NO_3_PB: 590.3929 [*M*+H]^+^; found: 590.3931.

**5**: *a*=3, **L2**, THF, *b*=3, *c*=24, isolated by washing with methanol. ^1^H NMR (600 MHz, C_6_D_6_): *δ*=8.60 (t, *J*=1.6 Hz, 1H), 8.20 (dt, *J*=7.6, 1.1 Hz, 1H), 7.86 (s, 2H), 7.52 (dd, *J*=7.6, 3.6 Hz, 1H), 2.42 (s, 12H), 2.12–2.00 (m, 4H), 1.82 (d, *J*=9.6 Hz, 2H), 1.67 (d, *J*=12.1 Hz, 2H), 1.57 (d, *J*=8.5 Hz, 4H), 1.35 (qt, *J*=12.7, 3.8 Hz, 2H), 1.23–1.05 (m, 32H). ^13^C NMR (151 MHz, C_6_D_6_): *δ*=153.6 (153.57), 153.6 (153.56), 150.8, 150.6, 139.7, 139.6, 137.1, 137.0 (137.04), 137.0 (137.02), 136.9, 134.4, 132.9 (132.94), 132.9 (132.89), 121.8, 83.7 (83.71), 83.7 (83.70), 45.2, 35.6, 35.5, 31.9, 31.7, 30.4, 30.3, 28.1, 28.0, 27.7, 27.6, 27.0, 25.1, 25.0. ^31^P NMR (243 MHz, C_6_D_6_): *δ*=−8.8. HRMS‐ESI (*m*/*z*): calcd for C_40_H_63_B_2_N_2_O_4_P: 689.4784 [*M*+H]^+^; found: 689.4791.

**6**: *a*=3, **L2**, THF, *b*=3, *c*=24, isolated by washing with MeCN. ^1^H NMR (600 MHz, C_6_D_6_): *δ*=7.82 (d, *J*=2.8 Hz, 1H), 7.47 (s, 2H), 4.12–4.04(m, 2H), 1.79 (d, *J*=12.6 Hz, 2H), 1.60 (t, *J*=7.6 Hz, 2H), 1.45 (d, *J*=11.9 Hz, 18H), 1.40–1.23 (m, 10H), 1.13 (s, 12H), 1.08 (s, 12H), 0.97–0.87 (m, 6H). ^13^C NMR (151 MHz, C_6_D_6_): *δ*=155.0, 151.8, 151.7, 135.2, 133.9, 130.5, 122.9 (122.94), 122.9 (122.93), 112.9, 84.2, 83.0, 76.7, 33.5, 33.4, 32.3, 31.5, 31.2, 31.1, 25.6, 25.1, 25.0, 23.7 (23.74), 23.7 (23.71). ^31^P NMR (243 MHz, C_6_D_6_): *δ*=8.4. HRMS‐ESI (*m*/*z*): calcd for C_41_H_67_B_2_N_2_O_6_P: 737.4996 [*M*+H]^+^; found: 737.5000.

**7**: *a*=3, **L2**, THF, *b*=3, *c*=24, isolated by washing with methanol. ^1^H NMR (600 MHz, C_6_D_6_): *δ*=7.27 (d, *J*=2.9 Hz, 6H), 3.22 (s, 18H), 1.15 (s, 36H). ^13^C NMR (151 MHz, C_6_D_6_): *δ*=162.6, 162.5, 122.4, 122.3, 110.7, 83.6, 55.7, 25.1. ^31^P NMR (243 MHz, C_6_D_6_): *δ*=−61.9. HRMS‐ESI (*m*/*z*): calcd for C_42_H_60_B_3_O_12_P: 821.4174 [*M*+H]^+^; found: 821.4172.

**12**: *a*=3, **L2**, THF, 1.1 equiv. HBpin, *c*=24, isolated by column chromatography on silica gel (pentane/EtOAc 40 : 1, *v*/*v*). ^1^H NMR (600 MHz, C_6_D_6_): *δ*=8.13 (ddd, *J*=7.4, 1.5, 0.6 Hz, 1H), 7.49 (tt, *J*=6.8, 1.4 Hz, 2H), 7.39–7.34 (m, 2H), 7.31 (dddd, *J*=7.6, 3.8, 1.5, 0.5 Hz, 1H), 7.22 (dddd, *J*=7.5, 4.5, 1.5, 0.6 Hz, 1H), 7.14–7.09 (m, 4H), 7.08–7.05 (m, 3H), 7.05–7.00 (m, 4H), 1.06 (s, 6H), 0.99 (s, 6H). ^13^C NMR (151 MHz, C_6_D_6_): *δ*=150.5, 150.3, 148.4 (148.43), 148.4 (148.39), 139.6, 139.5, 138.3, 138.2, 137.3, 137.2, 135.4, 134.8, 134.6, 134.2, 134.0, 133.6, 130.8 (130.81), 130.8 (130.79), 130.7 (130.71), 130.7 (130.67), 129.8, 128.7, 128.6, 128.5 (128.53), 128.5 (128.48), 128.4, 127.3, 126.7, 83.2, 25.1, 24.5. ^31^P NMR (243 MHz, C_6_D_6_): *δ*=−13.5. HRMS‐ESI (*m*/*z*): calcd for C_30_H_30_BO_2_P: 465.2149 [*M*+H]^+^; found: 465.2151.

**13**: *a*=3, **L2**, *n*‐octane, *b*=2.2, *c*=24, isolated by column chromatography on silica gel (pentane/EtOAc 40 : 1, *v*/*v*). ^1^H NMR (600 MHz, C_6_D_6_): *δ*=8.16 (dd, *J*=7.6, 3.1 Hz, 1H), 7.47 (d, *J*=8.1 Hz, 2H), 7.38 (t, *J*=7.3 Hz, 2H), 7.10 (d, *J*=4.5 Hz, 1H), 7.07 (t, *J*=7.5 Hz, 2H), 6.99–6.96 (m, 1H), 6.91 (dd, *J*=7.7, 1.8 Hz, 2H), 2.00 (s, 6H), 1.91 (s, 3H), 1.03 (s, 12H). ^13^C NMR (151 MHz, C_6_D_6_): *δ*=145.5, 145.4, 140.8, 140.0, 139.9, 138.0 (138.03), 138.0 (137.98), 136.6, 136.5, 135.5, 135.4, 134.7, 134.6, 133.9, 133.8, 131.9, 131.8, 129.4, 128.7, 128.6, 128.4, 83.7, 24.8, 21.6, 21.3. ^31^P NMR (122 MHz, C_6_D_6_): *δ*=−3.5. HRMS‐ESI (*m*/*z*): calcd for C_27_H_32_O_2_PB: 431.2306 [*M*+H]^+^; found: 431.2306.

**14**: *a*=3, **L2**, *n*‐octane, *b*=2.2, *c*=24, isolated by column chromatography on silica gel (pentane/DCM 2 : 1, *v*/*v*). ^1^H NMR (600 MHz, C_6_D_6_): *δ*=7.88 (ddd, *J*=8.0, 3.0, 0.4 Hz, 1H), 7.48 (dddd, *J*=7.8, 2.1, 1.5, 0.4 Hz, 2H), 7.08 (ddd, *J*=3.6, 2.1, 0.4 Hz, 1H), 7.05 (dd, *J*=7.9, 2.1 Hz, 1H), 7.01–6.96 (m, 4H), 6.71–6.66 (m, 2H), 0.94 (s, 12H). ^13^C NMR (151 MHz, C_6_D_6_): *δ*=146.6, 146.4, 140.7, 140.6, 138.2 (138.23), 138.2 (138.21), 138.2 (138.17), 135.3, 135.2, 134.4, 134.2, 132.5 (132.50), 132.5 (132.49), 132.3, 132.2, 130.3, 130.2, 129.3, 84.3, 24.6. ^31^P NMR (243 MHz, C_6_D_6_): *δ*=−3.1. HRMS‐ESI (*m*/*z*): calcd for C_24_H_23_O_2_BPCl_3_: 491.0667 [*M*+H]^+^; found: 491.0667.

**15**: *a*=3, **L2**, *n*‐octane, *b*=2.2, *c*=24, isolated by column chromatography on silica gel (pentane/DCM 2 : 1, *v*/*v*). ^1^H NMR (600 MHz, C_6_D_6_): *δ*=7.40 (dddd, *J*=7.4, 2.0, 1.4, 0.4 Hz, 2H), 7.01 (dt, *J*=8.0, 0.7 Hz, 1H), 6.98 (ddd, *J*=8.0, 2.1, 1.0 Hz, 2H), 6.96–6.94 (m, 2H), 6.81 (ddd, *J*=7.7, 3.8, 0.9 Hz, 1H), 6.69 (td, *J*=7.8, 1.6 Hz, 2H), 6.61 (td, *J*=7.9, 0.7 Hz, 1H), 1.23 (s, 12H). ^13^C NMR (151 MHz, C_6_D_6_): *δ*=140.2, 140.1, 137.7, 137.6, 136.6, 136.5, 133.3, 133.2, 131.5, 131.3, 129.8, 129.7, 129.4, 128.9, 128.2 (128.23), 128.2 (128.18), 127.8, 127.2, 83.0, 23.2 (23.17), 23.2 (23.16). ^31^P NMR (243 MHz, C_6_D_6_): *δ*=−6.4. HRMS‐ESI (*m*/*z*): calcd for C_24_H_23_O_2_BPCl_3_: 491.0667 [*M*+H]^+^; found: 491.0667.

**16**: *a*=3, **L2**, *n*‐octane, *b*=2.2, *c*=72, isolated by washing with MeCN. ^1^H NMR (600 MHz, C_6_D_6_): *δ*=7.76 (ddd, *J*=8.1, 6.9, 1.5 Hz, 2H), 7.27 (ddd, *J*=7.9, 6.2, 1.4 Hz, 2H), 7.22–7.19 (m, 2H), 7.18–7.17 (m, 1H), 7.05–7.01 (m, 2H), 7.01–6.97 (m, 1H), 4.55 (dd, *J*=2.4, 1.3 Hz, 1H), 4.29–4.20 (m, 2H), 4.19–4.14 (m, 2H), 4.10 (s, 4H), 1.54 (dd, *J*=12.8, 7.1 Hz, 3H), 1.10 (d, *J*=3.1 Hz, 12H). ^13^C NMR (151 MHz, C_6_D_6_): *δ*=138.7, 138.5, 136.8, 136.7, 135.2, 135.1, 132.9, 132.8, 128.9, 128.1, 127.2, 99.3, 99.2, 82.4, 73.1, 70.8, 70.2 (70.16), 70.2 (70.19), 68.8, 30.8, 30.7, 25.0, 24.7, 18.3, 18.2. ^31^P NMR (122 MHz, C_6_D_6_): *δ*=13.36. HRMS‐ESI (*m*/*z*): calcd for C_30_H_34_BFeO_2_P: 525.1812 [*M*+H]^+^; found: 525.1811.

**Functional group interconversion**: The synthesis of **19** and **20** from **1** was carried out according to a modified literature procedures[^6c^, ^7a^, ^29^] (see the Supporting Information).

**19**: ^1^H NMR (300 MHz, C_6_D_6_): *δ*=7.24 (s, 2H), 7.09 (dd, *J*=8.2, 4.0 Hz, 1H), 7.03–7.00 (m, 1H), 6.45 (dd, *J*=8.3, 2.6 Hz, 1H), 3.84 (s, 1H), 2.96–2.75 (m, 3H), 1.95–1.58 (m, 13H), 1.47 (d, *J*=6.9 Hz, 6H), 1.31–1.27 (m, 7H), 1.25–1.13 (m, 14H). ^13^C NMR (151 MHz, C_6_D_6_): *δ*=154.6, 148.3, 147.3, 140.0, 139.8, 139.0, 138.9, 137.0, 136.9, 132.7 (132.73), 132.7 (132.69), 120.6, 118.9 (118.87), 118.9 (118.85), 115.3, 34.9, 34.8 (34.79), 34.8 (34.75), 31.5, 31.4, 31.1, 29.7, 29.6, 27.9 (27.94), 27.9 (27.86), 27.7, 27.6, 26.8, 26.4, 24.4, 23.4 (23.38), 23.4 (23.37). ^31^P NMR (122 MHz, C_6_D_6_): *δ*=−11.3. HRMS‐ESI (*m*/*z*): calcd for C_33_H_49_OP: 493.3594 [*M*+H]^+^; found: 493.3592.

**20**: ^1^H NMR (300 MHz, C_6_D_6_): *δ*=7.38 (t, *J*=2.2 Hz, 1H), 7.24 (s, 2H), 7.18 (d, *J*=4.1 Hz, 1H), 6.64 (dd, *J*=8.4, 2.7 Hz, 1H), 3.36 (s, 3H), 2.94‐2.77 (m, 3H), 1.95‐1.87 (m, 2H), 1.82‐1.73 (m, 4H), 1.67‐1.54 (m, 6H), 1.45 (d, *J*=6.9 Hz, 6H), 1.29‐1.11 (m, 22H). ^13^C NMR (75 MHz, C_6_D_6_): *δ*=158.7, 148.6, 147.6, 140.5, 140.0, 139.2, 138.9, 137.3 (137.34), 137.3 (137.26), 133.0, 132.9, 128.9, 120.9, 119.4 (119.44), 119.4 (119.40), 112.9, 55.0, 35.2, 35.1, 35.0, 31.9, 31.7, 31.4 (31.42), 31.4 (31.39), 30.0, 29.8, 28.2, 28.1, 27.9, 27.8, 27.1, 26.7, 24.7, 23.7, 23.6. ^31^P NMR (122 MHz, C_6_D_6_): *δ*=−11.2. HRMS‐ESI (*m*/*z*): calcd for C_34_H_51_OP: 507.3750 [*M*+H]^+^; found: 507.3758.

**General procedure for phosphine self‐assisted Suzuki–Miyaura coupling**: Borylated phosphine (1.00 equiv.), aryl bromide (1.20 equiv), K_3_PO_4_ (3.00 equiv.), toluene and water (4 : 1 *v*/*v*) were added to a Schlenk flask, and the solution was purged with argon before **21** (5 mol %) was added. The reaction mixture was heated at 90 °C until the borylated phosphine was fully converted, cooled to room temperature and filtered over a pad of celite using THF. The filtrate was concentrated under reduced pressure and the product was isolated by washing with acetonitrile or methanol.

**22**: 1.5 equiv. aryl bromide was used and K_2_CO_3_ was used instead of K_3_PO_4_; isolated in 82 % yield by washing with acetonitrile. ^1^H NMR (300 MHz, C_6_D_6_): *δ*=8.14 (t, *J*=2.0 Hz, 1H), 7.59 (dd, *J*=7.8, 1.8 Hz, 1H), 7.37 (dd, *J*=7.9, 3.9 Hz, 1H), 7.32–7.26 (m, 3H), 7.21–7.17 (m, 1H), 7.04 (dd, *J*=2.6, 1.6 Hz, 1H), 6.60 (ddd, *J*=8.2, 2.6, 1.0 Hz, 1H), 2.94–2.82 (m, 3H), 2.57 (s, 6H), 2.00–1.86 (m, 6H), 1.72–1.52 (m, 7H), 1.47 (d, *J*=6.9 Hz, 6H), 1.30–1.24 (m, 10H), 1.20–1.09 (m, 11H). ^13^C NMR (151 MHz, C_6_D_6_): *δ*=151.4, 148.5, 147.1, 147.0, 146.9, 142.7, 141.1, 137.8, 137.7, 137.1, 137.0, 132.3 (132.29), 132.3 (132.25), 131.8, 131.7, 129.9, 127.3, 120.7, 116.3, 112.3, 112.2, 40.2, 35.0, 34.8 (34.84), 34.8 (34.82), 31.7, 31.6, 31.2 (31.24), 31.2 (31.23), 29.8, 29.7, 28.0, 27.9, 27.7, 27.6, 26.9, 26.5, 24.4, 23.4 (23.41), 23.4 (23.40). ^31^P NMR (122 MHz, C_6_D_6_): *δ*=−11.5. HRMS‐ESI (*m*/*z*): calcd for C_41_H_58_NP: 596.4380 [*M*+H]^+^; found: 596.4385.

**23**: Toluene/water ratio was 6 : 1 (*v*/*v*); isolated in 63 % yield by washing with methanol; ^1^H NMR (600 MHz, C_6_D_6_): *δ*=8.73–8.69 (m, 1H), 8.32 (ddd, *J*=7.7, 1.6, 1.1 Hz, 1H), 8.12 (dd, *J*=10.9, 2.0 Hz, 1H), 7.55 (ddd, *J*=7.8, 2.0, 1.2 Hz, 1H), 7.36 (ddd, *J*=7.9, 2.0, 1.2 Hz, 1H), 7.30–7.21 (m, 3H), 7.19 (td, *J*=7.8, 0.5 Hz, 1H), 2.91 (hept, *J*=6.9 Hz, 1H), 2.84 (hept, *J*=6.8 Hz, 2H), 2.27 (q, *J*=7.6 Hz, 2H), 2.09–1.94 (m, 4H), 1.84 (d, *J*=13.2 Hz, 2H), 1.73–1.35 (m, 16H), 1.30 (d, *J*=6.9 Hz, 6H), 1.25–0.99 (m, 12H), 0.96 (t, *J*=7.6 Hz, 3H). ^13^C NMR (151 MHz, C_6_D_6_): *δ*=181.1, 168.6, 148.4, 146.6, 145.9, 145.8, 141.4, 138.4 (138.43), 138.4 (138.36), 136.6, 136.5, 134.7 (134.74), 134.7 (134.67), 133.8, 133.3, 130.6 (130.64), 130.6 (130.57), 129.9 (129.91), 129.9 (129.85), 128.8 (128.78), 128.8 (128.77), 128.7, 127.0, 126.6, 120.6, 38.7, 38.2, 34.8, 31.6, 27.2, 27.1, 27.0, 26.9, 26.8 (26.82), 26.8 (26.80), 26.6 (26.58), 26.6 (26.56), 26.5, 26.4, 24.4, 23.4, 20.1, 10.5. ^31^P NMR (122 MHz, C_6_D_6_): *δ*=−11.4. HRMS‐ESI (*m*/*z*): calcd for C_43_H_57_N_2_OP: 649.4281 [*M*+H]^+^; found: 649.4285.

**24**: Isolated in 90 % yield by washing with MeCN; ^1^H NMR (600 MHz, C_6_D_6_): *δ*=8.80 (dd, *J*=4.1, 1.9 Hz, 1H), 8.45 (t, *J*=2.0 Hz, 1H), 7.69–7.64 (m, 2H), 7.55 (ddd, *J*=8.2, 1.9, 0.4 Hz, 1H), 7.45 (dd, *J*=7.8, 3.9 Hz, 1H), 7.36 (dd, *J*=8.5, 1.5 Hz, 1H), 7.29 (d, *J*=0.5 Hz, 2H), 7.22 (dd, *J*=8.1, 7.1 Hz, 1H), 6.78 (dd, *J*=8.2, 4.1 Hz, 1H), 2.97 (hept, *J*=6.8 Hz, 2H), 2.94–2.88 (m, 1H), 2.14–2.02 (m, 4H), 1.95 (tq, *J*=12.3, 3.2 Hz, 2H), 1.73–1.66 (m, 4H), 1.63–1.58 (m, 2H), 1.51 (d, *J*=6.9 Hz, 6H), 1.42–1.31 (m, 6H), 1.29 (d, *J*=6.9 Hz, 6H), 1.28–1.23 (m, 3H), 1.19 (d, *J*=6.7 Hz, 6H), 1.03–1.01 (m, 1H). ^13^C NMR (151 MHz, C_6_D_6_): *δ*=150.1, 148.3, 147.0, 146.8, 146.7, 141.3, 137.9, 137.3 (137.32), 137.3 (137.28), 136.3 (136.34), 136.3 (136.32), 136.2, 136.0, 131.5, 131.4, 130.5, 130.1, 129.1, 127.6, 126.5, 121.0, 120.7, 35.0, 34.8 (34.84), 34.8 (34.76), 31.6, 31.5, 31.2 (31.18), 31.2 (31.16), 29.8, 29.7, 28.2, 28.1, 27.7, 27.6, 26.9, 26.5, 24.4, 23.4 (23.40), 23.4 (23.38). ^31^P NMR (122 MHz, C_6_D_6_): *δ*=−11.1. HRMS‐ESI (*m*/*z*): calcd for C_42_H_54_NP: 604.4067 [*M*+H]^+^; found: 604.4071.

**25**: Toluene/water ratio 6 : 1 (*v*/*v*); 1.5 equiv. iodopentafluorobenzene was used, and the reaction mixture was heated at 100 °C; isolated in 52 % yield by washing with MeCN; ^1^H NMR (600 MHz, C_6_D_6_): *δ*=7.93–7.88 (m, 1H), 7.39 (dd, *J*=7.9, 3.7 Hz, 1H), 7.28–7.23 (m, 3H), 2.89 (hept, *J*=6.9 Hz, 1H), 2.76 (hept, *J*=6.8 Hz, 2H), 2.02–1.85 (m, 6H), 1.72–1.56 (m, 10H), 1.45 (d, *J*=6.9 Hz, 6H), 1.34–1.25 (m, 12H), 1.15 (d, *J*=6.7 Hz, 6H). ^13^C NMR (151 MHz, C_6_D_6_): *δ*=149.8, 149.6, 149.0, 146.8, 138.7, 138.5, 136.3 (136.29), 136.3 (136.26), 134.4 (134.38), 134.4 (134.36), 132.2 (132.23), 132.2 (132.19), 129.7, 124.9, 120.9, 35.1, 35.0, 34.8, 31.5, 31.4, 31.3 (31.29), 31.3 (31.28), 29.8, 29.7, 28.0, 27.9, 27.7, 27.6, 26.8, 26.4, 24.4, 23.3 (23.29), 23.3 (23.28). ^31^P NMR (243 MHz, C_6_D_6_) δ–12.1. ^19^F NMR (282 MHz, C_6_D_6_): *δ*=−143.8 to −144.0 (m, 2F), −156.0 (t, *J*=21.8 Hz, 1F), −162.1 to −162.4 (m, 2F). HRMS‐ESI (*m*/*z*): calcd for C_39_H_48_PF_5_: 643.3487 [*M*+H]^+^; found: 643.3484.

**26**: 1.5 equiv. aryl bromide was used; isolated in 81 % yield by washing with MeCN; ^1^H NMR (600 MHz, C_6_D_6_): *δ*=8.11 (t, *J*=1.9 Hz, 1H), 7.45 (ddd, *J*=7.9, 2.0, 0.5 Hz, 1H), 7.27–7.23 (m, 3H), 7.22 (dd, *J*=3.6, 1.2 Hz, 1H), 6.86 (dd, *J*=5.1, 1.2 Hz, 1H), 6.80 (dd, *J*=5.1, 3.6 Hz, 1H), 2.90 (hept, *J*=6.9 Hz, 1H), 2.81 (hept, *J*=6.8 Hz, 2H), 1.99–1.81 (m, 8H), 1.69–1.54 (m, 10H), 1.47 (d, *J*=6.9 Hz, 6H), 1.31–1.28 (m, 8H), 1.17 (d, *J*=6.7 Hz, 6H), 1.10–1.07 (m, 2H). ^13^C NMR (151 MHz, C_6_D_6_): *δ*=148.7, 147.6, 147.4, 146.9, 144.8, 138.3, 138.2, 136.8, 136.7, 133.0, 132.5 (132.51), 132.5 (132.47), 130.0 (130.04), 130.0 (130.02), 125.7, 125.0, 123.4, 120.8, 34.9, 34.8 (34.81), 34.8 (34.76), 31.6, 31.5, 31.2 (31.22), 31.2 (31.21), 29.7, 29.6, 28.0, 27.9, 27.6 (27.64), 27.6 (27.58), 26.8, 26.4, 24.4, 23.4, 23.3. ^31^P NMR (243 MHz, C_6_D_6_): *δ*=−12.0. HRMS‐ESI (*m*/*z*): calcd for C_37_H_51_PS: 559.3522 [*M*+H]^+^; found: 559.3526.

**27**: 1.5 equiv. aryl bromide was used; isolated in 72 % yield by washing with MeCN; ^1^H NMR (300 MHz, C_6_D_6_): *δ*=8.08 (t, *J*=2.0 Hz, 1H), 7.56–7.45 (m, 1H), 7.38 (dd, *J*=7.9, 3.8 Hz, 1H), 7.30–7.21 (m, 3H), 7.10 (d, *J*=2.1 Hz, 1H), 6.66 (d, *J*=8.3 Hz, 1H), 3.47 (s, 3H), 3.41 (s, 3H), 2.98–2.79 (m, 3H), 2.02–1.85 (m, 6H), 1.69–1.53 (m, 6H), 1.48 (d, *J*=6.9 Hz, 6H), 1.35–1.17 (m, 20H), 1.16–1.12 (m, 2H). ^13^C NMR (151 MHz, C_6_D_6_): *δ*=150.5, 150.0, 148.6, 147.0, 146.8, 146.6, 140.0, 137.9, 137.8, 137.0 (137.00), 137.0 (136.97), 134.6, 132.4, 132.3, 131.3, 131.2, 128.6, 126.9, 120.8, 119.8, 112.7, 112.0, 55.6, 55.5, 35.0, 34.9, 34.8, 31.7, 31.6, 31.3 (31.28), 31.3 (31.26), 29.8, 29.7, 28.0, 27.9, 27.7, 27.6, 26.9, 26.5, 24.4, 23.4 (23.42), 23.4 (23.41). ^31^P NMR (122 MHz, C_6_D_6_): *δ*=−11.5. HRMS‐ESI (*m*/*z*): calcd for C_41_H_57_O_2_P: 613.4169 [*M*+H]^+^; found: 613.4166.

**28**: 0.5 equiv. aryl bromide was used; isolated in 77 % yield by washing with MeCN; ^1^H NMR (300 MHz, C_6_D_6_): *δ*=8.04 (s, 2H), 7.62 (d, *J*=8.2 Hz, 4H), 7.53 (d, *J*=8.4 Hz, 4H), 7.35–7.31 (m, 4H), 7.27 (s, 4H), 2.96–2.80 (m, 6H), 1.97–1.79 (m, 15H), 1.66–1.53 (m, 16H), 1.48 (d, *J*=6.8 Hz, 12H), 1.32–1.27 (m, 18H), 1.22–1.17 (m, 19H). ^13^C NMR (151 MHz, C_6_D_6_): *δ*=148.4, 147.5, 147.3, 146.5, 141.0, 138.3, 137.9, 137.8, 136.5, 136.4, 132.2 (132.24), 132.2 (132.18), 132.1, 130.6 (130.64), 130.6 (130.62), 127.1, 126.7, 122.6, 120.4, 90.6, 34.5 (34.50), 34.5 (34.46), 34.4, 31.3, 31.2, 30.9, 29.4, 29.3, 27.6, 27.5, 27.2 (27.22), 27.2 (27.16), 26.4, 26.1, 24.1, 23.0 (23.03), 23.0 (23.02). ^31^P NMR (122 MHz, C_6_D_6_): *δ*=−11.9. HRMS‐ESI (*m*/*z*): calcd for C_80_H_104_P_2_: 1127.7686 [*M*+H]^+^; found: 1127.7683.

**Formation of borane adduct**: The synthesis of **29** from **1** was performed according to a modified literature procedure^[30]^ (see the Supporting Information).

**29**: ^1^H NMR (300 MHz, C_6_D_6_): *δ*=8.68 (dd, *J*=8.6, 1.2 Hz, 1H), 8.09 (dt, *J*=7.6, 1.4 Hz, 1H), 7.31 (dd, *J*=7.6, 3.5 Hz, 1H), 7.21 (s, 2H), 2.91 (hept, *J*=6.9 Hz, 1H), 2.67 (hept, *J*=6.7 Hz, 2H), 2.19–2.01 (m, 2H), 1.99–1.76(m, 4H), 1.76–1.36 (m, 16H), 1.31 (d, *J*=6.9 Hz, 6H), 1.08 (s, 12H), 1.05 (d, *J*=6.7 Hz, 6H), 1.02–0.88 (m, 6H). ^13^C NMR (75 MHz, C_6_D_6_): *δ*=151.4, 151.3, 149.6, 146.8, 140.6, 140.5, 136.9 (136.92), 136.9 (136.89), 136.7, 136.6, 134.7, 134.5, 121.0, 84.5, 36.0, 35.5, 35.2, 31.6, 28.5, 28.3, 27.8, 27.7, 27.5, 27.3, 26.7, 26.5, 25.2, 24.8, 23.0. ^31^P NMR (122 MHz, C_6_D_6_): *δ*=29.3. ^11^B NMR (96 MHz, C_6_D_6_): *δ*=34.6, −41.49. HRMS‐ESI (*m*/*z*): calcd for C_39_H_63_B_2_O_2_P: 639.4644 [*M*+Na]^+^; found: 639.4647.

**Crystal structures**: Deposition Numbers 2174990 (for **1**), 2174988 (for **18**), 2174989 (for oxidation product of **19**) contain the supplementary crystallographic data for this paper. These data are provided free of charge by the joint Cambridge Crystallographic Data Centre and Fachinformationszentrum Karlsruhe Access Structures service.

## Conflict of interest

The authors declare no conflict of interest.

1

## Supporting information

As a service to our authors and readers, this journal provides supporting information supplied by the authors. Such materials are peer reviewed and may be re‐organized for online delivery, but are not copy‐edited or typeset. Technical support issues arising from supporting information (other than missing files) should be addressed to the authors.

Supporting Information

## Data Availability

The data that support the findings of this study are available in the supplementary material of this article.
