# Supplementary material for: Sterically Controlled Late‐Stage Functionalization of Bulky Phosphines
Source: Chemistry. 2022 Aug 1;28(51):e202202074. doi: 10.1002/chem.202202074 (PMC9544633; doi:10.1002/chem.202202074)
Supplement: Supplementary file 1 — Supporting Information [file CHEM-28-0-s001.pdf]

Chemistry–A European Journal

Supporting Information

Sterically Controlled Late-Stage Functionalization of Bulky Phosphines

Hao Deng, Marco Bengsch, Nico Tchorz, and Constanze N. Neumann*

General Information 3
Instruments 3
Chemicals 3
Initial optimization of phosphine borylation 4
Effect of the presence of **L** on borylation of phosphines 5
Substrates yielding mixtures of regioisomers 6
Reported Ir-catalyzed borylation reactions of phosphines 7
Attempted reduction of GPhos phosphine oxide 7
Attempted borylation of trifurylphosphine 8
Borylation of diphosphine substrates 9
Attempted monoborylation of **6** at lower temperature 9
Attempted monoborylation of **7** at lower temperature 10
Irreversible catalyst inhibition during synthesis of **6** at lower temperature 11
Suzuki coupling of 1-chloro-4-fluorobenzene 12
Experimental Procedures 15
Dicyclohexyl(2',4',6'-triisopropyl-4-(4,4,5,5-tetramethyl-1,3,2-dioxaborolan-2-yl)-[1,1'-biphenyl]-2-yl)phosphane (Borylated XPhos, **1**) 15
Dicyclohexyl(2',6'-diisopropoxy-4,4'-bis(4,4,5,5-tetramethyl-1,3,2-dioxaborolan-2-yl)-[1,1'-biphenyl]-2-yl)phosphane (Borylated RuPhos, **2**) 16
(3-(tert-butoxy)-2',6'-diisopropyl-6-methoxy-4'-(4,4,5,5-tetramethyl-1,3,2-dioxaborolan-2-yl)-[1,1'-biphenyl]-2-yl)dicyclohexylphosphane (Borylated GPhos, **3**) 17
4-(2-(((1r,3R,5S)-adamantan-1-yl)(adamantan-1-yl)phosphaneyl)-5-(4,4,5,5-tetramethyl-1,3,2-dioxaborolan-2-yl)phenyl)morpholine (Borylated MorDalPhos, **4**) 18
2'-(dicyclohexylphosphaneyl)-N^2,N^2,N^6,N^6-tetramethyl-4,4'-bis(4,4,5,5-tetramethyl-1,3,2-dioxaborolan-2-yl)-[1,1'-biphenyl]-2,6-diamine (Borylated CPhos, **5**) 19
1-(2,6-bis(cyclohexyloxy)-4-(4,4,5,5-tetramethyl-1,3,2-dioxaborolan-2-yl)phenyl)-2-(di-tert-butylphosphaneyl)-4-(4,4,5,5-tetramethyl-1,3,2-dioxaborolan-2-yl)-1H-imidazole (**6**) 20
Tris(2,6-dimethoxy-4-(4,4,5,5-tetramethyl-1,3,2-dioxaborolan-2-yl)phenyl)phosphane (**7**) 21
Borylation of [1,1'-binaphthalen]-2-yldi-tert-butylphosphane (8) 22
Borylation of 4-(anthracen-9-yl)-3-(tert-butyl)-2,3-dihydrobenzo[d][1,3]oxaphosphole (9) 22
Borylation of tri(naphthalen-1-yl)phosphane (10) 22
Diphenyl(2'-(4,4,5,5-tetramethyl-1,3,2-dioxaborolan-2-yl)-[1,1'-biphenyl]-2-yl)phosphane (Borylated PhJohnPhos, **12**) 23

(5-methyl-2-(4,4,5,5-tetramethyl-1,3,2-dioxaborolan-2-yl)phenyl)di-m-tolylphosphane (**13**) 24

(5-chloro-2-(4,4,5,5-tetramethyl-1,3,2-dioxaborolan-2-yl)phenyl)bis(3-chlorophenyl)phosphane (**14**) and (3-chloro-2-(4,4,5,5-tetramethyl-1,3,2-dioxaborolan-2-yl)phenyl)bis(3-chlorophenyl)phosphane (**15**) 25

Borylation of [1-(Diphenylphosphino)ethyl]ferrocene (16) 27

2-(dicyclohexylphosphaneyl)-2',4',6'-triisopropyl-[1,1'-biphenyl]-4-ol (**19**) 28

Dicyclohexyl(2',4',6'-triisopropyl-4-methoxy-[1,1'-biphenyl]-2-yl)phosphane (**20**) 30

3'-(dicyclohexylphosphaneyl)-2'',4'',6''-triisopropyl-N,N-dimethyl-[1,1':4',1''-terphenyl]-3-amine (**22**) 32

3-(3'-(dicyclohexylphosphaneyl)-2'',4'',6''-triisopropyl-[1,1':4',1''-terphenyl]-3-yl)-5-ethyl-1,2,4-oxadiazole (**23**) 33

8-(2-(dicyclohexylphosphaneyl)-2',4',6'-triisopropyl-[1,1'-biphenyl]-4-yl)quinolone (**24**) 34

Dicyclohexyl(2'',3'',4'',5'',6''-pentafluoro-2,4,6-triisopropyl-[1,1':4',1''-terphenyl]-2'-yl)phosphane (**25**) 35

Dicyclohexyl(2',4',6'-triisopropyl-4-(thiophen-2-yl)-[1,1'-biphenyl]-2-yl)phosphane (**26**) 36

Dicyclohexyl(2,4,6-triisopropyl-3'',4''-dimethoxy-[1,1':4',1''-terphenyl]-2'-yl)phosphane (**27**) 37

1,2-bis(3'-(dicyclohexylphosphaneyl)-2'',4'',6''-triisopropyl-[1,1':4',1''-terphenyl]-4-yl)ethyne (**28**) 38

Borane adduct of borylated XPhos (**29**) 39

X-ray structures 40

X-ray structure of **1** 40

X-ray structure of **18** 52

X-ray structure of oxidation product of **19** 56

NMR spectra of isolated compounds 66

References 144

General Information

Instruments

Mass Spectrometry (MS): A QExactive instrument from Thermo Fisher Scientific with direct injection to the sprayer was used to collect ESI measurements.

Nuclear Magnetic Resonance (NMR) Spectroscopy: NMR data were recorded using a Bruker AVIII HD 300 MHz, Bruker AVIII HD 400 MHz, or Bruker AVNeo 600 MHz NMR spectrometer. ^{1}H and ^{13}C chemical shifts are referenced to the deuterated solvent as internal standard. Determination of which position underwent borylation was made based on ^{1}H, ^{13}C, [^{1}H, ^{1}H]-COSY, [^{13}C, ^{1}H]-HSQC, [^{13}C, ^{1}H]-HMBC, [^{31}P, ^{1}H]-HMBC, [^{15}N, ^{1}H]-HMBC and [^{1}H, ^{1}H]-NOESY NMR spectra of isolated compounds (and confirmed by single crystal X-ray crystallography in the case of **1**).

Single crystal X-ray diffraction (SC-XRD): SC-XRD data were recorded on Bruker AXS Enraf-Nonius KappaCCD diffractometer with a FR591 rotating Mo-anode X-ray source and a Bruker-AXS Kappa Mach3 with APEX-II detector and IµS microfocus Mo-anode X-ray source.

Flash column chromatography: VWR silica gel (40-63 µm) was used.

Chemicals

Unless otherwise specified, chemicals were obtained from commercial suppliers and used as received.

For NMR analysis of air-sensitive samples, benzene-d_6 was stored over 4 Å molecular in an argon glovebox. The 4 Å molecular sieves were activated at 300 °C under dynamic vacuum (5×10^{-6} mbar) for 3 days prior to use.

Pd catalyst **21**[24], GPhos[25], and $[Ir(COD)OH]_2$[26] were synthesized according to literature procedures. Other reagents were purchased from the suppliers listed in Table S1.

**Chemical Name**	**Manufacturer**
4,4′-Di-tert-butyl-2,2′-dipyridyl (**L1**)	Sigma-Aldrich Chemie GmbH
(1,5-Cyclooctadiene)(methoxy)iridium(I) dimer	
Di(1-adamantyl)-2-morpholinophenylphosphine (MorDalphos)	
2-Dicyclohexylphosphino-2′,6′-bis(N,N-dimethylamino)biphenyl (CPhos)	
Tris(2,6-dimethoxyphenyl)phosphine	
Iodopentafluorobenzene	
2-Bromothiophene	
4-Bromoveratrol	
3,4,7,8 -tetramethyl-1,10-phenanthroline (**L2**)	TCI Deutschland GmbH
Tri(2-furyl)phosphine	COMBI-BLOCKS
Tri-m-tolylphosphine	
Dicyclohexyl[2-(2,4,6-triisopropylphenyl)phenyl]phosphane (XPhos)	
Bis(pinacolato)diboron (B_2Pin_2)	

2-Dicyclohexylphosphino-2',6'-di-i-propoxy-1,1'-biphenyl (RuPhos)	
3-(3-Bromophenyl)-5-ethyl-1,2,4-oxadiazole	
8-Bromoquinoline	Fisher Scientific GmbH
Bis(4-bromophenyl)acetylene	
n-Octane (anhydrous)	
1-[2,6-Bis(cyclohexyloxy)phenyl]-2-(di-tertbutylphosphanyl)-1H-imidazole	ABCR GmbH
3-Bromo-N,N-dimethylaniline	Chempur Feinchemikalien

Table S1. Reagents obtained from commercial suppliers.

Initial optimization of phosphine borylation

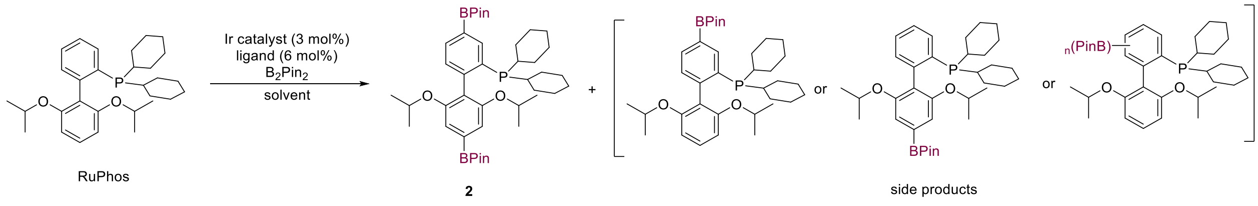

Entry	Catalyst	Ligand	B_2Pin_2(equiv)	Solvent	Time	SM : **2** : (side products) [a]
1	$[Ir(COD)Cl]_2$	neocuprine	1.1	THF	2d	\
2	$[Ir(COD)Cl]_2$	neocuprine	1.1	*n*-octane	2d	\
3	$[Ir(COD)OH]_2$	neocuprine	1.1	*n*-octane	2d	20 : 51 : (21 : 5 : 3)
4	$[Ir(COD)OH]_2$	**L1**	1.1	*n*-octane	2d	22 : 43 : (29 : 3 : 3)
5	$[Ir(COD)OH]_2$	**L1**	1.1	THF	2d	17 : 47 : (28 : 3 : 5)
6	$[Ir(COD)OMe]_2$	neocuprine	1.1	THF	1d	42 : 37 : (15 : 5 : 1)
7	$[Ir(COD)OMe]_2$	**L1**	1.1	THF	3d	11 : 51 : (27 : 3 : 8)
8	$[Ir(COD)OMe]_2$	**L1**	1.5	THF	3d	1 : 69 : (10 : 4 : 16)
9	$[Ir(COD)OMe]_2$	**L1**	1.5	THF	1d	6 : 62 : (22 : 2 : 8)
10	$[Ir(COD)OMe]_2$	**L1**	2.2	THF	1d	0 : 91 : (0 : 0 : 9)

Table S2. Initial optimization. Reaction condition: RuPhos (0.06 mmol, 1.00 equiv), B_2Pin_2, Ir catalyst (3 mol%) and ligand (6 mol%) in solvent (0.8 ml) at 78 °C under an argon atmosphere; [a] Ratio determined by ^{31}P NMR.

Promising results could be obtained with either $[Ir(COD)OH]_2$ or $[Ir(COD)OMe]_2$. Due to the commercial availability of $[Ir(COD)OMe]_2$, further optimization was carried out with $[Ir(COD)OMe]_2$. Because characterization by NMR spectroscopy and mass spectrometry revealed that major isomer **2** was a diborylated compound, we increased the amount of B_2Pin_2 from 1.1 equiv to 2.2 equiv after which high conversion to **2** could be achieved (Table S2, entry 10).

Effect of the presence of **L** on borylation of phosphines

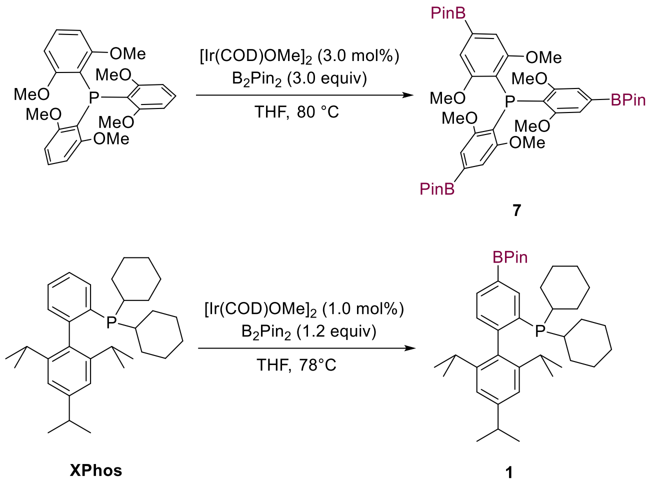

Ligand	Yield[a]
**L2** (6 mol%)	92%
–	n.d.
**L1** (2 mol%)	79%
–	n.d.

Scheme S1. Comparison of undirected iridium-catalyzed borylation reactions carried out in the presence and absence of diamine ligand. [a] Determined by ^{31}P NMR; n.d. = not detected.

Unlike substrates undergoing sterically controlled borylation reactions, such as those yielding **1** or **7** (Scheme S1), comparable (**12**) or increased (**13**, **14** and **15**) rates of borylation were observed in the absence of added diamine ligand for substrates undergoing phosphine directed borylation reactions. Isolated **13**, **14** and **15** were nonetheless obtained from reactions carried out in the presence of **L2** because the more efficient borylation reaction taking place in the absence of ligand could not be arrested at a point where all starting material had been consumed and little to no diborylation had occurred.

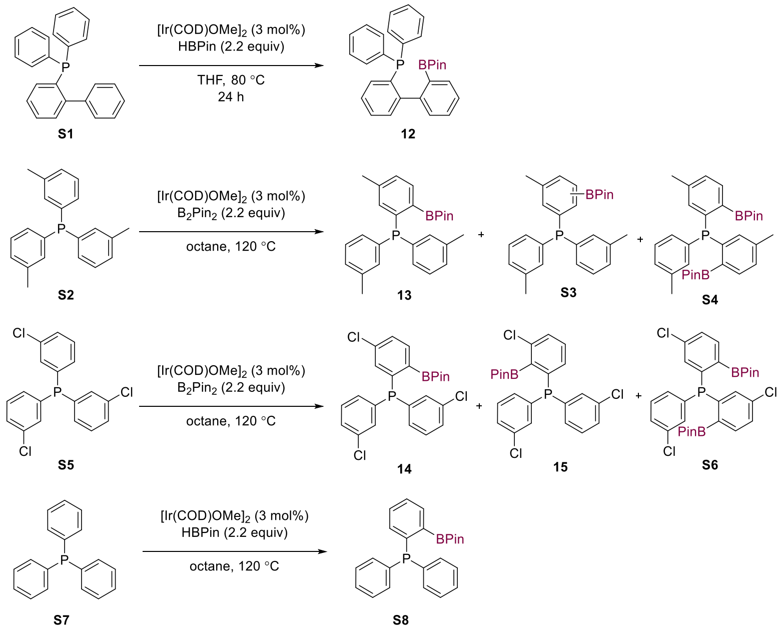

Ligand	time	ratio determined by ^{31}P NMR
**L2** (6 mol%)	24 h	**S1** :**12** = 50 : 50
–	24 h	**S1** : **12** = 50 : 50
**L2** (6 mol%)	24 h	**S2** : **13** : **S3** : **S4** = 0 : 95 : 5 : 0
–	17 h	**S2** : **13** : **S3** : **S4** = 0 : 5: 8 : 87
**L2** (6 mol%)	24 h	**S5** : **14** : **15** : **S6** = 66 : 32 : 2 : 0
**L2** (6 mol%)	32 h	**S5** : **14** : **15** : **S6** = 0 : 87 : 10 : 3
–	17 h	**S5** : **14** : **15** : **S6** = 0 : 67 : 13 : 20
–	40 h	**S5** : **14** : **15** : **S6** = 0 : 43 : 9 : 48
**L2** (6 mol%)	17 h	**S7** : **S8** = 83 : 17
**L2** (6 mol%)	40 h	**S7** : **S8** = 81 : 19
-	17 h	**S7** : **S8** = 83 : 17
-	40 h	**S7** : **S8** = 81 : 19

Scheme S2. Comparison of iridium-catalyzed borylation reactions of triarylphosphines carried out in the presence and absence of **L2**. Results highlighted in blue correspond to the reaction conditions employed for the isolation of analytically pure **12**, **13**, **14** and **15** (see Scheme 2).

Substrates yielding mixtures of regioisomers

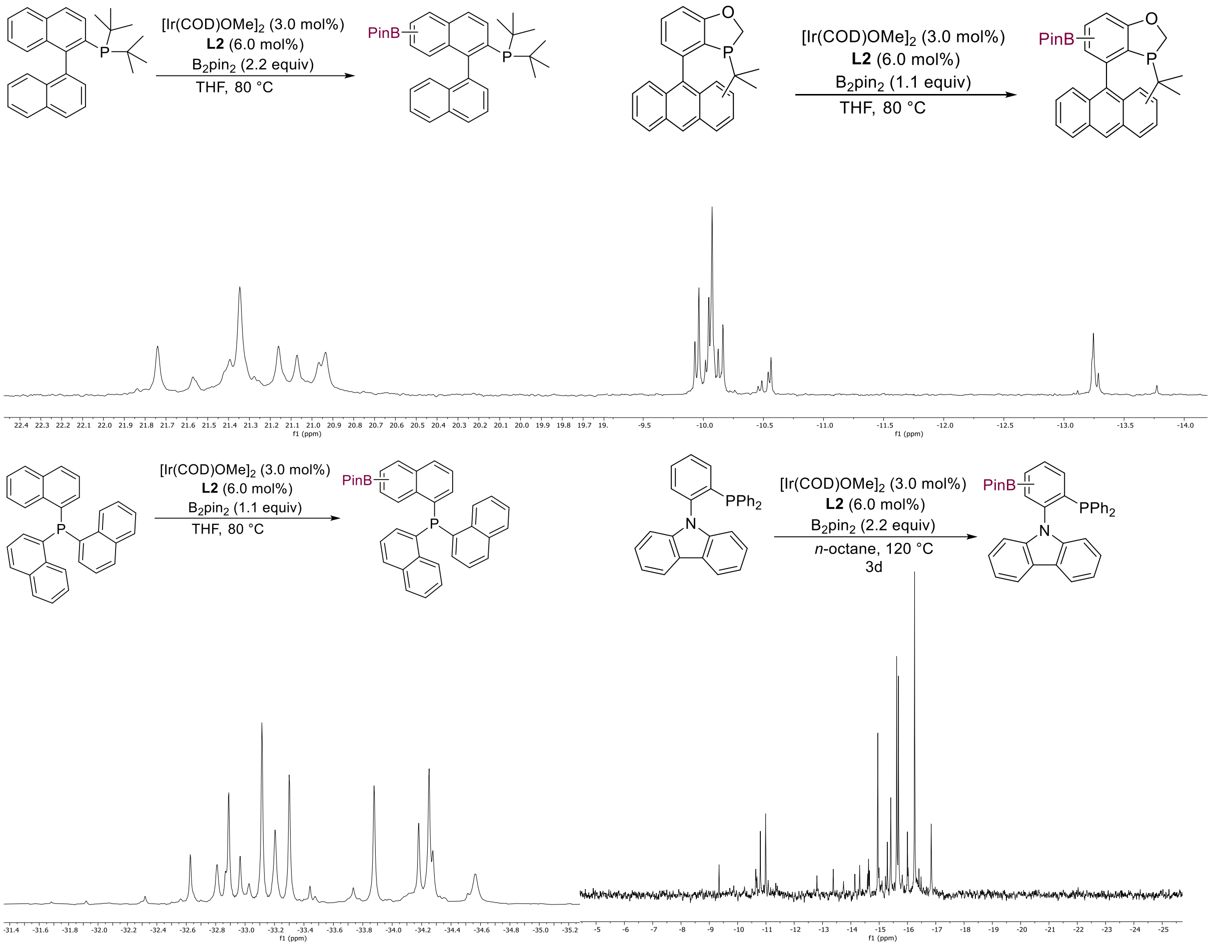

Scheme S3. ^{31}P NMR spectra of substrates for which many different borylated isomers were obtained.

The borylation of phosphines containing a large number of C–H bonds that are not sterically shielded from undergoing iridium catalyzed functionalization yielded intractable mixtures of borylated products. Based on ^{31}P NMR and high-resolution mass spectrometry, we concluded that the reaction mixtures consisted of different isomers containing one, two or three BPin substituents. Isolation of single regioisomers from the mixtures was not attempted.

Reported Ir-catalyzed borylation reactions of phosphines

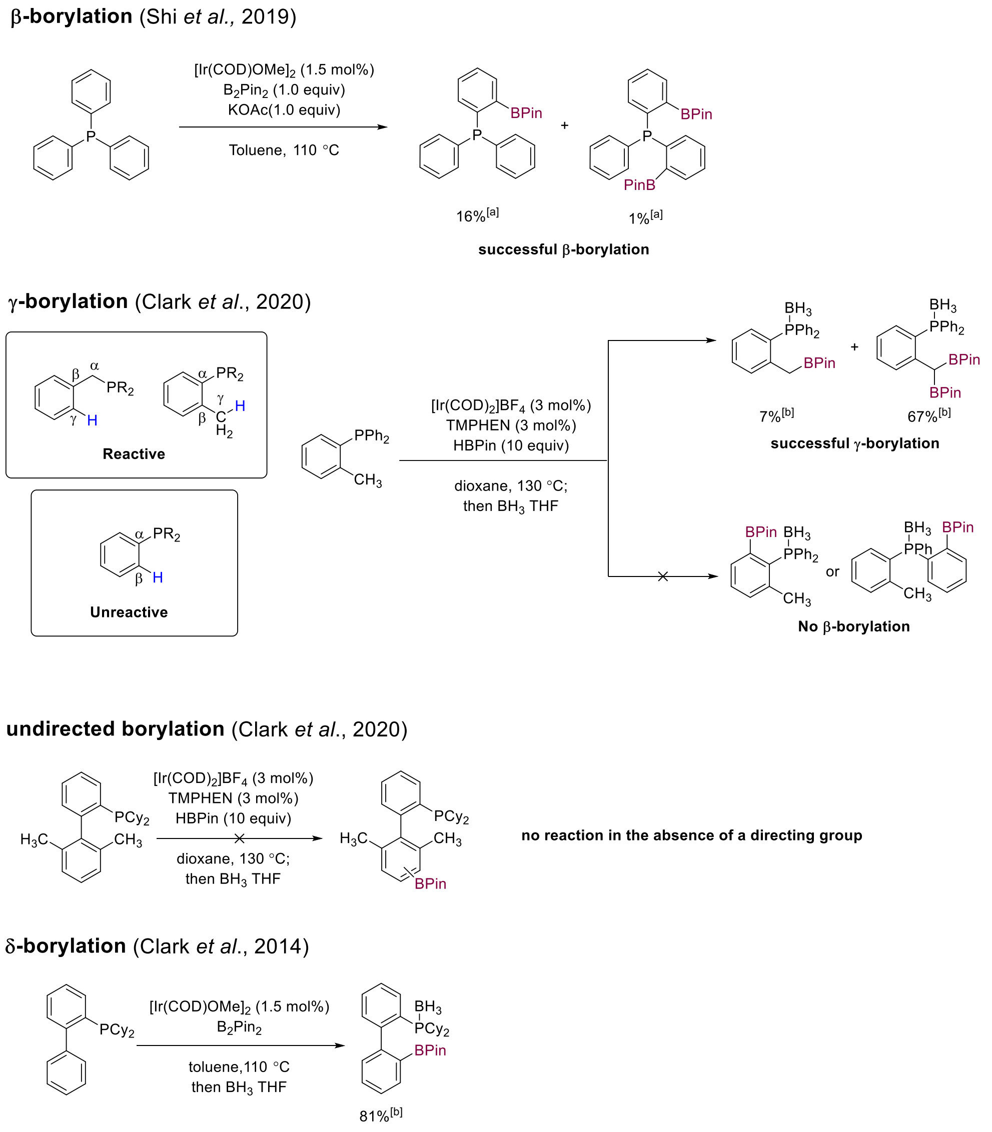

Scheme S4. References: Shi *et al*. 2019[28], Clark *et al.* 2020[29], Clark *et al.* 2014[30]. [a] Determined by GC; [b] Isolated yield.

Attempted reduction of GPhos phosphine oxide

Reduction of phosphine oxides to phosphines can be challenging especially for sterically encumbered biaryl phosphines. In our hands, common conditions[27] for phosphine oxide reduction proved unsuccessful for the synthesis of GPhos from the corresponding phosphine oxide (Figure S1).

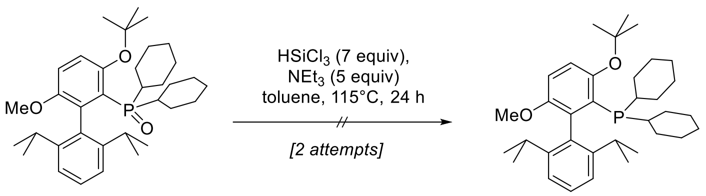

Scheme S5. Failed attempt at synthesizing GPhos from the corresponding phosphine oxide.

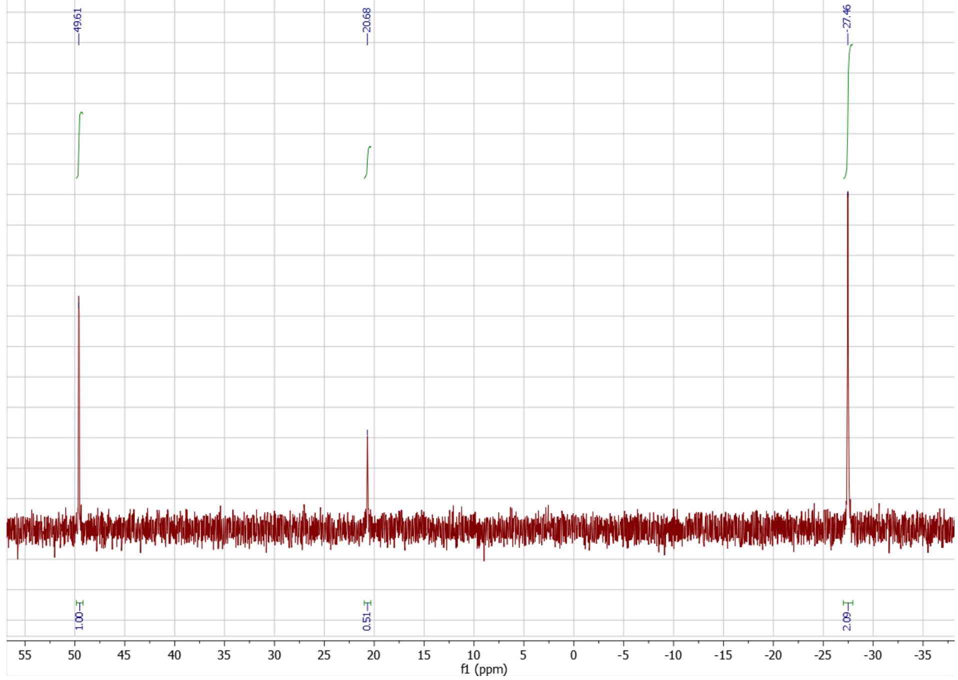

Figure S1. ^{31}P NMR spectrum obtained of the product of phosphine oxide reduction (see Figure S1). ^{31}P NMR shift measured for an authentic sample of GPhos: –4.6 ppm.

Attempted borylation of trifurylphosphine

Contrary to all other iridium-catalyzed borylation reactions carried out in THF, which resulted in clear solutions, colorless crystals were observed at the end of the attempted borylation of trifurylphosphine. The crystals were collected and subjected to single-crystal X-ray diffraction, which indictated the formation of **18**. Based on the residual electron density close to the iridium center and the arrangement of the phosphine ligands around iridium, the presence of a hydride ligand was suspected. We dissolved a portion of the crystals in DCM-d_2, but could not identify a signal corresponding to iridium hydride by ^1H NMR. IR spectroscopy (Figure S3) and mass spectrometric analysis of the DCM-solution of **18** confirmed the presence of a hydride ligand.

HRMS-ESI (m/z): Calculated for $C_{48}H_{37}IrO_{12}P_4$ $[M+H]^+$, 1123.0938 ; Found: 1123.0944.

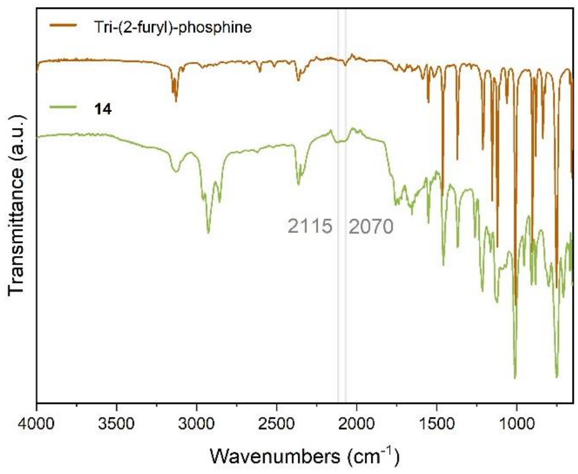

Figure S2. IR spectrum of trifurylphosphine as well as a DCM solution of iridium complex **18**.

Borylation of diphosphine substrates

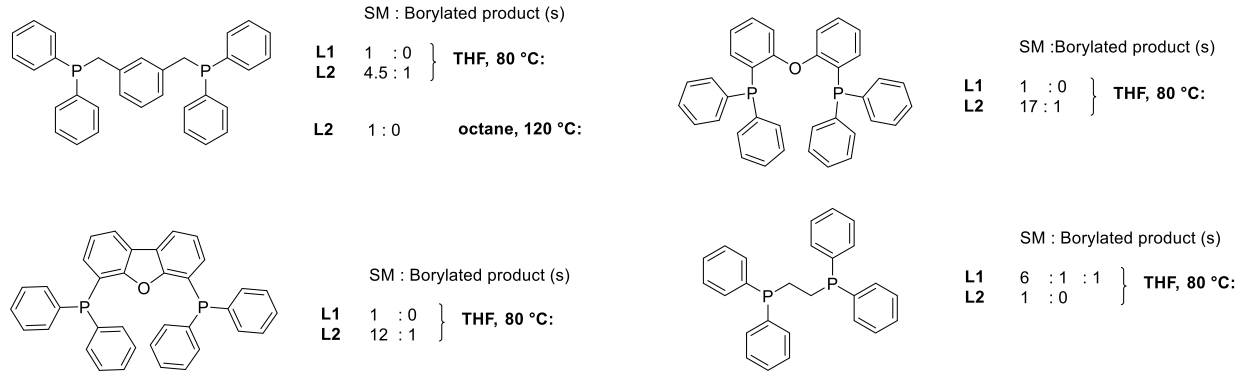

Scheme S6. Iridium-catalyzed borylation of substrates containing two phosphorous centers resulted in no or low conversion. Ratios of starting material (SM) and borylated products were determined by ^{31}P NMR.

Attempted monoborylation of **6** at lower temperature

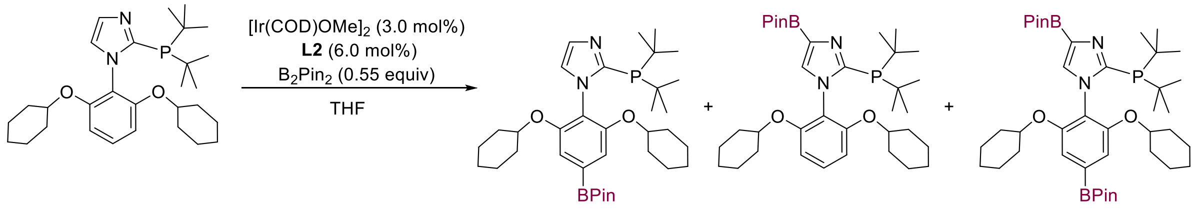

S9 **S10** **S11** **6**

Scheme S7. Attempted monoborylation of **6** at lower temperature with a reduced amount of B_2Pin_2.

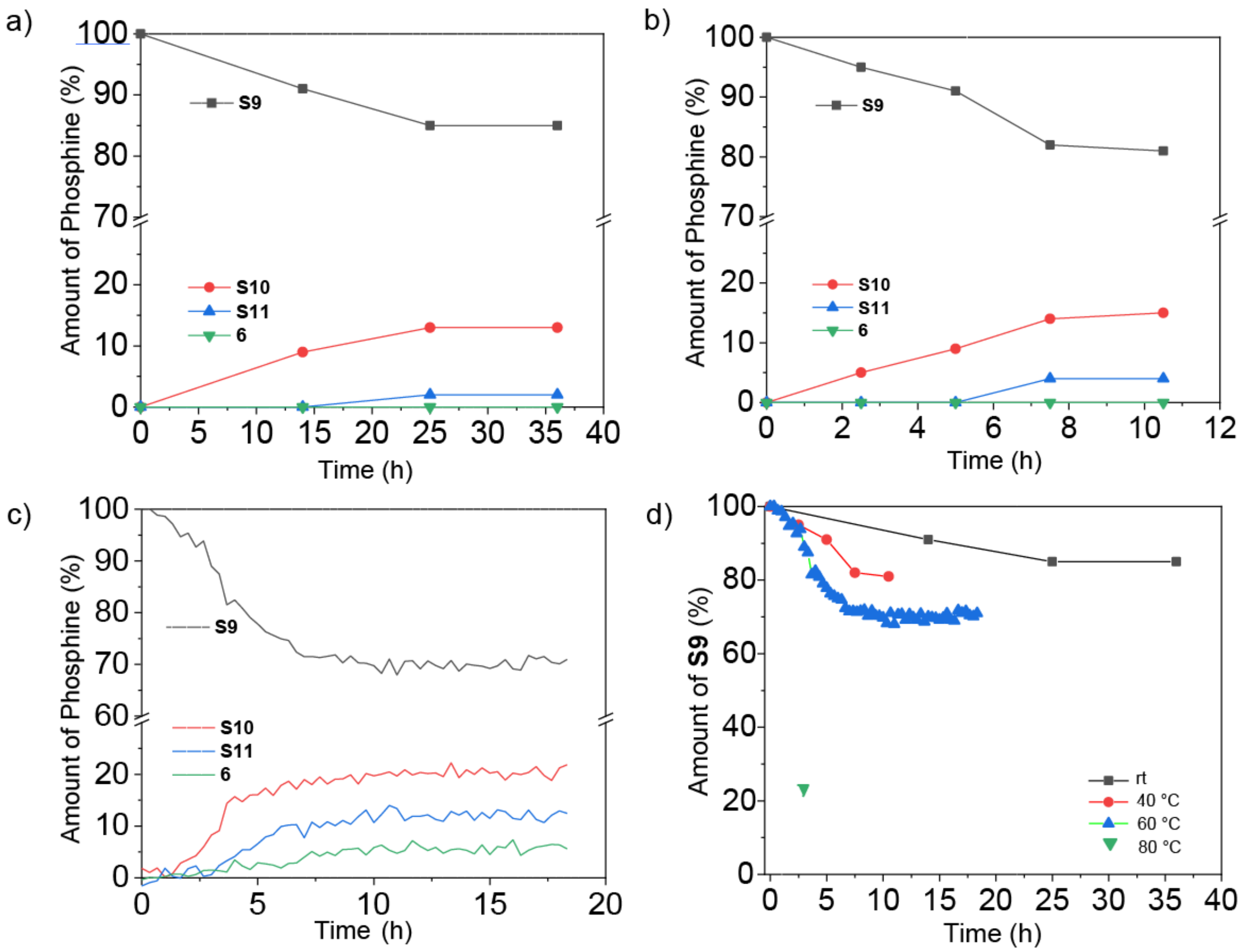

Figure S3. Iridium-catalyzed borylation of **S9** at a) 25 °C, b) 40 °C, c) 60 °C did not result in the selective formation of one monoborylated isomer. The rate of formation of **S11** was only slightly slower than that of **S10**, so neither monoborylated isomer could be obtained in high yield. d) The conversion of **S9** over time was monitored by ^{31}P NMR spectroscopy at different reaction temperatures. For temperatures of 60 °C or below, the reaction stalled at low conversion, whereas C–H borylation at 80 °C resulted in full conversion of the 0.55 equiv B_2Pin_2 added to the reaction mixture after 3 h resulting in **S9** : **S10** : **S11** : **6** = 23 : 38 : 13 : 27.

Attempted monoborylation of 7 at lower temperature

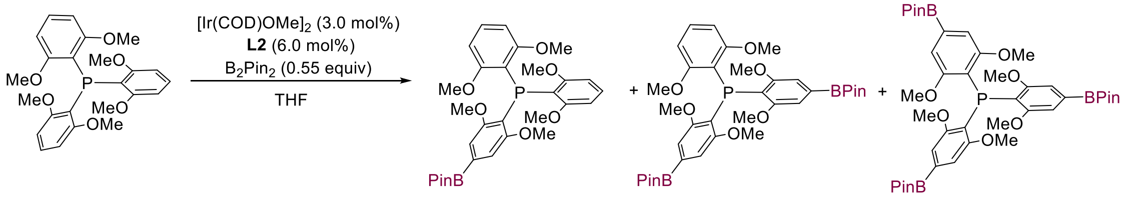

S12 **S13** **S14** **7**

Scheme S8. Attempted monoborylation of **7** at lower temperature with a reduced amount of B_2Pin_2.

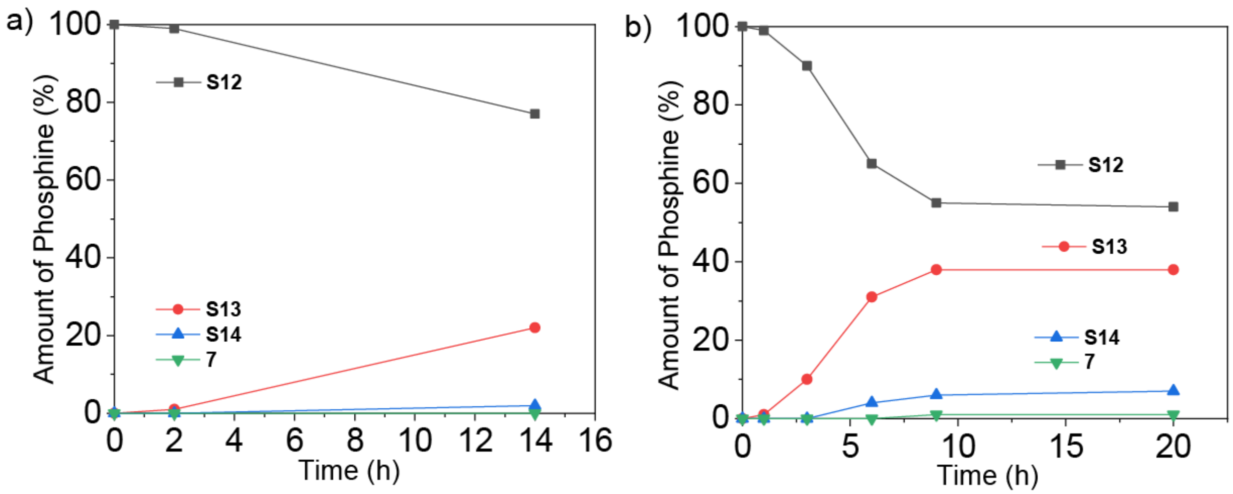

Figure S4. Iridium-catalyzed borylation of **S12** was carried out at a) 25 °C and b) 40 °C. The diborylated product **S14** was formed with an appreciably slower rate compared with monoborylated product **S13** so that synthesis of monoborylated **S13** in a reasonable yield may be possible. We did not attempt to isolate pure **S13**.

Irreversible catalyst inhibition during synthesis of 6 at lower temperature

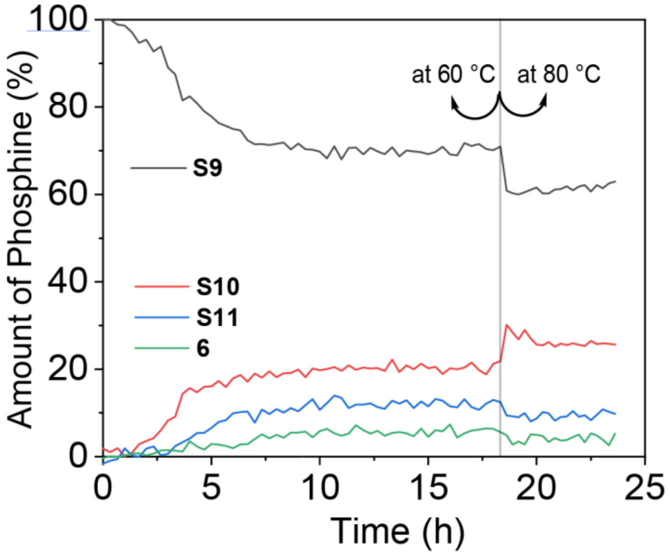

Figure S5. After 18 h, Iridium-catalyzed borylation of **S9** at 60 °C did not show any further conversion. After increasing the reaction temperature from 60 °C to 80 °C, only a slight increase in conversion was observed before the reaction stalled once more (compare with Figure S3).

Suzuki coupling of 1-chloro-4-fluorobenzene

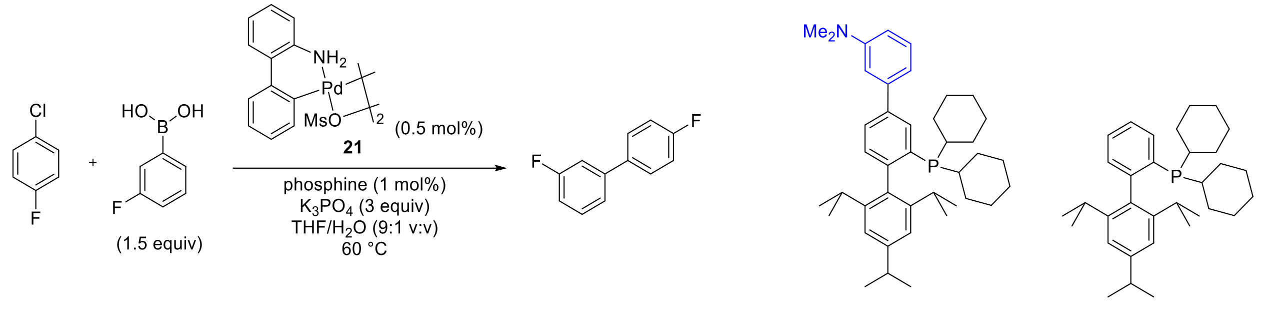

Two Suzuki reactions were set up side-by-side as described below, for which phosphine **22** was used in one reaction and XPhos was used in the second reaction.

1-chloro-4-fluorobenzene (100 mg, 0.766 mmol, 1.00 equiv), 3-fluorophenylboronic acid (161 mg, 1.15 mmol, 1.50 equiv), K_3PO_4 (488 mg, 2.30 mmol, 3.00 equiv), THF (1.8 mL) and water (0.2 mL) were added to a Schlenk flask containing a stir bar. The biphasic reaction mixture was purged with argon for 15 minutes before the palladium catalyst (**21**, 2.8 mg, 3.8 µmol, 0.50 mol%) and the respective phosphine ligand (7.7 µmol, 1.0 mol%) were added. The biphasic solution was purged with argon for an additional 5 minutes. The reaction mixture was stirred at 60 °C for 2 hours before heating and stirring were temporarily paused to remove a small aliquot of the organic phase to monitor reaction progress. Heating and stirring was then resumed and further aliquots were removed from the reaction mixture at stated intervals (Figure S8). Whenever an aliquot was removed it was added to an NMR tube containing acetone (0.45 mL) and acetone-d^6 (0.05 mL) and a ^{19}F NMR was collected. The extent of conversion of 1-chloro-4-fluorobenzene to the biaryl product was monitored by integration of the ^{19}F NMR signals of the respective species.

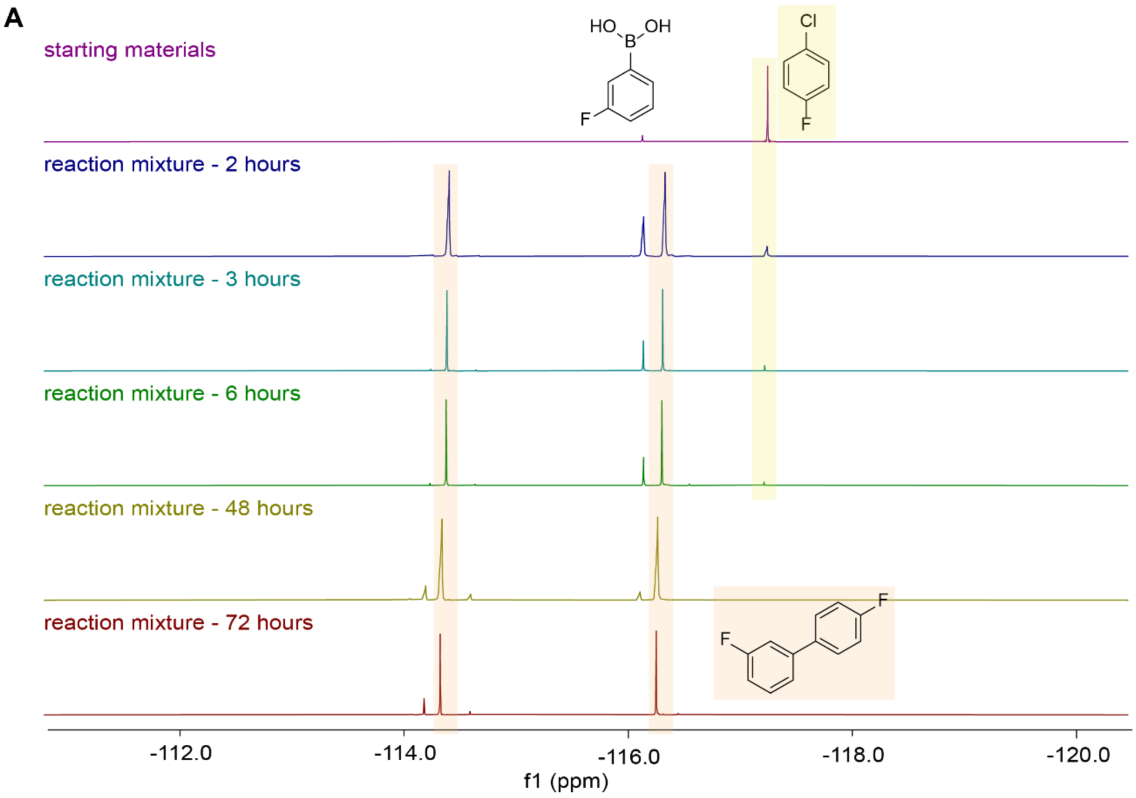

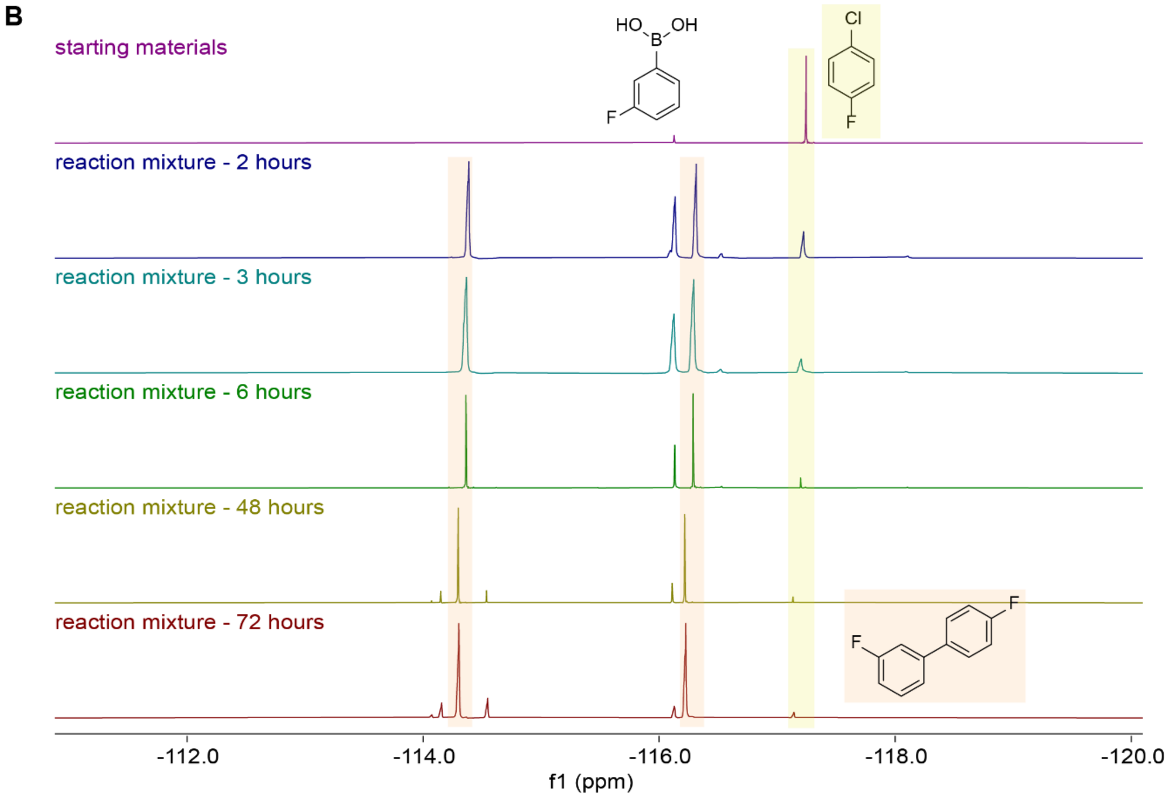

Figure S6. ^{19}F NMR spectra of aliquots of the Suzuki reaction of 1-chloro-4-fluorobenzene with 3-fluoroboronic acid removed at stated intervals from reaction mixtures containing **22** (**A**) and XPhos (**B**).

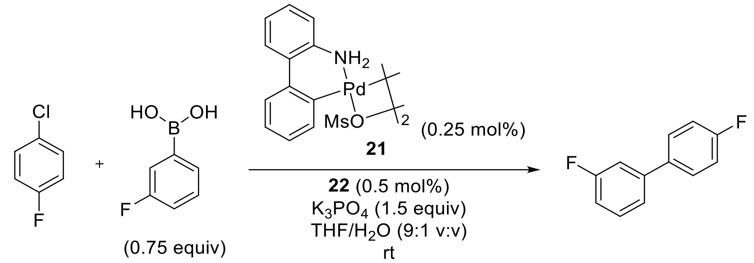

1-chloro-4-fluorobenzene (200 mg, 1.53 mmol, 1.00 equiv), 3-fluorophenylboronic acid (161 mg, 1.15 mmol, 0.75 equiv), K_3PO_4 (488 mg, 2.30 mmol, 1.50 equiv), THF (1.8 mL) and water (0.2 mL) were added to a Schlenk flask containing a stir bar. The biphasic reaction mixture was purged with argon for 15 minutes before the palladium catalyst (**21**, 2.8 mg, 3.8 µmol, 0.25 mol%) and **22** (7.7 µmol, 0.5 mol%) were added. The biphasic solution was purged with argon for an additional 5 minutes. The reaction mixture was stirred at room temperature, and small aliquots of the organic phase were removed at stated intervals to monitor reaction progress (Table S3). Whenever an aliquot was removed it was added to an NMR tube containing acetone (0.45 mL) and acetone-d^6 (0.05 mL) and a ^{19}F NMR was collected. The extent of conversion of 1-chloro-4-fluorobenzene to the biaryl product was monitored by integration of the ^{19}F NMR signals of the respective species.

Time (h)	Ratio of biaryl product to 1-chloro-4-fluorobenzene
2	0.35 : 1.00
3	0.55 : 1.00
5	0.62 : 1.00

Table S3. Time course for the cross coupling of 1-chloro-4-fluorobenzene with 3-fluorophenylboronic acid at room temperature (ratio of starting material and product were determined by integration of the respective ^{19}F NMR signals).

Experimental Procedures

*Dicyclohexyl(2',4',6'-triisopropyl-4-(4,4,5,5-tetramethyl-1,3,2-dioxaborolan-2-yl)-[1,1'-biphenyl]-2-yl)phosphane (Borylated XPhos, **1**)*

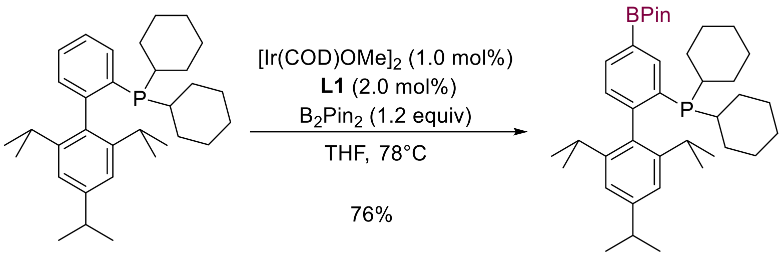

XPhos **1 (XPhos-Bpin)**

In an argon glovebox, 2-dicyclohexylphosphino-2′,4′,6′-triisopropylbiphenyl (XPhos, 2.000 g, 4.200 mmol, 1.000 equiv), $[Ir(COD)OMe]_2$ (27.8 mg, 42.0 µmol, 1.00 mol%), 4,4'-di-tert.-butyl-2,2'-dipyridyl (**L1**, 22.5 mg, 84.0 µmol, 2.00 mol%), B_2Pin_2 (1.280 g, 5.000 mmol, 1.200 equiv) and THF (41 ml) were added to a 60 ml Schlenk flask. The resulting suspension was heated at 78 °C outside the glovebox. After 24 h, the reaction mixture was left to cool to room temperature and concentrated, after which acetonitrile (150 mL) was added and the suspension was subjected to sonication for 5 min. Filtration over a medium frit gave rise to a white to slightly pink solid, which was washed with additional MeCN (150 ml). The resulting solid was dried under vacuum to afford the title compound as a white solid (1.93 g, 76% yield).

Note: **1** can be stored at room temperature under air in the solid state (no decomposition or oxidation observed over the course of 3 months), but it is liable to undergo oxidation to the phosphine oxide in solution.

NMR Spectroscopy:

^{1}H NMR (600 MHz, C_6D_6) δ 8.35 (s, 1H), 7.95 (d, J = 7.6 Hz, 1H), 7.16 (dd, J = 7.6, 3.7 Hz, 1H), 7.04 (s, 2H), 2.69 (hept, J = 6.9 Hz, 1H), 2.58 (hept, J = 6.9 Hz, 2H), 1.80 – 1.69 (m, 4H), 1.67 – 1.58 (m, 2H), 1.46 – 1.34 (m, 6H), 1.24 (d, J = 6.8 Hz, 6H), 1.12 – 1.00 (m, 12H), 0.96 – 0.81 (m, 22H).

^{13}C NMR (151 MHz, C_6D_6) δ 151.8, 151.6, 148.5, 146.5, 139.3 (139.32), 139.3 (139.30), 137.5, 137.4, 137.1, 137.0, 135.0, 131.7 (131.73), 131.7 (131.69), 128.6, 120.7, 83.9, 34.8, 34.7, 34.6, 31.7, 31.6, 31.2 (31.22), 31.2 (31.21), 29.9, 29.8, 27.9, 27.8, 27.5, 27.4, 26.8, 26.3, 25.0, 24.4, 23.4, 23.3.

^{31}P NMR (122 MHz, C_6D_6) δ –11.5.

HRMS-ESI (m/z): Calculated for $C_{39}H_{60}BO_2P$ $[M+H]^+$, 603.4497; Found: 603.4499.

*Dicyclohexyl(2',6'-diisopropoxy-4,4'-bis(4,4,5,5-tetramethyl-1,3,2-dioxaborolan-2-yl)-[1,1'-biphenyl]-2-yl)phosphane (Borylated RuPhos, **2**)*

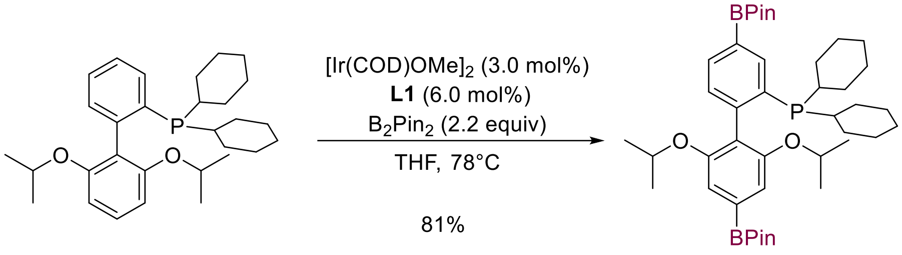

RuPhos **2 (RuPhos-Bpin)**

In an argon glovebox, 2-dicyclohexylphosphino-2',6'-diisopropoxybiphenyl (RuPhos, 400 mg, 0.857 mmol, 1.00 equiv), $[Ir(COD)OMe]_2$ (17.1 mg, 25.7 µmol, 3.00 mol%), 4,4'-Di-tert.-butyl-2,2'-dipyridyl (**L1**, 13.8 mg, 51.4 µmol, 6.00 mol%), B_2Pin_2 (479 mg, 1.89 mmol, 2.20 equiv) and THF (8 ml) were added to a Schlenk tube. The resulting suspension was heated at 78 °C outside the glovebox. After 24 h, the reaction mixture was left to cool to room temperature and concentrated. Recrystallization from methanol followed by drying of the resulting solid under vacuum afforded the title compound as a white solid (410 mg, 81% yield).

Note: **2** can be stored at room temperature under air in the solid state (no decomposition or oxidation observed over the course of 3 months), but it is liable to undergo oxidation to the phosphine oxide in solution.

NMR Spectroscopy:

^{1}H NMR (300 MHz, C_6D_6) δ 8.53 (t, *J* = 1.7 Hz, 1H), 8.20 (dt, *J* = 7.6, 1.1 Hz, 1H), 7.44 (s, 2H), 7.35 (dd, *J* = 7.6, 3.4 Hz, 1H), 4.28 (hept, *J* = 6.0 Hz, 2H), 2.08 – 1.79 (m, 6H), 1.70 – 1.56 (m, 6H), 1.34 – 1.10 (m, 21H), 1.08 – 0.98 (m, 19H), 0.87 (d, *J* = 6.0 Hz, 6H).

^{13}C NMR (151 MHz, C_6D_6) δ 156.5 (156.50), 156.5 (156.49), 148.5, 148.2, 139.1 (139.10), 139.1 (139.08), 137.3, 137.1, 134.8, 131.2, 131.1, 127.5, 127.4, 112.7, 83.8, 83.6, 70.2, 35.1, 35.0, 31.0, 30.9, 30.6, 30.5, 27.7, 27.6 (27.64), 27.6 (27.56), 27.1, 25.1 (25.10), 25.1 (25.05), 22.5, 22.2.

^{31}P NMR (122 MHz, C_6D_6) δ –8.8.

HRMS-ESI (m/z): Calculated for $C_{42}H_{65}B_2O_6P$ $[M+H]^+$, 719.4778; Found: 719.4781.

*(3-(tert-butoxy)-2',6'-diisopropyl-6-methoxy-4'-(4,4,5,5-tetramethyl-1,3,2-dioxaborolan-2-yl)-[1,1'-biphenyl]-2-yl)dicyclohexylphosphane (Borylated GPhos, **3**)*

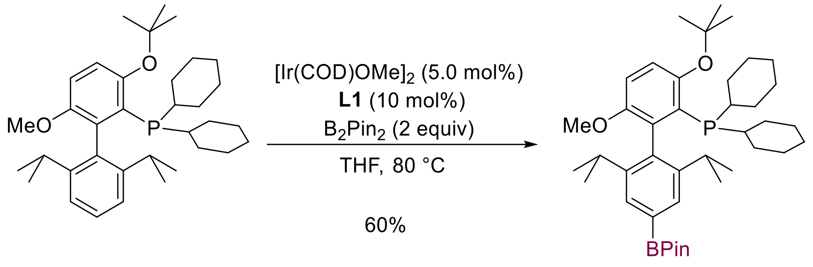

In an argon glovebox, (3-(tert-butoxy)-2′,6′-diisopropyl-6-methoxy-[1,1′-biphenyl]-2-yl)dicyclohexylphosphane (**GPhos**, 300 mg, 0.559 mmol, 1.00 equiv), $[Ir(COD)OMe]_2$ (18.5 mg, 27.9 µmol, 5.00 mol%), 4,4'-di-tert.-butyl-2,2'-dipyridyl (**L1**, 15 mg, 55.9 µmol, 10.0 mol%), B_2Pin_2 (284 mg, 1.12 mmol, 2.00 equiv) and THF (7 ml) were added to a Schlenk tube. The resulting suspension was heated at 80 °C outside the glovebox. After 72 h, the reaction mixture was left to cool to room temperature and concentrated. Methanol (5 ml) was added to the vial, which was closed with a septum cap and flushed with argon. The vial was sonicated for 5 min subjected to centrifugation (5 min, 4500 rpm), after which the supernatant was removed using a syringe. The washing process was repeated 3 times, using methanol (5 mL) each time. The resulting solid was dried under vacuum to afford the title compound as a white solid (224 mg, 60% yield).

NMR Spectroscopy:

^{1}H NMR (600 MHz, C_6D_6) δ 8.23 (s, 2H), 6.70 (d, *J* = 9.0 Hz, 1H), 6.46 (d, *J* = 9.0 Hz, 1H), 3.16 (s, 3H), 2.83 (hept, *J* = 6.8 Hz, 2H), 2.45 (tt, *J* = 12.1, 3.2 Hz, 2H), 2.02 – 1.92 (m, 2H), 1.79 – 1.67 (m, 6H), 1.66 – 1.60 (m, 2H), 1.44 (d, *J* = 6.8 Hz, 6H), 1.41 (s, 9H), 1.38 – 1.17 (m, 10H), 1.15 (d, *J* = 6.7 Hz, 6H), 1.13 (s, 12H).

^{13}C NMR (151 MHz, C_6D_6) δ 153.1 (153.14), 153.1 (153.12), 151.5, 151.4, 146.5, 146.4, 140.4, 140.2, 139.9 (139.93), 139.9 (139.87), 129.6, 127.3, 127.1, 122.6, 112.7, 110.6, 83.4, 77.1, 54.1, 38.3, 38.2, 34.0, 33.9, 30.9, 30.8 (30.84), 30.8 (30.80), 29.0, 28.5 (28.53), 28.5 (28.49), 28.1, 28.0, 26.9, 25.6, 25.1, 24.1.

^{31}P NMR (243 MHz, C_6D_6) δ –4.5.

HRMS-ESI (m/z): Calculated for $C_{41}H_{64}BO_4P$ $[M+H]^+$, 663.4708; Found: 663.4713.

*4-(2-(((1r,3R,5S)-adamantan-1-yl)(adamantan-1-yl)phosphaneyl)-5-(4,4,5,5-tetramethyl-1,3,2-dioxaborolan-2-yl)phenyl)morpholine (Borylated MorDalPhos, **4**)*

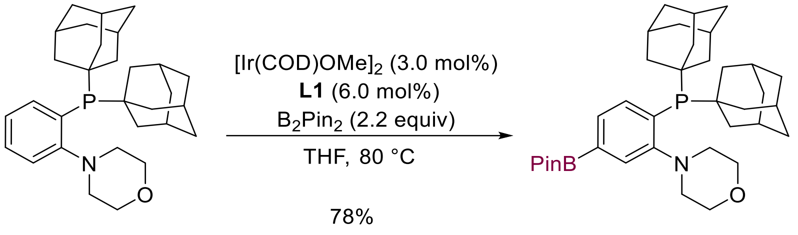

MorDalPhos **4 (MorDalPhos-BPin)**

In an argon glovebox, di(1-adamantyl)-2-morpholinophenylphosphine (MorDalphos, 23.2 mg, 50.0 µmol, 1.00 equiv), $[Ir(COD)OMe]_2$ (1.0 mg, 1.5 µmol, 3.0 mol%), 4,4'-di-tert.-butyl-2,2'-dipyridyl (**L1**, 0.8 mg, 3 µmol, 6 mol%), B_2Pin_2 (28.0 mg, 110 µmol, 2.20 equiv) and THF (1 ml) were added to a 5 ml borosilicate vial. The resulting suspension was heated at 80 °C outside the glovebox. After 24 h, the reaction mixture was left to cool to room temperature and concentrated. Methanol (2 ml) was added to the vial, which was closed with a septum cap and flushed with argon. The vial was sonicated for 5 min subjected to centrifugation (5 min, 4500 rpm), after which the supernatant was removed using a syringe. The washing process was repeated 3 times, using methanol (2 mL) each time. The resulting solid was dried under vacuum to afford the title compound as a white solid (23 mg, 78% yield).

NMR Spectroscopy:

^{1}H NMR (600 MHz, C_6D_6) δ 7.97 (dd, *J* = 7.5, 1.2 Hz, 1H), 7.94 (dd, *J* = 4.6, 1.2 Hz, 1H), 7.86 (dd, *J* = 7.5, 1.4 Hz, 1H), 3.82 (t, *J* = 4.4 Hz, 4H), 3.01 (t, *J* = 4.5 Hz, 4H), 2.16 – 2.02 (m, 12H), 1.86 (t, *J* = 3.1 Hz, 6H), 1.64 (d, *J* = 3.3 Hz, 12H), 1.16 (s, 12H).

^{13}C NMR (151 MHz, C_6D_6) δ 159.7, 159.6, 137.3, 129.2, 127.1, 83.9, 67.3, 54.1, 54.0, 42.4, 42.3, 37.4, 37.3, 37.2, 29.4 (29.41), 29.4 (29.36), 25.0.

^{31}P NMR (243 MHz, C_6D_6) δ 20.5.

HRMS-ESI (m/z): Calculated for $C_{36}H_{53}NO_3PB$ $[M+H]^+$, 590.3929; Found: 590.3931.

*2'-(dicyclohexylphosphaneyl)-N^2,N^2,N^6,N^6-tetramethyl-4,4'-bis(4,4,5,5-tetramethyl-1,3,2-dioxaborolan-2-yl)-[1,1'-biphenyl]-2,6-diamine (Borylated CPhos, **5**)*

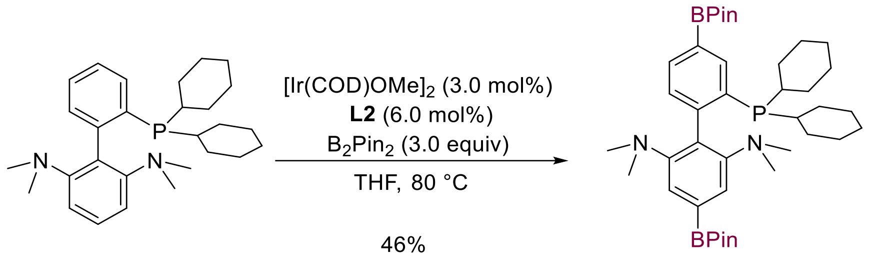

CPhos **5 (CPhos-Bpin)**

In an argon glovebox, 2-dicyclohexylphosphino-2′,6′-bis(N,N-dimethylamino)biphenyl (CPhos, 44.7 mg, 100 µmol, 1.00 equiv), $[Ir(COD)OMe]_2$ (2.0 mg, 3.0 µmol, 3.0 mol%), 3,4,7,8-tetramethyl-1,10-phenanthroline (**L2**, 1.4 mg, 6.0 µmol, 6.0 mol%), B_2Pin_2 (76.2 mg, 300 µmol, 3.00 equiv) and THF (2 ml) were added to an 8 ml borosilicate vial. The resulting suspension was heated at 80 °C outside the glovebox. After 24 h, the reaction mixture was left to cool to room temperature and concentrated. Methanol (3 ml) was added to the vial, which was closed with a septum cap and flushed with argon. The vial was sonicated for 5 min subjected to centrifugation (5 min, 4500 rpm), after which the supernatant was removed using a syringe. The washing process was repeated 3 times, using methanol (3 mL) each time. The resulting solid was dried under vacuum to afford the title compound as a white solid (32 mg, 46% yield).

NMR Spectroscopy:

^{1}H NMR (600 MHz, C_6D_6) δ 8.60 (t, *J* = 1.6 Hz, 1H), 8.20 (dt, *J* = 7.6, 1.1 Hz, 1H), 7.86 (s, 2H), 7.52 (dd, *J* = 7.6, 3.6 Hz, 1H), 2.42 (s, 12H), 2.12 – 2.00 (m, 4H), 1.82 (d, *J* = 9.6 Hz, 2H), 1.67 (d, *J* = 12.1 Hz, 2H), 1.57 (d, *J* = 8.5 Hz, 4H), 1.35 (qt, *J* = 12.7, 3.8 Hz, 2H), 1.23 – 1.05 (m, 32H).

^{13}C NMR (151 MHz, C_6D_6) δ 153.6 (153.57), 153.6 (153.56), 150.8, 150.6, 139.7, 139.6, 137.1, 137.0 (137.04), 137.0 (137.02), 136.9, 134.4, 132.9 (132.94), 132.9 (132.89), 121.8, 83.7 (83.71), 83.7 (83.70), 45.2, 35.6, 35.5, 31.9, 31.7, 30.4, 30.3, 28.1, 28.0, 27.7, 27.6, 27.0, 25.1, 25.0.

^{31}P NMR (243 MHz, C_6D_6) δ –8.8.

HRMS-ESI (m/z): Calculated for $C_{40}H_{63}B_2N_2O_4P$ $[M+H]^+$, 689.4784; Found: 689.4791.

*1-(2,6-bis(cyclohexyloxy)-4-(4,4,5,5-tetramethyl-1,3,2-dioxaborolan-2-yl)phenyl)-2-(di-tert-butylphosphaneyl)-4-(4,4,5,5-tetramethyl-1,3,2-dioxaborolan-2-yl)-1H-imidazole (**6**)*

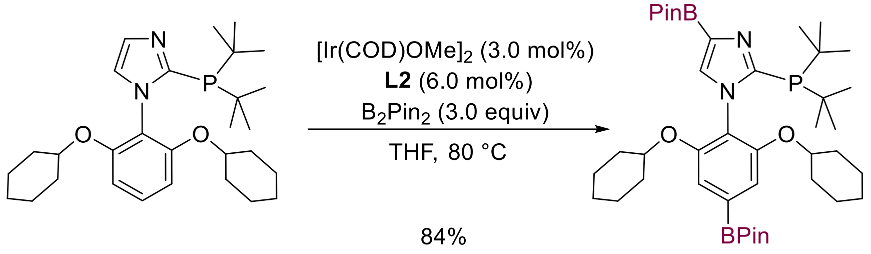

6

In an argon glovebox, 1-[2,6-bis(cyclohexyloxy)phenyl]-2-(di-tertbutylphosphaneyl)-1H-imidazole (48.4 mg, 100 µmol, 1.00 equiv), $[Ir(COD)OMe]_2$ (2.0 mg, 3.0 µmol, 3.0 mol%), 3,4,7,8-tetramethyl-1,10-phenanthroline (**L2**, 1.4 mg, 6.0 µmol, 6.0 mol%), B_2Pin_2 (76.2 mg, 300 µmol, 3.00 equiv) and THF (2 ml) were added to a 5 ml borosilicate vial. The resulting suspension was heated at 80 °C outside the glovebox. After 24 h, the reaction mixture was left to cool to room temperature and concentrated. Acetonitrile (1 ml) was added to the vial, which was closed with a septum cap and flushed with argon. The vial was sonicated for 5 min subjected to centrifugation (5 min, 4500 rpm), after which the supernatant was removed using a syringe. The washing process was repeated 3 times, using acetonitrile (1 mL) each time. The resulting solid was dried under vacuum to afford the title compound as a white solid (51 mg, 84% yield).

NMR Spectroscopy:

^{1}H NMR (600 MHz, C_6D_6) δ 7.82 (d, *J* = 2.8 Hz, 1H), 7.47 (s, 2H), 4.12 – 4.04(m, 2H), 1.79 (d, *J* = 12.6 Hz, 2H), 1.60 (t, *J* = 7.6 Hz, 2H), 1.45 (d, *J* = 11.9 Hz, 18H), 1.40 – 1.23 (m, 10H), 1.13 (s, 12H), 1.08 (s, 12H), 0.97 – 0.87 (m, 6H).

^{13}C NMR (151 MHz, C_6D_6) δ 155.0, 151.8, 151.7, 135.2, 133.9, 130.5, 122.9 (122.94), 122.9 (122.93), 112.9, 84.2, 83.0, 76.7, 33.5, 33.4, 32.3, 31.5, 31.2, 31.1, 25.6, 25.1, 25.0, 23.7 (23.74), 23.7 (23.71).

^{31}P NMR (243 MHz, C_6D_6) δ 8.4.

HRMS-ESI (m/z): Calculated for $C_{41}H_{67}B_2N_2O_6P$ $[M+H]^+$, 737.4996; Found 737.5000

*Tris(2,6-dimethoxy-4-(4,4,5,5-tetramethyl-1,3,2-dioxaborolan-2-yl)phenyl)phosphane (**7**)*

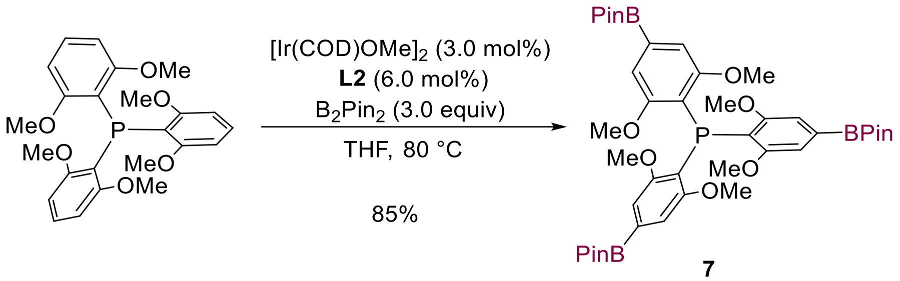

In an argon glovebox, tris(2,6-dimethoxyphenyl)phosphine (44.3 mg, 100 µmol, 1.00 equiv), $[Ir(COD)OMe]_2$ (2.0 mg, 3.0 µmol, 3.0 mol%), 3,4,7,8-tetramethyl-1,10-phenanthroline (**L2**, 1.4 mg, 6.0 µmol, 6.0 mol%), B_2Pin_2 (76.2 mg, 300 µmol, 3.00 equiv) and THF (2 ml) were added to an 8 ml borosilicate vial. The resulting suspension was heated at 80 °C outside the glovebox. After 24 h, the reaction mixture was left to cool to room temperature and concentrated. Methanol (0.5 ml) was added to the vial, which was closed with a septum cap and flushed with argon. The vial was sonicated for 5 min subjected to centrifugation (5 min, 4500 rpm), after which the supernatant was removed using a syringe. The washing process was repeated 3 times, using methanol (0.5 mL) each time. The resulting solid was dried under vacuum to afford the title compound as a white solid (69 mg, 85% yield).

NMR Spectroscopy:

^{1}**H NMR** (600 MHz, C_6D_6) δ 7.27 (d, *J* = 2.9 Hz, 6H), 3.22 (s, 18H), 1.15 (s, 36H).

^{13}**C NMR** (151 MHz, C_6D_6) δ 162.6, 162.5, 122.4, 122.3, 110.7, 83.6, 55.7, 25.1.

^{31}**P NMR** (243 MHz, C_6D_6) δ –61.9.

HRMS-ESI (m/z): Calculated for $C_{42}H_{60}B_3O_{12}P$ $[M+H]^+$, 821.4174; Found: 821.4172.

Borylation of [1,1'-binaphthalen]-2-yldi-tert-butylphosphane (8)

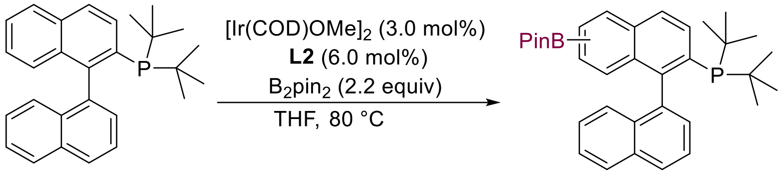

8

In an argon glovebox, [1,1'-binaphthalen]-2-yldi-tert-butylphosphane (39.9 mg, 100 µmol, 1.00 equiv), $[Ir(COD)OMe]_2$ (2.0 mg, 3.0 µmol, 3.0 mol%), 3,4,7,8-tetramethyl-1,10-phenanthroline (**L2**, 1.4 mg, 6.0 µmol, 6.0 mol%), B_2Pin_2 (55.9 mg, 220 µmol, 2.20 equiv) and THF (2 ml) were added to an 8 ml borosilicate vial. The resulting suspension was heated at 80 °C outside the glovebox. After 24 h, the reaction mixture was left to cool to room temperature and concentrated.

Borylation of 4-(anthracen-9-yl)-3-(tert-butyl)-2,3-dihydrobenzo[d][1,3]oxaphosphole (9)

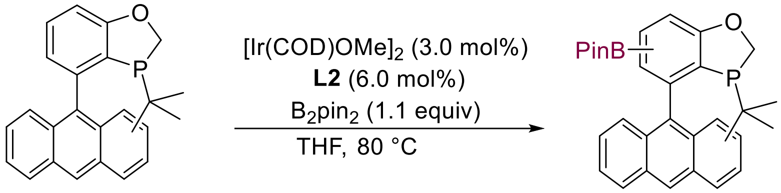

9

In an argon glovebox, 4-(anthracen-9-yl)-3-(tert-butyl)-2,3-dihydrobenzo[d][1,3]oxaphosphole (9.3 mg, 25 µmol, 1.00 equiv), $[Ir(COD)OMe]_2$ (0.50 mg, 0.75 µmol, 3.0 mol%), 3,4,7,8-tetramethyl-1,10-phenanthroline (**L2**, 0.35 mg, 1.5 µmol, 6.0 mol%), B_2Pin_2 (6.9 mg, 28 µmol, 1.1 equiv) and THF (0.5 ml) were added to an 5 ml borosilicate vial. The resulting suspension was heated at 80 °C outside the glovebox. After 24 h, the reaction mixture was left to cool to room temperature and concentrated.

Borylation of tri(naphthalen-1-yl)phosphane (10)

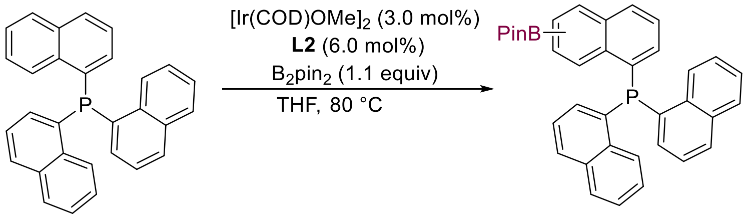

10

In an argon glovebox, tri(naphthalen-1-yl)phosphane (21 mg, 50 µmol, 1.0 equiv), $[Ir(COD)OMe]_2$ (1.0 mg, 1.5 µmol, 3.0 mol%), 3,4,7,8-tetramethyl-1,10-phenanthroline (**L2**, 0.70 mg, 3.0 µmol, 6.0 mol%), B_2Pin_2 (14 mg, 55 µmol, 1.1 equiv) and THF (1 ml) were added to an 5 ml borosilicate vial. The resulting suspension was heated at 80 °C outside the glovebox. After 24 h, the reaction mixture was left to cool to room temperature and concentrated.

*Diphenyl(2'-(4,4,5,5-tetramethyl-1,3,2-dioxaborolan-2-yl)-[1,1'-biphenyl]-2-yl)phosphane (Borylated PhJohnPhos, **12**)*

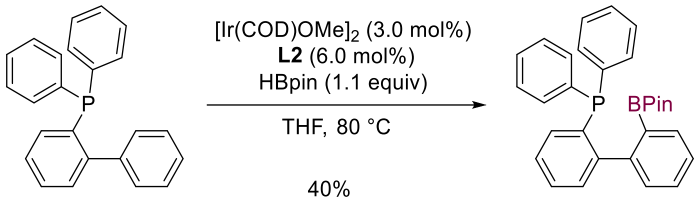

PhJohnPhos **12 (PhJohnPhos-BPin)**

In an argon glovebox, 2-(Diphenylphosphino)biphenyl (67.7 mg, 200 µmol, 1.00 equiv), $[Ir(COD)OMe]_2$ (4.0 mg, 6.0 µmol, 3.0 mol%), 3,4,7,8-tetramethyl-1,10-phenanthroline (**L2**, 2.8 mg, 12 µmol, 6.0 mol%), HBpin (28.2 mg, 220 µmol, 1.10 equiv) and THF (4 ml) were added to a 12 ml borosilicate vial. The resulting suspension was heated at 80 °C outside the glovebox. After 24 h, the reaction mixture was left to cool to room temperature and concentrated. The residue was purified by flash column chromatography on silica gel (pentane/EtOAc = 40:1, v/v) to afford the title compound as a colorless liquid (R_f = 0.4, 37 mg, 40% yield).

NMR Spectroscopy:

^{1}H NMR (600 MHz, C_6D_6) δ 8.13 (ddd, *J* = 7.4, 1.5, 0.6 Hz, 1H), 7.49 (tt, *J* = 6.8, 1.4 Hz, 2H), 7.39 – 7.34 (m, 2H), 7.31 (dddd, *J* = 7.6, 3.8, 1.5, 0.5 Hz, 1H), 7.22 (dddd, *J* = 7.5, 4.5, 1.5, 0.6 Hz, 1H), 7.14 – 7.09 (m, 4H), 7.08 – 7.05 (m, 3H), 7.05 – 7.00 (m, 4H), 1.06 (s, 6H), 0.99 (s, 6H).

^{13}C NMR (151 MHz, C_6D_6) δ 150.5, 150.3, 148.4 (148.43), 148.4 (148.39), 139.6, 139.5, 138.3, 138.2, 137.3, 137.2, 135.4, 134.8, 134.6, 134.2, 134.0, 133.6, 130.8 (130.81), 130.8 (130.79), 130.7 (130.71), 130.7 (130.67), 129.8, 128.7, 128.6, 128.5 (128.53), 128.5 (128.48), 128.4, 127.3, 126.7, 83.2, 25.1, 24.5.

^{31}P NMR (243 MHz, C_6D_6) δ –13.5.

HRMS-ESI (m/z): Calculated for $C_{30}H_{30}BO_2P$ $[M+H]^+$, 465.2149; Found: 465.2151.

*(5-methyl-2-(4,4,5,5-tetramethyl-1,3,2-dioxaborolan-2-yl)phenyl)di-m-tolylphosphane (**13**)*

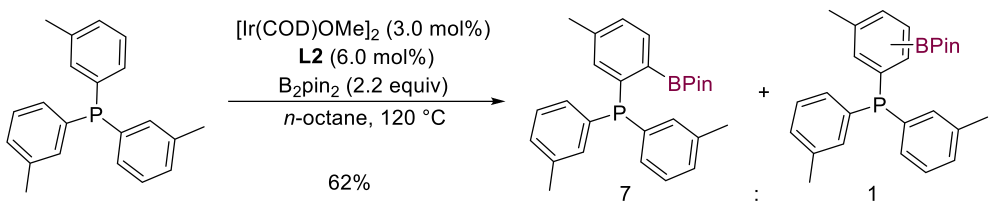

13

In an argon glovebox, tri-m-tolylphosphine (60.9 mg, 200 µmol, 1.00 equiv), $[Ir(COD)OMe]_2$ (4.0 mg, 6.0 µmol, 3.0 mol%), 3,4,7,8-tetramethyl-1,10-phenanthroline (**L2**, 2.8 mg, 12 µmol, 6.0 mol%), B_2pin_2 (112 mg, 440 µmol, 2.20 equiv) and *n*-octane (4 ml) were added to a 12 ml borosilicate vial. The resulting suspension was heated at 120 °C outside the glovebox. After 24 h, the reaction mixture was left to cool to room temperature and concentrated. The residue was purified by column chromatography on silica gel (pentane/EtOAc = 40:1, v/v) to afford a 7 : 1 mixture of the title compound and a regioisomer as a colorless liquid (R_f =0.6, 53 mg, 62% yield).

NMR Spectroscopy:

^{1}H NMR (600 MHz, C_6D_6) δ 8.16 (dd, *J* = 7.6, 3.1 Hz, 1H), 7.47 (d, *J* = 8.1 Hz, 2H), 7.38 (t, *J* = 7.3 Hz, 2H), 7.10 (d, *J* = 4.5 Hz, 1H), 7.07 (t, *J* = 7.5 Hz, 2H), 6.99 – 6.96 (m, 1H), 6.91 (dd, *J* = 7.7, 1.8 Hz, 2H), 2.00 (s, 6H), 1.91 (s, 3H), 1.03 (s, 12H).

^{13}C NMR (151 MHz, C_6D_6) δ 145.5, 145.4, 140.8, 140.0, 139.9, 138.0 (138.03), 138.0 (137.98), 136.6, 136.5, 135.5, 135.4, 134.7, 134.6, 133.9, 133.8, 131.9, 131.8, 129.4, 128.7, 128.6, 128.4, 83.7, 24.8, 21.6, 21.3.

^{31}P NMR (122 MHz, C_6D_6) δ –3.5.

HRMS-ESI (m/z): Calculated for $C_{27}H_{32}O_2PB$ $[M+H]^+$, 431.2306; Found: 431.2306.

*(5-chloro-2-(4,4,5,5-tetramethyl-1,3,2-dioxaborolan-2-yl)phenyl)bis(3-chlorophenyl)phosphane (**14**) and (3-chloro-2-(4,4,5,5-tetramethyl-1,3,2-dioxaborolan-2-yl)phenyl)bis(3-chlorophenyl)phosphane (**15**)*

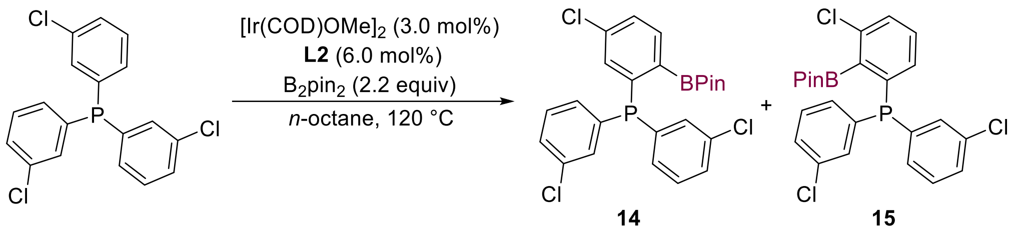

In an argon glovebox, tri(m-chlorophenyl)phosphine (73.1 mg, 200 µmol, 1.00 equiv), $[Ir(COD)OMe]_2$ (4.0 mg, 6.0 µmol, 3.0 mol%), 3,4,7,8-tetramethyl-1,10-phenanthroline (**L2**, 2.8 mg, 12 µmol, 6.0 mol%), B_2pin_2 (112 mg, 440 µmol, 2.20 equiv) and *n*-octane (4 ml) were added to a 12 ml borosilicate vial. The resulting suspension was heated at 120 °C outside the glovebox. After 24 h, the reaction mixture was left to cool to room temperature and concentrated. The residue was purified by flash column chromatography on silica gel (pentane/DCM = 2:1, v/v) to afford **14** as a colorless liquid (R_f = 0.60, 34 mg, 35% yield) and **15** as a colorless liquid (R_f = 0.65, 8 mg, 8% yield).

Note: Compounds **14** and **15** can undergo oxidation to the corresponding phosphine oxides in the presence of air. The time needed to carry out purification via flash column chromatography should not exceed one hour to limit formation of phosphine oxide side products.

NMR Spectroscopy for 14:

^{1}**H NMR** (600 MHz, C_6D_6) δ 7.88 (ddd, *J* = 8.0, 3.0, 0.4 Hz, 1H), 7.48 (dddd, *J* = 7.8, 2.1, 1.5, 0.4 Hz, 2H), 7.08 (ddd, *J* = 3.6, 2.1, 0.4 Hz, 1H), 7.05 (dd, *J* = 7.9, 2.1 Hz, 1H), 7.01 – 6.96 (m, 4H), 6.71 – 6.66 (m, 2H), 0.94 (s, 12H).

^{13}**C NMR** (151 MHz, C_6D_6) δ 146.6, 146.4, 140.7, 140.6, 138.2 (138.23), 138.2 (138.21), 138.2 (138.17), 135.3, 135.2, 134.4, 134.2, 132.5 (132.50), 132.5 (132.49), 132.3, 132.2, 130.3, 130.2, 129.3, 84.3, 24.6.

^{31}**P NMR** (243 MHz, C_6D_6) δ –3.1.

HRMS-ESI (m/z): Calculated for $C_{24}H_{23}O_2BPCl_3$ $[M+H]^+$, 491.0667; Found: 491.0667.

NMR Spectroscopy for 15:

^{1}**H NMR** (600 MHz, C_6D_6) δ 7.40 (dddd, *J* = 7.4, 2.0, 1.4, 0.4 Hz, 2H), 7.01 (dt, *J* = 8.0, 0.7 Hz, 1H), 6.98 (ddd, *J* = 8.0, 2.1, 1.0 Hz, 2H), 6.96 – 6.94 (m, 2H), 6.81 (ddd, *J* = 7.7, 3.8, 0.9 Hz, 1H), 6.69 (td, *J* = 7.8, 1.6 Hz, 2H), 6.61 (td, *J* = 7.9, 0.7 Hz, 1H), 1.23 (s, 12H).

^{13}**C NMR** (151 MHz, C_6D_6) δ 140.2, 140.1, 137.7, 137.6, 136.6, 136.5, 133.3, 133.2, 131.5, 131.3, 129.8, 129.7, 129.4, 128.9, 128.2 (128.23), 128.2 (128.18), 127.8, 127.2, 83.0, 23.2 (23.17), 23.2 (23.16).

^{31}**P NMR** (243 MHz, C_6D_6) δ –6.4.

HRMS-ESI (m/z): Calculated for $C_{24}H_{23}O_2BPCl_3$ $[M+H]^+$, 491.0667; Found: 491.0667.

Borylation of [1-(Diphenylphosphino)ethyl]ferrocene (16)

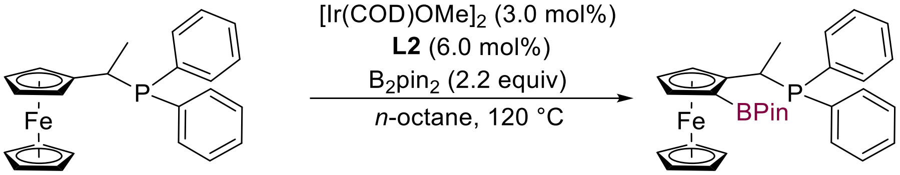

48% **16**

In an argon glovebox, [1-(Diphenylphosphino)ethyl]ferrocene (39.8 mg, 100 µmol, 1.00 equiv), $[Ir(COD)OMe]_2$ (2.0 mg, 3.0 µmol, 3.0 mol%), 3,4,7,8-tetramethyl-1,10-phenanthroline (**L2**, 1.4 mg, 6 µmol, 6.0 mol%), B_2pin_2 (55.9 mg, 220 µmol, 2.20 equiv) and *n*-octane (2 ml) were added to a 8 ml borosilicate vial. The resulting suspension was heated at 120 °C outside the glovebox. After 72 h, the reaction mixture was left to cool to room temperature and concentrated. MeCN (1 ml) was added to the vial, which was closed with a septum cap and flushed with argon. The vial was sonicated for 5 min and subjected to centrifugation (5 min, 4500 rpm), after which the supernatant was removed using a syringe. The washing process was repeated 3 times, using MeCN (1 mL) each time. The resulting solid was dried under vacuum to afford the title compound as a yellow solid (25 mg, 48% yield).

NMR Spectroscopy:

^{1}H NMR (600 MHz, C_6D_6) δ 7.76 (ddd, *J* = 8.1, 6.9, 1.5 Hz, 2H), 7.27 (ddd, *J* = 7.9, 6.2, 1.4 Hz, 2H), 7.22 – 7.19 (m, 2H), 7.18 – 7.17 (m, 1H), 7.05 – 7.01 (m, 2H), 7.01 – 6.97 (m, 1H), 4.55 (dd, *J* = 2.4, 1.3 Hz, 1H), 4.29 – 4.20 (m, 2H), 4.19 – 4.14 (m, 2H), 4.10 (s, 4H), 1.54 (dd, *J* = 12.8, 7.1 Hz, 3H), 1.10 (d, *J* = 3.1 Hz, 12H).

^{13}C NMR (151 MHz, C_6D_6) δ 138.7, 138.5, 136.8, 136.7, 135.2, 135.1, 132.9, 132.8, 128.9, 128.1, 127.2, 99.3, 99.2, 82.4, 73.1, 70.8, 70.2 (70.16), 70.2 (70.19), 68.8, 30.8, 30.7, 25.0, 24.7, 18.3, 18.2.

^{31}P NMR (122 MHz, C_6D_6) δ 13.36.

HRMS-ESI (m/z): Calculated for $C_{30}H_{34}BFeO_2P$ $[M+H]^+$, 525.1812; Found: 525.1811.

*2-(dicyclohexylphosphaneyl)-2',4',6'-triisopropyl-[1,1'-biphenyl]-4-ol (**19**)*

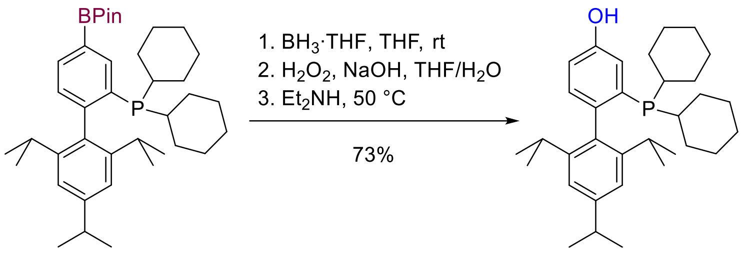

1 **19**

The transformation was carried out according to a modified literature procedure.[30]

Step 1:

A solution of borylated XPhos (**1**, 200.0 mg, 331.9 µmol, 1.00 equiv) in THF (8 mL) in a 25 mL round-bottom flask containing a stir bar was purged with argon for 10 minutes before a 1M solution of BH_3·THF in THF (498 µL, 498 µmol, 1.50 equiv) was added. The resulting solution was left to stir until TLC analysis (7:1 hexanes : ethyl acetate) indicated completion. No starting material could be detected after 15 min, whereupon water (0.5 mL) was added to quench any remaining borane reagent. The reaction mixture was concentrated under reduced pressure, after which THF (2 × 7 mL) was added and the solution concentrated again to remove water residue. MeCN (15 mL) was added to the resulting solid and the suspension was subjected to sonication for 5 minutes before the solid was removed by filtration and dried under vacuum at room temperature.

Step 2:

The borane adduct of XPhos (**29**) obtained in Step 1 was placed in a 100 mL round bottom flask containing a stir bar along with THF (27 mL) and water (3 mL), before H_2O_2 (35% aqueous solution, 112 µL, 996 µmol, 3.00 equiv) and NaOH (1M aqueous solution, 664 µL, 664 µmol, 2.00 equiv) were added. The reaction mixture was stirred at room temperature until TLC analysis (4 : 1 hexanes : ethyl acetate) indicated completion of the reaction. After a reaction time of 15 minutes, no starting material could be detected, upon which the reaction was quenched by the addition of 1M HCl (1 mL) and transferred into a separatory funnel. The reaction mixture was extracted with ethyl acetate (3 × 30 mL) and the combined extracts were washed with water (30 mL) and dried over anhydrous Na_2SO_4. The resulting solution was concentrated under reduced pressure before being subjected to trituration with DCM (6 mL) to remove small amounts of phosphine oxide as well as boron-containing impurities.

Step 3:

The material obtained in step 2 was placed in a 25 mL round bottom flask and diethylamine (6 mL) was added before the reaction mixture was purged with argon for 10 minutes and subsequently heated to 50 °C for 1 hour. Concentration by rotary evaporator followed by drying under vacuum at room temperature yielded the title compound as a white solid (119 mg, 73% yield).

NMR Spectroscopy:

^{1}**H NMR** (300 MHz, C_6D_6) δ 7.24 (s, 2H), 7.09 (dd, *J* = 8.2, 4.0 Hz, 1H), 7.03 – 7.00 (m, 1H), 6.45 (dd, *J* = 8.3, 2.6 Hz, 1H), 3.84 (s, 1H), 2.96 – 2.75 (m, 3H), 1.95 – 1.58 (m, 13H), 1.47 (d, *J* = 6.9 Hz, 6H), 1.31 – 1.27 (m, 7H), 1.25 – 1.13 (m, 14H).

^{13}**C NMR** (151 MHz, C_6D_6) δ 154.6, 148.3, 147.3, 140.0, 139.8, 139.0, 138.9, 137.0, 136.9, 132.7 (132.73), 132.7 (132.69), 120.6, 118.9 (118.87), 118.9 (118.85), 115.3, 34.9, 34.8 (34.79), 34.8 (34.75), 31.5, 31.4, 31.1, 29.7, 29.6, 27.9 (27.94), 27.9 (27.86), 27.7, 27.6, 26.8, 26.4, 24.4, 23.4 (23.38), 23.4 (23.37).

^{31}**P NMR** (122 MHz, C_6D_6) δ –11.3.

HRMS-ESI (m/z): Calculated for $C_{33}H_{49}OP$ $[M+H]^+$, 493.3594; Found: 493.3592.

*Dicyclohexyl(2',4',6'-triisopropyl-4-methoxy-[1,1'-biphenyl]-2-yl)phosphane (**20**)*

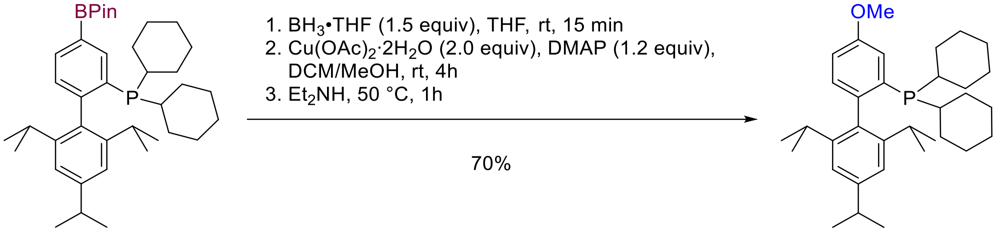

1, XPhos-BPin **20**

The transformation was performed according to modified literature procedures.[28, 30-31]

Step 1:

A solution of borylated XPhos (**1**, 120.0 mg, 199 µmol, 1.00 equiv) in THF (8 mL) in a 25 mL round-bottom flask containing a stir bar was purged with argon for 10 minutes before a 1M solution of BH_3·THF in THF (299 µL, 299 µmol, 1.50 equiv) was added. The resulting solution was left to stir until TLC analysis (7:1 hexanes : ethyl acetate) indicated completion. No starting material could be detected after 15 min, whereupon water (0.5 mL) was added to quench any remaining borane reagent. The reaction mixture was concentrated under reduced pressure, after which THF (2 × 5 mL) was added and the solution concentrated again to remove water residue. MeCN (10 mL) was added to the resulting solid and the suspension was subjected to sonication for 5 minutes before the solid was removed by filtration and dried under vacuum at room temperature.

Step 2:

The borane adduct of XPhos (**29**) obtained in Step 1 was placed in a 100 mL round bottom flask containing a stir bar along with $Cu(OAc)_2$·$2H_2O$ (72.3 mg, 398 µmol, 2.00 equiv), 4-(dimethylamino)-pyridine (29.2 mg, 239 µmol, 1.20 equiv), powdered 4Å molecular sieves (60 mg), dichloromethane (4 mL) and methanol (4 mL). The resulting suspension was stirred at room temperature until TLC analysis (7:1 hexanes : ethyl acetate) indicated completion of the reaction. After a reaction time of 4 hours, no starting material could be detected, upon which the reaction mixture was filtered over a pad of silica using dichloromethane as eluent, and the filtrate was concentrated by rotary evaporation. The resulting solid was recrystallized from acetonitrile (8 mL) to yield colorless crystals.

Step 3:

The crystals obtained in step 2 were placed in a 25 mL round bottom flask and diethylamine (4 mL) was added before the reaction mixture was purged with argon for 10 minutes and subsequently heated to 50 °C for 1 hour. Concentration by rotary evaporator followed by drying under vacuum at room temperature yielded the title compound as a white solid (71.0 mg, 70% yield).

NMR Spectroscopy:

^{1}H NMR (300 MHz, C_6D_6) δ 7.38 (t, *J* = 2.2 Hz, 1H), 7.24 (s, 2H), 7.18 (d, *J* = 4.1 Hz, 1H), 6.64 (dd, *J* = 8.4, 2.7 Hz, 1H), 3.36 (s, 3H), 2.94–2.77 (m, 3H), 1.95–1.87 (m, 2H), 1.82–1.73 (m, 4H), 1.67–1.54 (m, 6H), 1.45 (d, *J* = 6.9 Hz, 6H), 1.29–1.11 (m, 22H).

^{13}C NMR (75 MHz, C_6D_6) δ 158.7, 148.6, 147.6, 140.5, 140.0, 139.2, 138.9, 137.3 (137.34), 137.3 (137.26), 133.0, 132.9, 128.9, 120.9, 119.4 (119.44), 119.4 (119.40), 112.9, 55.0, 35.2, 35.1, 35.0, 31.9, 31.7, 31.4 (31.42), 31.4 (31.39), 30.0, 29.8, 28.2, 28.1, 27.9, 27.8, 27.1, 26.7, 24.7, 23.7, 23.6.

^{31}P NMR (122 MHz, C_6D_6) δ –11.2.

HRMS-ESI (m/z): Calculated for $C_{34}H_{51}OP$ $[M+H]^+$, 507.3750; Found: 507.3758.

*3'-(dicyclohexylphosphaneyl)-2'',4'',6''-triisopropyl-N,N-dimethyl-[1,1':4',1''-terphenyl]-3-amine (**22**)*

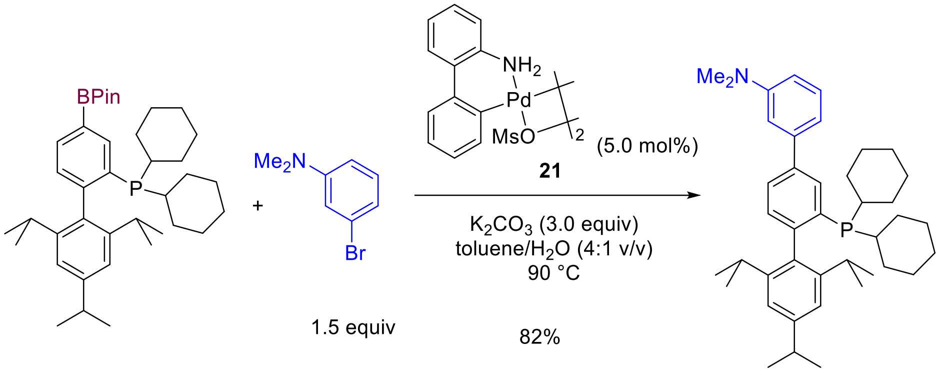

1, XPhos-BPin **22**

XPhos-Bpin (**1**, 200 mg, 0.332 mmol, 1.00 equiv), 3-Bromo-N,N-dimethylaniline (99.6 mg, 0.498 mmol, 1.50 equiv), K_2CO_3 (138 mg, 1.00 mmol, 3.00 equiv), toluene (2.4 ml) and water (0.6 ml) were added to a Schlenk tube containing a stir bar. The resulting suspension was purged with argon for 15 min before the palladium catalyst (**21**, 12.3 mg, 16.6 µmol, 5.00 mol%) was added and the reaction mixture was again purged with argon for 5 min. The resulting suspension was stirred at 90 °C for 24 h. The reaction mixture was left to cool to room temperature and filtered through a pad of celite using THF. The filtrate was concentrated by rotary evaporator, and under an atmosphere of argon, a stir bar and acetonitrile which had been purged with argon (20 ml) was added to the crude reaction product. The suspension was stirred at room temperature for overnight before the solid product was removed by filtration. Drying of the light yellow-white solid afforded the title compound (162 mg, 82% yield).

Note: The title compound shows reasonable stability in the solid phase, but is readily oxidized to the phosphine oxide in solution in the presence of air.

NMR Spectroscopy:

^{1}H NMR (300 MHz, C_6D_6) δ 8.14 (t, *J* = 2.0 Hz, 1H), 7.59 (dd, *J* = 7.8, 1.8 Hz, 1H), 7.37 (dd, *J* = 7.9, 3.9 Hz, 1H), 7.32 – 7.26 (m, 3H), 7.21 – 7.17 (m, 1H), 7.04 (dd, *J* = 2.6, 1.6 Hz, 1H), 6.60 (ddd, *J* = 8.2, 2.6, 1.0 Hz, 1H), 2.94 – 2.82 (m, 3H), 2.57 (s, 6H), 2.00 – 1.86 (m, 6H), 1.72 – 1.52 (m, 7H), 1.47 (d, *J* = 6.9 Hz, 6H), 1.30 – 1.24 (m, 10H), 1.20 – 1.09 (m, 11H).

^{13}C NMR (151 MHz, C_6D_6) δ 151.4, 148.5, 147.1, 147.0, 146.9, 142.7, 141.1, 137.8, 137.7, 137.1, 137.0, 132.3 (132.29), 132.3 (132.25), 131.8, 131.7, 129.9, 127.3, 120.7, 116.3, 112.3, 112.2, 40.2, 35.0, 34.8 (34.84), 34.8 (34.82), 31.7, 31.6, 31.2 (31.24), 31.2 (31.23), 29.8, 29.7, 28.0, 27.9, 27.7, 27.6, 26.9, 26.5, 24.4, 23.4 (23.41), 23.4 (23.40).

^{31}P NMR (122 MHz, C_6D_6) δ –11.5.

HRMS-ESI (m/z): Calculated for $C_{41}H_{58}NP$ $[M+H]^+$, 596.4380; Found: 596.4385.

*3-(3'-(dicyclohexylphosphaneyl)-2'',4'',6''-triisopropyl-[1,1':4',1''-terphenyl]-3-yl)-5-ethyl-1,2,4-oxadiazole (**23**)*

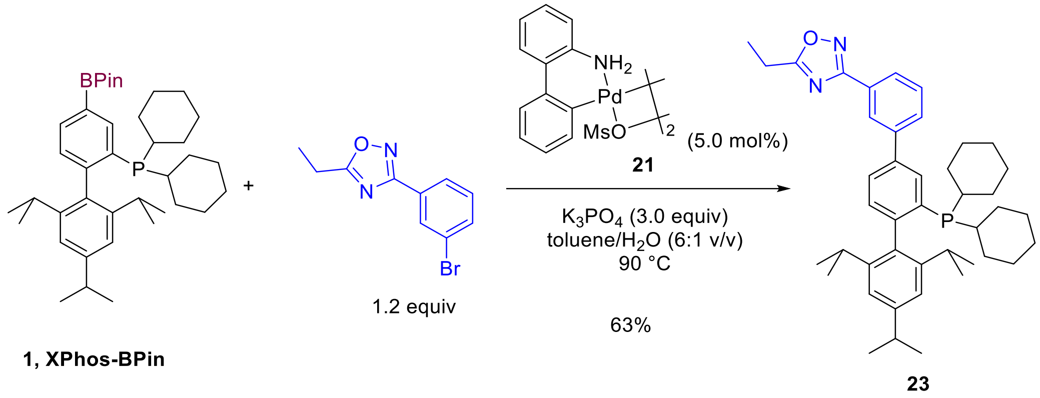

XPhos-Bpin (**1**, 60.3 mg, 100 µmol, 1.00 equiv), 3-(3-Bromphenyl)-5-ethyl-1,2,4-oxadiazol (30.4 mg, 120 µmol, 1.20 equiv), K_3PO_4 (63.7 mg, 0.300 mmol, 3.00 equiv), toluene (1.5 ml) and water (0.25 ml) were added to a Schlenk tube containing a stir bar. The resulting suspension was purged with argon for 15 min before the palladium catalyst (**21**, 3.7 mg, 5.0 µmol, 5.0 mol%) was added and the reaction mixture was again purged with argon for 5 min. The resulting suspension was stirred at 90 °C for 2 days. The reaction mixture was left to cool to room temperature and filtered through a pad of celite using THF. The filtrate was concentrated under reduced pressure. Under an atmosphere of argon, a stir bar and methanol (20 mL), which had been purged with argon, was added to the crude reaction product. The suspension was stirred at room temperature for 8 h before the solid product was removed by filtration. Drying of the light yellow-white solid afforded the title compound (41 mg, 63% yield).

Note: The title compound shows reasonable stability in the solid phase, but is readily oxidized to the phosphine oxide in solution in the presence of air.

NMR Spectroscopy:

^{1}H NMR (600 MHz, C_6D_6) δ 8.73 – 8.69 (m, 1H), 8.32 (ddd, *J* = 7.7, 1.6, 1.1 Hz, 1H), 8.12 (dd, *J* = 10.9, 2.0 Hz, 1H), 7.55 (ddd, *J* = 7.8, 2.0, 1.2 Hz, 1H), 7.36 (ddd, *J* = 7.9, 2.0, 1.2 Hz, 1H), 7.30 – 7.21 (m, 3H), 7.19 (td, *J* = 7.8, 0.5 Hz, 1H), 2.91 (hept, *J* = 6.9 Hz, 1H), 2.84 (hept, *J* = 6.8 Hz, 2H), 2.27 (q, *J* = 7.6 Hz, 2H), 2.09 – 1.94 (m, 4H), 1.84 (d, *J* = 13.2 Hz, 2H), 1.73 – 1.35 (m, 16H), 1.30 (d, *J* = 6.9 Hz, 6H), 1.25 – 0.99 (m, 12H), 0.96 (t, *J* = 7.6 Hz, 3H).

^{13}C NMR (151 MHz, C_6D_6) δ 181.1, 168.6, 148.4, 146.6, 145.9, 145.8, 141.4, 138.4 (138.43), 138.4 (138.36), 136.6, 136.5, 134.7 (134.74), 134.7 (134.67), 133.8, 133.3, 130.6 (130.64), 130.6 (130.57), 129.9 (129.91), 129.9 (129.85), 128.8 (128.78), 128.8 (128.77), 128.7, 127.0, 126.6, 120.6, 38.7, 38.2, 34.8, 31.6, 27.2, 27.1, 27.0, 26.9, 26.8 (26.82), 26.8 (26.80), 26.6 (26.58), 26.6 (26.56), 26.5, 26.4, 24.4, 23.4, 20.1, 10.5.

^{31}P NMR (122 MHz, C_6D_6) δ –11.4.

HRMS-ESI (m/z): Calculated for $C_{43}H_{57}N_2OP$ $[M+H]^+$, 649.4281; Found: 649.4285.

*8-(2-(dicyclohexylphosphaneyl)-2',4',6'-triisopropyl-[1,1'-biphenyl]-4-yl)quinolone (**24**)*

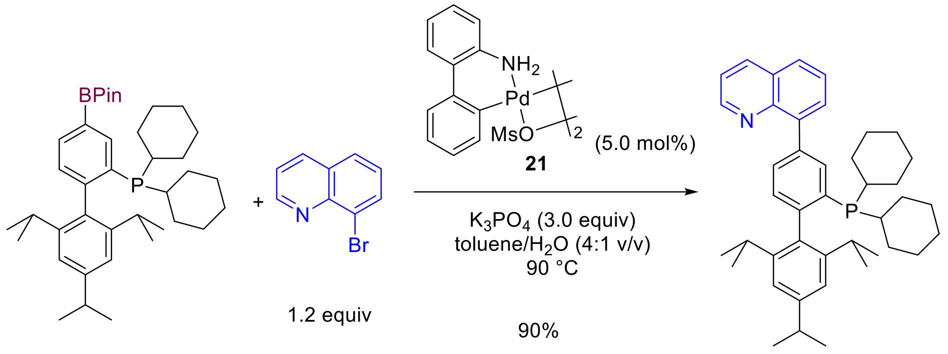

1, XPhos-BPin **24**

XPhos-Bpin (**1**, 200 mg, 0.332 mmol, 1.00 equiv), 8-Bromoquinoline (82.9 mg, 0.398 mmol, 1.20 equiv), K_3PO_4 (211 mg, 1.00 mmol, 3.00 equiv), toluene (2.4 ml) and water (0.6 ml) were added to a Schlenk tube containing a stir bar. The resulting suspension was purged with argon for 15 min before the palladium catalyst (**21**, 12.3 mg, 16.6 µmol, 5.00 mol%) was added and the reaction mixture was again purged with argon for 5 min. The resulting suspension was stirred at 90 °C for 2 days. The reaction mixture was left to cool to room temperature and filtered through a pad of celite using THF. The filtrate was concentrated by rotary evaporator, and under an atmosphere of argon, a stir bar and acetonitrile which had been purged with argon (20 ml) was added to the crude reaction product. The suspension was stirred at room temperature for overnight before the solid product was removed by filtration. Drying of the light yellow-white solid afforded the title compound (180 mg, 90% yield).

Note: The title compound shows reasonable stability in the solid phase, but is readily oxidized to the phosphine oxide in solution in the presence of air.

NMR Spectroscopy:

^{1}H NMR (600 MHz, C_6D_6) δ 8.80 (dd, *J* = 4.1, 1.9 Hz, 1H), 8.45 (t, *J* = 2.0 Hz, 1H), 7.69 – 7.64 (m, 2H), 7.55 (ddd, *J* = 8.2, 1.9, 0.4 Hz, 1H), 7.45 (dd, *J* = 7.8, 3.9 Hz, 1H), 7.36 (dd, *J* = 8.5, 1.5 Hz, 1H), 7.29 (d, *J* = 0.5 Hz, 2H), 7.22 (dd, *J* = 8.1, 7.1 Hz, 1H), 6.78 (dd, *J* = 8.2, 4.1 Hz, 1H), 2.97 (hept, *J* = 6.8 Hz, 2H), 2.94 – 2.88 (m, 1H), 2.14 – 2.02 (m, 4H), 1.95 (tq, *J* = 12.3, 3.2 Hz, 2H), 1.73 – 1.66 (m, 4H), 1.63 – 1.58 (m, 2H), 1.51 (d, *J* = 6.9 Hz, 6H), 1.42 – 1.31 (m, 6H), 1.29 (d, *J* = 6.9 Hz, 6H), 1.28 – 1.23 (m, 3H), 1.19 (d, *J* = 6.7 Hz, 6H), 1.03 – 1.01 (m, 1H).

^{13}C NMR (151 MHz, C_6D_6) δ 150.1, 148.3, 147.0, 146.8, 146.7, 141.3, 137.9, 137.3 (137.32), 137.3 (137.28), 136.3 (136.34), 136.3 (136.32), 136.2, 136.0, 131.5, 131.4, 130.5, 130.1, 129.1, 127.6, 126.5, 121.0, 120.7, 35.0, 34.8 (34.84), 34.8 (34.76), 31.6, 31.5, 31.2 (31.18), 31.2 (31.16), 29.8, 29.7, 28.2, 28.1, 27.7, 27.6, 26.9, 26.5, 24.4, 23.4 (23.40), 23.4 (23.38).

^{31}P NMR (122 MHz, C_6D_6) δ –11.1.

HRMS-ESI (m/z): Calculated for $C_{42}H_{54}NP$ $[M+H]^+$, 604.4067; Found: 604.4071.

*Dicyclohexyl(2'',3'',4'',5'',6''-pentafluoro-2,4,6-triisopropyl-[1,1':4',1''-terphenyl]-2'-yl)phosphane (**25**)*

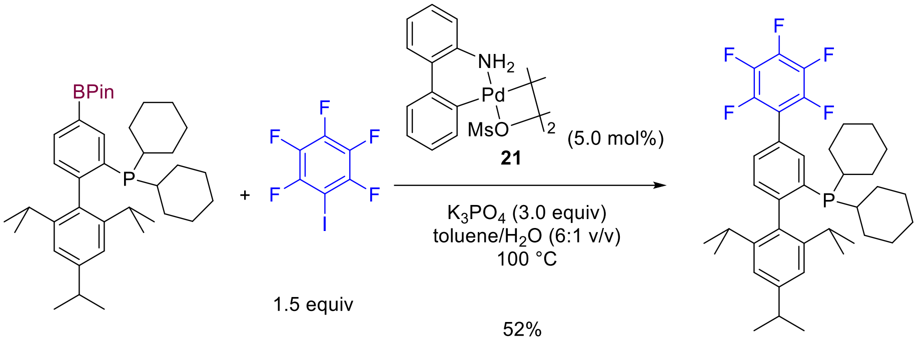

XPhos-Bpin (**1**, 121 mg, 0.200 mmol, 1.00 equiv), iodopentafluorobenzene (88.2 mg, 0.300 mmol, 1.50 equiv), K_3PO_4 (127 mg, 0.600 mmol, 3.00 equiv), toluene (3 ml) and water (0.5 ml) were added to a Schlenk tube containing a stir bar. The resulting suspension was purged with argon for 15 min before the palladium catalyst (**21**, 7.4 mg, 10 µmol, 5.0 mol%) was added and the reaction mixture was again purged with argon for 5 min. The resulting suspension was stirred at 100 °C for 3.5 days. The reaction mixture was left to cool to room temperature and filtered through a pad of celite using THF. The filtrate was concentrated by rotary evaporator, and under an atmosphere of argon, a stir bar and acetonitrile which had been purged with argon (20 ml) was added to the crude reaction product. The suspension was stirred at room temperature for overnight before the solid product was removed by filtration. Drying of the light yellow-white solid afforded the title compound (67 mg, 52% yield).

Note: Among all phosphines investigated in the course of this work, **25** was the most prone to oxidation to the corresponding phosphine oxide and it can only briefly be handled under air.

NMR Spectroscopy:

^{1}H NMR (600 MHz, C_6D_6) δ 7.93 – 7.88 (m, 1H), 7.39 (dd, *J* = 7.9, 3.7 Hz, 1H), 7.28 – 7.23 (m, 3H), 2.89 (hept, *J* = 6.9 Hz, 1H), 2.76 (hept, *J* = 6.8 Hz, 2H), 2.02 – 1.85 (m, 6H), 1.72 – 1.56 (m, 10H), 1.45 (d, *J* = 6.9 Hz, 6H), 1.34 – 1.25 (m, 12H), 1.15 (d, *J* = 6.7 Hz, 6H).

^{13}C NMR (151 MHz, C_6D_6) δ 149.8, 149.6, 149.0, 146.8, 138.7, 138.5, 136.3 (136.29), 136.3 (136.26), 134.4 (134.38), 134.4 (134.36), 132.2 (132.23), 132.2 (132.19), 129.7, 124.9, 120.9, 35.1, 35.0, 34.8, 31.5, 31.4, 31.3 (31.29), 31.3 (31.28), 29.8, 29.7, 28.0, 27.9, 27.7, 27.6, 26.8, 26.4, 24.4, 23.3 (23.29), 23.3 (23.28).

^{31}P NMR (243 MHz, C_6D_6) δ – 12.1.

^{19}F NMR (282 MHz, C_6D_6) δ (–143.8) – (–144.0) (m, 2F), –156.0 (t, *J* = 21.8 Hz, 1F), (–162.1) – (–162.4) (m, 2F).

HRMS-ESI (m/z): Calculated for $C_{39}H_{48}PF_5$ $[M+H]^+$, 643.3487; Found: 643.3484.

*Dicyclohexyl(2',4',6'-triisopropyl-4-(thiophen-2-yl)-[1,1'-biphenyl]-2-yl)phosphane (**26**)*

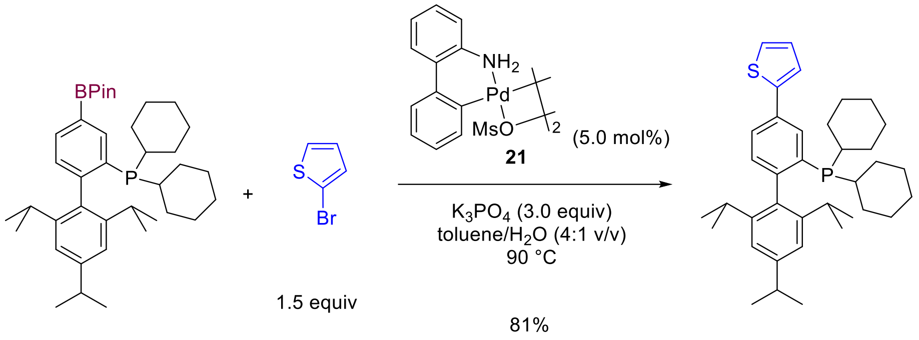

1, XPhos-BPin **26**

XPhos-Bpin (**1**, 200 mg, 0.332 mmol, 1.00 equiv), 2-bromothiophene (81.2 mg, 0.498 mmol, 1.50 equiv), K_3PO_4 (211 mg, 0.996 mmol, 3.00 equiv), toluene (2.4 ml) and water (0.6 ml) were added to a Schlenk tube containing a stir bar. The resulting suspension was purged with argon for 15 min before the palladium catalyst (**21**, 12.3 mg, 16.6 µmol, 5.00 mol%) was added and the reaction mixture was again purged with argon for 5 min. The resulting suspension was stirred at 90 °C for 3 days. The reaction mixture was left to cool to room temperature and filtered through a pad of celite using THF. The filtrate was concentrated by rotary evaporator, and under an atmosphere of argon, a stir bar and acetonitrile which had been purged with argon (2 ml) was added to the crude reaction product. The suspension was stirred at room temperature overnight before the solid product was removed by filtration. Drying of the white solid afforded the title compound (151 mg, 81% yield).

NMR Spectroscopy:

^{1}H NMR (600 MHz, C_6D_6) δ 8.11 (t, *J* = 1.9 Hz, 1H), 7.45 (ddd, *J* = 7.9, 2.0, 0.5 Hz, 1H), 7.27 – 7.23 (m, 3H), 7.22 (dd, *J* = 3.6, 1.2 Hz, 1H), 6.86 (dd, *J* = 5.1, 1.2 Hz, 1H), 6.80 (dd, *J* = 5.1, 3.6 Hz, 1H), 2.90 (hept, *J* = 6.9 Hz, 1H), 2.81 (hept, *J* = 6.8 Hz, 2H), 1.99 – 1.81 (m, 8H), 1.69 – 1.54 (m, 10H), 1.47 (d, *J* = 6.9 Hz, 6H), 1.31 – 1.28 (m, 8H), 1.17 (d, *J* = 6.7 Hz, 6H), 1.10 – 1.07 (m, 2H).

^{13}C NMR (151 MHz, C_6D_6) δ 148.7, 147.6, 147.4, 146.9, 144.8, 138.3, 138.2, 136.8, 136.7, 133.0, 132.5 (132.51), 132.5 (132.47), 130.0 (130.04), 130.0 (130.02), 125.7, 125.0, 123.4, 120.8, 34.9, 34.8 (34.81), 34.8 (34.76), 31.6, 31.5, 31.2 (31.22), 31.2 (31.21), 29.7, 29.6, 28.0, 27.9, 27.6 (27.64), 27.6 (27.58), 26.8, 26.4, 24.4, 23.4, 23.3.

^{31}P NMR (243 MHz, C_6D_6) δ –12.0.

HRMS-ESI (m/z): Calculated for $C_{37}H_{51}PS$ $[M+H]^+$, 559.3522; Found: 559.3526.

*Dicyclohexyl(2,4,6-triisopropyl-3'',4''-dimethoxy-[1,1':4',1''-terphenyl]-2'-yl)phosphane (**27**)*

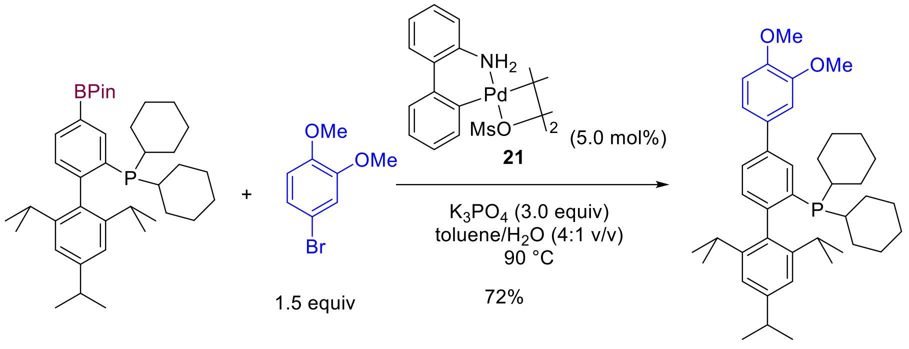

1, XPhos-BPin **27**

XPhos-Bpin (**1**, 200 mg, 0.332 mmol, 1.00 equiv), 4-bromoveratrol (108 mg, 0.498 mmol, 1.50 equiv), K_3PO_4 (211 mg, 0.996 mmol, 3.00 equiv), toluene (2.4 ml) and water (0.6 ml) were added to a Schlenk tube containing a stir bar. The resulting suspension was purged with argon for 15 min before the palladium catalyst (**21**, 12.3 mg, 16.6 µmol, 5.00 mol%) was added and the reaction mixture was again purged with argon for 5 min. The resulting suspension was stirred at 90 °C for 3 days. The reaction mixture was left to cool to room temperature and filtered through a pad of celite using THF. The filtrate was concentrated by rotary evaporator, and under an atmosphere of argon, acetonitrile which had been purged with argon (2 ml) was added to the crude reaction product. White crystals formed from the resulting solution, which had been placed in a refrigerator overnight. The crystals were removed by filtration and dried to afford the title compound (146 mg, 72% yield).

NMR Spectroscopy:

^{1}H NMR (300 MHz, C_6D_6) δ 8.08 (t, *J* = 2.0 Hz, 1H), 7.56 – 7.45 (m, 1H), 7.38 (dd, *J* = 7.9, 3.8 Hz, 1H), 7.30 – 7.21 (m, 3H), 7.10 (d, *J* = 2.1 Hz, 1H), 6.66 (d, *J* = 8.3 Hz, 1H), 3.47 (s, 3H), 3.41 (s, 3H), 2.98 – 2.79 (m, 3H), 2.02 – 1.85 (m, 6H), 1.69 – 1.53 (m, 6H), 1.48 (d, *J* = 6.9 Hz, 6H), 1.35 – 1.17 (m, 20H), 1.16 – 1.12 (m, 2H).

^{13}C NMR (151 MHz, C_6D_6) δ 150.5, 150.0, 148.6, 147.0, 146.8, 146.6, 140.0, 137.9, 137.8, 137.0 (137.00), 137.0 (136.97), 134.6, 132.4, 132.3, 131.3, 131.2, 128.6, 126.9, 120.8, 119.8, 112.7, 112.0, 55.6, 55.5, 35.0, 34.9, 34.8, 31.7, 31.6, 31.3 (31.28), 31.3 (31.26), 29.8, 29.7, 28.0, 27.9, 27.7, 27.6, 26.9, 26.5, 24.4, 23.4 (23.42), 23.4 (23.41).

^{31}P NMR (122 MHz, C_6D_6) δ –11.5.

HRMS-ESI (m/z): Calculated for $C_{41}H_{57}O_2P$ $[M+H]^+$, 613.4169; Found: 613.4166.

*1,2-bis(3'-(dicyclohexylphosphaneyl)-2'',4'',6''-triisopropyl-[1,1':4',1''-terphenyl]-4-yl)ethyne (**28**)*

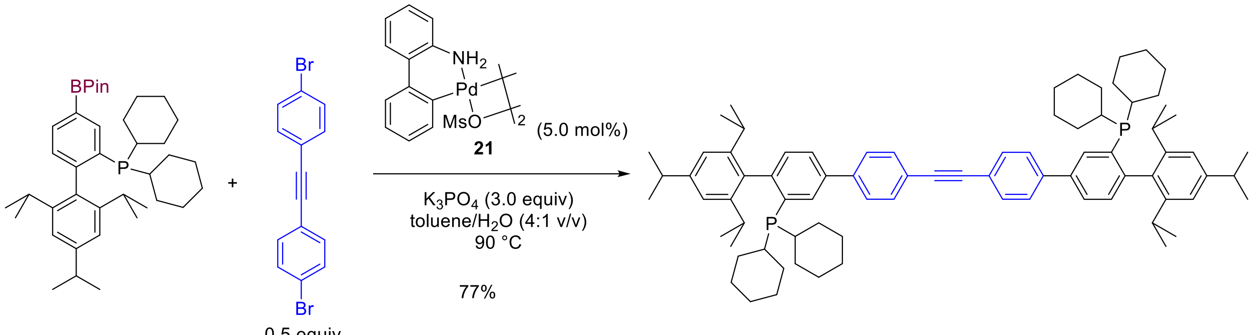

1, XPhos-BPin **28**

XPhos-Bpin (**1**, 200 mg, 0.332 mmol, 1.00 equiv), bis(4-bromophenyl)acetylene (55.8 mg, 0.166 mmol, 0.50 equiv), K_3PO_4 (211 mg, 0.996 mmol, 3.00 equiv), toluene (2.4 ml) and water (0.6 ml) were added to a Schlenk tube containing a stir bar. The resulting suspension was purged with argon for 15 min before the palladium catalyst (**21**, 12.3 mg, 16.6 µmol, 5.00 mol%) was added and the reaction mixture was again purged with argon for 5 min. The resulting suspension was stirred at 90 °C for 3 days. The reaction mixture was left to cool to room temperature and filtered through a pad of celite using THF. The filtrate was concentrated by rotary evaporator, and under an atmosphere of argon, a stir bar and acetonitrile which had been purged with argon (20 ml) was added to the crude reaction product. The suspension was stirred overnight at room temperature before the solid product was removed by filtration. Drying of the yellow solid afforded the title compound (144 mg, 77% yield).

NMR Spectroscopy:

^{1}H NMR (300 MHz, C_6D_6) δ 8.04 (s, 2H), 7.62 (d, *J* = 8.2 Hz, 4H), 7.53 (d, *J* = 8.4 Hz, 4H), 7.35 – 7.31 (m, 4H), 7.27 (s, 4H), 2.96 – 2.80 (m, 6H), 1.97 – 1.79 (m, 15H), 1.66 – 1.53 (m, 16H), 1.48 (d, *J* = 6.8 Hz, 12H), 1.32 – 1.27 (m, 18H), 1.22 – 1.17 (m, 19H).

^{13}C NMR (151 MHz, C_6D_6) δ 148.4, 147.5, 147.3, 146.5, 141.0, 138.3, 137.9, 137.8, 136.5, 136.4, 132.2 (132.24), 132.2 (132.18), 132.1, 130.6 (130.64), 130.6 (130.62), 127.1, 126.7, 122.6, 120.4, 90.6, 34.5 (34.50), 34.5 (34.46), 34.4, 31.3, 31.2, 30.9, 29.4, 29.3, 27.6, 27.5, 27.2 (27.22), 27.2 (27.16), 26.4, 26.1, 24.1, 23.0 (23.03), 23.0 (23.02).

^{31}P NMR (122 MHz, C_6D_6) δ –11.9.

HRMS-ESI (m/z): Calculated for $C_{80}H_{104}P_2$ $[M+H]^+$, 1127.7686; Found: 1127.7683.

*Borane adduct of borylated XPhos (**29**)*

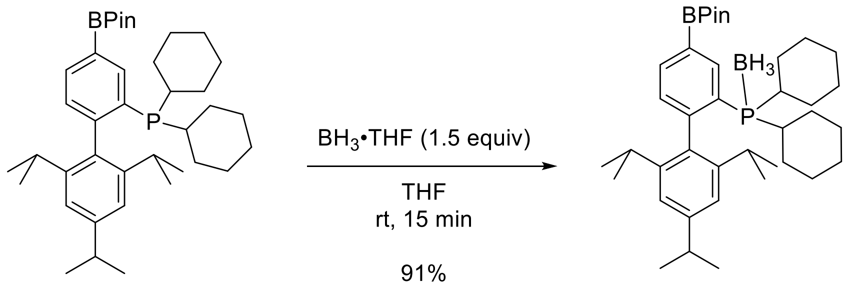

1, XPhos-BPin **29**

The transformation was performed according to a modified literature procedure.[32]

A solution of borylated XPhos (**1**, 120.0 mg, 199 µmol, 1.00 equiv) in THF (8 mL) in a 25 mL round-bottom flask containing a stir bar was purged with argon for 10 minutes before a 1M solution of BH_3·THF in THF (299 µL, 299 µmol, 1.50 equiv) was added. The resulting solution was left to stir until TLC analysis (7:1 hexanes : ethyl acetate) indicated completion. No starting material could be detected after 15 min, whereupon water (0.5 mL) was added to quench any remaining borane reagent. The reaction mixture was concentrated by rotary evaporator, after which THF (2 × 5 mL) was added and the solution concentrated again to remove water residue. To the resulting solid MeCN (10 mL) was added and the suspension was subjected to sonication for 5 minutes before the solid was removed by filtration and dried under vacuum at room temperature. The title compound was obtained as a white solid (112 mg, 91% yield).

NMR Spectroscopy:

^{1}H NMR (300 MHz, C_6D_6) δ 8.68 (dd, *J* = 8.6, 1.2 Hz, 1H), 8.09 (dt, *J* = 7.6, 1.4 Hz, 1H), 7.31 (dd, *J* = 7.6, 3.5 Hz, 1H), 7.21 (s, 2H), 2.91 (hept, *J* = 6.9 Hz, 1H), 2.67 (hept, *J* = 6.7 Hz, 2H), 2.19 – 2.01 (m, 2H), 1.99 – 1.76(m, 4H), 1.76 – 1.36 (m, 16H), 1.31 (d, *J* = 6.9 Hz, 6H), 1.08 (s, 12H), 1.05 (d, *J* = 6.7 Hz, 6H), 1.02 – 0.88 (m, 6H).

^{13}C NMR (75 MHz, C_6D_6) δ 151.4, 151.3, 149.6, 146.8, 140.6, 140.5, 136.9 (136.92), 136.9 (136.89), 136.7, 136.6, 134.7, 134.5, 121.0, 84.5, 36.0, 35.5, 35.2, 31.6, 28.5, 28.3, 27.8, 27.7, 27.5, 27.3, 26.7, 26.5, 25.2, 24.8, 23.0.

^{31}P NMR (122 MHz, C_6D_6) δ 29.3.

^{11}B NMR (96 MHz, C_6D_6) δ 34.6, –41.49.

HRMS-ESI (m/z): Calculated for $C_{39}H_{63}B_2O_2P$ $[M+Na]^+$, 639.4644; Found: 639.4647.

X-ray structures

X-ray structure of **1**

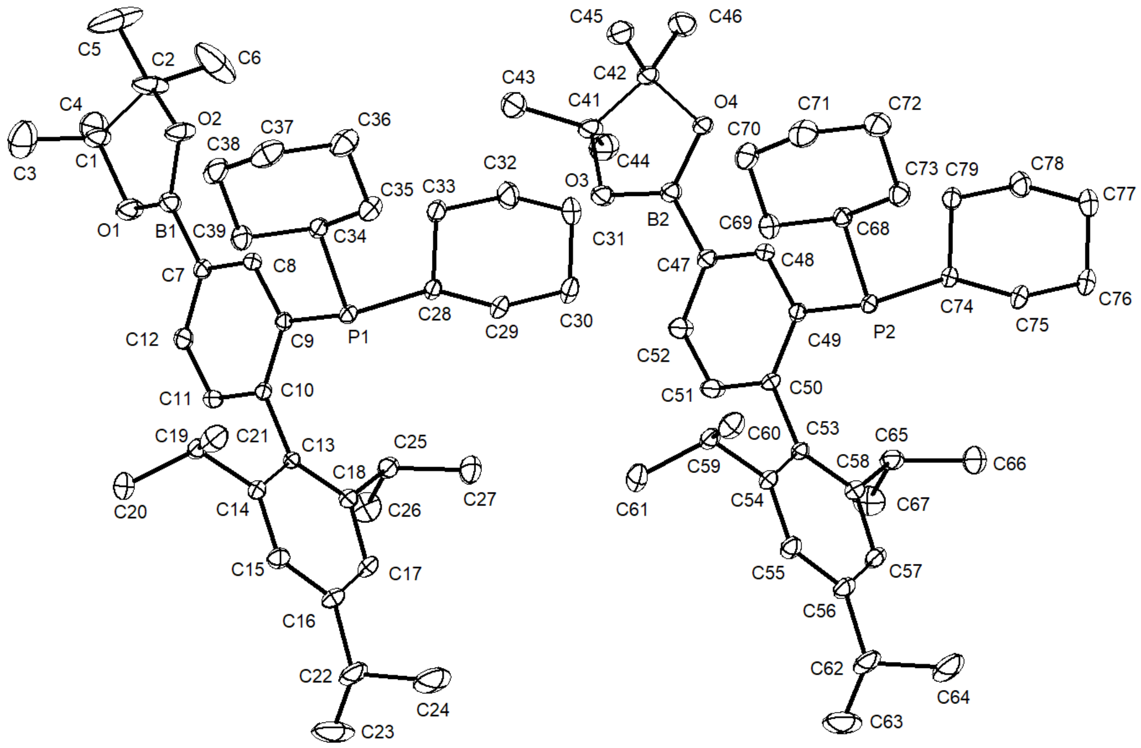

Molecular structure of **1**. H atoms have been removed for clarity.

$C_{39}H_{60}BO_2P$, M_r = 602.65 g mol^{-1}, colourless prism, crystal size 0.147 x 0.067 x 0.043 mm^3, triclinic, space group *P*-1 [2], *a* = 14.1297(13) Å, b = 15.1372(15) Å, *c* = 19.3066(18) Å, α= 94.165(4)°, β= 108.492(4)°, γ = 107.213(4)°, *V* = 3677.0(6) Å^3, *T* = 100(2) K, *Z* = 3, D_{calc} = 1.089 g·cm^3, λ = 0.71073 Å, $\mu(Mo\text{-}K_\alpha)$ = 0.105 mm^{-1}, Gaussian absorption correction ($T_{\min}$ = 0.98957, $T_{\max}$ 0.99707), Bruker AXS Enraf-Nonius KappaCCD diffractometer with a FR591 rotating Mo-anode X-ray source, 1.131 < θ < 30.508°, 120977 measured reflections, 22407 independent reflections, 14516 reflections with $I > 2\sigma(I)$, R_{int} = 0.0577. The structure was solved by *SHELXT* and refined by full-matrix least-squares (*SHELXL*) against F^2 to R_1 = 0.0510 [$I > 2\sigma(I)$], wR_2 = 0.1418 [all data], 795 parameters and 0 restraints.

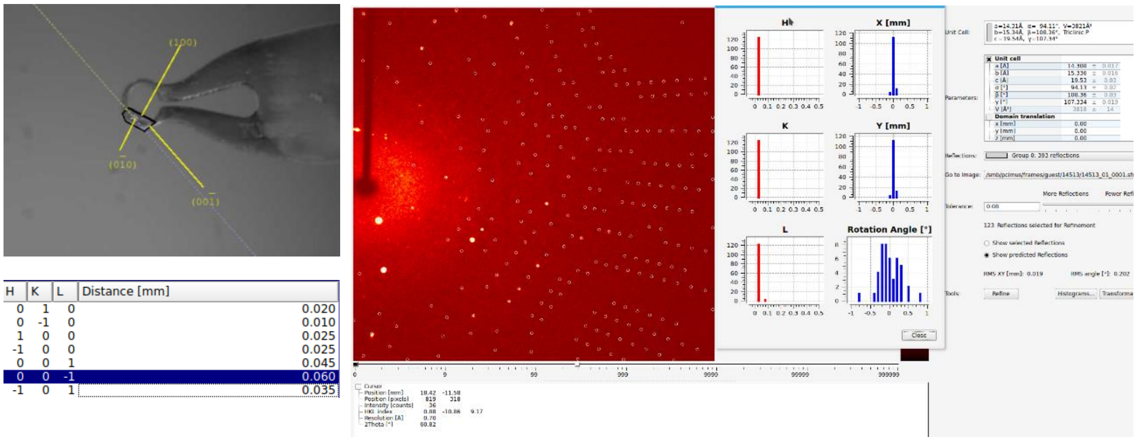

INTENSITY STATISTICS FOR DATASET

Resolution	#Data	#Theory	%Complete	Redundancy	Mean I	Mean I/s	Rmerge	Rsigma
Inf - 2.70	388	396	98.0	9.10	28.17	56.96	0.0196	0.0106
2.70 - 1.81	893	902	99.0	9.30	11.19	45.13	0.0256	0.0128
1.81 - 1.43	1318	1332	98.9	9.35	4.84	31.85	0.0397	0.0180
1.43 - 1.25	1302	1306	99.7	9.27	4.33	28.01	0.0450	0.0207
1.25 - 1.14	1259	1264	99.6	8.93	4.00	25.75	0.0521	0.0235
1.14 - 1.06	1271	1271	100.0	7.69	2.86	18.76	0.0685	0.0341
1.06 - 1.00	1241	1243	99.8	6.02	2.12	13.10	0.0885	0.0522
1.00 - 0.94	1535	1537	99.9	5.09	1.72	10.23	0.1049	0.0708
0.94 - 0.90	1318	1319	99.9	4.47	1.43	8.13	0.1256	0.0913
0.90 - 0.87	1120	1121	99.9	4.09	1.35	7.32	0.1267	0.1031
0.87 - 0.84	1300	1303	99.8	3.89	1.24	6.45	0.1395	0.1179
0.84 - 0.81	1518	1518	100.0	3.78	1.07	5.52	0.1669	0.1414
0.81 - 0.79	1088	1088	100.0	3.69	1.14	5.60	0.1630	0.1414
0.79 - 0.77	1287	1287	100.0	3.52	0.96	4.69	0.1963	0.1729
0.77 - 0.75	1355	1356	99.9	3.43	0.87	4.10	0.2162	0.1992
0.75 - 0.73	1588	1590	99.9	3.33	0.89	3.95	0.2243	0.2050
0.73 - 0.72	808	809	99.9	3.22	0.79	3.33	0.2612	0.2387
0.72 - 0.70	1823	1830	99.6	3.12	0.75	3.08	0.2768	0.2597
0.70 - 0.69	959	964	99.5	3.03	0.62	2.66	0.2969	0.3214
0.69 - 0.68	1065	1072	99.3	2.99	0.60	2.60	0.3161	0.3370
0.68 - 0.67	1109	1430	77.6	2.01	0.57	2.26	0.3260	0.3894
0.77 - 0.67	8707	9051	96.2	3.01	0.74	3.21	0.2610	0.2607
Inf - 0.67	25545	25938	98.5	5.01	2.45	11.93	0.0576	0.0620

The BPin subunit shows some residual electron density indicating a slightly rotational disorder over two positions with less than 10% occupancy. Modelling this disorder led to a Platon/CheckCIF alert level B (PLAT112_ALERT_2_B ADDSYM Detects New (Pseudo) Symm. Elem c/2 96%). This pseudo symmetry was tested by overlaying the two molecules of the asymmetric unit. This indicates that the c axis of the unit cell has been assigned correctly. Therefore, we decided not to model the disorder and uploaded the structure in the current version. A resolution cut off (SHEL 99 0.7) was applied to the data set to exclude poorly determined

intensities at higher diffraction angles. Complete .cif-data of the compound are available under the CCDC number **CCDC- 2174990**.

Crystal data and structure refinement

Identification code	**14513**	
Empirical formula	$C_{39}H_{60}BO_2P$	
Color	colourless	
Formula weight	602.65 g·mol^{-1}	
Temperature	100(2) K	
Wavelength	0.71073 Å	
Crystal system	triclinic	
Space group	*P*-1, (no. 2)	
Unit cell dimensions	a = 14.1297(13) Å	α= 94.165(4)°.
	b = 15.1372(15) Å	β= 108.492(4)°.
	c = 19.3066(18) Å	γ = 107.213(4)°.
Volume	3677.0(6) Å^3	
Z	4	
Density (calculated)	1.089 Mg·m^{-3}	
Absorption coefficient	0.105 mm^{-1}	
F(000)	1320 e	
Crystal size	0.147 x 0.067 x 0.043 mm^3	
θ range for data collection	1.131 to 30.508°.	
Index ranges	$-20 \le h \le 20$, $-21 \le k \le 21$, $-27 \le l \le 27$	
Reflections collected	120977	
Independent reflections	22407 [R_{int} = 0.0577]	
Reflections with I>2σ(I)	14516	
Completeness to θ = 25.242°	99.6 %	
Absorption correction	Gaussian	
Max. and min. transmission	0.99707 and 0.98957	
Refinement method	Full-matrix least-squares on F^2	
Data / restraints / parameters	22407 / 0 / 795	
Goodness-of-fit on F^2	1.021	
Final R indices [I>2σ(I)]	R_1 = 0.0510	wR^2 = 0.1233
R indices (all data)	R_1 = 0.0935	wR^2 = 0.1418
Extinction coefficient	n/a	

Largest diff. peak and hole 1.048 and -0.305 $e \cdot Å^{-3}$

Bond lengths [Å] and angles [°]

P(1)-C(9)	1.8458(13)	P(1)-C(28)	1.8749(14)
P(1)-C(34)	1.8626(14)	O(1)-C(1)	1.4628(18)
O(1)-B(1)	1.3779(19)	O(2)-C(2)	1.4539(19)
O(2)-B(1)	1.3577(19)	C(1)-C(2)	1.561(2)
C(1)-C(3)	1.505(2)	C(1)-C(4)	1.508(2)
C(2)-C(5)	1.492(3)	C(2)-C(6)	1.532(3)
C(3)-H(3A)	0.9800	C(3)-H(3B)	0.9800
C(3)-H(3C)	0.9800	C(4)-H(4A)	0.9800
C(4)-H(4B)	0.9800	C(4)-H(4C)	0.9800
C(5)-H(5A)	0.9800	C(5)-H(5B)	0.9800
C(5)-H(5C)	0.9800	C(6)-H(6A)	0.9800
C(6)-H(6B)	0.9800	C(6)-H(6C)	0.9800
C(7)-C(8)	1.3996(18)	C(7)-C(12)	1.3954(19)
C(7)-B(1)	1.550(2)	C(8)-H(8)	0.9500
C(8)-C(9)	1.4014(18)	C(9)-C(10)	1.4074(18)
C(10)-C(11)	1.3970(18)	C(10)-C(13)	1.5006(18)
C(11)-H(11)	0.9500	C(11)-C(12)	1.3826(19)
C(12)-H(12)	0.9500	C(13)-C(14)	1.405(2)
C(13)-C(18)	1.4056(19)	C(14)-C(15)	1.3904(19)
C(14)-C(19)	1.5216(19)	C(15)-H(15)	0.9500
C(15)-C(16)	1.394(2)	C(16)-C(17)	1.390(2)
C(16)-C(22)	1.517(2)	C(17)-H(17)	0.9500
C(17)-C(18)	1.3938(19)	C(18)-C(25)	1.521(2)
C(19)-H(19)	1.0000	C(19)-C(20)	1.529(2)
C(19)-C(21)	1.531(2)	C(20)-H(20A)	0.9800
C(20)-H(20B)	0.9800	C(20)-H(20C)	0.9800
C(21)-H(21A)	0.9800	C(21)-H(21B)	0.9800
C(21)-H(21C)	0.9800	C(22)-H(22)	1.0000
C(22)-C(23)	1.501(3)	C(22)-C(24)	1.505(2)
C(23)-H(23A)	0.9800	C(23)-H(23B)	0.9800
C(23)-H(23C)	0.9800	C(24)-H(24A)	0.9800
C(24)-H(24B)	0.9800	C(24)-H(24C)	0.9800

C(25)-H(25)	1.0000	C(25)-C(26)	1.527(2)
C(25)-C(27)	1.530(2)	C(26)-H(26A)	0.9800
C(26)-H(26B)	0.9800	C(26)-H(26C)	0.9800
C(27)-H(27A)	0.9800	C(27)-H(27B)	0.9800
C(27)-H(27C)	0.9800	C(28)-H(28)	1.0000
C(28)-C(29)	1.5348(18)	C(28)-C(33)	1.5301(19)
C(29)-H(29A)	0.9900	C(29)-H(29B)	0.9900
C(29)-C(30)	1.527(2)	C(30)-H(30A)	0.9900
C(30)-H(30B)	0.9900	C(30)-C(31)	1.520(2)
C(31)-H(31A)	0.9900	C(31)-H(31B)	0.9900
C(31)-C(32)	1.523(2)	C(32)-H(32A)	0.9900
C(32)-H(32B)	0.9900	C(32)-C(33)	1.5298(19)
C(33)-H(33A)	0.9900	C(33)-H(33B)	0.9900
C(34)-H(34)	1.0000	C(34)-C(35)	1.5339(19)
C(34)-C(39)	1.5354(19)	C(35)-H(35A)	0.9900
C(35)-H(35B)	0.9900	C(35)-C(36)	1.526(2)
C(36)-H(36A)	0.9900	C(36)-H(36B)	0.9900
C(36)-C(37)	1.524(2)	C(37)-H(37A)	0.9900
C(37)-H(37B)	0.9900	C(37)-C(38)	1.518(2)
C(38)-H(38A)	0.9900	C(38)-H(38B)	0.9900
C(38)-C(39)	1.528(2)	C(39)-H(39A)	0.9900
C(39)-H(39B)	0.9900	P(2)-C(49)	1.8449(13)
P(2)-C(68)	1.8612(14)	P(2)-C(74)	1.8769(14)
O(3)-C(41)	1.4609(18)	O(3)-B(2)	1.3660(17)
O(4)-C(42)	1.4609(16)	O(4)-B(2)	1.3652(18)
C(41)-C(42)	1.5687(19)	C(41)-C(43)	1.514(2)
C(41)-C(44)	1.518(2)	C(42)-C(45)	1.524(2)
C(42)-C(46)	1.507(2)	C(43)-H(43A)	0.9800
C(43)-H(43B)	0.9800	C(43)-H(43C)	0.9800
C(44)-H(44A)	0.9800	C(44)-H(44B)	0.9800
C(44)-H(44C)	0.9800	C(45)-H(45A)	0.9800
C(45)-H(45B)	0.9800	C(45)-H(45C)	0.9800
C(46)-H(46A)	0.9800	C(46)-H(46B)	0.9800
C(46)-H(46C)	0.9800	C(47)-C(48)	1.3946(18)
C(47)-C(52)	1.3924(19)	C(47)-B(2)	1.553(2)
C(48)-H(48)	0.9500	C(48)-C(49)	1.4049(18)

C(49)-C(50)	1.4109(18)	C(50)-C(51)	1.3979(18)
C(50)-C(53)	1.4991(18)	C(51)-H(51)	0.9500
C(51)-C(52)	1.382(2)	C(52)-H(52)	0.9500
C(53)-C(54)	1.405(2)	C(53)-C(58)	1.4071(19)
C(54)-C(55)	1.3922(19)	C(54)-C(59)	1.5179(19)
C(55)-H(55)	0.9500	C(55)-C(56)	1.3907(19)
C(56)-C(57)	1.390(2)	C(56)-C(62)	1.517(2)
C(57)-H(57)	0.9500	C(57)-C(58)	1.3897(19)
C(58)-C(65)	1.518(2)	C(59)-H(59)	1.0000
C(59)-C(60)	1.528(2)	C(59)-C(61)	1.528(2)
C(60)-H(60A)	0.9800	C(60)-H(60B)	0.9800
C(60)-H(60C)	0.9800	C(61)-H(61A)	0.9800
C(61)-H(61B)	0.9800	C(61)-H(61C)	0.9800
C(62)-H(62)	1.0000	C(62)-C(63)	1.513(2)
C(62)-C(64)	1.523(2)	C(63)-H(63A)	0.9800
C(63)-H(63B)	0.9800	C(63)-H(63C)	0.9800
C(64)-H(64A)	0.9800	C(64)-H(64B)	0.9800
C(64)-H(64C)	0.9800	C(65)-H(65)	1.0000
C(65)-C(66)	1.530(2)	C(65)-C(67)	1.531(2)
C(66)-H(66A)	0.9800	C(66)-H(66B)	0.9800
C(66)-H(66C)	0.9800	C(67)-H(67A)	0.9800
C(67)-H(67B)	0.9800	C(67)-H(67C)	0.9800
C(68)-H(68)	1.0000	C(68)-C(69)	1.5337(19)
C(68)-C(73)	1.5328(18)	C(69)-H(69A)	0.9900
C(69)-H(69B)	0.9900	C(69)-C(70)	1.527(2)
C(70)-H(70A)	0.9900	C(70)-H(70B)	0.9900
C(70)-C(71)	1.518(2)	C(71)-H(71A)	0.9900
C(71)-H(71B)	0.9900	C(71)-C(72)	1.524(2)
C(72)-H(72A)	0.9900	C(72)-H(72B)	0.9900
C(72)-C(73)	1.527(2)	C(73)-H(73A)	0.9900
C(73)-H(73B)	0.9900	C(74)-H(74)	1.0000
C(74)-C(75)	1.5355(19)	C(74)-C(79)	1.5329(19)
C(75)-H(75A)	0.9900	C(75)-H(75B)	0.9900
C(75)-C(76)	1.524(2)	C(76)-H(76A)	0.9900
C(76)-H(76B)	0.9900	C(76)-C(77)	1.523(2)
C(77)-H(77A)	0.9900	C(77)-H(77B)	0.9900

C(77)-C(78)	1.520(2)	C(78)-H(78A)	0.9900
C(78)-H(78B)	0.9900	C(78)-C(79)	1.5290(19)
C(79)-H(79A)	0.9900	C(79)-H(79B)	0.9900
C(9)-P(1)-C(28)	100.55(6)	C(9)-P(1)-C(34)	101.83(6)
C(34)-P(1)-C(28)	104.71(6)	B(1)-O(1)-C(1)	106.72(11)
B(1)-O(2)-C(2)	107.89(12)	O(1)-C(1)-C(2)	102.14(11)
O(1)-C(1)-C(3)	106.71(13)	O(1)-C(1)-C(4)	108.61(13)
C(3)-C(1)-C(2)	113.44(15)	C(3)-C(1)-C(4)	110.51(13)
C(4)-C(1)-C(2)	114.70(14)	O(2)-C(2)-C(1)	102.72(12)
O(2)-C(2)-C(5)	110.01(16)	O(2)-C(2)-C(6)	105.47(15)
C(5)-C(2)-C(1)	114.60(16)	C(5)-C(2)-C(6)	110.92(19)
C(6)-C(2)-C(1)	112.40(16)	C(1)-C(3)-H(3A)	109.5
C(1)-C(3)-H(3B)	109.5	C(1)-C(3)-H(3C)	109.5
H(3A)-C(3)-H(3B)	109.5	H(3A)-C(3)-H(3C)	109.5
H(3B)-C(3)-H(3C)	109.5	C(1)-C(4)-H(4A)	109.5
C(1)-C(4)-H(4B)	109.5	C(1)-C(4)-H(4C)	109.5
H(4A)-C(4)-H(4B)	109.5	H(4A)-C(4)-H(4C)	109.5
H(4B)-C(4)-H(4C)	109.5	C(2)-C(5)-H(5A)	109.5
C(2)-C(5)-H(5B)	109.5	C(2)-C(5)-H(5C)	109.5
H(5A)-C(5)-H(5B)	109.5	H(5A)-C(5)-H(5C)	109.5
H(5B)-C(5)-H(5C)	109.5	C(2)-C(6)-H(6A)	109.5
C(2)-C(6)-H(6B)	109.5	C(2)-C(6)-H(6C)	109.5
H(6A)-C(6)-H(6B)	109.5	H(6A)-C(6)-H(6C)	109.5
H(6B)-C(6)-H(6C)	109.5	C(8)-C(7)-B(1)	122.52(12)
C(12)-C(7)-C(8)	117.69(12)	C(12)-C(7)-B(1)	119.78(12)
C(7)-C(8)-H(8)	118.7	C(7)-C(8)-C(9)	122.58(12)
C(9)-C(8)-H(8)	118.7	C(8)-C(9)-P(1)	123.84(10)
C(8)-C(9)-C(10)	118.20(12)	C(10)-C(9)-P(1)	117.93(10)
C(9)-C(10)-C(13)	123.68(11)	C(11)-C(10)-C(9)	119.52(12)
C(11)-C(10)-C(13)	116.78(11)	C(10)-C(11)-H(11)	119.5
C(12)-C(11)-C(10)	121.02(12)	C(12)-C(11)-H(11)	119.5
C(7)-C(12)-H(12)	119.5	C(11)-C(12)-C(7)	120.98(12)
C(11)-C(12)-H(12)	119.5	C(14)-C(13)-C(10)	119.88(12)
C(14)-C(13)-C(18)	120.04(12)	C(18)-C(13)-C(10)	119.82(12)
C(13)-C(14)-C(19)	121.22(12)	C(15)-C(14)-C(13)	118.99(12)

C(15)-C(14)-C(19)	119.79(13)	C(14)-C(15)-H(15)	118.9
C(14)-C(15)-C(16)	122.12(14)	C(16)-C(15)-H(15)	118.9
C(15)-C(16)-C(22)	120.76(14)	C(17)-C(16)-C(15)	117.79(13)
C(17)-C(16)-C(22)	121.45(13)	C(16)-C(17)-H(17)	118.9
C(16)-C(17)-C(18)	122.15(13)	C(18)-C(17)-H(17)	118.9
C(13)-C(18)-C(25)	121.72(12)	C(17)-C(18)-C(13)	118.87(13)
C(17)-C(18)-C(25)	119.40(12)	C(14)-C(19)-H(19)	107.6
C(14)-C(19)-C(20)	111.54(12)	C(14)-C(19)-C(21)	111.56(12)
C(20)-C(19)-H(19)	107.6	C(20)-C(19)-C(21)	110.86(13)
C(21)-C(19)-H(19)	107.6	C(19)-C(20)-H(20A)	109.5
C(19)-C(20)-H(20B)	109.5	C(19)-C(20)-H(20C)	109.5
H(20A)-C(20)-H(20B)	109.5	H(20A)-C(20)-H(20C)	109.5
H(20B)-C(20)-H(20C)	109.5	C(19)-C(21)-H(21A)	109.5
C(19)-C(21)-H(21B)	109.5	C(19)-C(21)-H(21C)	109.5
H(21A)-C(21)-H(21B)	109.5	H(21A)-C(21)-H(21C)	109.5
H(21B)-C(21)-H(21C)	109.5	C(16)-C(22)-H(22)	107.2
C(23)-C(22)-C(16)	111.70(14)	C(23)-C(22)-H(22)	107.2
C(23)-C(22)-C(24)	110.20(16)	C(24)-C(22)-C(16)	113.01(14)
C(24)-C(22)-H(22)	107.2	C(22)-C(23)-H(23A)	109.5
C(22)-C(23)-H(23B)	109.5	C(22)-C(23)-H(23C)	109.5
H(23A)-C(23)-H(23B)	109.5	H(23A)-C(23)-H(23C)	109.5
H(23B)-C(23)-H(23C)	109.5	C(22)-C(24)-H(24A)	109.5
C(22)-C(24)-H(24B)	109.5	C(22)-C(24)-H(24C)	109.5
H(24A)-C(24)-H(24B)	109.5	H(24A)-C(24)-H(24C)	109.5
H(24B)-C(24)-H(24C)	109.5	C(18)-C(25)-H(25)	107.5
C(18)-C(25)-C(26)	111.34(12)	C(18)-C(25)-C(27)	112.03(12)
C(26)-C(25)-H(25)	107.5	C(26)-C(25)-C(27)	110.71(13)
C(27)-C(25)-H(25)	107.5	C(25)-C(26)-H(26A)	109.5
C(25)-C(26)-H(26B)	109.5	C(25)-C(26)-H(26C)	109.5
H(26A)-C(26)-H(26B)	109.5	H(26A)-C(26)-H(26C)	109.5
H(26B)-C(26)-H(26C)	109.5	C(25)-C(27)-H(27A)	109.5
C(25)-C(27)-H(27B)	109.5	C(25)-C(27)-H(27C)	109.5
H(27A)-C(27)-H(27B)	109.5	H(27A)-C(27)-H(27C)	109.5
H(27B)-C(27)-H(27C)	109.5	P(1)-C(28)-H(28)	106.3
C(29)-C(28)-P(1)	110.08(10)	C(29)-C(28)-H(28)	106.3
C(33)-C(28)-P(1)	116.07(9)	C(33)-C(28)-H(28)	106.3

C(33)-C(28)-C(29)	111.13(11)	C(28)-C(29)-H(29A)	109.1
C(28)-C(29)-H(29B)	109.1	H(29A)-C(29)-H(29B)	107.8
C(30)-C(29)-C(28)	112.44(12)	C(30)-C(29)-H(29A)	109.1
C(30)-C(29)-H(29B)	109.1	C(29)-C(30)-H(30A)	109.4
C(29)-C(30)-H(30B)	109.4	H(30A)-C(30)-H(30B)	108.0
C(31)-C(30)-C(29)	111.36(12)	C(31)-C(30)-H(30A)	109.4
C(31)-C(30)-H(30B)	109.4	C(30)-C(31)-H(31A)	109.5
C(30)-C(31)-H(31B)	109.5	C(30)-C(31)-C(32)	110.51(12)
H(31A)-C(31)-H(31B)	108.1	C(32)-C(31)-H(31A)	109.5
C(32)-C(31)-H(31B)	109.5	C(31)-C(32)-H(32A)	109.4
C(31)-C(32)-H(32B)	109.4	C(31)-C(32)-C(33)	110.99(13)
H(32A)-C(32)-H(32B)	108.0	C(33)-C(32)-H(32A)	109.4
C(33)-C(32)-H(32B)	109.4	C(28)-C(33)-H(33A)	109.2
C(28)-C(33)-H(33B)	109.2	C(32)-C(33)-C(28)	112.23(11)
C(32)-C(33)-H(33A)	109.2	C(32)-C(33)-H(33B)	109.2
H(33A)-C(33)-H(33B)	107.9	P(1)-C(34)-H(34)	109.4
C(35)-C(34)-P(1)	109.71(9)	C(35)-C(34)-H(34)	109.4
C(35)-C(34)-C(39)	110.00(12)	C(39)-C(34)-P(1)	109.06(9)
C(39)-C(34)-H(34)	109.4	C(34)-C(35)-H(35A)	109.2
C(34)-C(35)-H(35B)	109.2	H(35A)-C(35)-H(35B)	107.9
C(36)-C(35)-C(34)	112.04(12)	C(36)-C(35)-H(35A)	109.2
C(36)-C(35)-H(35B)	109.2	C(35)-C(36)-H(36A)	109.3
C(35)-C(36)-H(36B)	109.3	H(36A)-C(36)-H(36B)	108.0
C(37)-C(36)-C(35)	111.51(13)	C(37)-C(36)-H(36A)	109.3
C(37)-C(36)-H(36B)	109.3	C(36)-C(37)-H(37A)	109.5
C(36)-C(37)-H(37B)	109.5	H(37A)-C(37)-H(37B)	108.1
C(38)-C(37)-C(36)	110.83(13)	C(38)-C(37)-H(37A)	109.5
C(38)-C(37)-H(37B)	109.5	C(37)-C(38)-H(38A)	109.5
C(37)-C(38)-H(38B)	109.5	C(37)-C(38)-C(39)	110.91(13)
H(38A)-C(38)-H(38B)	108.0	C(39)-C(38)-H(38A)	109.5
C(39)-C(38)-H(38B)	109.5	C(34)-C(39)-H(39A)	109.3
C(34)-C(39)-H(39B)	109.3	C(38)-C(39)-C(34)	111.73(12)
C(38)-C(39)-H(39A)	109.3	C(38)-C(39)-H(39B)	109.3
H(39A)-C(39)-H(39B)	107.9	O(1)-B(1)-C(7)	122.50(13)
O(2)-B(1)-O(1)	113.17(13)	O(2)-B(1)-C(7)	124.28(13)
C(49)-P(2)-C(68)	100.76(6)	C(49)-P(2)-C(74)	101.77(6)

C(68)-P(2)-C(74)	103.77(6)	B(2)-O(3)-C(41)	107.47(11)
B(2)-O(4)-C(42)	107.56(10)	O(3)-C(41)-C(42)	102.35(10)
O(3)-C(41)-C(43)	108.40(13)	O(3)-C(41)-C(44)	106.95(12)
C(43)-C(41)-C(42)	114.34(13)	C(43)-C(41)-C(44)	110.38(12)
C(44)-C(41)-C(42)	113.73(13)	O(4)-C(42)-C(41)	102.53(11)
O(4)-C(42)-C(45)	105.95(12)	O(4)-C(42)-C(46)	109.31(12)
C(45)-C(42)-C(41)	113.70(13)	C(46)-C(42)-C(41)	114.51(13)
C(46)-C(42)-C(45)	110.13(13)	C(41)-C(43)-H(43A)	109.5
C(41)-C(43)-H(43B)	109.5	C(41)-C(43)-H(43C)	109.5
H(43A)-C(43)-H(43B)	109.5	H(43A)-C(43)-H(43C)	109.5
H(43B)-C(43)-H(43C)	109.5	C(41)-C(44)-H(44A)	109.5
C(41)-C(44)-H(44B)	109.5	C(41)-C(44)-H(44C)	109.5
H(44A)-C(44)-H(44B)	109.5	H(44A)-C(44)-H(44C)	109.5
H(44B)-C(44)-H(44C)	109.5	C(42)-C(45)-H(45A)	109.5
C(42)-C(45)-H(45B)	109.5	C(42)-C(45)-H(45C)	109.5
H(45A)-C(45)-H(45B)	109.5	H(45A)-C(45)-H(45C)	109.5
H(45B)-C(45)-H(45C)	109.5	C(42)-C(46)-H(46A)	109.5
C(42)-C(46)-H(46B)	109.5	C(42)-C(46)-H(46C)	109.5
H(46A)-C(46)-H(46B)	109.5	H(46A)-C(46)-H(46C)	109.5
H(46B)-C(46)-H(46C)	109.5	C(48)-C(47)-B(2)	122.95(12)
C(52)-C(47)-C(48)	117.51(12)	C(52)-C(47)-B(2)	119.54(12)
C(47)-C(48)-H(48)	118.6	C(47)-C(48)-C(49)	122.77(12)
C(49)-C(48)-H(48)	118.6	C(48)-C(49)-P(2)	123.29(10)
C(48)-C(49)-C(50)	118.15(11)	C(50)-C(49)-P(2)	118.47(10)
C(49)-C(50)-C(53)	124.10(11)	C(51)-C(50)-C(49)	119.18(12)
C(51)-C(50)-C(53)	116.71(11)	C(50)-C(51)-H(51)	119.5
C(52)-C(51)-C(50)	121.03(13)	C(52)-C(51)-H(51)	119.5
C(47)-C(52)-H(52)	119.4	C(51)-C(52)-C(47)	121.29(13)
C(51)-C(52)-H(52)	119.4	C(54)-C(53)-C(50)	119.61(12)
C(54)-C(53)-C(58)	119.79(12)	C(58)-C(53)-C(50)	120.40(12)
C(53)-C(54)-C(59)	121.45(12)	C(55)-C(54)-C(53)	119.09(12)
C(55)-C(54)-C(59)	119.46(13)	C(54)-C(55)-H(55)	118.9
C(56)-C(55)-C(54)	122.16(13)	C(56)-C(55)-H(55)	118.9
C(55)-C(56)-C(62)	120.81(13)	C(57)-C(56)-C(55)	117.61(13)
C(57)-C(56)-C(62)	121.58(13)	C(56)-C(57)-H(57)	118.8
C(58)-C(57)-C(56)	122.46(13)	C(58)-C(57)-H(57)	118.8

C(53)-C(58)-C(65)	121.56(12)	C(57)-C(58)-C(53)	118.88(13)
C(57)-C(58)-C(65)	119.55(12)	C(54)-C(59)-H(59)	107.4
C(54)-C(59)-C(60)	111.55(11)	C(54)-C(59)-C(61)	111.42(12)
C(60)-C(59)-H(59)	107.4	C(61)-C(59)-H(59)	107.4
C(61)-C(59)-C(60)	111.39(13)	C(59)-C(60)-H(60A)	109.5
C(59)-C(60)-H(60B)	109.5	C(59)-C(60)-H(60C)	109.5
H(60A)-C(60)-H(60B)	109.5	H(60A)-C(60)-H(60C)	109.5
H(60B)-C(60)-H(60C)	109.5	C(59)-C(61)-H(61A)	109.5
C(59)-C(61)-H(61B)	109.5	C(59)-C(61)-H(61C)	109.5
H(61A)-C(61)-H(61B)	109.5	H(61A)-C(61)-H(61C)	109.5
H(61B)-C(61)-H(61C)	109.5	C(56)-C(62)-H(62)	107.5
C(56)-C(62)-C(64)	112.59(13)	C(63)-C(62)-C(56)	111.40(13)
C(63)-C(62)-H(62)	107.5	C(63)-C(62)-C(64)	110.19(14)
C(64)-C(62)-H(62)	107.5	C(62)-C(63)-H(63A)	109.5
C(62)-C(63)-H(63B)	109.5	C(62)-C(63)-H(63C)	109.5
H(63A)-C(63)-H(63B)	109.5	H(63A)-C(63)-H(63C)	109.5
H(63B)-C(63)-H(63C)	109.5	C(62)-C(64)-H(64A)	109.5
C(62)-C(64)-H(64B)	109.5	C(62)-C(64)-H(64C)	109.5
H(64A)-C(64)-H(64B)	109.5	H(64A)-C(64)-H(64C)	109.5
H(64B)-C(64)-H(64C)	109.5	C(58)-C(65)-H(65)	107.5
C(58)-C(65)-C(66)	111.31(12)	C(58)-C(65)-C(67)	111.68(12)
C(66)-C(65)-H(65)	107.5	C(66)-C(65)-C(67)	111.21(13)
C(67)-C(65)-H(65)	107.5	C(65)-C(66)-H(66A)	109.5
C(65)-C(66)-H(66B)	109.5	C(65)-C(66)-H(66C)	109.5
H(66A)-C(66)-H(66B)	109.5	H(66A)-C(66)-H(66C)	109.5
H(66B)-C(66)-H(66C)	109.5	C(65)-C(67)-H(67A)	109.5
C(65)-C(67)-H(67B)	109.5	C(65)-C(67)-H(67C)	109.5
H(67A)-C(67)-H(67B)	109.5	H(67A)-C(67)-H(67C)	109.5
H(67B)-C(67)-H(67C)	109.5	P(2)-C(68)-H(68)	108.9
C(69)-C(68)-P(2)	110.07(9)	C(69)-C(68)-H(68)	108.9
C(73)-C(68)-P(2)	110.58(9)	C(73)-C(68)-H(68)	108.9
C(73)-C(68)-C(69)	109.59(11)	C(68)-C(69)-H(69A)	109.4
C(68)-C(69)-H(69B)	109.4	H(69A)-C(69)-H(69B)	108.0
C(70)-C(69)-C(68)	111.02(12)	C(70)-C(69)-H(69A)	109.4
C(70)-C(69)-H(69B)	109.4	C(69)-C(70)-H(70A)	109.4
C(69)-C(70)-H(70B)	109.4	H(70A)-C(70)-H(70B)	108.0

C(71)-C(70)-C(69)	111.06(12)	C(71)-C(70)-H(70A)	109.4
C(71)-C(70)-H(70B)	109.4	C(70)-C(71)-H(71A)	109.4
C(70)-C(71)-H(71B)	109.4	C(70)-C(71)-C(72)	111.11(13)
H(71A)-C(71)-H(71B)	108.0	C(72)-C(71)-H(71A)	109.4
C(72)-C(71)-H(71B)	109.4	C(71)-C(72)-H(72A)	109.4
C(71)-C(72)-H(72B)	109.4	C(71)-C(72)-C(73)	110.95(12)
H(72A)-C(72)-H(72B)	108.0	C(73)-C(72)-H(72A)	109.4
C(73)-C(72)-H(72B)	109.4	C(68)-C(73)-H(73A)	109.3
C(68)-C(73)-H(73B)	109.3	C(72)-C(73)-C(68)	111.61(11)
C(72)-C(73)-H(73A)	109.3	C(72)-C(73)-H(73B)	109.3
H(73A)-C(73)-H(73B)	108.0	P(2)-C(74)-H(74)	106.0
C(75)-C(74)-P(2)	109.53(10)	C(75)-C(74)-H(74)	106.0
C(79)-C(74)-P(2)	117.56(9)	C(79)-C(74)-H(74)	106.0
C(79)-C(74)-C(75)	111.06(11)	C(74)-C(75)-H(75A)	109.1
C(74)-C(75)-H(75B)	109.1	H(75A)-C(75)-H(75B)	107.9
C(76)-C(75)-C(74)	112.29(13)	C(76)-C(75)-H(75A)	109.1
C(76)-C(75)-H(75B)	109.1	C(75)-C(76)-H(76A)	109.4
C(75)-C(76)-H(76B)	109.4	H(76A)-C(76)-H(76B)	108.0
C(77)-C(76)-C(75)	111.27(12)	C(77)-C(76)-H(76A)	109.4
C(77)-C(76)-H(76B)	109.4	C(76)-C(77)-H(77A)	109.5
C(76)-C(77)-H(77B)	109.5	H(77A)-C(77)-H(77B)	108.1
C(78)-C(77)-C(76)	110.54(12)	C(78)-C(77)-H(77A)	109.5
C(78)-C(77)-H(77B)	109.5	C(77)-C(78)-H(78A)	109.3
C(77)-C(78)-H(78B)	109.3	C(77)-C(78)-C(79)	111.77(13)
H(78A)-C(78)-H(78B)	107.9	C(79)-C(78)-H(78A)	109.3
C(79)-C(78)-H(78B)	109.3	C(74)-C(79)-H(79A)	109.3
C(74)-C(79)-H(79B)	109.3	C(78)-C(79)-C(74)	111.81(11)
C(78)-C(79)-H(79A)	109.3	C(78)-C(79)-H(79B)	109.3
H(79A)-C(79)-H(79B)	107.9	O(3)-B(2)-C(47)	121.98(13)
O(4)-B(2)-O(3)	113.50(12)	O(4)-B(2)-C(47)	124.52(12)

X-ray structure of **18**

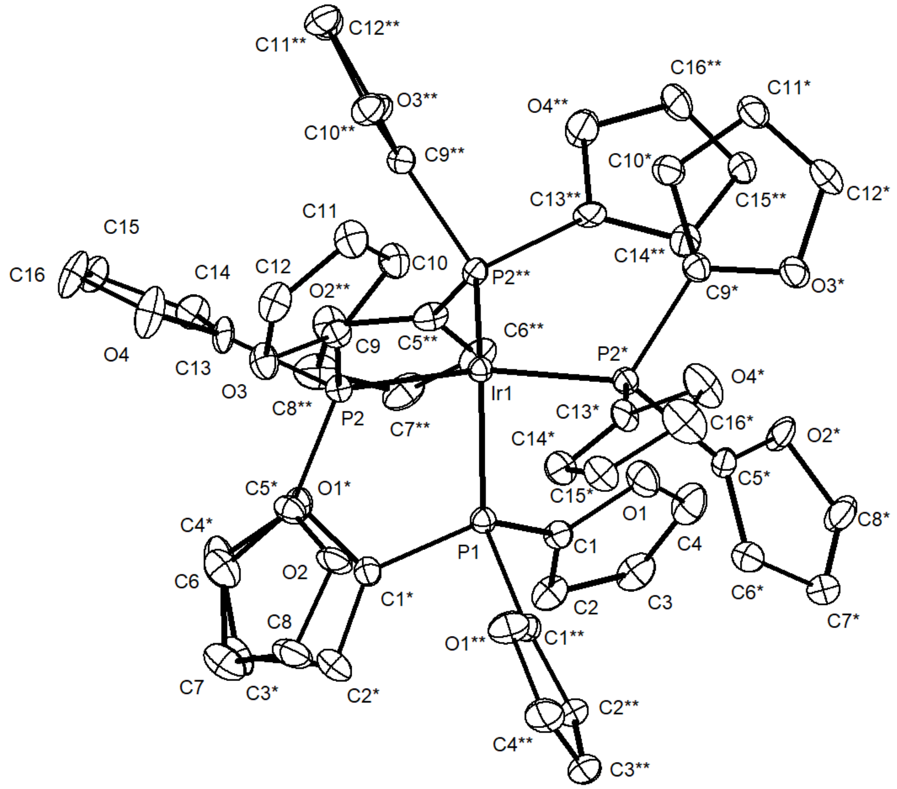

Molecular structure of **18**. H atoms have been removed for clarity.

X-ray Crystal Structure Analysis

$C_{48}H_{37}IrO_{12}P_4$, M_r = 1121.85 g mol^{-1}, pale yellow needle, crystal size 0.10 x 0.03 x 0.03 mm^3, trigonal, space group *R*3 [146], a = 19.1442(13) Å, c = 10.0582(14) Å, V = 3192.5(6) Å^3, T = 100(2) K, Z = 3, D_{calc} = 1.751 g·cm^3, λ = 0.71073 Å, $\mu(Mo\text{-}K_\alpha)$ = 3.355 mm^{-1}, Gaussian absorption correction ($T_{\min}$ = 0.98964, $T_{\max}$ = 0.99665), Bruker AXS Enraf-Nonius KappaCCD diffractometer with a FR591 rotating Mo-anode X-ray source, 3.185 < θ < 30.030°, 18604 measured reflections, 4147 independent reflections, 3989 reflections with $I > 2\sigma(I)$, R_{int} = 0.0822. The structure was solved by *SHELXT* and refined by full-matrix least-squares (*SHELXL*) against F^2 to R_1 = 0.0389 [$I > 2\sigma(I)$], wR_2 = 0.0701 [all data], 4147 parameters and 1 restraints.

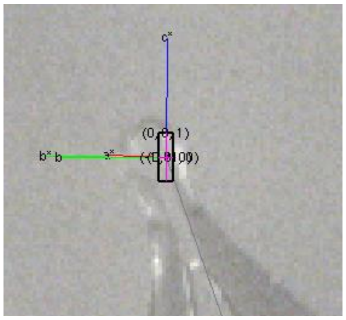

	h	k	l	distance (mm)
1.	-1	0	0	0.015
2.	1	0	0	0.015
3.	0	1	0	0.015
4.	0	-1	0	0.015
5.	0	0	1	0.05
6.	0	0	-1	0.05

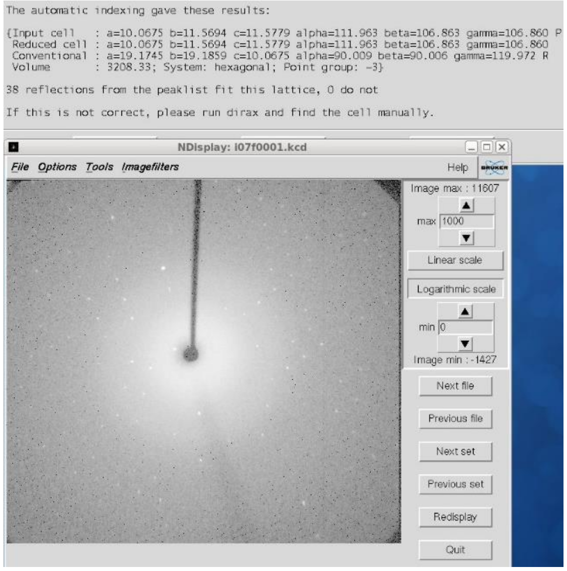

INTENSITY STATISTICS FOR DATASET

Resolution	#Data	#Theory	%Complete	Redundancy	Mean I	Mean I/s	Rmerge	Rsigma
Inf - 2.63	84	88	95.5	8.20	186.30	46.45	0.0487	0.0205
2.63 - 1.77	191	192	99.5	6.48	121.35	34.98	0.0519	0.0281
1.77 - 1.40	262	262	100.0	5.90	100.06	30.44	0.0540	0.0319
1.40 - 1.22	292	292	100.0	5.48	66.26	24.02	0.0638	0.0395
1.22 - 1.11	256	256	100.0	5.19	63.56	22.04	0.0667	0.0435
1.11 - 1.03	276	276	100.0	5.01	57.21	19.95	0.0745	0.0488
1.03 - 0.97	270	270	100.0	4.71	46.26	16.80	0.0839	0.0585
0.97 - 0.92	266	266	100.0	4.53	40.93	14.72	0.0969	0.0669
0.92 - 0.88	292	292	100.0	4.26	36.50	12.73	0.1092	0.0775
0.88 - 0.85	238	238	100.0	4.08	31.98	11.20	0.1226	0.0900
0.85 - 0.82	268	268	100.0	3.86	27.09	9.60	0.1436	0.1069
0.82 - 0.79	316	316	100.0	3.74	24.97	8.62	0.1587	0.1214
0.79 - 0.77	212	214	99.1	3.59	22.89	7.70	0.1803	0.1375
0.77 - 0.75	285	286	99.7	3.43	20.70	6.63	0.2059	0.1590
0.75 - 0.73	312	314	99.4	3.38	20.35	6.01	0.2280	0.1773
0.73 - 0.71	327	332	98.5	3.20	17.48	4.86	0.2574	0.2193
0.71 - 0.70	168	178	94.4	2.97	17.54	4.53	0.2809	0.2443
0.70 - 0.68	366	374	97.9	3.11	15.07	3.73	0.3017	0.2940
0.68 - 0.67	223	226	98.7	2.97	13.80	3.02	0.3265	0.3762
0.67 - 0.66	231	240	96.2	2.88	12.27	2.51	0.3769	0.4671
0.66 - 0.65	241	254	94.9	2.79	12.33	2.40	0.3745	0.4651
0.75 - 0.65	1868	1918	97.4	3.07	15.74	3.97	0.2908	0.2919
Inf - 0.65	5376	5434	98.9	4.12	39.70	12.61	0.0934	0.0888

A resolution cut off (SHEL 99 0.71) was applied to the data set to exclude poorly determined intensities at higher diffraction angles. The residual electron density map inidicates the hydride H atom attached to the Ir metal centre. This is confirmed by characteristic hydride IR absorption bands and HRMS. Complete .cif-data of the compound are available under the CCDC number **CCDC- 2174988**.

Crystal data and structure refinement.

Identification code	**14340**	
Empirical formula	C_{48} H_{37} Ir O_{12} P_4	
Color	pale yellow	
Formula weight	1121.85 g·mol^{-1}	
Temperature	100(2) K	
Wavelength	0.71073 Å	
Crystal system	trigonal	
Space group	*R*3, (no. 146)	
Unit cell dimensions	a = 19.1442(13) Å	α= 90°.
	b = 19.1442(13) Å	β= 90°.
	c = 10.0582(14) Å	γ = 120°.
Volume	3192.5(6) Å^3	
Z	3	
Density (calculated)	1.751 Mg·m^{-3}	
Absorption coefficient	3.355 mm^{-1}	
F(000)	1674 e	
Crystal size	0.10 x 0.03 x 0.03 mm^3	
θ range for data collection	3.185 to 30.030°.	
Index ranges	-26 ≤ h ≤ 26, -26≤ k ≤ 26, -13 ≤ l ≤ 14	
Reflections collected	18604	
Independent reflections	4147 [R_{int} = 0.0822]	
Reflections with I>2σ(I)	3989	
Completeness to θ = 25.242°	99.8 %	
Absorption correction	Gaussian	
Max. and min. transmission	0.99665 and 0.98964	
Refinement method	Full-matrix least-squares on F^2	
Data / restraints / parameters	4147 / 1 / 197	
Goodness-of-fit on F^2	1.071	
Final R indices [I>2σ(I)]	R_1 = 0.0389	wR^2 = 0.0701
R indices (all data)	R_1 = 0.0441	wR^2 = 0.0726
Absolute structure parameter	0.002(6)	
Extinction coefficient	n/a	
Largest diff. peak and hole	0.840 and -2.765 e·Å^{-3}	

Bond lengths [Å] and angles [°]

Ir(1)-H	1.70(13)	Ir(1)-P(1)	2.299(3)
Ir(1)-P(2)#1	2.2609(17)	Ir(1)-P(2)	2.2609(17)
Ir(1)-P(2)#2	2.2608(17)	P(1)-C(1)#2	1.819(7)
P(1)-C(1)	1.819(7)	P(1)-C(1)#1	1.819(7)
P(2)-C(5)	1.812(7)	P(2)-C(9)	1.812(7)
P(2)-C(13)	1.831(8)	O(1)-C(1)	1.371(8)
O(1)-C(4)	1.370(9)	O(2)-C(5)	1.367(9)
O(2)-C(8)	1.376(9)	O(3)-C(9)	1.369(8)
O(3)-C(12)	1.364(8)	O(4)-C(13)	1.374(8)
O(4)-C(16)	1.360(9)	C(1)-C(2)	1.353(9)
C(2)-H(2)	0.9500	C(2)-C(3)	1.434(10)
C(3)-H(3)	0.9500	C(3)-C(4)	1.343(11)
C(4)-H(4)	0.9500	C(5)-C(6)	1.360(10)
C(6)-H(6)	0.9500	C(6)-C(7)	1.422(11)
C(7)-H(7)	0.9500	C(7)-C(8)	1.334(12)
C(8)-H(8)	0.9500	C(9)-C(10)	1.364(9)
C(10)-H(10)	0.9500	C(10)-C(11)	1.437(10)
C(11)-H(11)	0.9500	C(11)-C(12)	1.341(10)
C(12)-H(12)	0.9500	C(13)-C(14)	1.339(10)
C(14)-H(14)	0.9500	C(14)-C(15)	1.432(10)
C(15)-H(15)	0.9500	C(15)-C(16)	1.327(11)
C(16)-H(16)	0.9500		
P(1)-Ir(1)-H	180.00(4)	P(2)#2-Ir(1)-H	81.48(4)
P(2)-Ir(1)-H	81.48(5)	P(2)#1-Ir(1)-H	81.48(5)
P(2)-Ir(1)-P(1)	98.52(4)	P(2)#1-Ir(1)-P(1)	98.52(4)
P(2)#2-Ir(1)-P(1)	98.52(4)	P(2)#2-Ir(1)-P(2)#1	117.85(2)
P(2)-Ir(1)-P(2)#1	117.84(2)	P(2)#2-Ir(1)-P(2)	117.84(2)
C(1)#2-P(1)-Ir(1)	118.8(2)	C(1)-P(1)-Ir(1)	118.8(2)
C(1)#1-P(1)-Ir(1)	118.8(2)	C(1)#1-P(1)-C(1)	98.7(3)
C(1)#2-P(1)-C(1)	98.7(3)	C(1)#2-P(1)-C(1)#1	98.7(3)
C(5)-P(2)-Ir(1)	122.0(2)	C(5)-P(2)-C(13)	97.6(3)
C(9)-P(2)-Ir(1)	116.2(2)	C(9)-P(2)-C(5)	99.1(3)
C(9)-P(2)-C(13)	98.1(3)	C(13)-P(2)-Ir(1)	119.2(2)

C(4)-O(1)-C(1)	106.3(6)	C(5)-O(2)-C(8)	106.0(6)
C(12)-O(3)-C(9)	106.7(5)	C(16)-O(4)-C(13)	106.0(6)
O(1)-C(1)-P(1)	116.3(5)	C(2)-C(1)-P(1)	133.2(6)
C(2)-C(1)-O(1)	110.5(6)	C(1)-C(2)-H(2)	127.0
C(1)-C(2)-C(3)	105.9(7)	C(3)-C(2)-H(2)	127.0
C(2)-C(3)-H(3)	126.6	C(4)-C(3)-C(2)	106.8(6)
C(4)-C(3)-H(3)	126.6	O(1)-C(4)-H(4)	124.8
C(3)-C(4)-O(1)	110.4(7)	C(3)-C(4)-H(4)	124.8
O(2)-C(5)-P(2)	117.8(5)	C(6)-C(5)-P(2)	132.1(6)
C(6)-C(5)-O(2)	109.6(6)	C(5)-C(6)-H(6)	126.5
C(5)-C(6)-C(7)	107.0(7)	C(7)-C(6)-H(6)	126.5
C(6)-C(7)-H(7)	126.9	C(8)-C(7)-C(6)	106.2(7)
C(8)-C(7)-H(7)	126.9	O(2)-C(8)-H(8)	124.4
C(7)-C(8)-O(2)	111.2(7)	C(7)-C(8)-H(8)	124.4
O(3)-C(9)-P(2)	119.3(5)	C(10)-C(9)-P(2)	130.9(5)
C(10)-C(9)-O(3)	109.6(6)	C(9)-C(10)-H(10)	126.8
C(9)-C(10)-C(11)	106.4(6)	C(11)-C(10)-H(10)	126.8
C(10)-C(11)-H(11)	127.0	C(12)-C(11)-C(10)	106.0(6)
C(12)-C(11)-H(11)	127.0	O(3)-C(12)-H(12)	124.4
C(11)-C(12)-O(3)	111.2(6)	C(11)-C(12)-H(12)	124.4
O(4)-C(13)-P(2)	118.0(5)	C(14)-C(13)-P(2)	132.3(6)
C(14)-C(13)-O(4)	109.7(7)	C(13)-C(14)-H(14)	126.5
C(13)-C(14)-C(15)	107.0(7)	C(15)-C(14)-H(14)	126.5
C(14)-C(15)-H(15)	127.1	C(16)-C(15)-C(14)	105.8(6)
C(16)-C(15)-H(15)	127.1	O(4)-C(16)-H(16)	124.3
C(15)-C(16)-O(4)	111.5(7)	C(15)-C(16)-H(16)	124.3

Symmetry transformations used to generate equivalent atoms:
#1 -y+1,x-y,z #2 -x+y+1,-x+1,z

X-ray structure of oxidation product of **19**

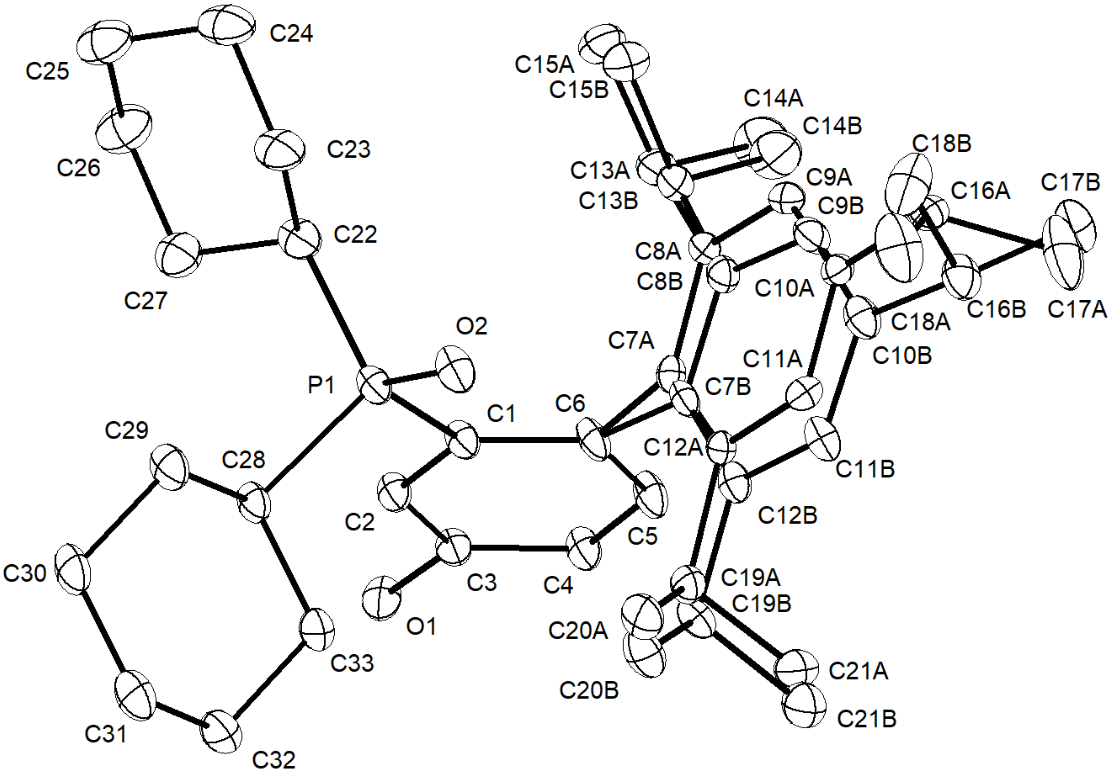

Molecular structure of oxidation product of **19**. H atoms have been removed for clarity.

X-ray Crystal Structure Analysis

$C_{33}H_{49}O_2P$, M_r = 508.69 g mol^{-1}, colorless block, crystal size 0.225 x 0.111 x 0.08 mm^3, monoclinic, space group $P2_1/c$ [14], a = 10.8991(7) Å, b = 18.0404(11) Å, c = 15.4367(9) Å, β = 104.441(2)°, V = 2939.3(3) Å^3, T = 100(2) K, Z = 4, D_{calc} = 1.150 g·cm^3, λ = 0.71073 Å, $\mu(Mo\text{-}K_\alpha)$ = 0.120 mm^{-1}, Gaussian absorption correction (T_{min} = 0.97893, T_{max} = 0.99136), Bruker-AXS Kappa Mach3 with APEX-II detector and IμS microfocus Mo-anode X-ray source, 1.769 < θ < 35.631°, 221491 measured reflections, 13565 independent reflections, 11273reflections with $I > 2\sigma(I)$, R_{int} = 0.0371. The structure was solved by *SHELXT* and refined by full-matrix least-squares (*SHELXL*) against F^2 to R_1 = 0.0365 [$I > 2\sigma(I)$], wR_2 = 0.1017 [all data], 473 parameters and 0 restraints.

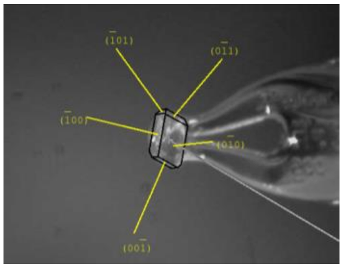

H	K	L	Distance [mm]
1	0	0	0.045
-1	0	0	0.055
0	1	-1	0.075
0	-1	1	0.080
0	1	0	0.040
0	-1	0	0.040
0	0	-1	0.090
0	0	1	0.100
-1	0	1	0.100
1	0	-1	0.090

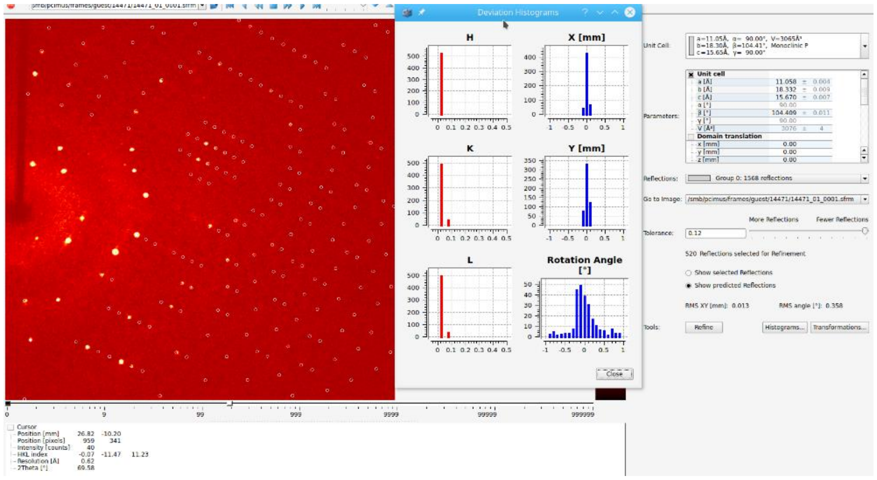

INTENSITY STATISTICS FOR DATASET

Resolution	#Data	#Theory	%Complete	Redundancy	Mean I	Mean I/s	Rmerge	Rsigma
Inf - 2.47	232	233	99.6	22.99	41.95	135.30	0.0202	0.0061
2.47 - 1.62	540	541	99.8	27.74	14.02	126.66	0.0208	0.0055
1.62 - 1.28	777	777	100.0	28.29	6.57	103.72	0.0250	0.0065
1.28 - 1.11	802	802	100.0	27.10	5.85	88.80	0.0288	0.0074
1.11 - 1.01	763	763	100.0	21.92	3.45	64.13	0.0377	0.0103
1.01 - 0.94	749	749	100.0	18.59	2.54	48.87	0.0468	0.0135
0.94 - 0.88	827	827	100.0	16.50	1.90	38.62	0.0553	0.0171
0.88 - 0.83	898	898	100.0	15.43	1.67	34.67	0.0630	0.0198
0.83 - 0.80	640	640	100.0	14.57	1.57	30.94	0.0701	0.0225
0.80 - 0.77	740	740	100.0	14.12	1.30	25.70	0.0833	0.0264
0.77 - 0.74	872	872	100.0	13.56	1.26	24.30	0.0885	0.0289
0.74 - 0.72	651	651	100.0	13.01	0.96	19.20	0.1051	0.0366
0.72 - 0.69	1157	1157	100.0	12.54	0.90	17.60	0.1176	0.0409
0.69 - 0.68	419	419	100.0	12.16	0.74	14.42	0.1426	0.0502
0.68 - 0.66	931	931	100.0	11.64	0.78	14.39	0.1393	0.0504
0.66 - 0.64	1040	1040	100.0	11.35	0.66	12.23	0.1638	0.0602
0.64 - 0.63	609	609	100.0	10.79	0.64	10.92	0.1790	0.0645
0.63 - 0.62	622	622	100.0	10.62	0.54	10.04	0.1962	0.0750
0.62 - 0.61	654	654	100.0	10.38	0.46	8.39	0.2405	0.0904
0.61 - 0.59	1515	1708	88.7	6.68	0.37	6.29	0.2665	0.1384
0.69 - 0.59	5790	5983	96.8	9.88	0.57	10.38	0.1806	0.0752
Inf - 0.59	15438	15633	98.8	15.09	2.77	35.83	0.0372	0.0157

A resolution cut off (SHEL 99 0.61) was applied to the data set to exclude poorly determined intensities at higher diffraction angles. The structure shows a positional disorder on the 2',4',6'-triisopropyl-[1,1'-biphenyl] unit over two positions with 0.5:0.5 occupancy. Complete .cif-data of the compound are available under the CCDC number **CCDC-2174989**.

Crystal data and structure refinement

Identification code	**14471**	
Empirical formula	$C_{33} H_{49} O_2 P$	
Color	colourless	
Formula weight	508.69 g·mol^{-1}	
Temperature	100(2) K	
Wavelength	0.71073 Å	
Crystal system	Monoclinic	
Space group	$P2_1/c$, (no. 14)	
Unit cell dimensions	a = 10.8991(7) Å	α= 90°.
	b = 18.0404(11) Å	β= 104.441(2)°.
	c = 15.4367(9) Å	γ = 90°.
Volume	2939.3(3) Å^3	
Z	4	
Density (calculated)	1.150 Mg·m^{-3}	
Absorption coefficient	0.120 mm^{-1}	
F(000)	1112 e	
Crystal size	0.225 x 0.111 x 0.08 mm^3	
θ range for data collection	1.769 to 35.631°.	
Index ranges	$-17 \le h \le 17$, $-29 \le k \le 29$, $-25 \le l \le 25$	
Reflections collected	221491	
Independent reflections	13565 [R_{int} = 0.0371]	
Reflections with I>2σ(I)	11273	
Completeness to θ = 25.242°	100.0 %	
Absorption correction	Gaussian	
Max. and min. transmission	0.99136 and 0.97893	
Refinement method	Full-matrix least-squares on F^2	
Data / restraints / parameters	13565 / 0 / 473	
Goodness-of-fit on F^2	1.081	
Final R indices [I>2σ(I)]	R_1 = 0.0365	wR^2 = 0.1017
R indices (all data)	R_1 = 0.0482	wR^2 = 0.1124
Extinction coefficient	n/a	
Largest diff. peak and hole	0.587 and -0.243 e·Å^{-3}	

Bond lengths [Å] and angles [°]

P(1)-O(2)	1.4946(6)	P(1)-C(1)	1.8131(6)
P(1)-C(22)	1.8334(8)	P(1)-C(28)	1.8251(6)
O(1)-H(1)	0.8400	O(1)-C(3)	1.3558(8)
C(1)-C(2)	1.4015(9)	C(1)-C(6)	1.4085(9)
C(2)-H(2)	0.9500	C(2)-C(3)	1.3903(8)
C(3)-C(4)	1.3963(9)	C(4)-H(4)	0.9500
C(4)-C(5)	1.3859(9)	C(5)-H(5)	0.9500
C(5)-C(6)	1.4013(9)	C(6)-C(7A)	1.471(5)
C(6)-C(7B)	1.554(5)	C(7A)-C(8A)	1.408(5)
C(7A)-C(12A)	1.401(4)	C(7B)-C(8B)	1.402(5)
C(7B)-C(12B)	1.400(4)	C(8A)-C(9A)	1.405(3)
C(8A)-C(13A)	1.505(3)	C(8B)-C(9B)	1.413(3)
C(8B)-C(13B)	1.509(3)	C(9A)-H(9A)	0.9500
C(9A)-C(10A)	1.390(2)	C(9B)-H(9B)	0.9500
C(9B)-C(10B)	1.388(3)	C(10A)-C(11A)	1.380(2)
C(10A)-C(16A)	1.5222(17)	C(10B)-C(11B)	1.373(2)
C(10B)-C(16B)	1.529(2)	C(11A)-H(11A)	0.9500
C(11A)-C(12A)	1.403(3)	C(11B)-H(11B)	0.9500
C(11B)-C(12B)	1.402(3)	C(12A)-C(19A)	1.513(4)
C(12B)-C(19B)	1.510(4)	C(13A)-H(13A)	1.0000
C(13A)-C(14A)	1.540(6)	C(13A)-C(15A)	1.530(3)
C(13B)-H(13B)	1.0000	C(13B)-C(14B)	1.527(6)
C(13B)-C(15B)	1.532(3)	C(14A)-H(14A)	0.9800
C(14A)-H(14B)	0.9800	C(14A)-H(14C)	0.9800
C(14B)-H(14D)	0.9800	C(14B)-H(14E)	0.9800
C(14B)-H(14F)	0.9800	C(15A)-H(15A)	0.9800
C(15A)-H(15B)	0.9800	C(15A)-H(15C)	0.9800
C(15B)-H(15D)	0.9800	C(15B)-H(15E)	0.9800
C(15B)-H(15F)	0.9800	C(16A)-H(16A)	1.0000
C(16A)-C(17A)	1.531(6)	C(16A)-C(18A)	1.539(2)
C(16B)-H(16B)	1.0000	C(16B)-C(17B)	1.506(4)
C(16B)-C(18B)	1.515(2)	C(17A)-H(17A)	0.9800
C(17A)-H(17B)	0.9800	C(17A)-H(17C)	0.9800
C(17B)-H(17D)	0.9800	C(17B)-H(17E)	0.9800

C(17B)-H(17F)	0.9800	C(18A)-H(18A)	0.9800
C(18A)-H(18B)	0.9800	C(18A)-H(18C)	0.9800
C(18B)-H(18D)	0.9800	C(18B)-H(18E)	0.9800
C(18B)-H(18F)	0.9800	C(19A)-H(19A)	1.0000
C(19A)-C(20A)	1.521(6)	C(19A)-C(21A)	1.523(4)
C(19B)-H(19B)	1.0000	C(19B)-C(20B)	1.536(5)
C(19B)-C(21B)	1.520(4)	C(20A)-H(20A)	0.9800
C(20A)-H(20B)	0.9800	C(20A)-H(20C)	0.9800
C(20B)-H(20D)	0.9800	C(20B)-H(20E)	0.9800
C(20B)-H(20F)	0.9800	C(21A)-H(21A)	0.9800
C(21A)-H(21B)	0.9800	C(21A)-H(21C)	0.9800
C(21B)-H(21D)	0.9800	C(21B)-H(21E)	0.9800
C(21B)-H(21F)	0.9800	C(22)-H(22)	1.0000
C(22)-C(23)	1.5445(10)	C(22)-C(27)	1.5368(11)
C(23)-H(23A)	0.9900	C(23)-H(23B)	0.9900
C(23)-C(24)	1.5273(12)	C(24)-H(24A)	0.9900
C(24)-H(24B)	0.9900	C(24)-C(25)	1.5199(15)
C(25)-H(25A)	0.9900	C(25)-H(25B)	0.9900
C(25)-C(26)	1.5298(12)	C(26)-H(26A)	0.9900
C(26)-H(26B)	0.9900	C(26)-C(27)	1.5292(12)
C(27)-H(27A)	0.9900	C(27)-H(27B)	0.9900
C(28)-H(28)	1.0000	C(28)-C(29)	1.5395(9)
C(28)-C(33)	1.5366(10)	C(29)-H(29A)	0.9900
C(29)-H(29B)	0.9900	C(29)-C(30)	1.5297(10)
C(30)-H(30A)	0.9900	C(30)-H(30B)	0.9900
C(30)-C(31)	1.5236(12)	C(31)-H(31A)	0.9900
C(31)-H(31B)	0.9900	C(31)-C(32)	1.5260(12)
C(32)-H(32A)	0.9900	C(32)-H(32B)	0.9900
C(32)-C(33)	1.5284(10)	C(33)-H(33A)	0.9900
C(33)-H(33B)	0.9900		

O(2)-P(1)-C(1)	111.91(3)	O(2)-P(1)-C(22)	112.42(3)
O(2)-P(1)-C(28)	110.96(3)	C(1)-P(1)-C(22)	106.48(3)
C(1)-P(1)-C(28)	105.46(3)	C(28)-P(1)-C(22)	109.27(3)
C(3)-O(1)-H(1)	109.5	C(2)-C(1)-P(1)	117.25(5)
C(2)-C(1)-C(6)	119.59(5)	C(6)-C(1)-P(1)	123.04(5)

C(1)-C(2)-H(2)	119.2	C(3)-C(2)-C(1)	121.64(6)
C(3)-C(2)-H(2)	119.2	O(1)-C(3)-C(2)	118.49(6)
O(1)-C(3)-C(4)	122.37(5)	C(2)-C(3)-C(4)	119.14(6)
C(3)-C(4)-H(4)	120.4	C(5)-C(4)-C(3)	119.21(6)
C(5)-C(4)-H(4)	120.4	C(4)-C(5)-H(5)	118.6
C(4)-C(5)-C(6)	122.80(6)	C(6)-C(5)-H(5)	118.6
C(1)-C(6)-C(7A)	123.00(18)	C(1)-C(6)-C(7B)	127.31(18)
C(5)-C(6)-C(1)	117.59(6)	C(5)-C(6)-C(7A)	119.15(19)
C(5)-C(6)-C(7B)	114.65(17)	C(8A)-C(7A)-C(6)	123.4(3)
C(12A)-C(7A)-C(6)	116.1(3)	C(12A)-C(7A)-C(8A)	120.2(4)
C(8B)-C(7B)-C(6)	116.6(2)	C(12B)-C(7B)-C(6)	122.9(3)
C(12B)-C(7B)-C(8B)	120.3(4)	C(7A)-C(8A)-C(13A)	121.6(3)
C(9A)-C(8A)-C(7A)	118.2(2)	C(9A)-C(8A)-C(13A)	120.3(2)
C(7B)-C(8B)-C(9B)	118.3(2)	C(7B)-C(8B)-C(13B)	121.7(2)
C(9B)-C(8B)-C(13B)	120.0(2)	C(8A)-C(9A)-H(9A)	118.8
C(10A)-C(9A)-C(8A)	122.38(15)	C(10A)-C(9A)-H(9A)	118.8
C(8B)-C(9B)-H(9B)	119.0	C(10B)-C(9B)-C(8B)	122.00(16)
C(10B)-C(9B)-H(9B)	119.0	C(9A)-C(10A)-C(16A)	120.67(13)
C(11A)-C(10A)-C(9A)	118.18(13)	C(11A)-C(10A)-C(16A)	121.14(14)
C(9B)-C(10B)-C(16B)	122.14(14)	C(11B)-C(10B)-C(9B)	118.21(14)
C(11B)-C(10B)-C(16B)	119.64(16)	C(10A)-C(11A)-H(11A)	119.1
C(10A)-C(11A)-C(12A)	121.86(16)	C(12A)-C(11A)-H(11A)	119.1
C(10B)-C(11B)-H(11B)	118.9	C(10B)-C(11B)-C(12B)	122.24(18)
C(12B)-C(11B)-H(11B)	118.9	C(7A)-C(12A)-C(11A)	119.2(3)
C(7A)-C(12A)-C(19A)	121.5(3)	C(11A)-C(12A)-C(19A)	119.3(2)
C(7B)-C(12B)-C(11B)	119.0(3)	C(7B)-C(12B)-C(19B)	122.5(3)
C(11B)-C(12B)-C(19B)	118.5(2)	C(8A)-C(13A)-H(13A)	107.8
C(8A)-C(13A)-C(14A)	110.3(2)	C(8A)-C(13A)-C(15A)	112.4(2)
C(14A)-C(13A)-H(13A)	107.8	C(15A)-C(13A)-H(13A)	107.8
C(15A)-C(13A)-C(14A)	110.5(3)	C(8B)-C(13B)-H(13B)	107.3
C(8B)-C(13B)-C(14B)	111.1(2)	C(8B)-C(13B)-C(15B)	112.6(2)
C(14B)-C(13B)-H(13B)	107.3	C(14B)-C(13B)-C(15B)	110.9(3)
C(15B)-C(13B)-H(13B)	107.3	C(13A)-C(14A)-H(14A)	109.5
C(13A)-C(14A)-H(14B)	109.5	C(13A)-C(14A)-H(14C)	109.5
H(14A)-C(14A)-H(14B)	109.5	H(14A)-C(14A)-H(14C)	109.5
H(14B)-C(14A)-H(14C)	109.5	C(13B)-C(14B)-H(14D)	109.5

C(13B)-C(14B)-H(14E)	109.5	C(13B)-C(14B)-H(14F)	109.5
H(14D)-C(14B)-H(14E)	109.5	H(14D)-C(14B)-H(14F)	109.5
H(14E)-C(14B)-H(14F)	109.5	C(13A)-C(15A)-H(15A)	109.5
C(13A)-C(15A)-H(15B)	109.5	C(13A)-C(15A)-H(15C)	109.5
H(15A)-C(15A)-H(15B)	109.5	H(15A)-C(15A)-H(15C)	109.5
H(15B)-C(15A)-H(15C)	109.5	C(13B)-C(15B)-H(15D)	109.5
C(13B)-C(15B)-H(15E)	109.5	C(13B)-C(15B)-H(15F)	109.5
H(15D)-C(15B)-H(15E)	109.5	H(15D)-C(15B)-H(15F)	109.5
H(15E)-C(15B)-H(15F)	109.5	C(10A)-C(16A)-H(16A)	108.1
C(10A)-C(16A)-C(17A)	110.7(2)	C(10A)-C(16A)-C(18A)	112.91(13)
C(17A)-C(16A)-H(16A)	108.1	C(17A)-C(16A)-C(18A)	108.9(2)
C(18A)-C(16A)-H(16A)	108.1	C(10B)-C(16B)-H(16B)	107.3
C(17B)-C(16B)-C(10B)	111.1(2)	C(17B)-C(16B)-H(16B)	107.3
C(17B)-C(16B)-C(18B)	112.0(2)	C(18B)-C(16B)-C(10B)	111.58(13)
C(18B)-C(16B)-H(16B)	107.3	C(16A)-C(17A)-H(17A)	109.5
C(16A)-C(17A)-H(17B)	109.5	C(16A)-C(17A)-H(17C)	109.5
H(17A)-C(17A)-H(17B)	109.5	H(17A)-C(17A)-H(17C)	109.5
H(17B)-C(17A)-H(17C)	109.5	C(16B)-C(17B)-H(17D)	109.5
C(16B)-C(17B)-H(17E)	109.5	C(16B)-C(17B)-H(17F)	109.5
H(17D)-C(17B)-H(17E)	109.5	H(17D)-C(17B)-H(17F)	109.5
H(17E)-C(17B)-H(17F)	109.5	C(16A)-C(18A)-H(18A)	109.5
C(16A)-C(18A)-H(18B)	109.5	C(16A)-C(18A)-H(18C)	109.5
H(18A)-C(18A)-H(18B)	109.5	H(18A)-C(18A)-H(18C)	109.5
H(18B)-C(18A)-H(18C)	109.5	C(16B)-C(18B)-H(18D)	109.5
C(16B)-C(18B)-H(18E)	109.5	C(16B)-C(18B)-H(18F)	109.5
H(18D)-C(18B)-H(18E)	109.5	H(18D)-C(18B)-H(18F)	109.5
H(18E)-C(18B)-H(18F)	109.5	C(12A)-C(19A)-H(19A)	107.3
C(12A)-C(19A)-C(20A)	112.1(2)	C(12A)-C(19A)-C(21A)	111.7(3)
C(20A)-C(19A)-H(19A)	107.3	C(20A)-C(19A)-C(21A)	110.9(3)
C(21A)-C(19A)-H(19A)	107.3	C(12B)-C(19B)-H(19B)	107.8
C(12B)-C(19B)-C(20B)	111.3(2)	C(12B)-C(19B)-C(21B)	112.0(3)
C(20B)-C(19B)-H(19B)	107.8	C(21B)-C(19B)-H(19B)	107.8
C(21B)-C(19B)-C(20B)	109.9(3)	C(19A)-C(20A)-H(20A)	109.5
C(19A)-C(20A)-H(20B)	109.5	C(19A)-C(20A)-H(20C)	109.5
H(20A)-C(20A)-H(20B)	109.5	H(20A)-C(20A)-H(20C)	109.5
H(20B)-C(20A)-H(20C)	109.5	C(19B)-C(20B)-H(20D)	109.5

C(19B)-C(20B)-H(20E)	109.5	C(19B)-C(20B)-H(20F)	109.5
H(20D)-C(20B)-H(20E)	109.5	H(20D)-C(20B)-H(20F)	109.5
H(20E)-C(20B)-H(20F)	109.5	C(19A)-C(21A)-H(21A)	109.5
C(19A)-C(21A)-H(21B)	109.5	C(19A)-C(21A)-H(21C)	109.5
H(21A)-C(21A)-H(21B)	109.5	H(21A)-C(21A)-H(21C)	109.5
H(21B)-C(21A)-H(21C)	109.5	C(19B)-C(21B)-H(21D)	109.5
C(19B)-C(21B)-H(21E)	109.5	C(19B)-C(21B)-H(21F)	109.5
H(21D)-C(21B)-H(21E)	109.5	H(21D)-C(21B)-H(21F)	109.5
H(21E)-C(21B)-H(21F)	109.5	P(1)-C(22)-H(22)	106.1
C(23)-C(22)-P(1)	111.66(5)	C(23)-C(22)-H(22)	106.1
C(27)-C(22)-P(1)	114.65(5)	C(27)-C(22)-H(22)	106.1
C(27)-C(22)-C(23)	111.42(6)	C(22)-C(23)-H(23A)	109.6
C(22)-C(23)-H(23B)	109.6	H(23A)-C(23)-H(23B)	108.1
C(24)-C(23)-C(22)	110.37(7)	C(24)-C(23)-H(23A)	109.6
C(24)-C(23)-H(23B)	109.6	C(23)-C(24)-H(24A)	109.3
C(23)-C(24)-H(24B)	109.3	H(24A)-C(24)-H(24B)	107.9
C(25)-C(24)-C(23)	111.67(7)	C(25)-C(24)-H(24A)	109.3
C(25)-C(24)-H(24B)	109.3	C(24)-C(25)-H(25A)	109.5
C(24)-C(25)-H(25B)	109.5	C(24)-C(25)-C(26)	110.52(7)
H(25A)-C(25)-H(25B)	108.1	C(26)-C(25)-H(25A)	109.5
C(26)-C(25)-H(25B)	109.5	C(25)-C(26)-H(26A)	109.3
C(25)-C(26)-H(26B)	109.3	H(26A)-C(26)-H(26B)	108.0
C(27)-C(26)-C(25)	111.63(7)	C(27)-C(26)-H(26A)	109.3
C(27)-C(26)-H(26B)	109.3	C(22)-C(27)-H(27A)	109.3
C(22)-C(27)-H(27B)	109.3	C(26)-C(27)-C(22)	111.57(7)
C(26)-C(27)-H(27A)	109.3	C(26)-C(27)-H(27B)	109.3
H(27A)-C(27)-H(27B)	108.0	P(1)-C(28)-H(28)	108.6
C(29)-C(28)-P(1)	111.50(4)	C(29)-C(28)-H(28)	108.6
C(33)-C(28)-P(1)	109.21(4)	C(33)-C(28)-H(28)	108.6
C(33)-C(28)-C(29)	110.40(6)	C(28)-C(29)-H(29A)	109.5
C(28)-C(29)-H(29B)	109.5	H(29A)-C(29)-H(29B)	108.1
C(30)-C(29)-C(28)	110.67(6)	C(30)-C(29)-H(29A)	109.5
C(30)-C(29)-H(29B)	109.5	C(29)-C(30)-H(30A)	109.4
C(29)-C(30)-H(30B)	109.4	H(30A)-C(30)-H(30B)	108.0
C(31)-C(30)-C(29)	111.20(6)	C(31)-C(30)-H(30A)	109.4
C(31)-C(30)-H(30B)	109.4	C(30)-C(31)-H(31A)	109.4

C(30)-C(31)-H(31B)	109.4	C(30)-C(31)-C(32)	111.30(7)
H(31A)-C(31)-H(31B)	108.0	C(32)-C(31)-H(31A)	109.4
C(32)-C(31)-H(31B)	109.4	C(31)-C(32)-H(32A)	109.3
C(31)-C(32)-H(32B)	109.3	C(31)-C(32)-C(33)	111.48(7)
H(32A)-C(32)-H(32B)	108.0	C(33)-C(32)-H(32A)	109.3
C(33)-C(32)-H(32B)	109.3	C(28)-C(33)-H(33A)	109.3
C(28)-C(33)-H(33B)	109.3	C(32)-C(33)-C(28)	111.71(6)
C(32)-C(33)-H(33A)	109.3	C(32)-C(33)-H(33B)	109.3
H(33A)-C(33)-H(33B)	107.9		

NMR spectra of isolated compounds

^{1}H NMR of dicyclohexyl(2',4',6'-triisopropyl-4-(4,4,5,5-tetramethyl-1,3,2-dioxaborolan-2-yl)-[1,1'-biphenyl]-2-yl)phosphane (Borylated XPhos, 1)

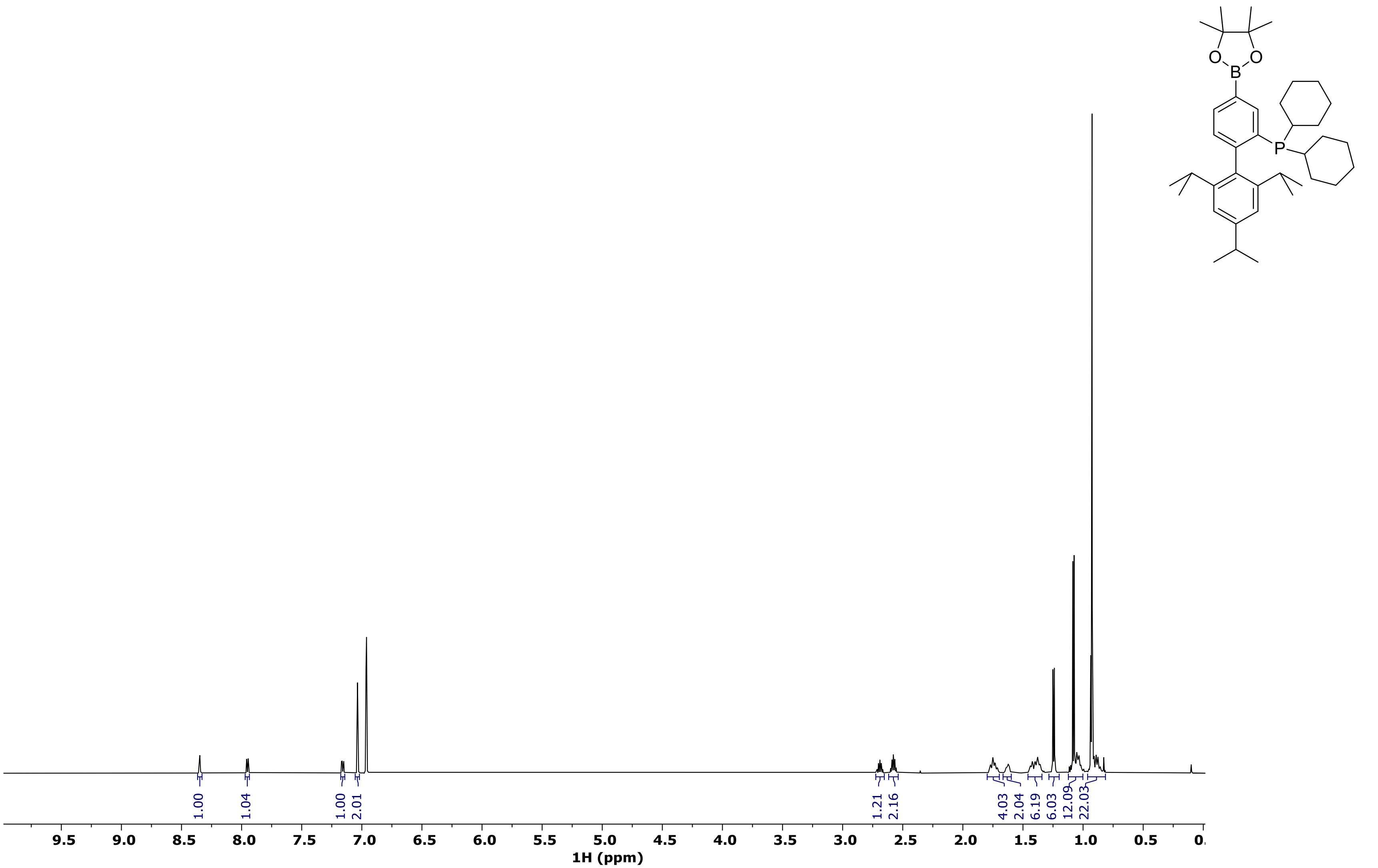

^{13}C NMR of dicyclohexyl(2',4',6'-triisopropyl-4-(4,4,5,5-tetramethyl-1,3,2-dioxaborolan-2-yl)-[1,1'-biphenyl]-2-yl)phosphane (Borylated XPhos, 1)

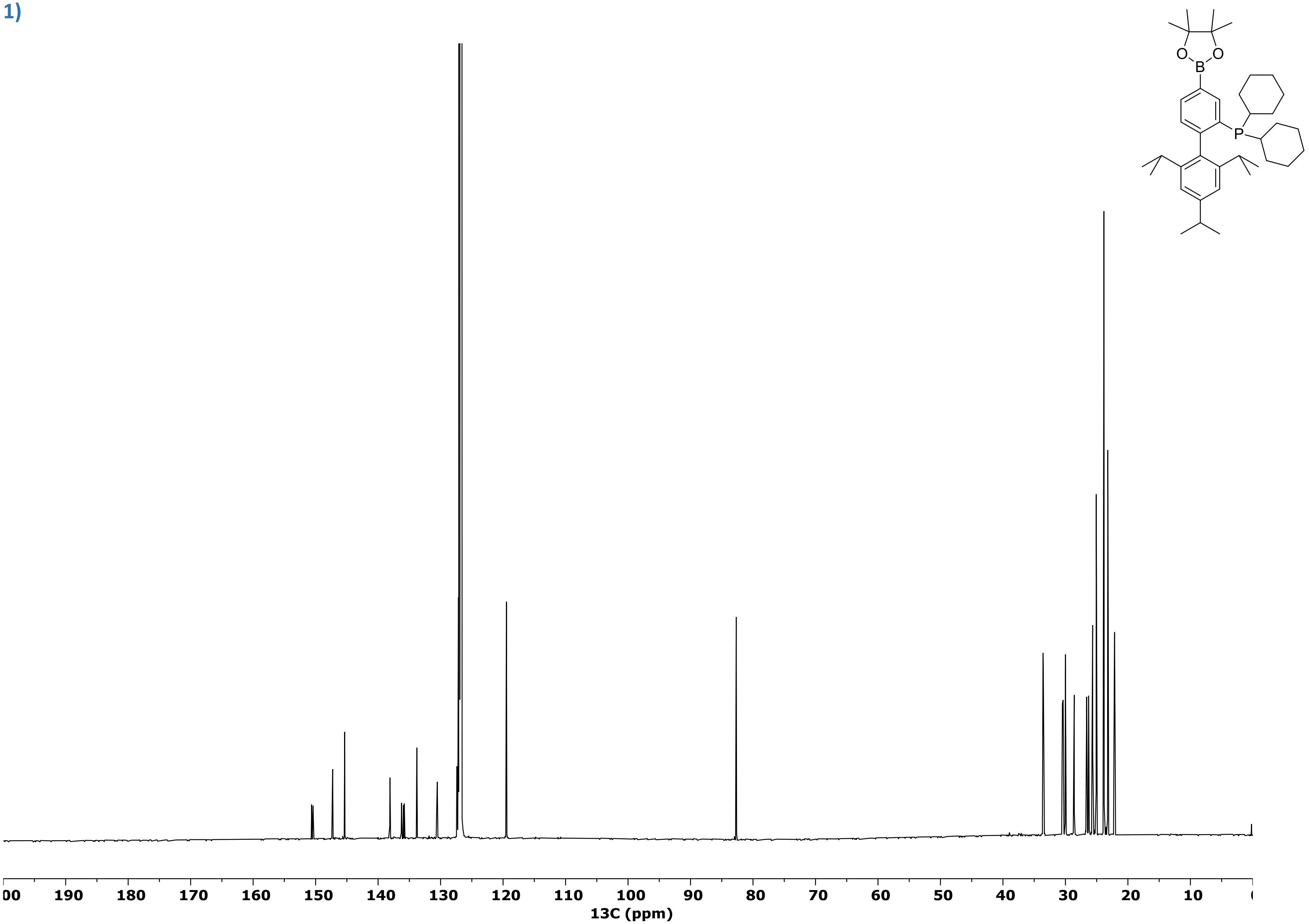

^{31}P NMR of dicyclohexyl(2',4',6'-triisopropyl-4-(4,4,5,5-tetramethyl-1,3,2-dioxaborolan-2-yl)-[1,1'-biphenyl]-2-yl)phosphane (Borylated XPhos, 1)

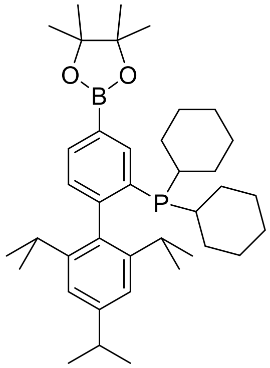

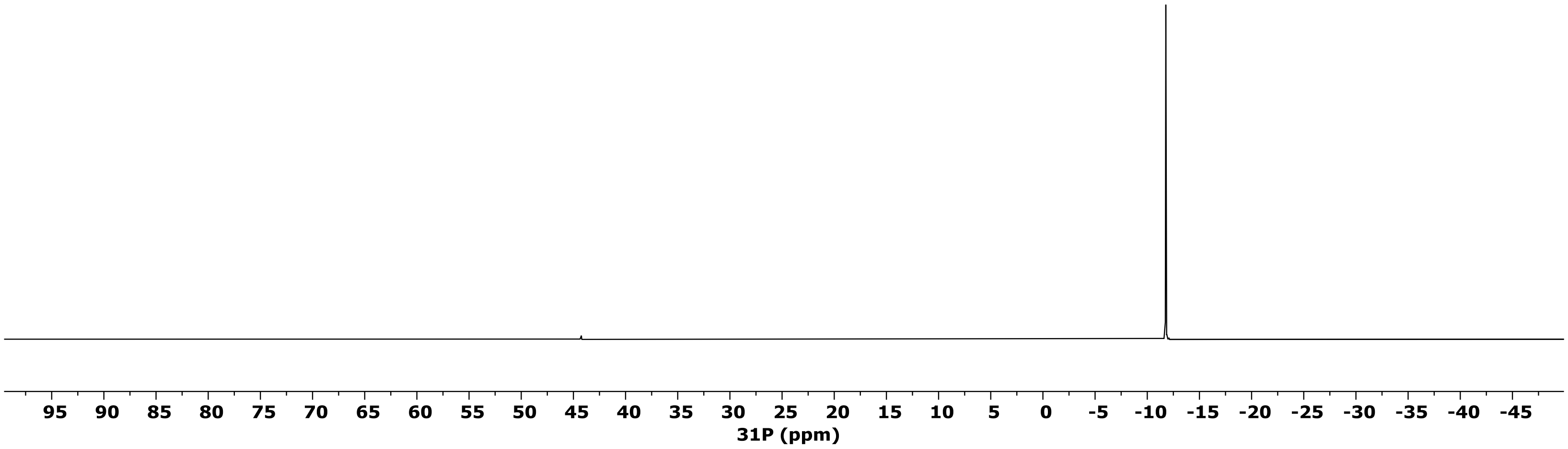

^{1}H NMR of dicyclohexyl(2',6'-diisopropoxy-4,4'-bis(4,4,5,5-tetramethyl-1,3,2-dioxaborolan-2-yl)-[1,1'-biphenyl]-2-yl)phosphane (Borylated RuPhos, 2)

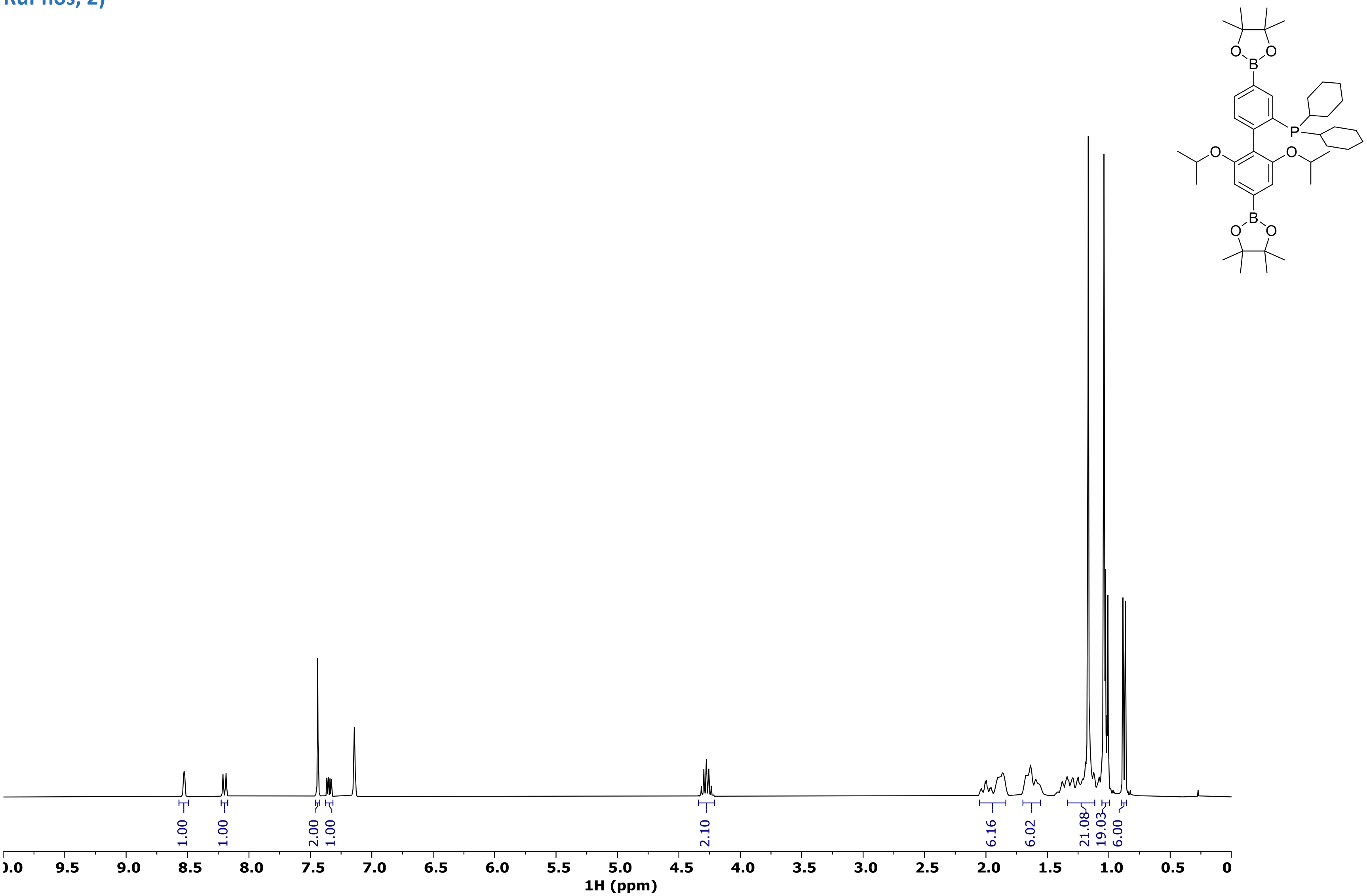

^{13}C NMR of dicyclohexyl(2',6'-diisopropoxy-4,4'-bis(4,4,5,5-tetramethyl-1,3,2-dioxaborolan-2-yl)-[1,1'-biphenyl]-2-yl)phosphane (Borylated RuPhos, 2)

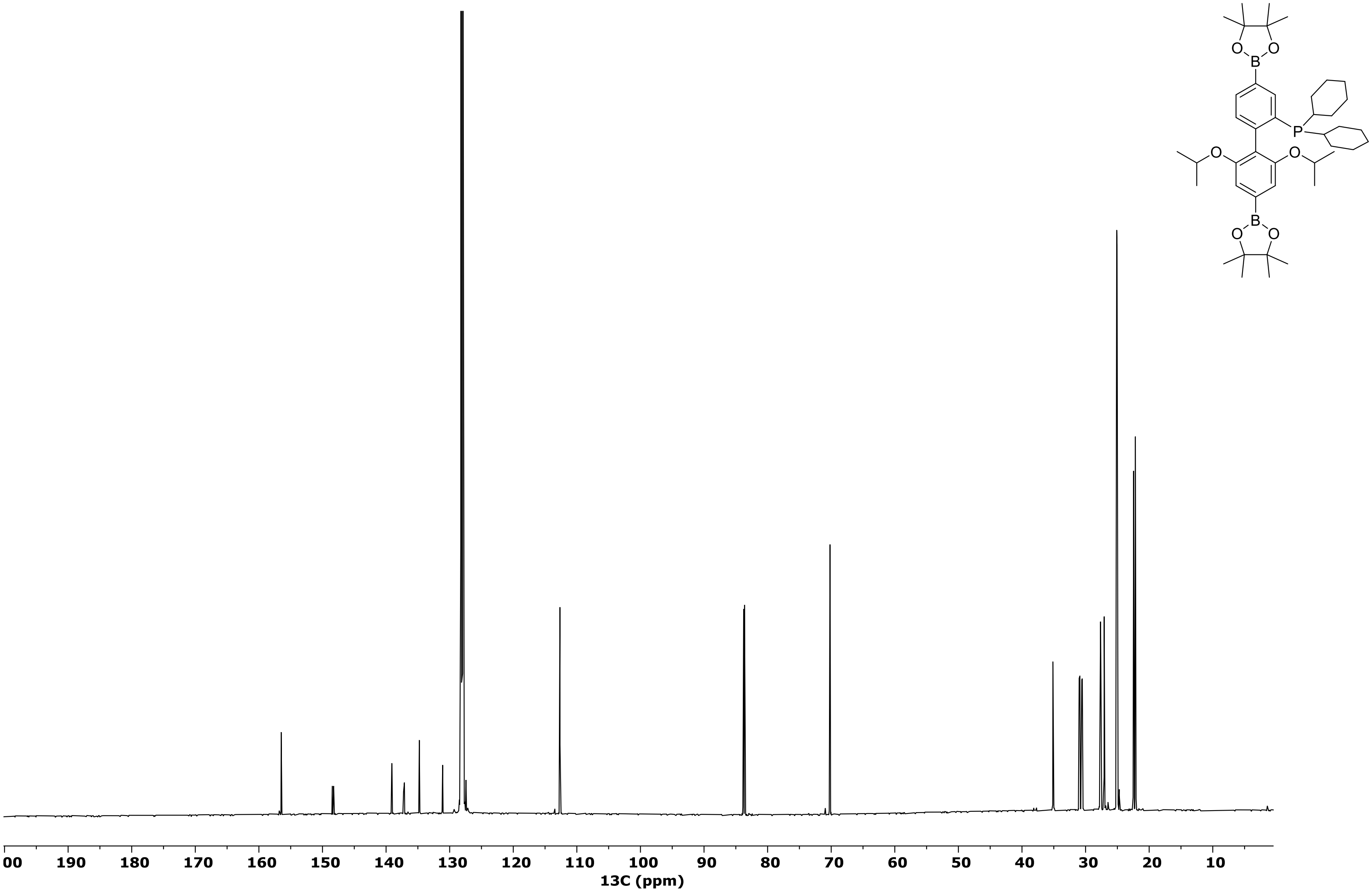

^{31}P NMR of dicyclohexyl(2',6'-diisopropoxy-4,4'-bis(4,4,5,5-tetramethyl-1,3,2-dioxaborolan-2-yl)-[1,1'-biphenyl]-2-yl)phosphane (Borylated RuPhos, 2)

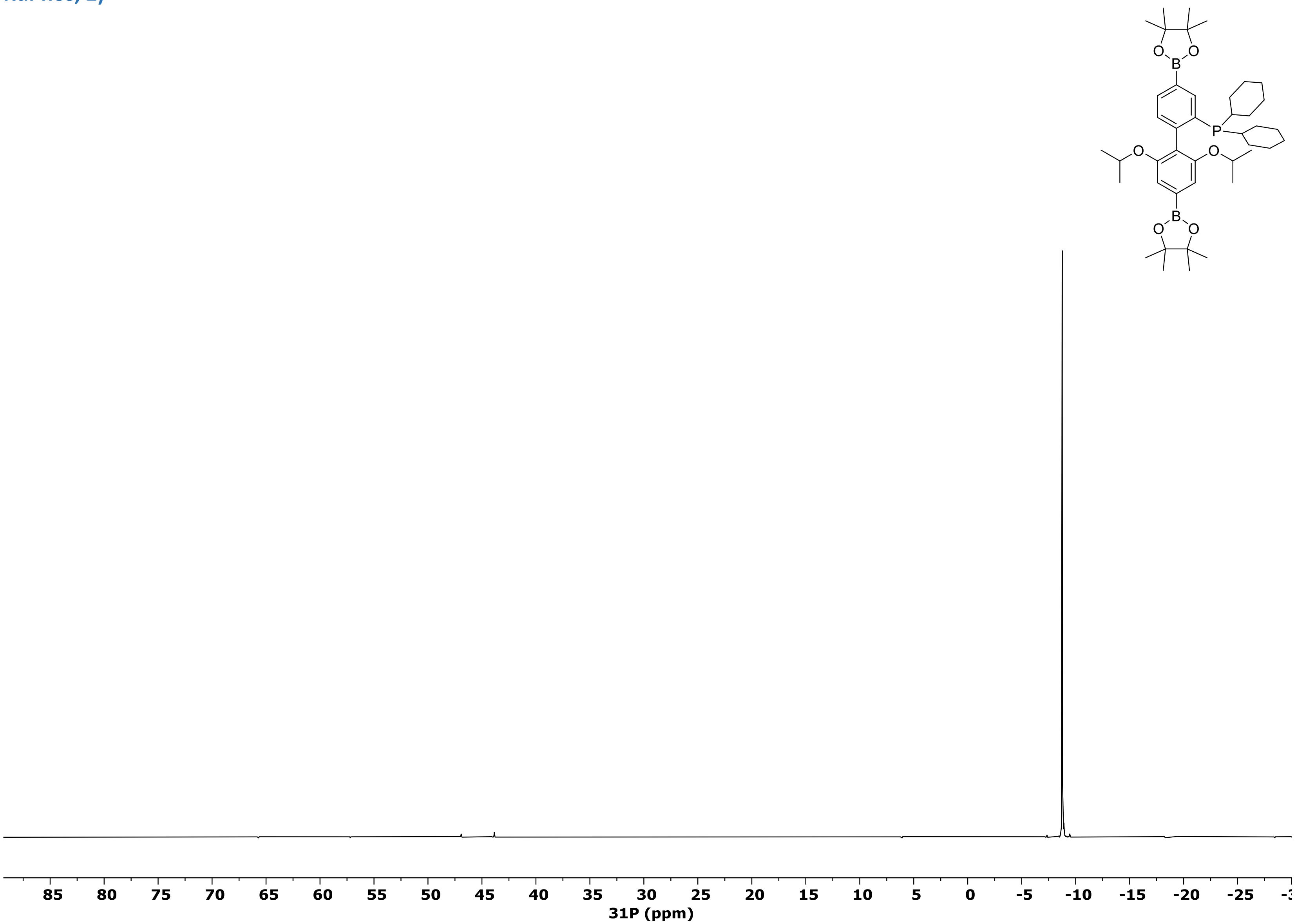

[^{31}P, ^{1}H]-HMBC of dicyclohexyl(2',6'-diisopropoxy-4,4'-bis(4,4,5,5-tetramethyl-1,3,2-dioxaborolan-2-yl)-[1,1'-biphenyl]-2-yl)phosphane (Borylated RuPhos, 2)

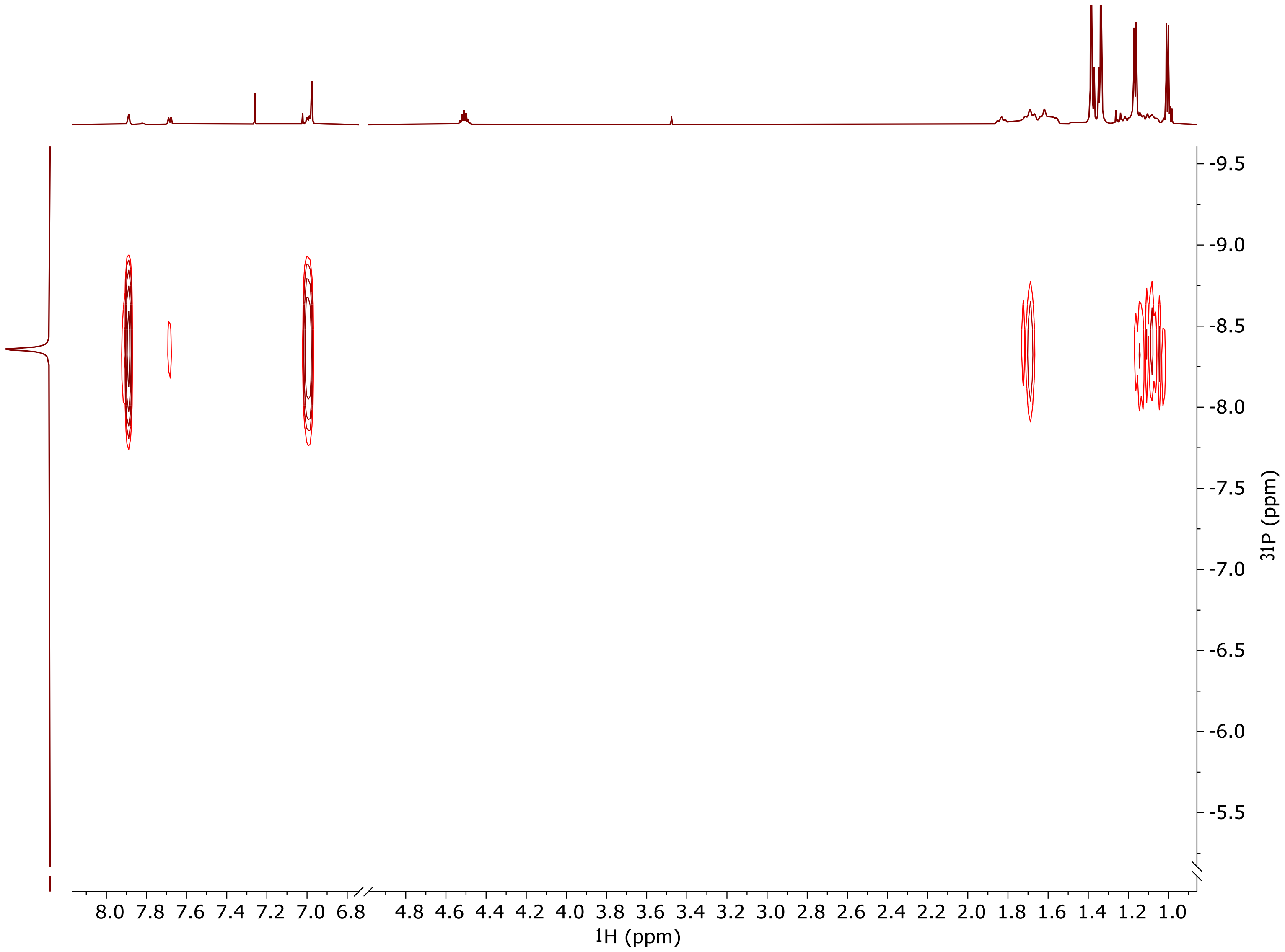

NOESY of dicyclohexyl(2',6'-diisopropoxy-4,4'-bis(4,4,5,5-tetramethyl-1,3,2-dioxaborolan-2-yl)-[1,1'-biphenyl]-2-yl)phosphane (Borylated RuPhos, 2)

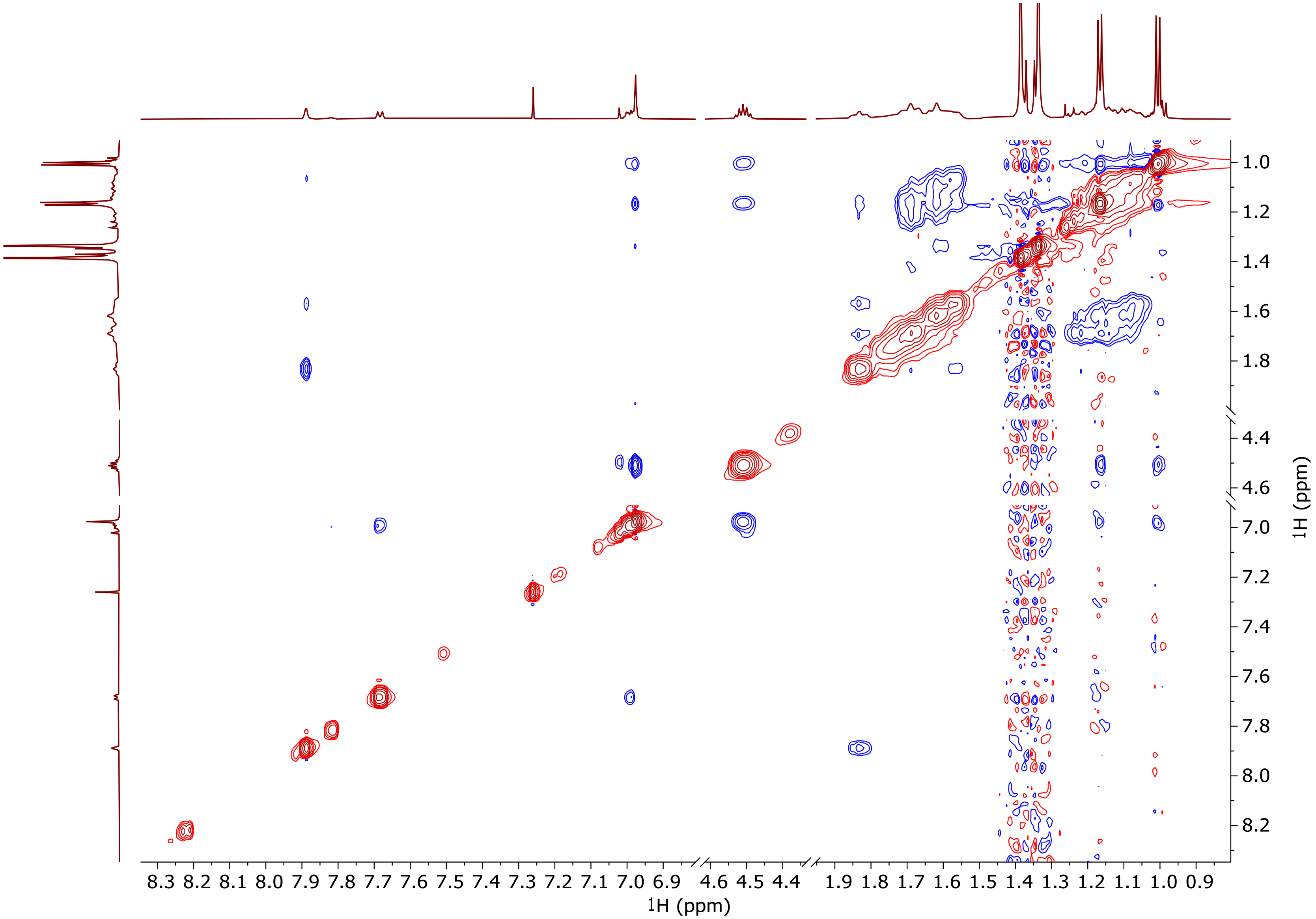

^{1}H NMR of (3-(tert-butoxy)-2',6'-diisopropyl-6-methoxy-4'-(4,4,5,5-tetramethyl-1,3,2-dioxaborolan-2-yl)-[1,1'-biphenyl]-2-yl)dicyclohexylphosphane (Borylated GPhos, 3)

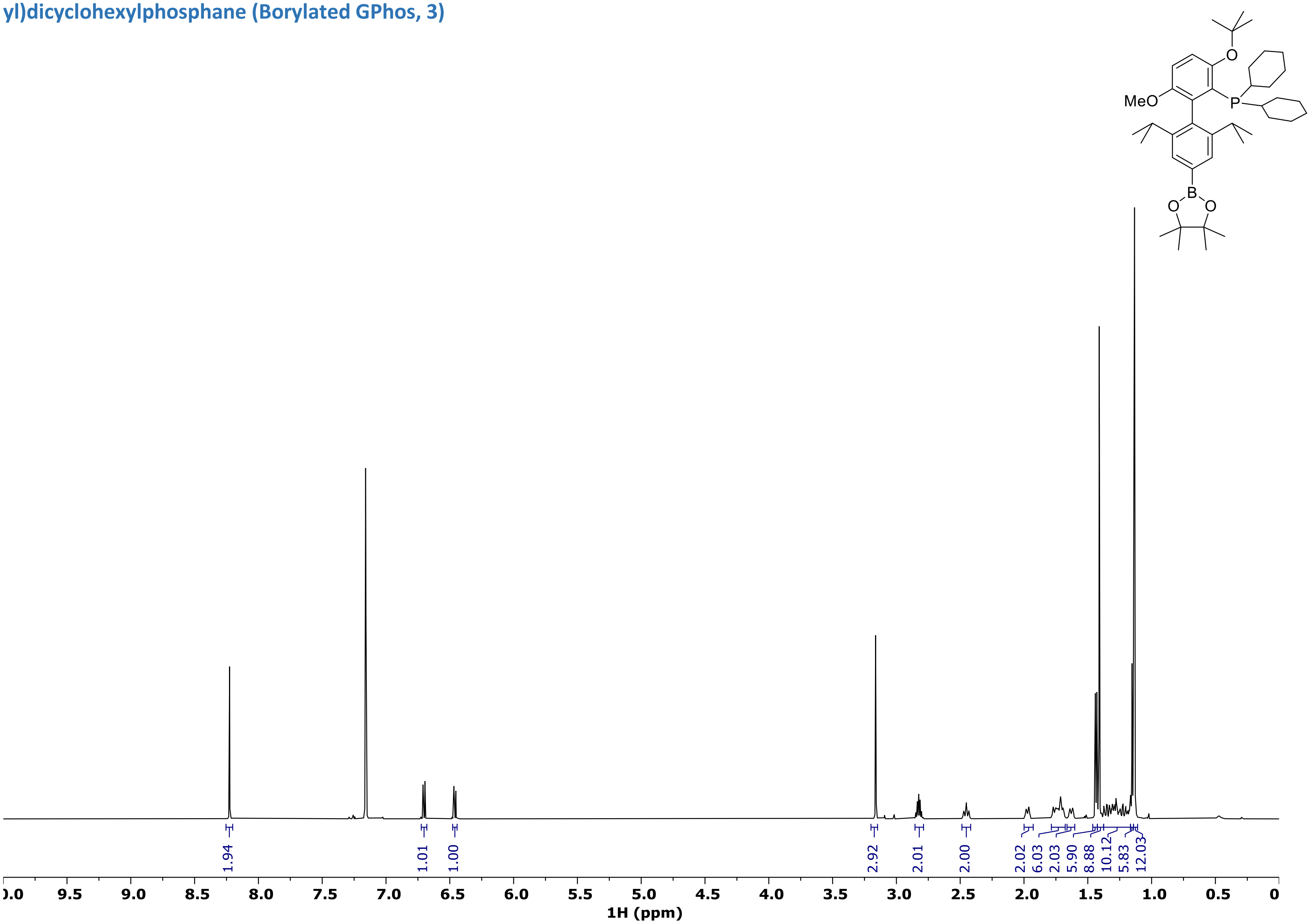

^{13}C NMR of (3-(tert-butoxy)-2',6'-diisopropyl-6-methoxy-4'-(4,4,5,5-tetramethyl-1,3,2-dioxaborolan-2-yl)-[1,1'-biphenyl]-2-yl)dicyclohexylphosphane (Borylated GPhos, 3)

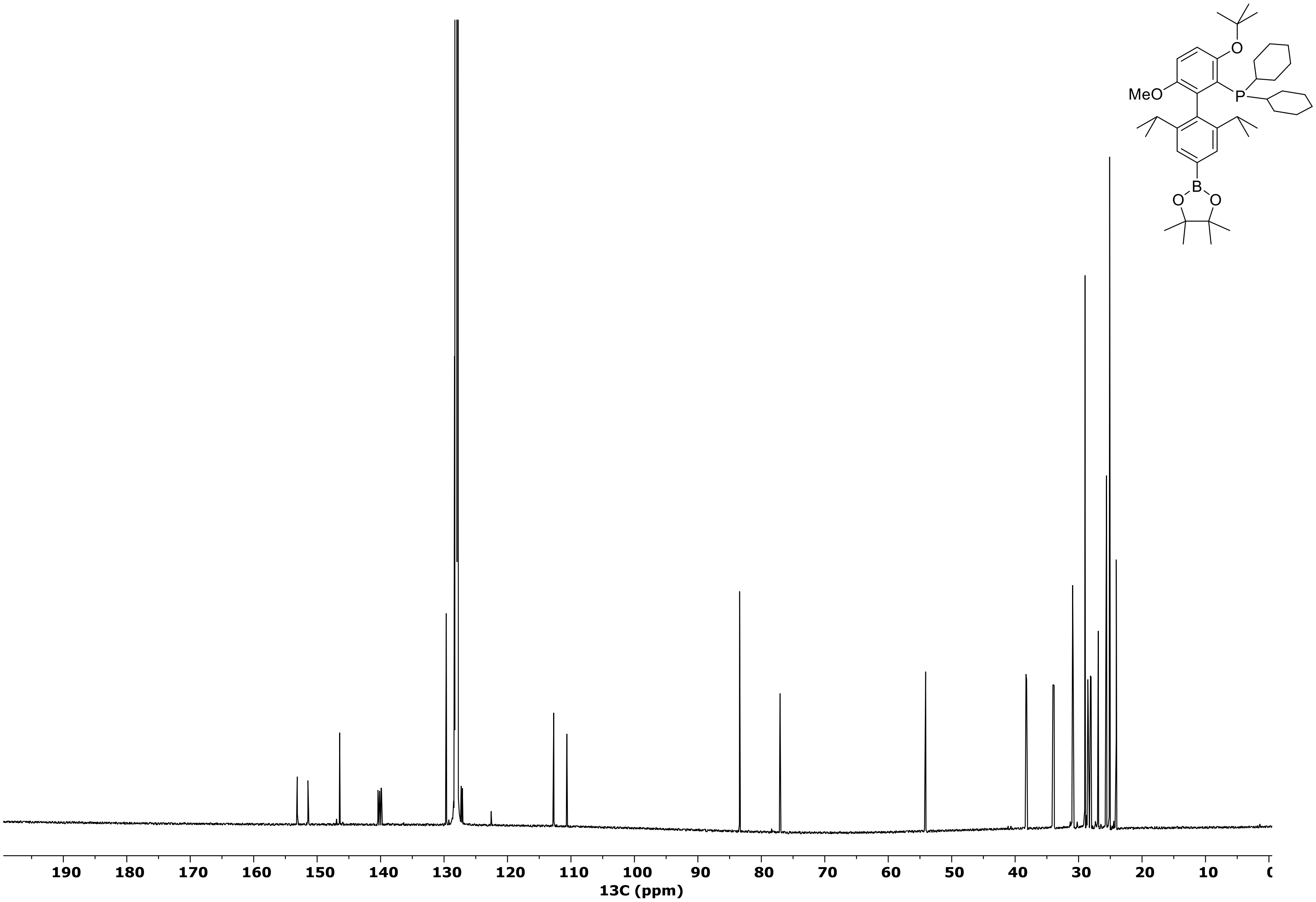

^{31}P NMR of (3-(tert-butoxy)-2',6'-diisopropyl-6-methoxy-4'-(4,4,5,5-tetramethyl-1,3,2-dioxaborolan-2-yl)-[1,1'-biphenyl]-2-yl)dicyclohexylphosphane (Borylated GPhos, 3)

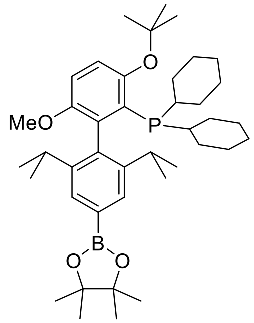

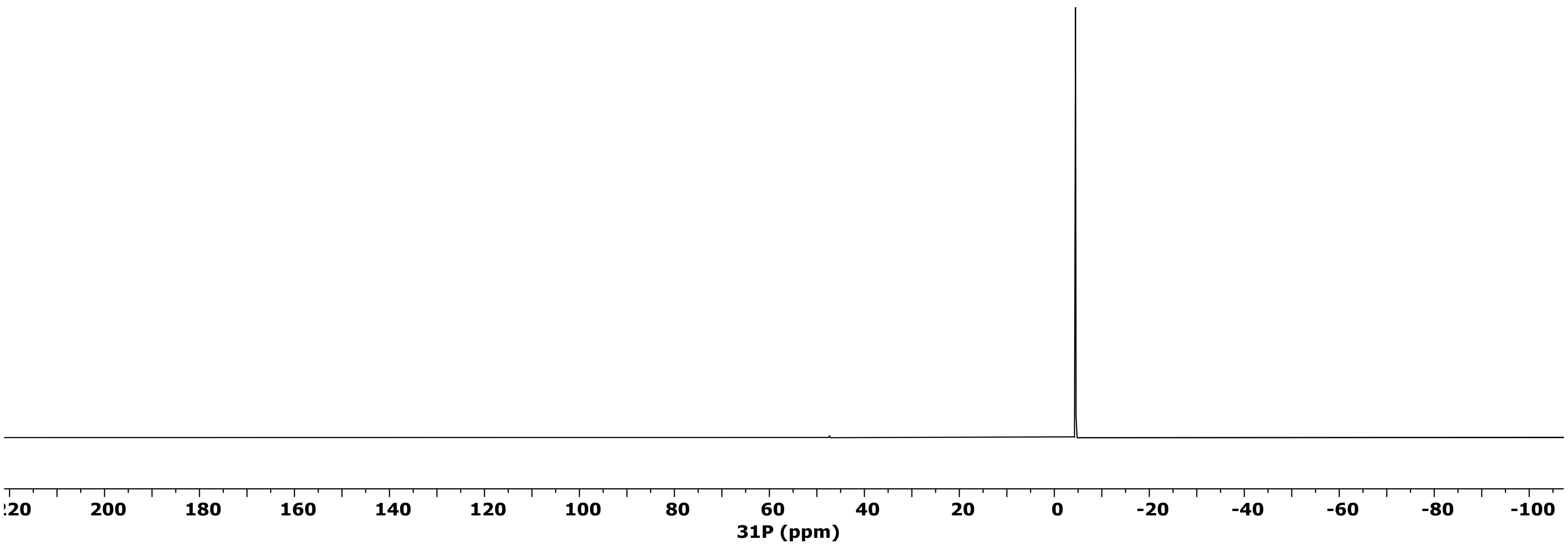

^{1}H NMR of 4-(2-(((1r,3R,5S)-adamantan-1-yl)(adamantan-1-yl)phosphaneyl)-5-(4,4,5,5-tetramethyl-1,3,2-dioxaborolan-2-yl)phenyl)morpholine (Borylated MorDalPhos, 4)

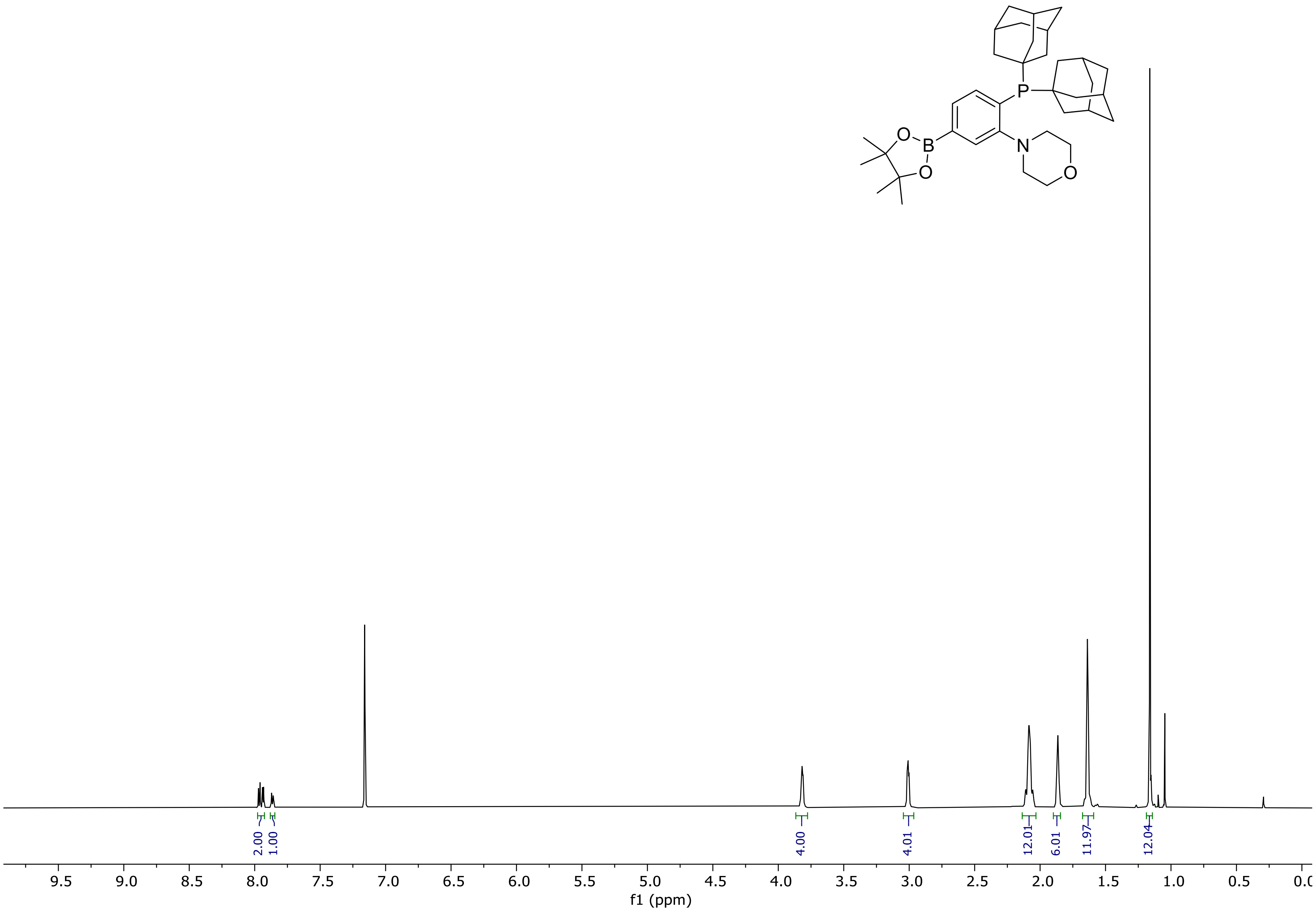

^{13}C NMR of 4-(2-(((1r,3R,5S)-adamantan-1-yl)(adamantan-1-yl)phosphaneyl)-5-(4,4,5,5-tetramethyl-1,3,2-dioxaborolan-2-yl)phenyl)morpholine (Borylated MorDalPhos, 4)

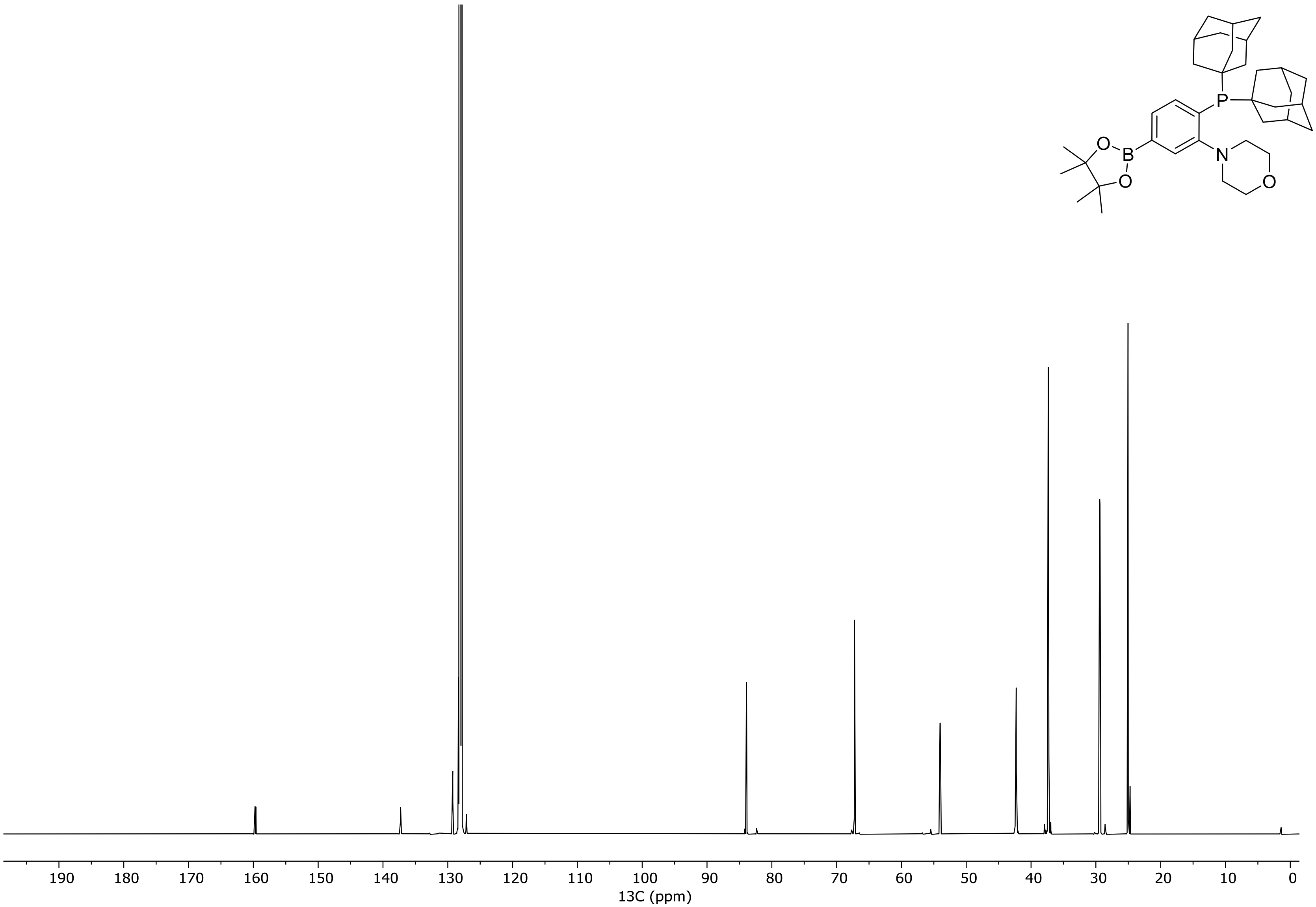

^{31}P NMR of 4-(2-(((1r,3R,5S)-adamantan-1-yl)(adamantan-1-yl)phosphaneyl)-5-(4,4,5,5-tetramethyl-1,3,2-dioxaborolan-2-yl)phenyl)morpholine (Borylated MorDalPhos, 4)

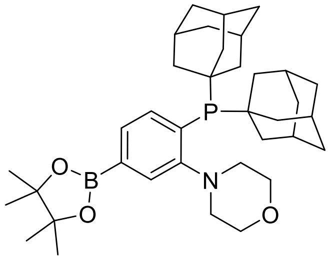

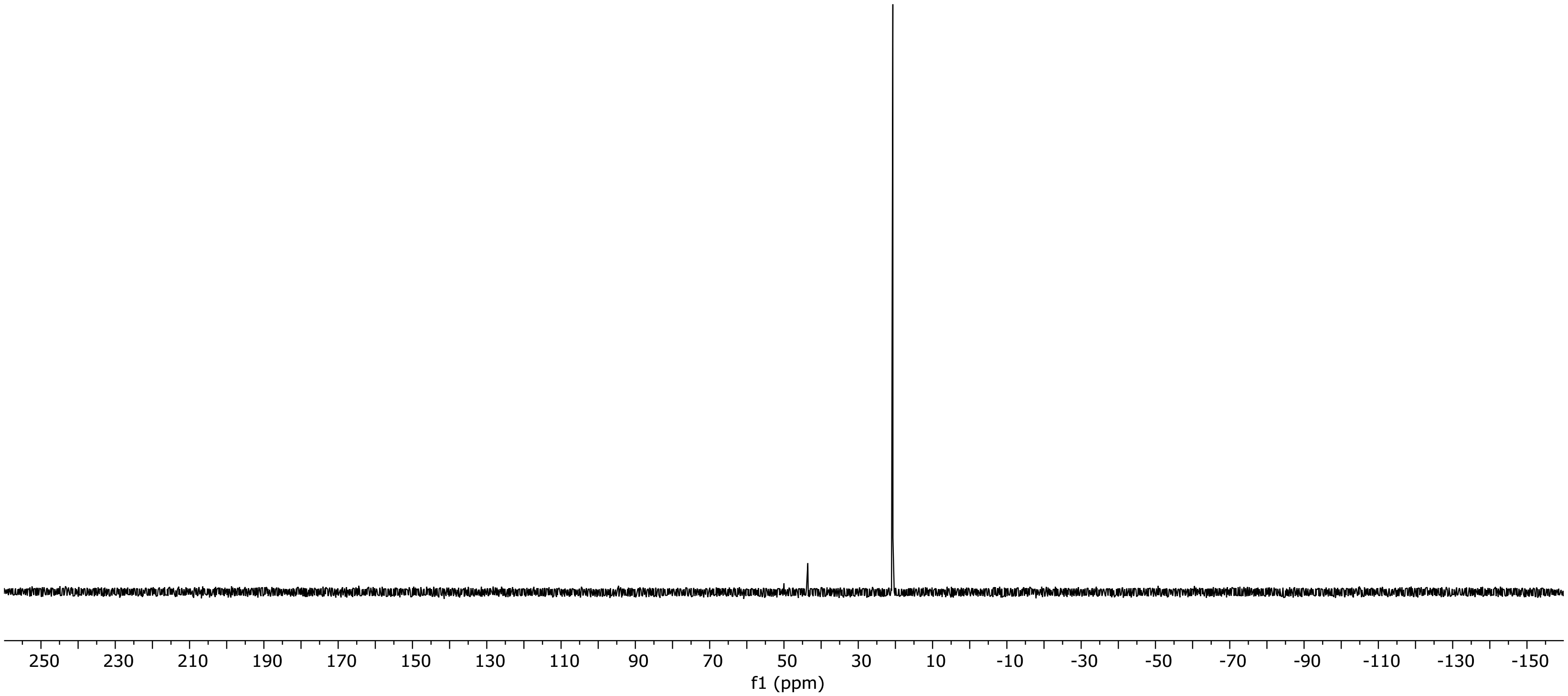

[^{31}P, ^{1}H]-HMBC of 4-(2-(((1r,3R,5S)-adamantan-1-yl)(adamantan-1-yl)phosphaneyl)-5-(4,4,5,5-tetramethyl-1,3,2-dioxaborolan-2-yl)phenyl)morpholine (Borylated MorDalPhos, 4)

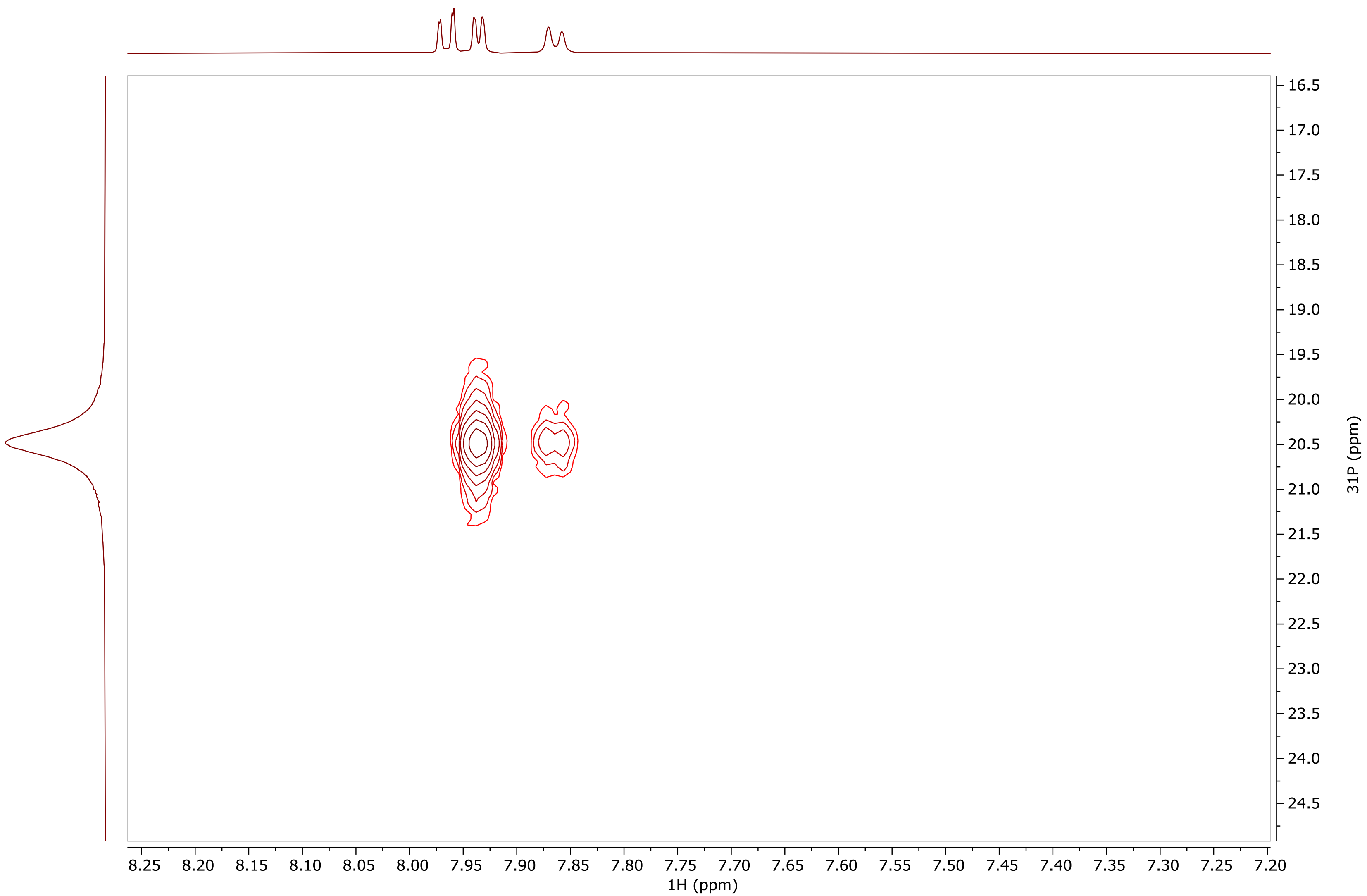

[^{15}N, ^{1}H]-HMBC of 4-(2-(((1r,3R,5S)-adamantan-1-yl)(adamantan-1-yl)phosphaneyl)-5-(4,4,5,5-tetramethyl-1,3,2-dioxaborolan-2-yl)phenyl)morpholine (Borylated MorDalPhos, 4)

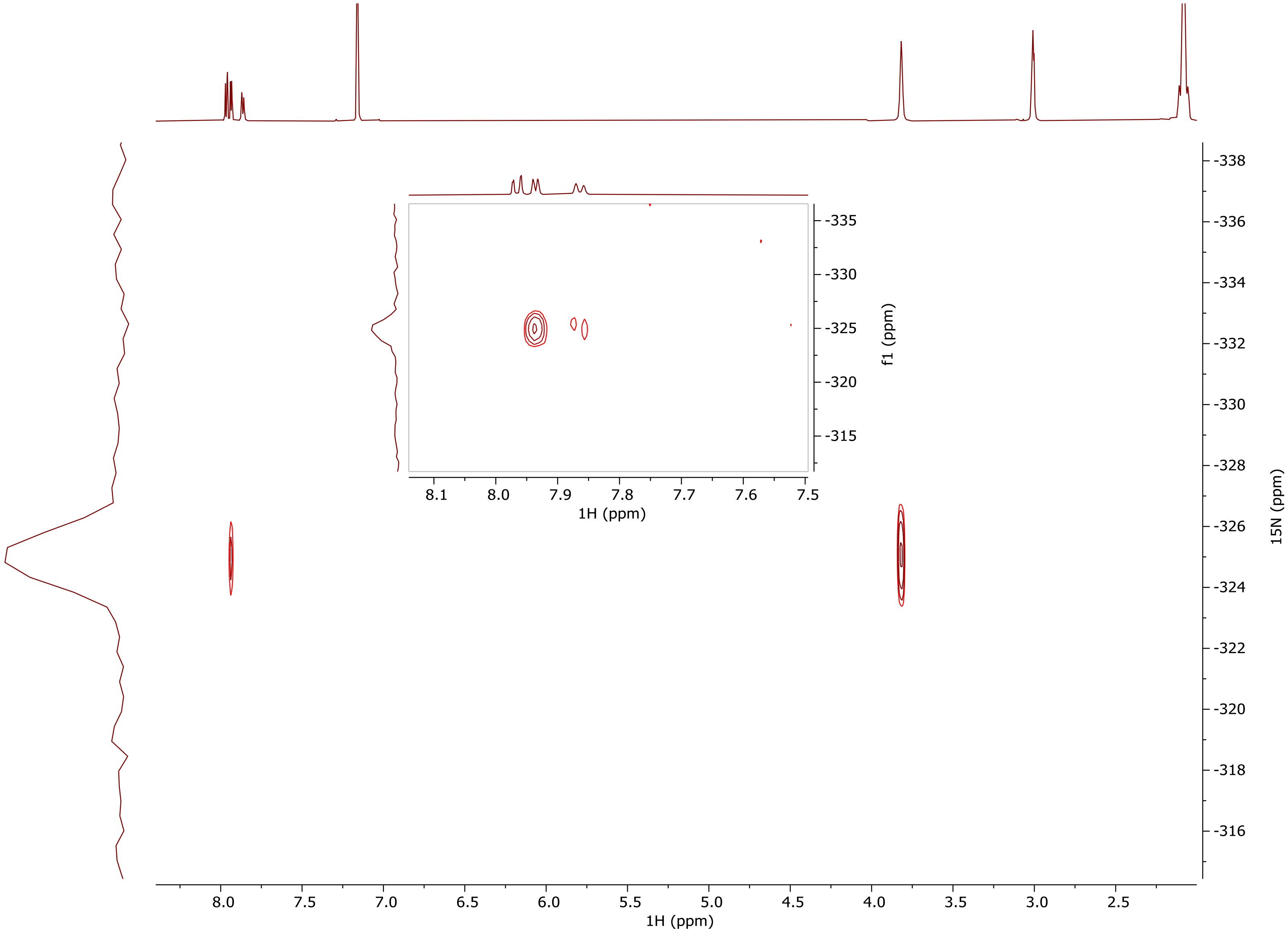

NOESY NMR of 4-(2-(((1r,3R,5S)-adamantan-1-yl)(adamantan-1-yl)phosphaneyl)-5-(4,4,5,5-tetramethyl-1,3,2-dioxaborolan-2-yl)phenyl)morpholine (Borylated MorDalPhos, 4)

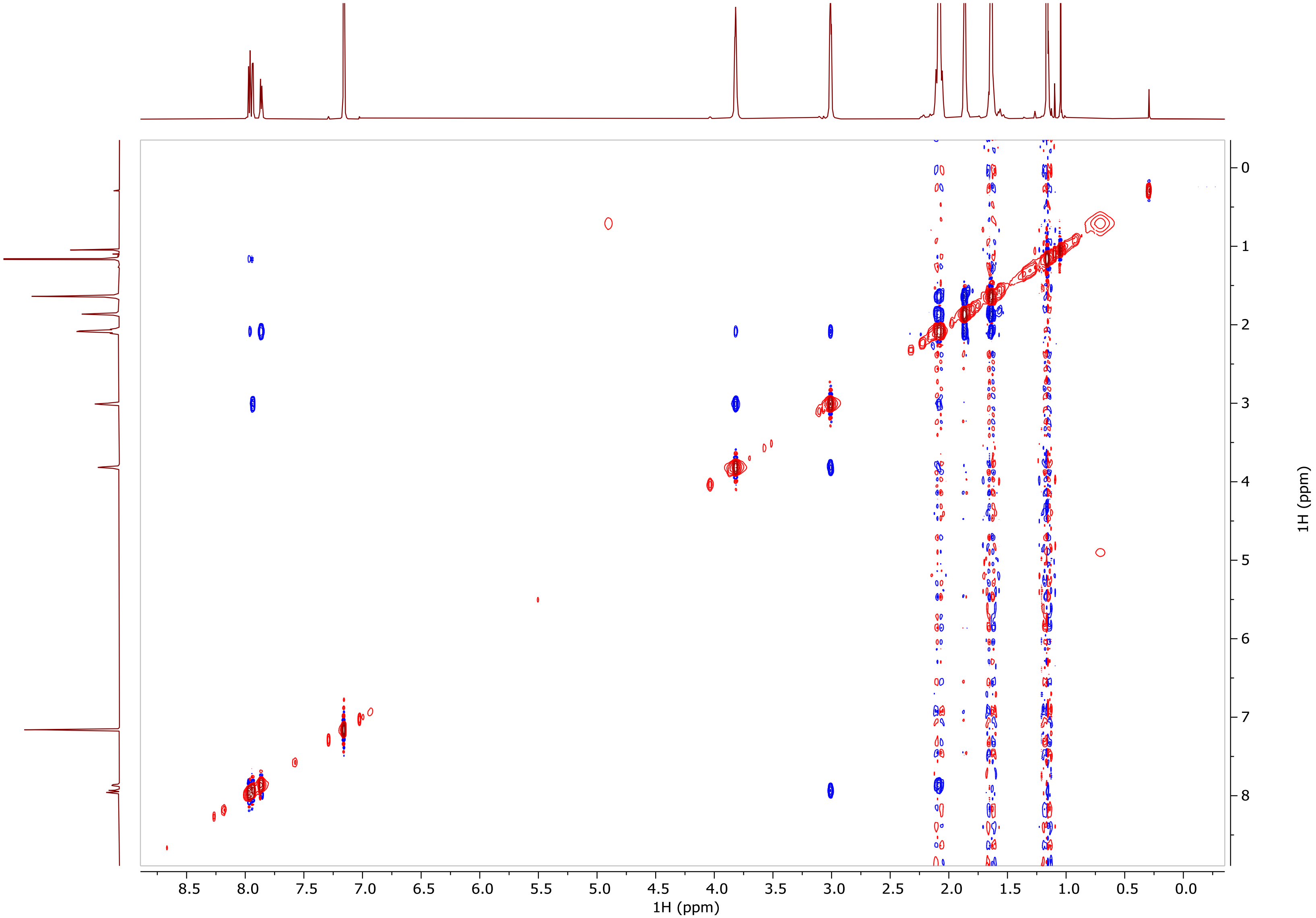

^{1}H NMR of 2'-(dicyclohexylphosphaneyl)-N^2,N^2,N^6,N^6-tetramethyl-4,4'-bis(4,4,5,5-tetramethyl-1,3,2-dioxaborolan-2-yl)-[1,1'-biphenyl]-2,6-diamine (Borylated CPhos, 5)

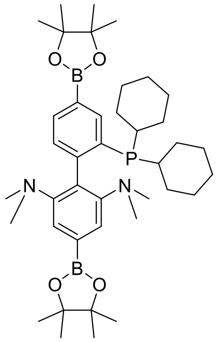

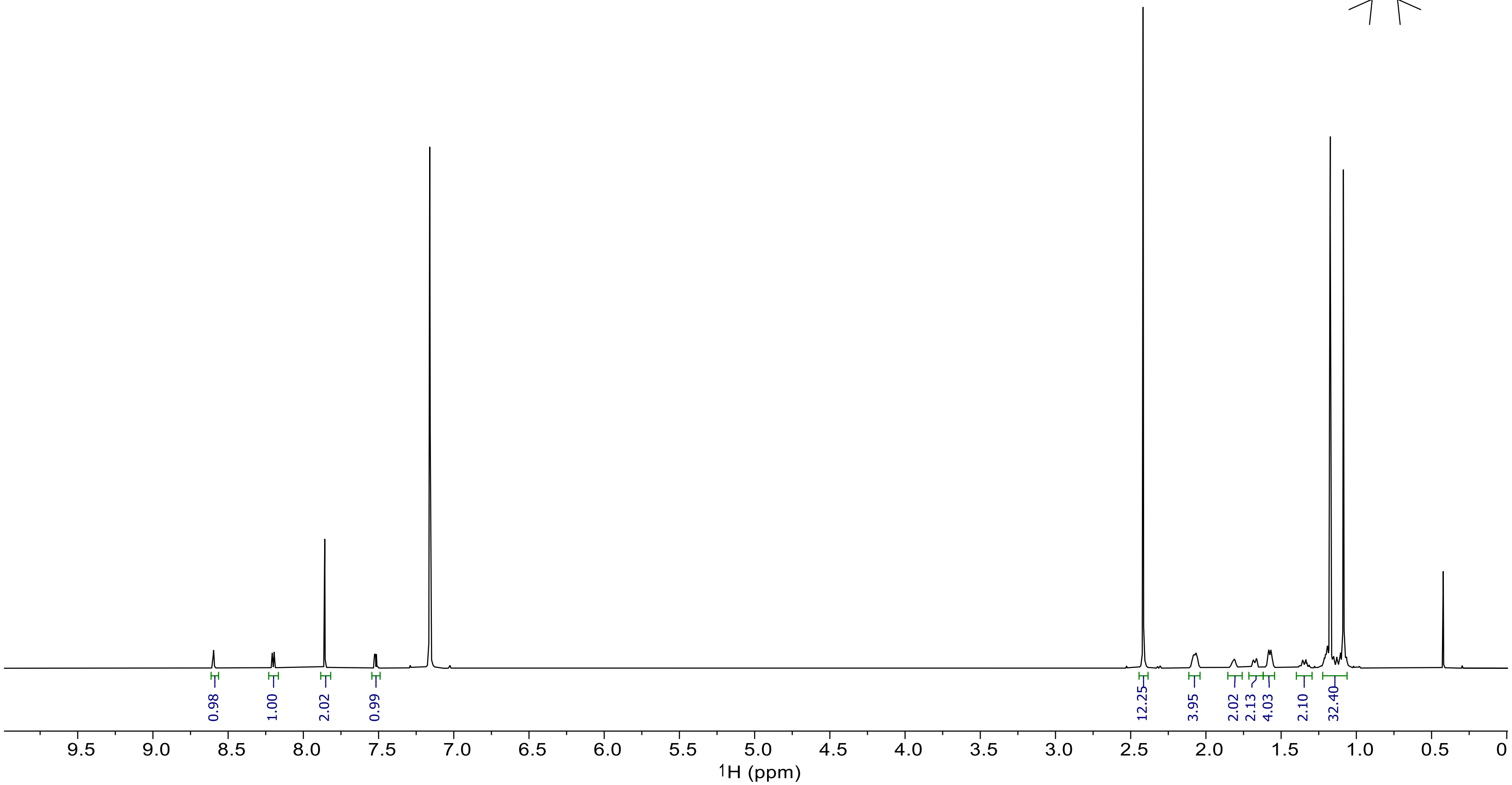

^{13}C NMR of 2'-(dicyclohexylphosphaneyl)-N[2],N[2],N[6],N[6]-tetramethyl-4,4'-bis(4,4,5,5-tetramethyl-1,3,2-dioxaborolan-2-yl)-[1,1'-biphenyl]-2,6-diamine (Borylated CPhos, 5)

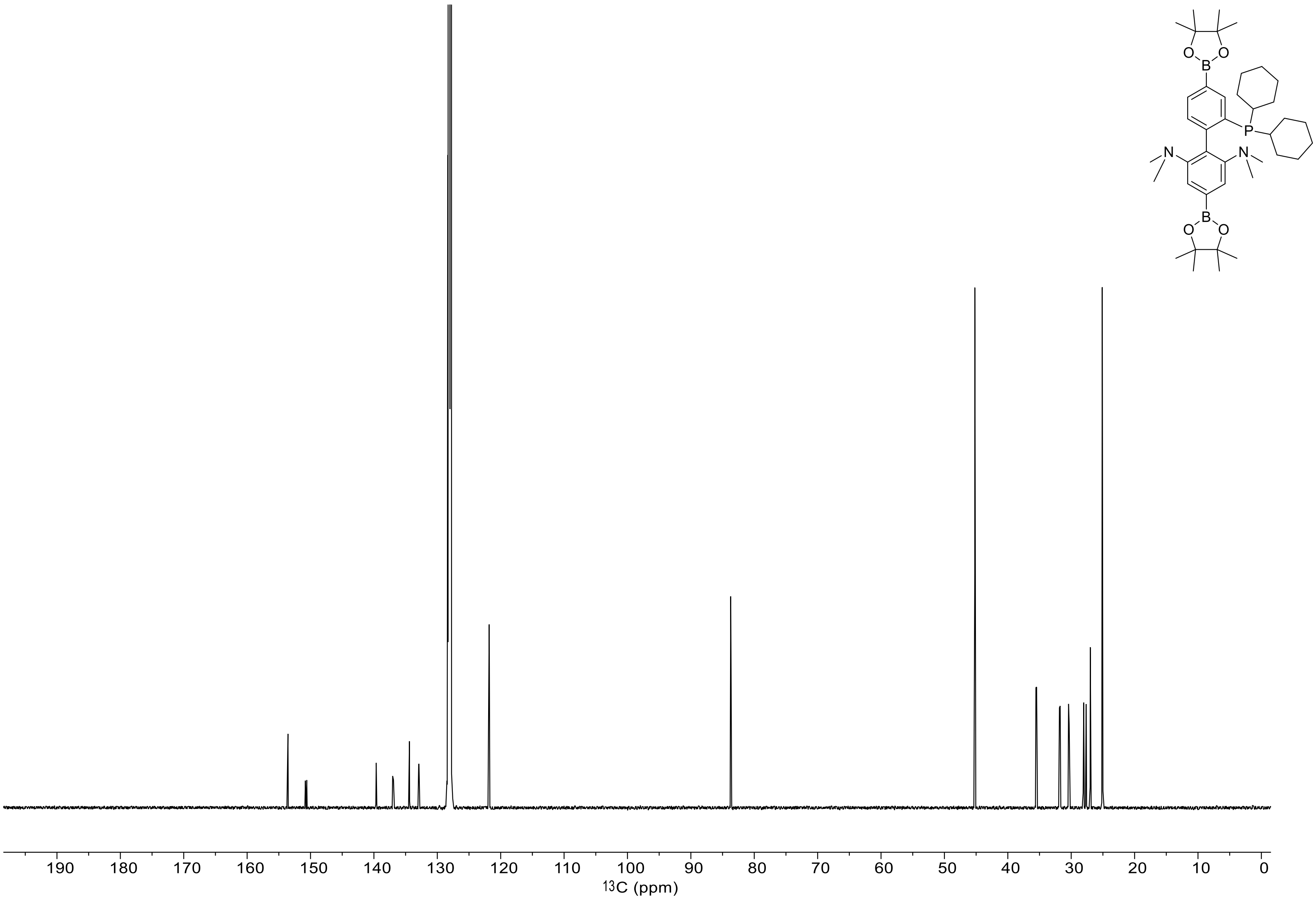

^{31}P NMR of 2'-(dicyclohexylphosphaneyl)-N^2,N^2,N^6,N^6-tetramethyl-4,4'-bis(4,4,5,5-tetramethyl-1,3,2-dioxaborolan-2-yl)-[1,1'-biphenyl]-2,6-diamine (Borylated CPhos, 5)

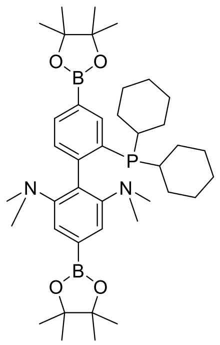

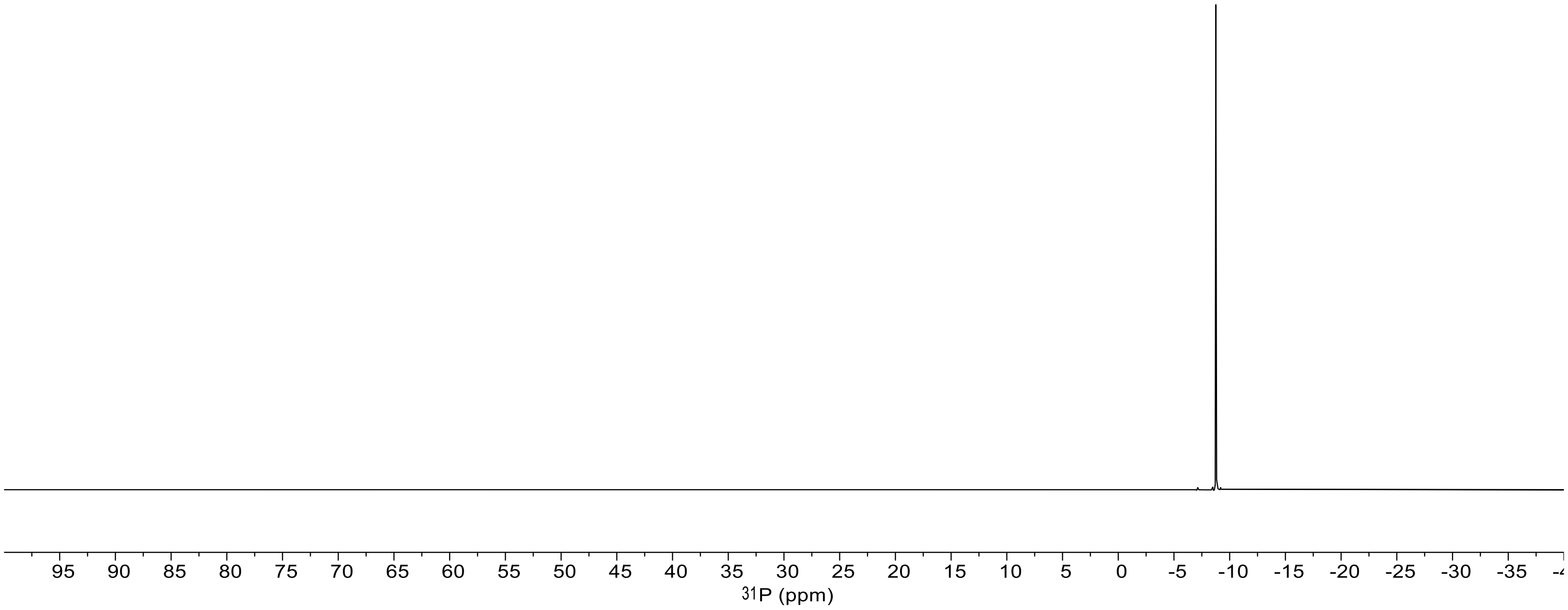

[^{31}P, ^{1}H]-HMBC of 2'-(dicyclohexylphosphaneyl)-N^2,N^2,N^6,N^6-tetramethyl-4,4'-bis(4,4,5,5-tetramethyl-1,3,2-dioxaborolan-2-yl)-[1,1'-biphenyl]-2,6-diamine (Borylated CPhos, 5)

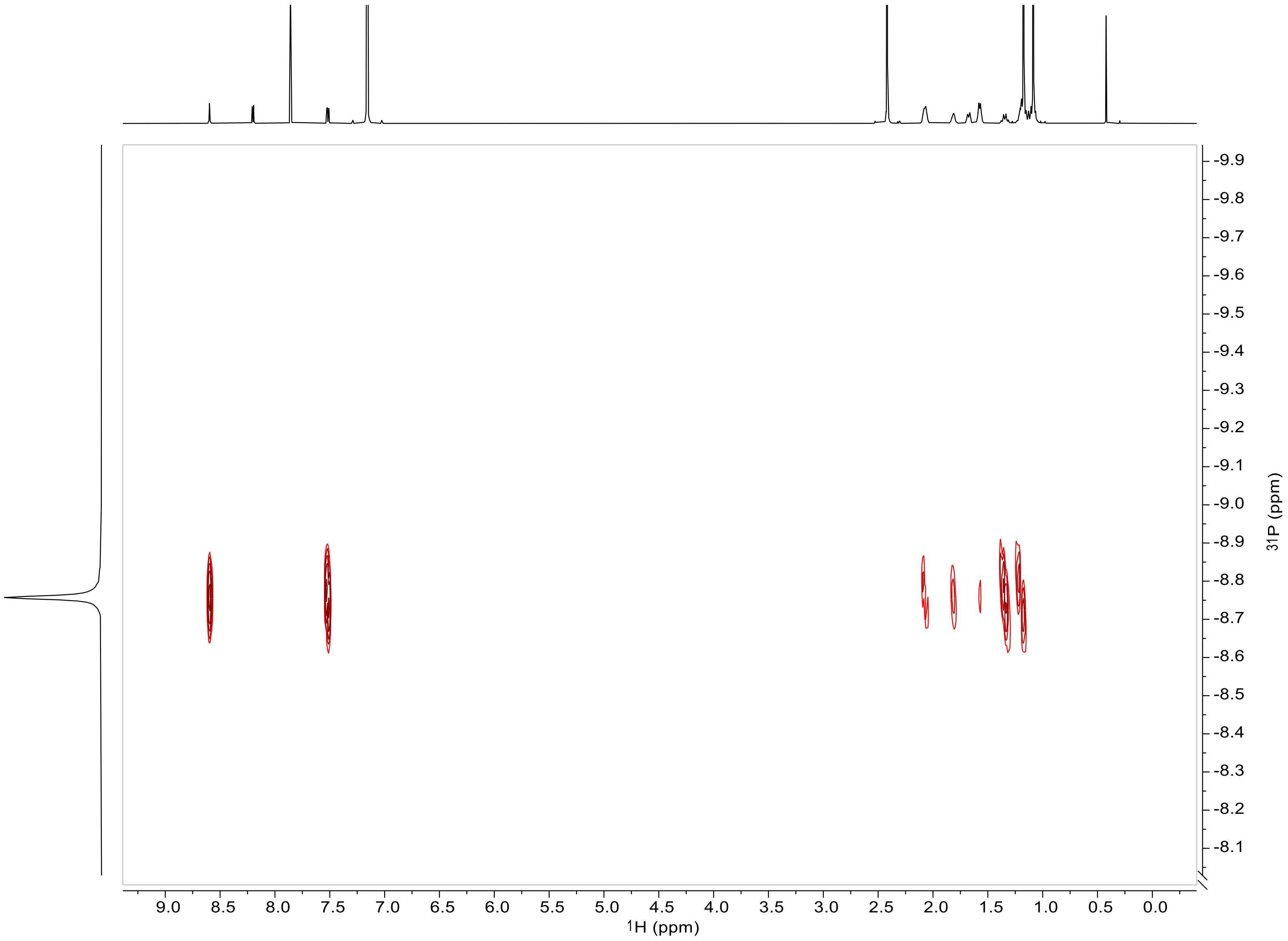

NOESY of 2'-(dicyclohexylphosphaneyl)-N^2,N^2,N^6,N^6-tetramethyl-4,4'-bis(4,4,5,5-tetramethyl-1,3,2-dioxaborolan-2-yl)-[1,1'-biphenyl]-2,6-diamine (Borylated CPhos, 5)

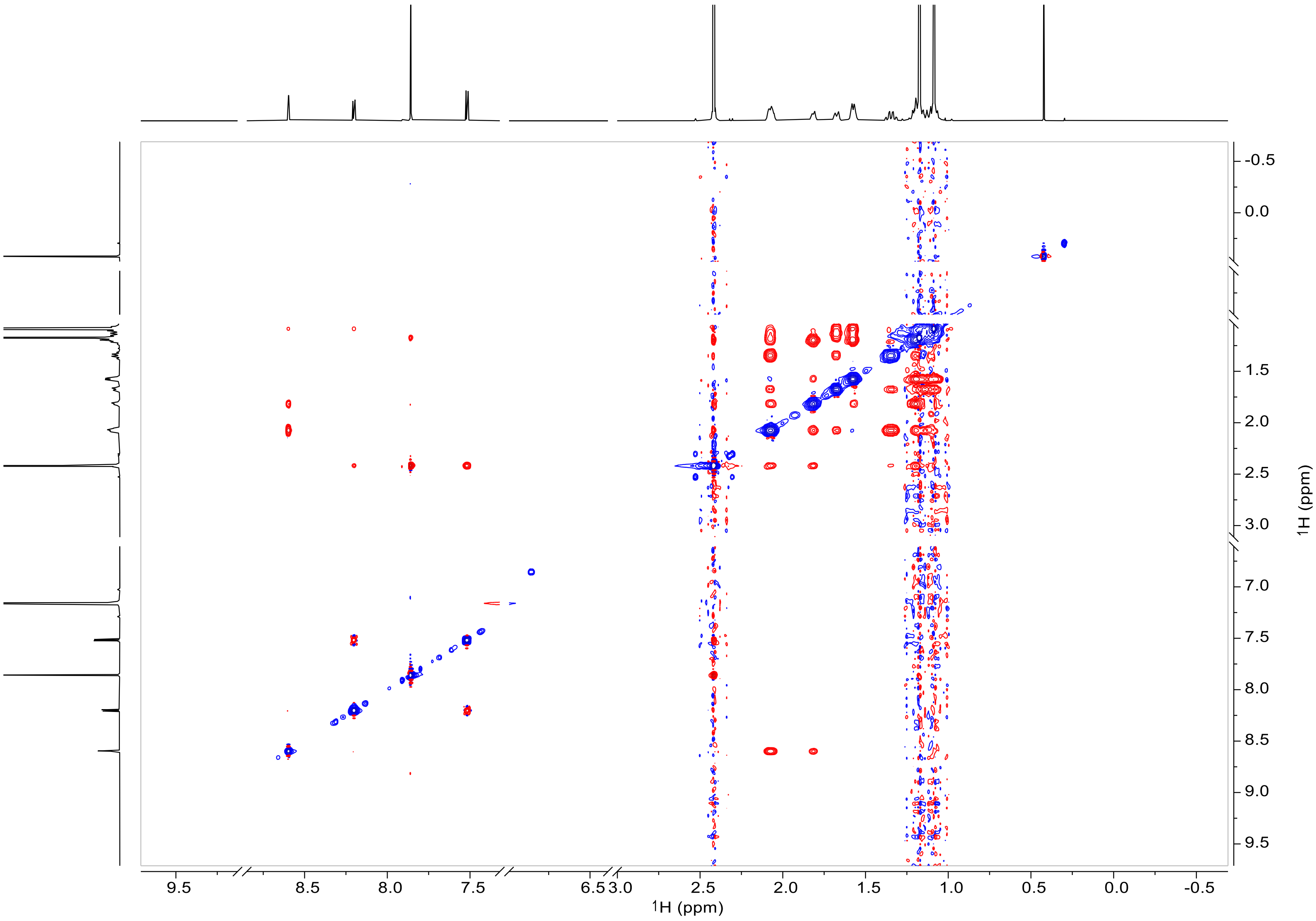

^{1}H NMR of 1-(2,6-bis(cyclohexyloxy)-4-(4,4,5,5-tetramethyl-1,3,2-dioxaborolan-2-yl)phenyl)-2-(di-tert-butylphosphaneyl)-4-(4,4,5,5-tetramethyl-1,3,2-dioxaborolan-2-yl)-1H-imidazole (6)

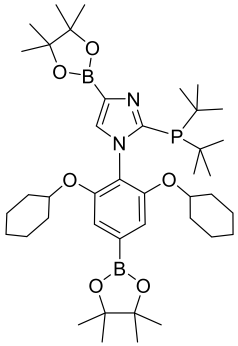

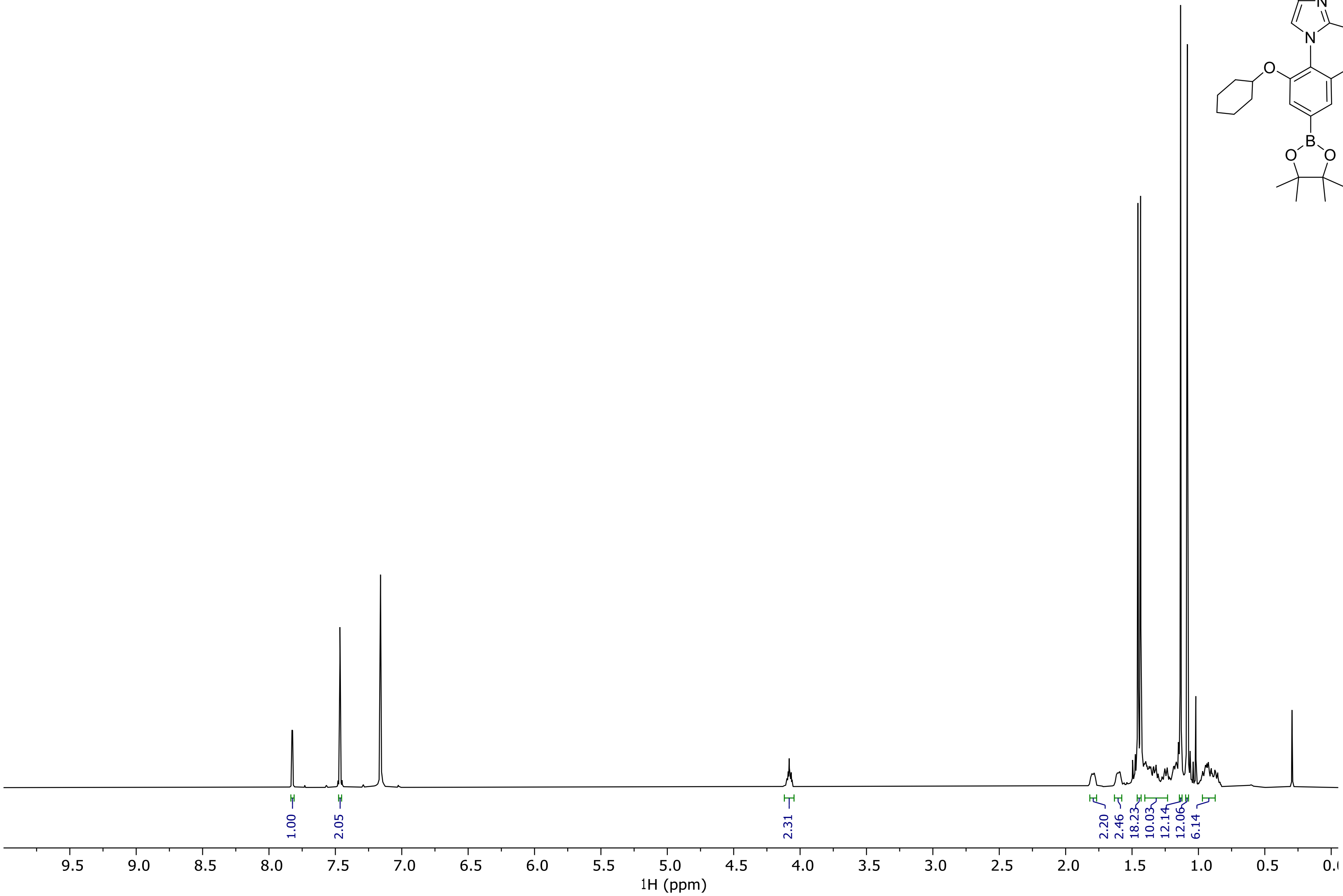

^{13}C NMR of 1-(2,6-bis(cyclohexyloxy)-4-(4,4,5,5-tetramethyl-1,3,2-dioxaborolan-2-yl)phenyl)-2-(di-tert-butylphosphaneyl)-4-(4,4,5,5-tetramethyl-1,3,2-dioxaborolan-2-yl)-1H-imidazole (6)

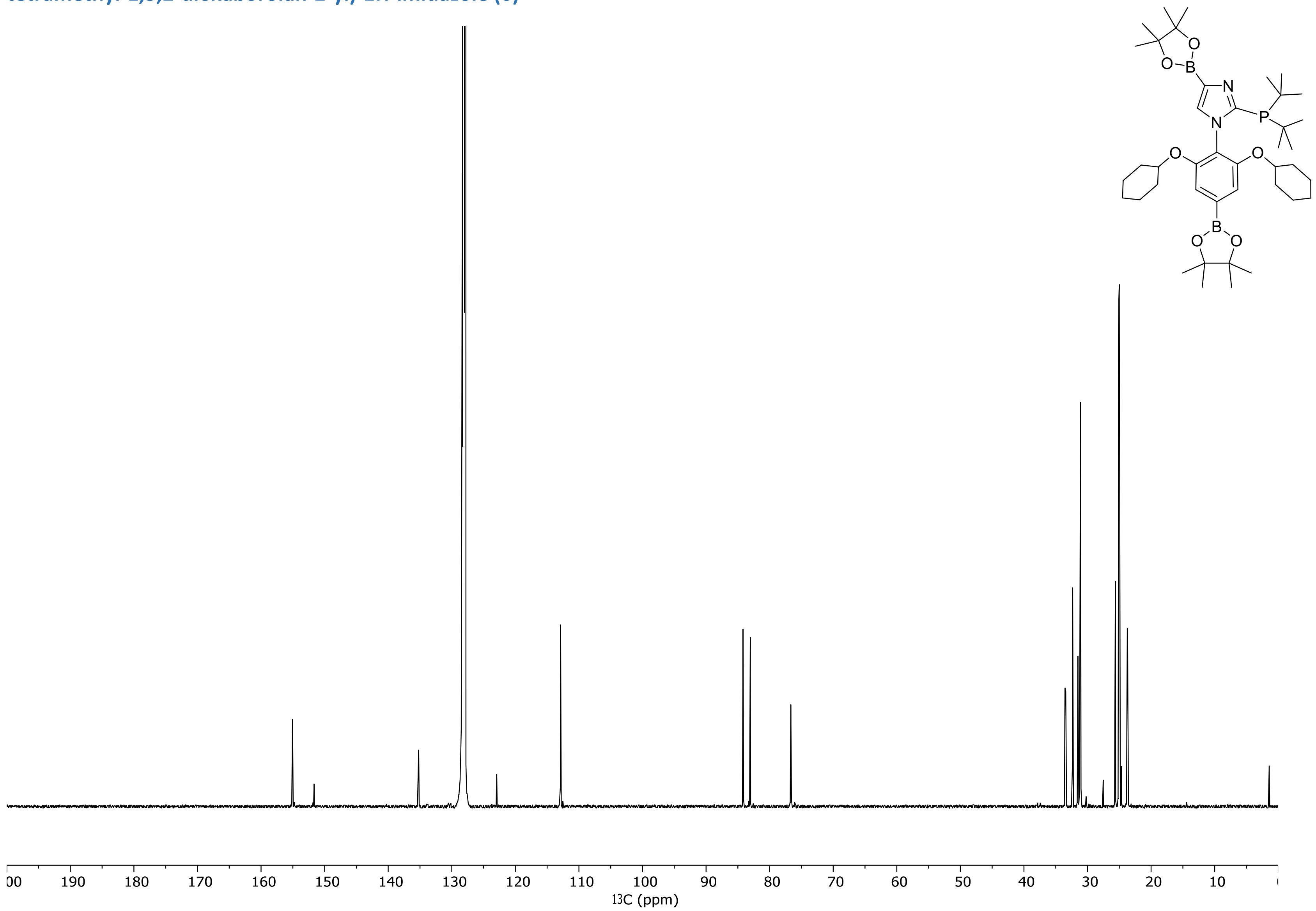

^{31}P NMR of 1-(2,6-bis(cyclohexyloxy)-4-(4,4,5,5-tetramethyl-1,3,2-dioxaborolan-2-yl)phenyl)-2-(di-tert-butylphosphaneyl)-4-(4,4,5,5-tetramethyl-1,3,2-dioxaborolan-2-yl)-1H-imidazole (6)

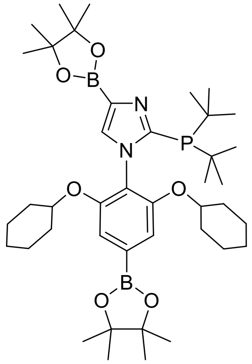

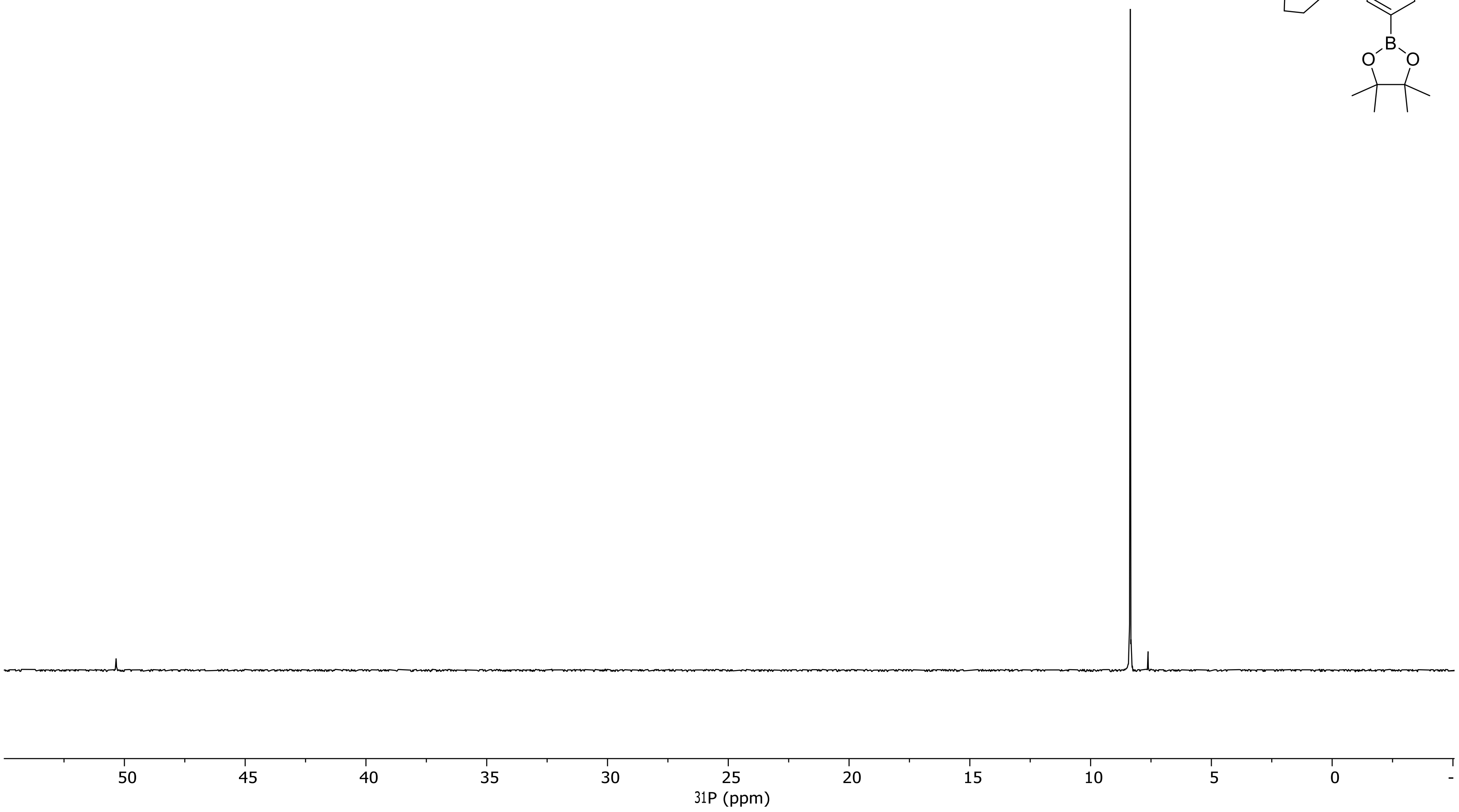

[^{13}C, ^{1}H]-HMBC of 1-(2,6-bis(cyclohexyloxy)-4-(4,4,5,5-tetramethyl-1,3,2-dioxaborolan-2-yl)phenyl)-2-(di-tert-butylphosphaneyl)-4-(4,4,5,5-tetramethyl-1,3,2-dioxaborolan-2-yl)-1H-imidazole (6)

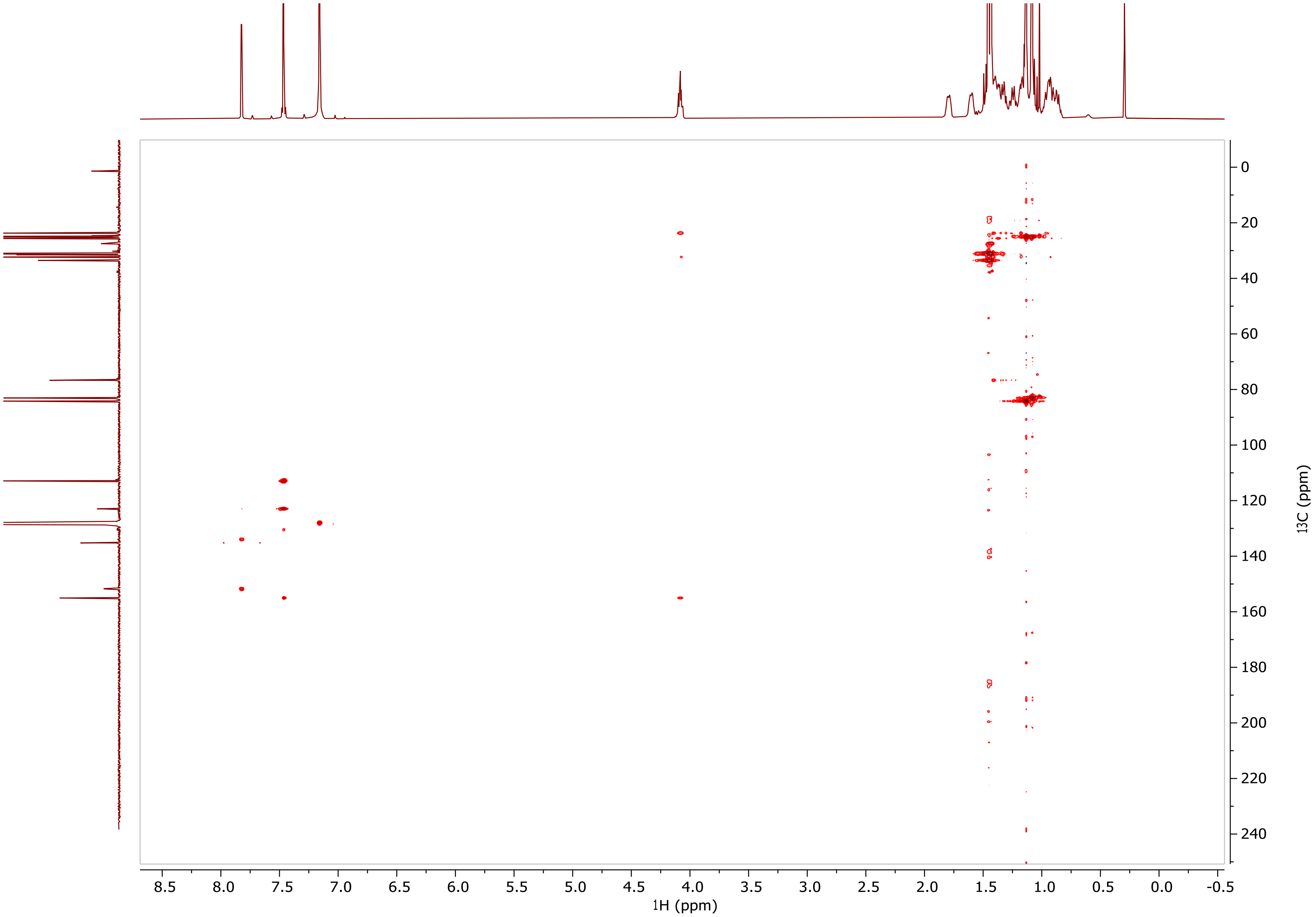

[^{31}P, ^{1}H]-HMBC of 1-(2,6-bis(cyclohexyloxy)-4-(4,4,5,5-tetramethyl-1,3,2-dioxaborolan-2-yl)phenyl)-2-(di-tert-butylphosphaneyl)-4-(4,4,5,5-tetramethyl-1,3,2-dioxaborolan-2-yl)-1H-imidazole (6)

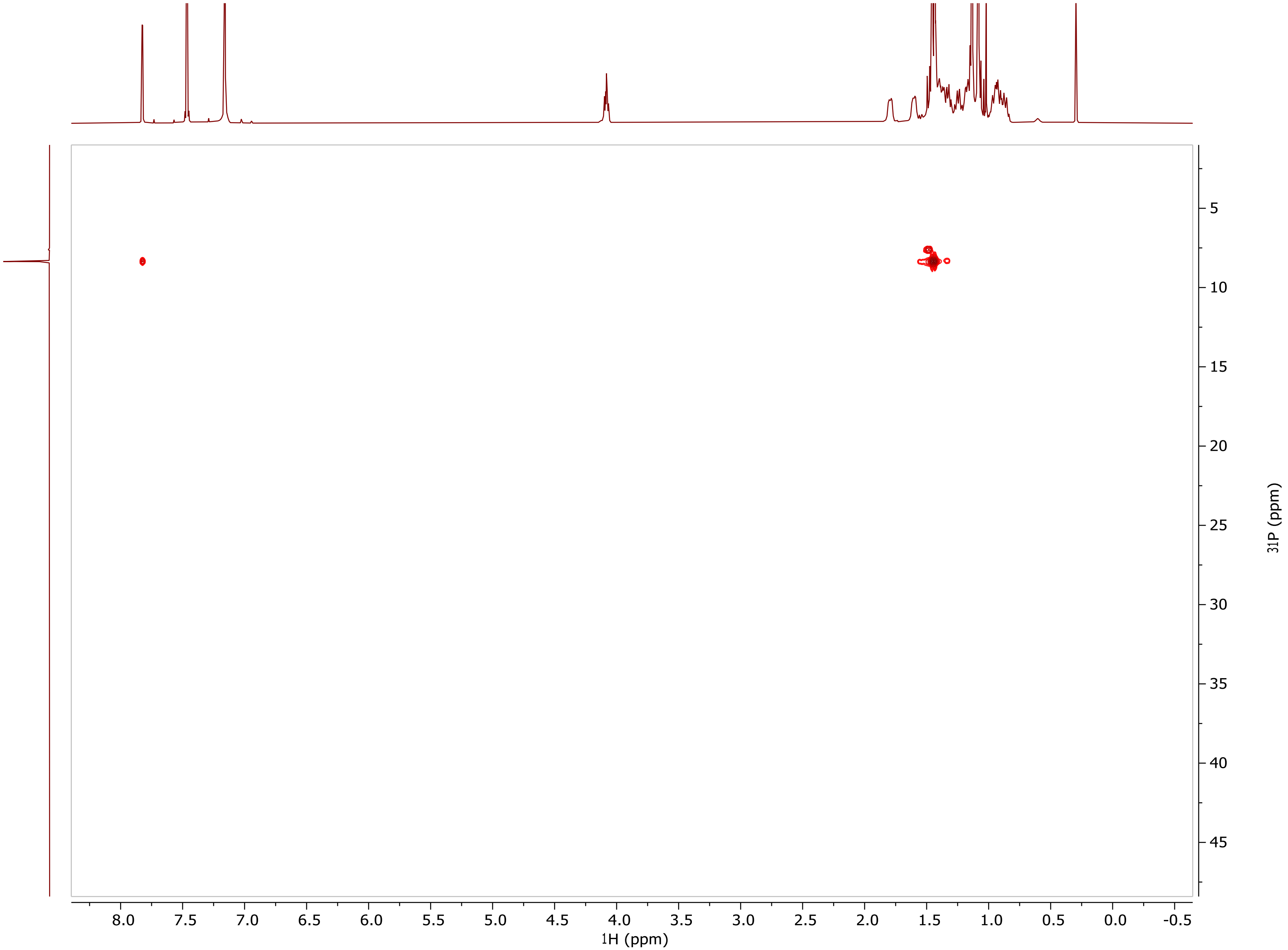

NOESY NMR of 1-(2,6-bis(cyclohexyloxy)-4-(4,4,5,5-tetramethyl-1,3,2-dioxaborolan-2-yl)phenyl)-2-(di-tert-butylphosphaneyl)-4-(4,4,5,5-tetramethyl-1,3,2-dioxaborolan-2-yl)-1H-imidazole (6)

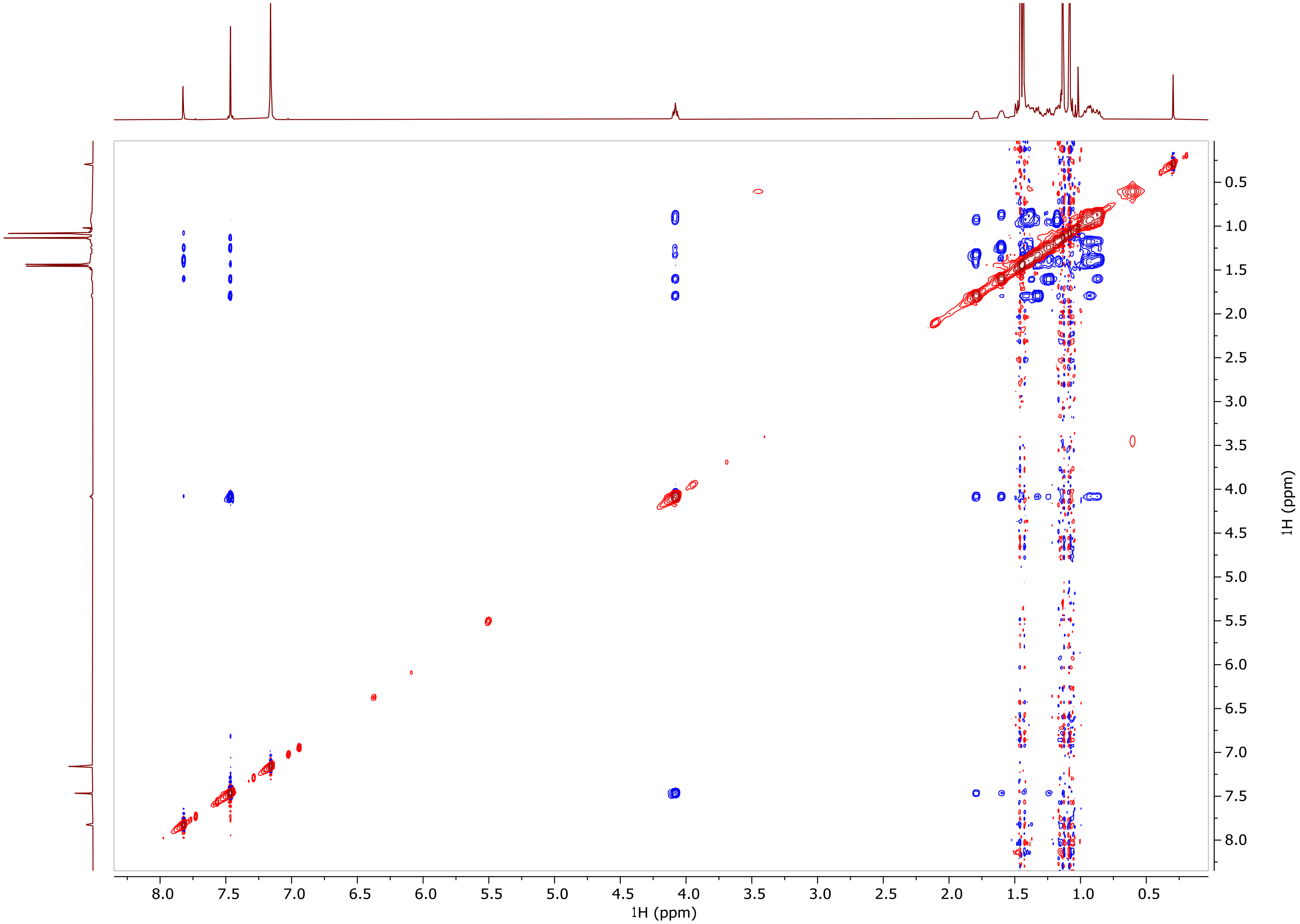

^{1}H NMR of tris(2,6-dimethoxy-4-(4,4,5,5-tetramethyl-1,3,2-dioxaborolan-2-yl)phenyl)phosphane (7)

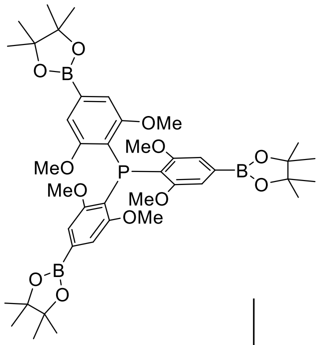

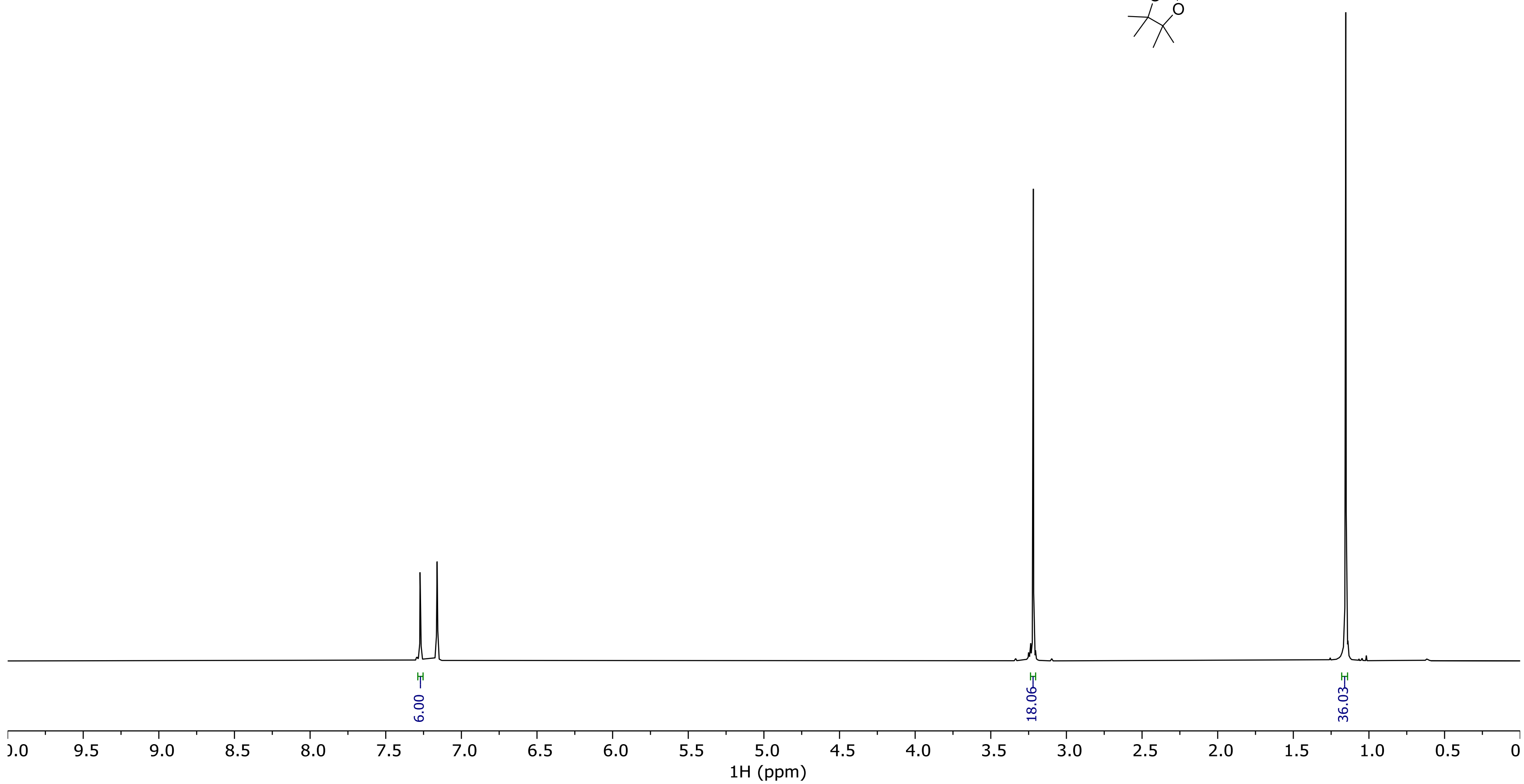

^{13}C NMR of tris(2,6-dimethoxy-4-(4,4,5,5-tetramethyl-1,3,2-dioxaborolan-2-yl)phenyl)phosphane (7)

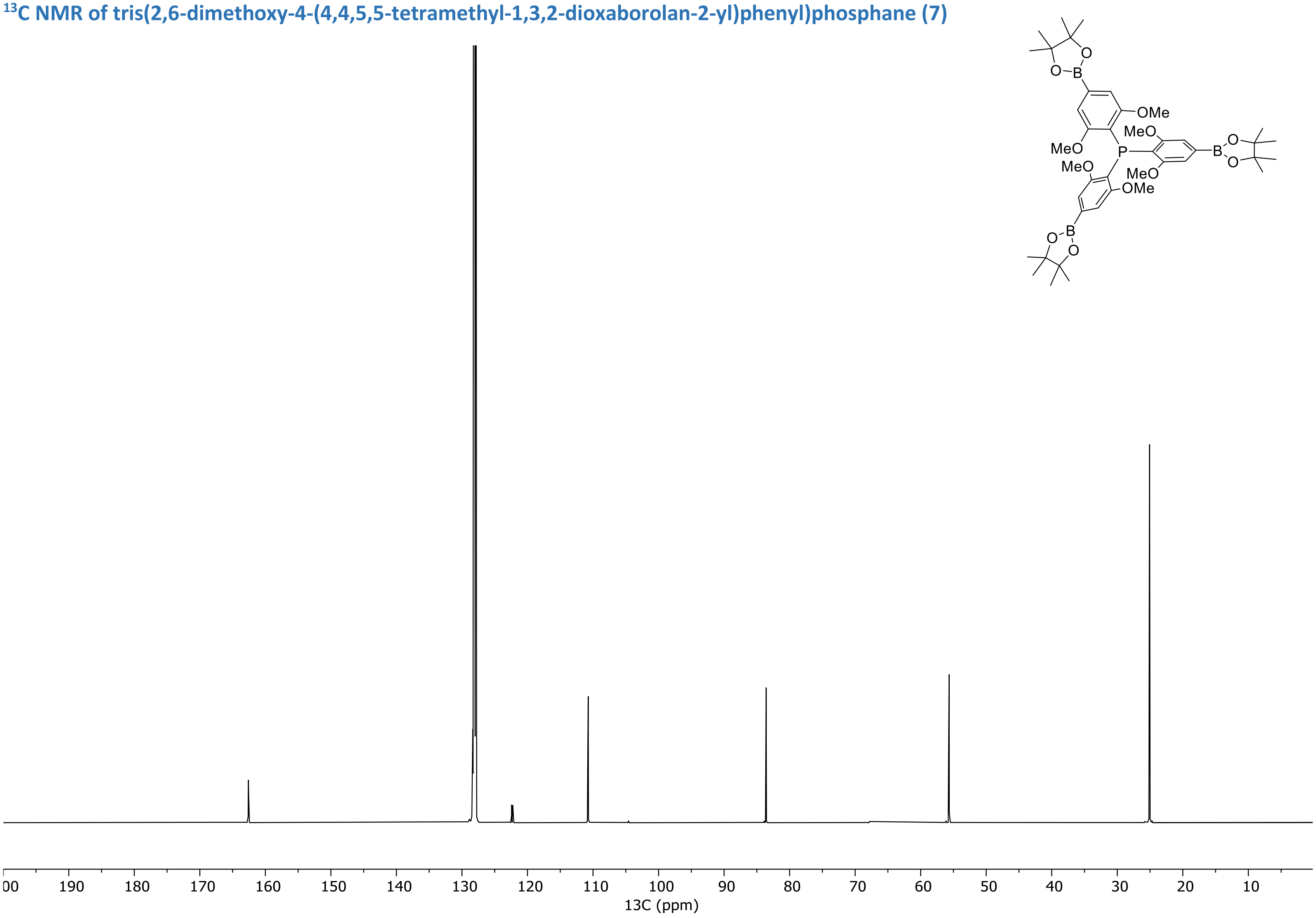

^{31}P NMR of tris(2,6-dimethoxy-4-(4,4,5,5-tetramethyl-1,3,2-dioxaborolan-2-yl)phenyl)phosphane (7)

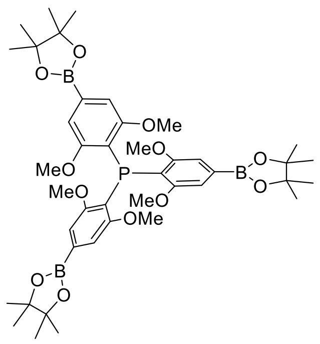

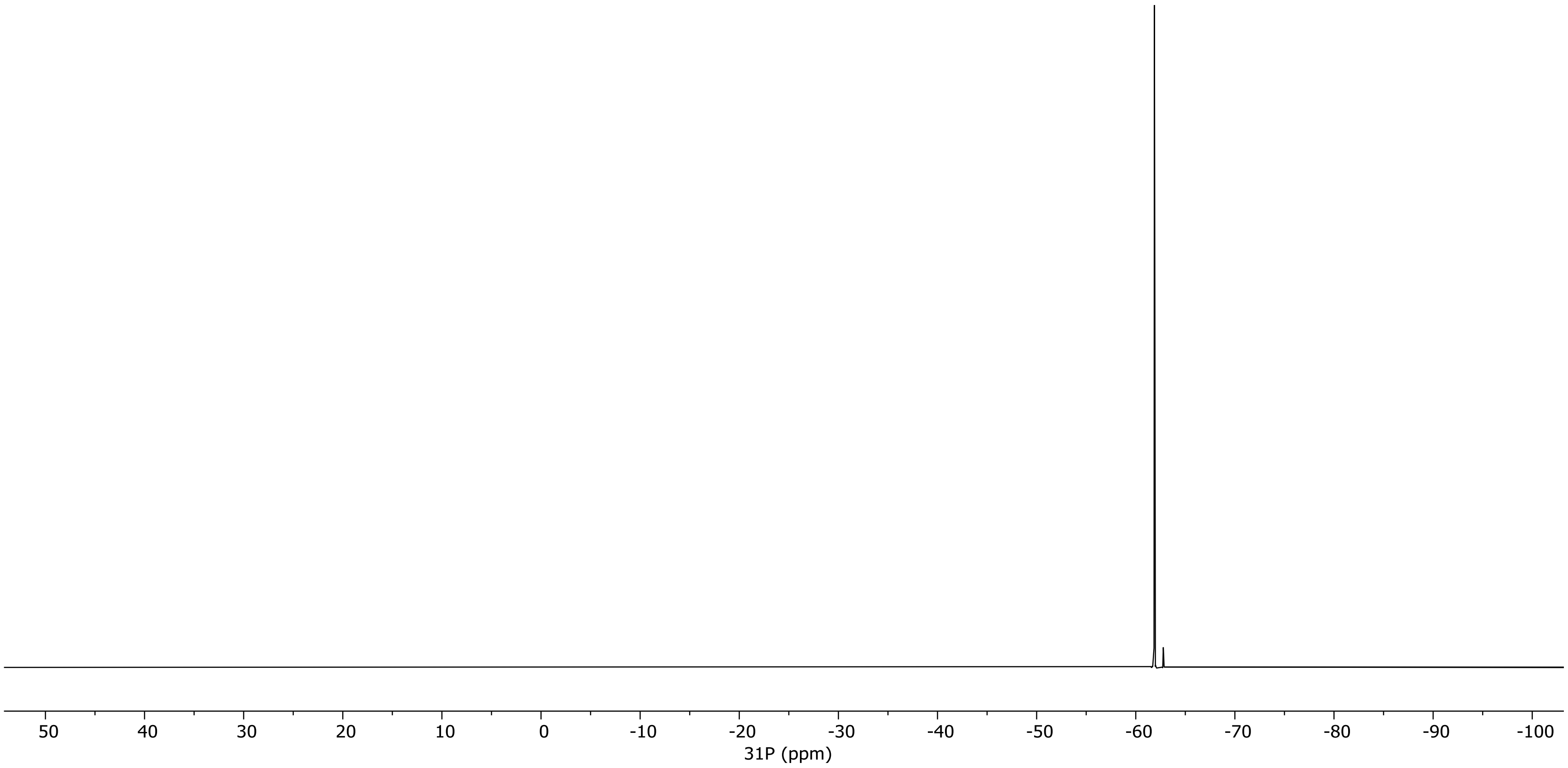

^{1}H NMR of diphenyl(2'-(4,4,5,5-tetramethyl-1,3,2-dioxaborolan-2-yl)-[1,1'-biphenyl]-2-yl)phosphane (12)

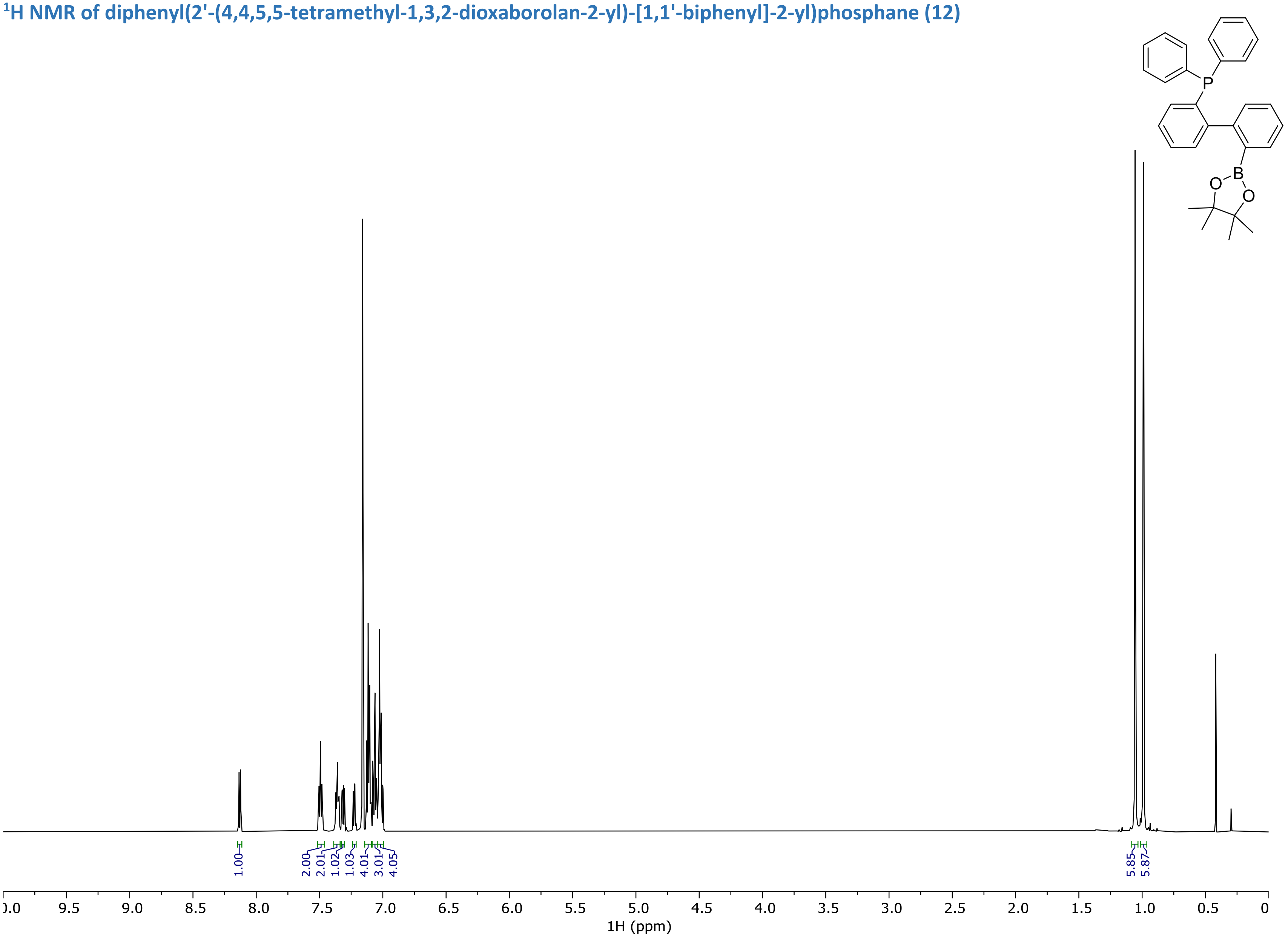

^{13}C NMR of diphenyl(2'-(4,4,5,5-tetramethyl-1,3,2-dioxaborolan-2-yl)-[1,1'-biphenyl]-2-yl)phosphane (12)

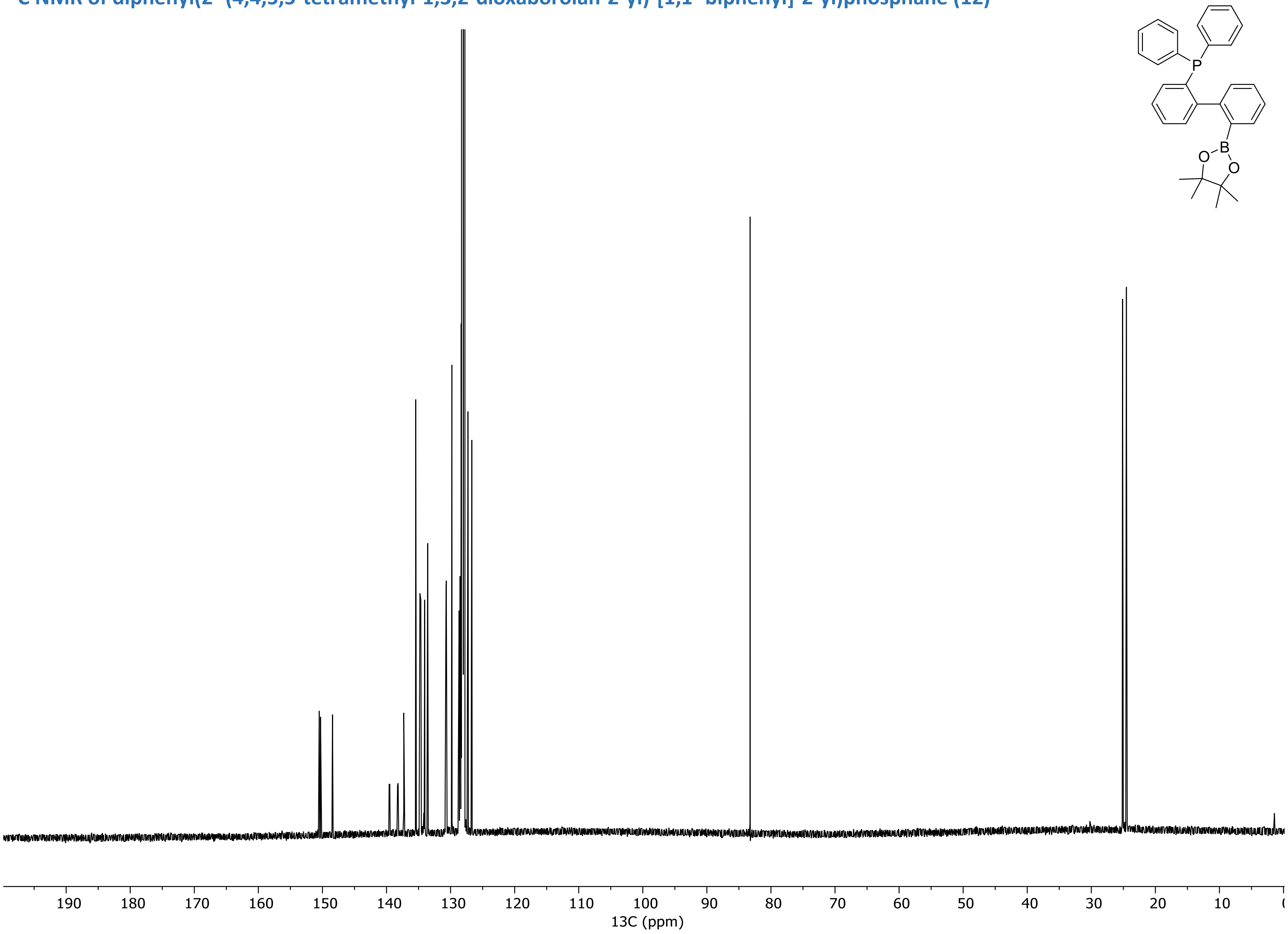

^{31}P NMR of diphenyl(2'-(4,4,5,5-tetramethyl-1,3,2-dioxaborolan-2-yl)-[1,1'-biphenyl]-2-yl)phosphane (12)

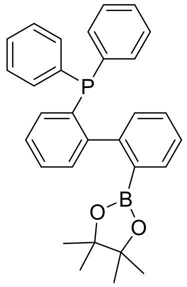

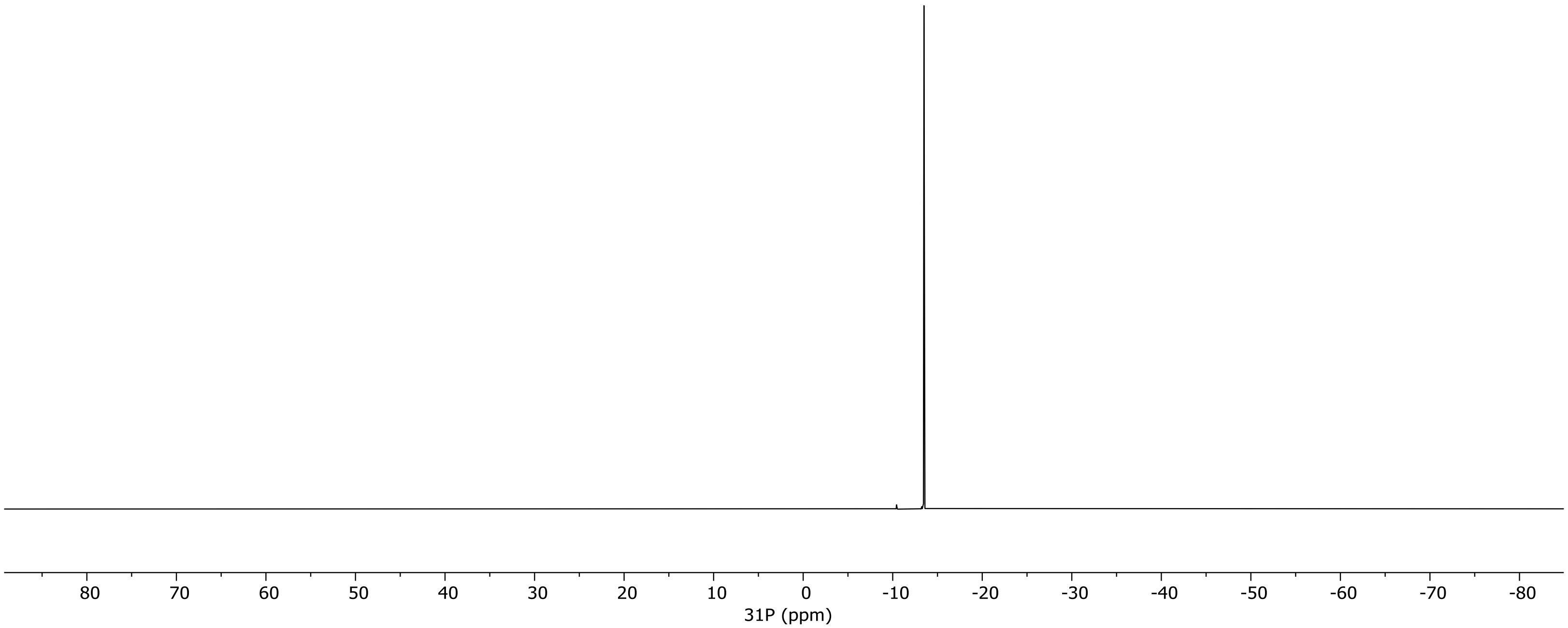

^{1}H NMR of (5-methyl-2-(4,4,5,5-tetramethyl-1,3,2-dioxaborolan-2-yl)phenyl)di-m-tolylphosphane (13 : S3 = 7 : 1)

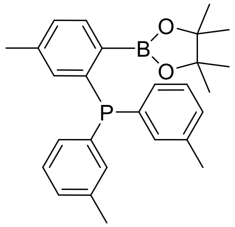

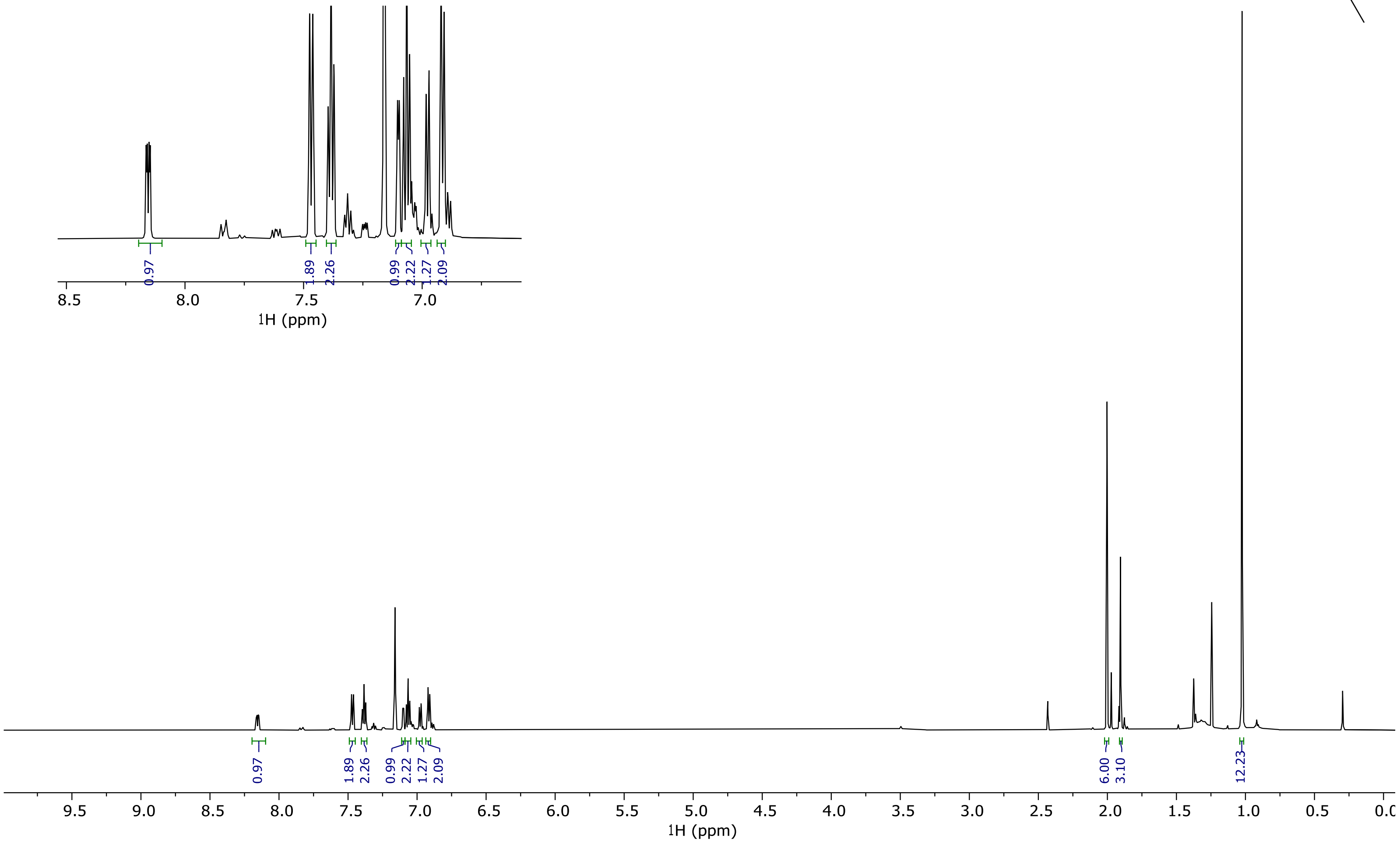

^{13}C NMR of (5-methyl-2-(4,4,5,5-tetramethyl-1,3,2-dioxaborolan-2-yl)phenyl)di-m-tolylphosphane (13 : S3 = 7 : 1)

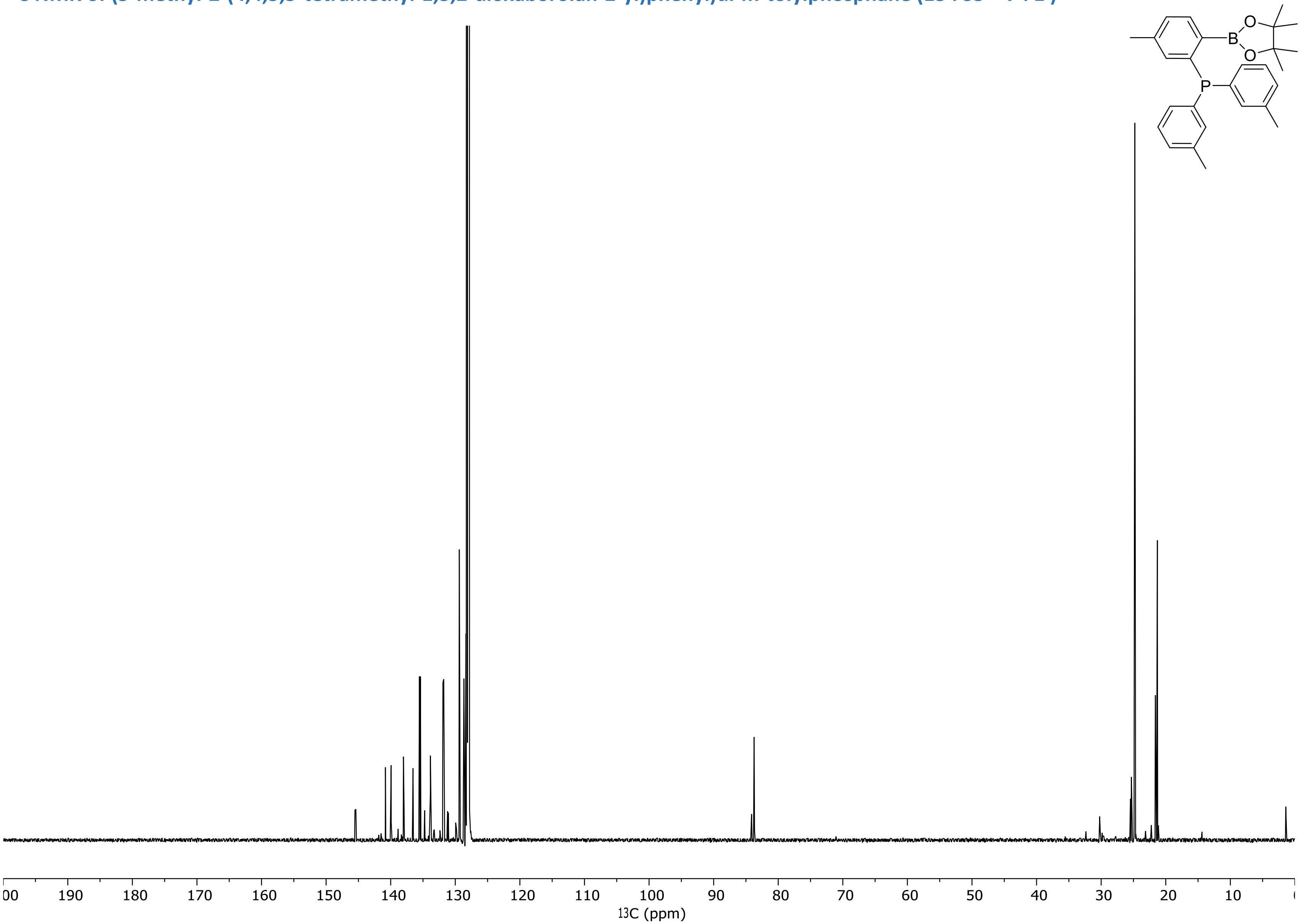

^{31}P NMR of (5-methyl-2-(4,4,5,5-tetramethyl-1,3,2-dioxaborolan-2-yl)phenyl)di-m-tolylphosphane (13 : S3 = 7 : 1)

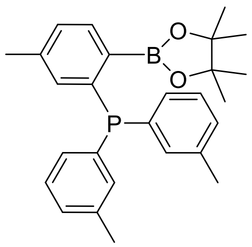

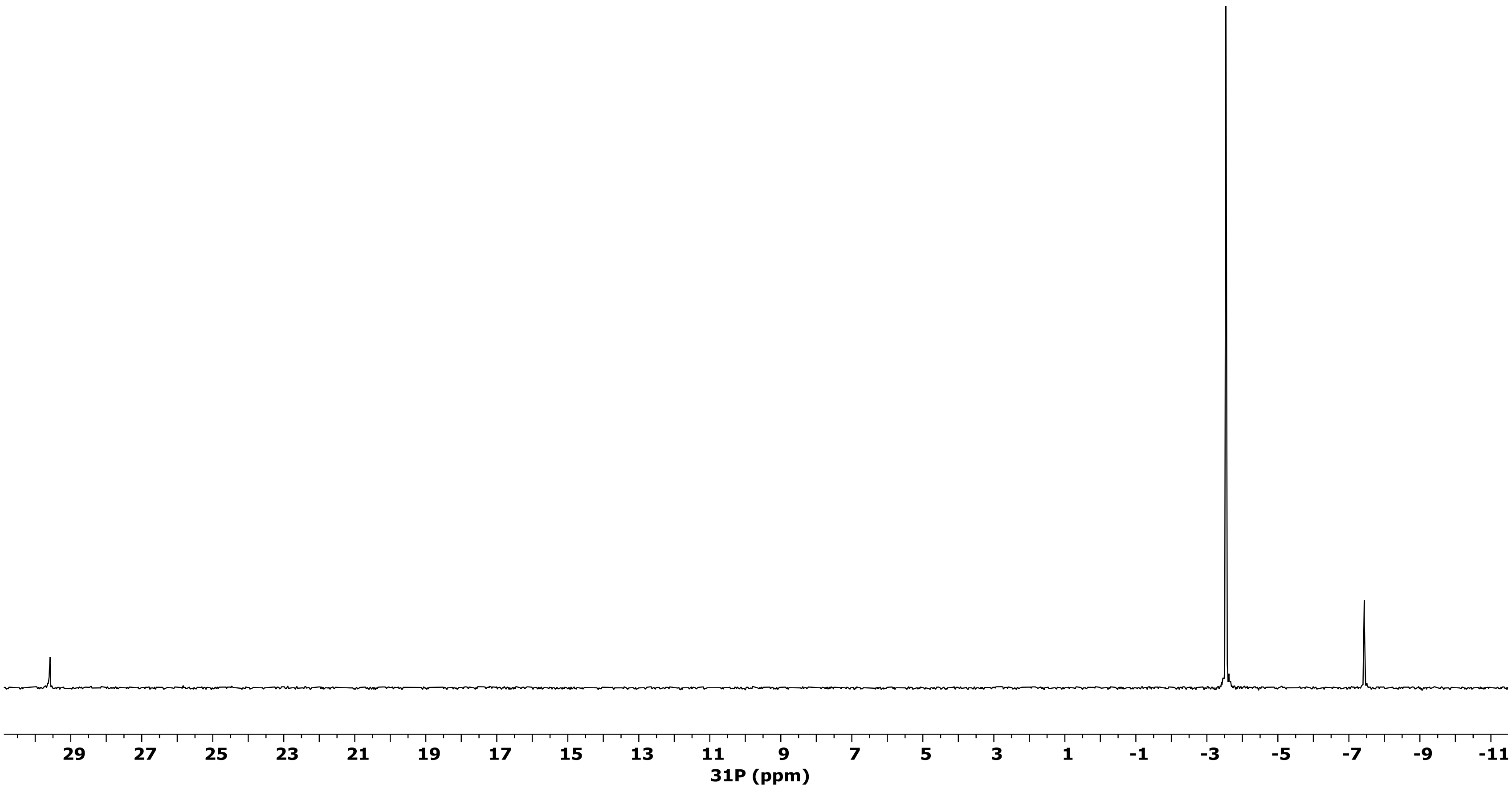

^{1}H NMR of (5-chloro-2-(4,4,5,5-tetramethyl-1,3,2-dioxaborolan-2-yl)phenyl)bis(3-chlorophenyl)phosphane (14)

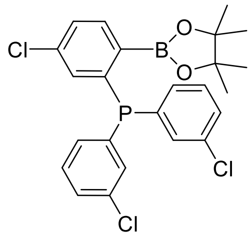

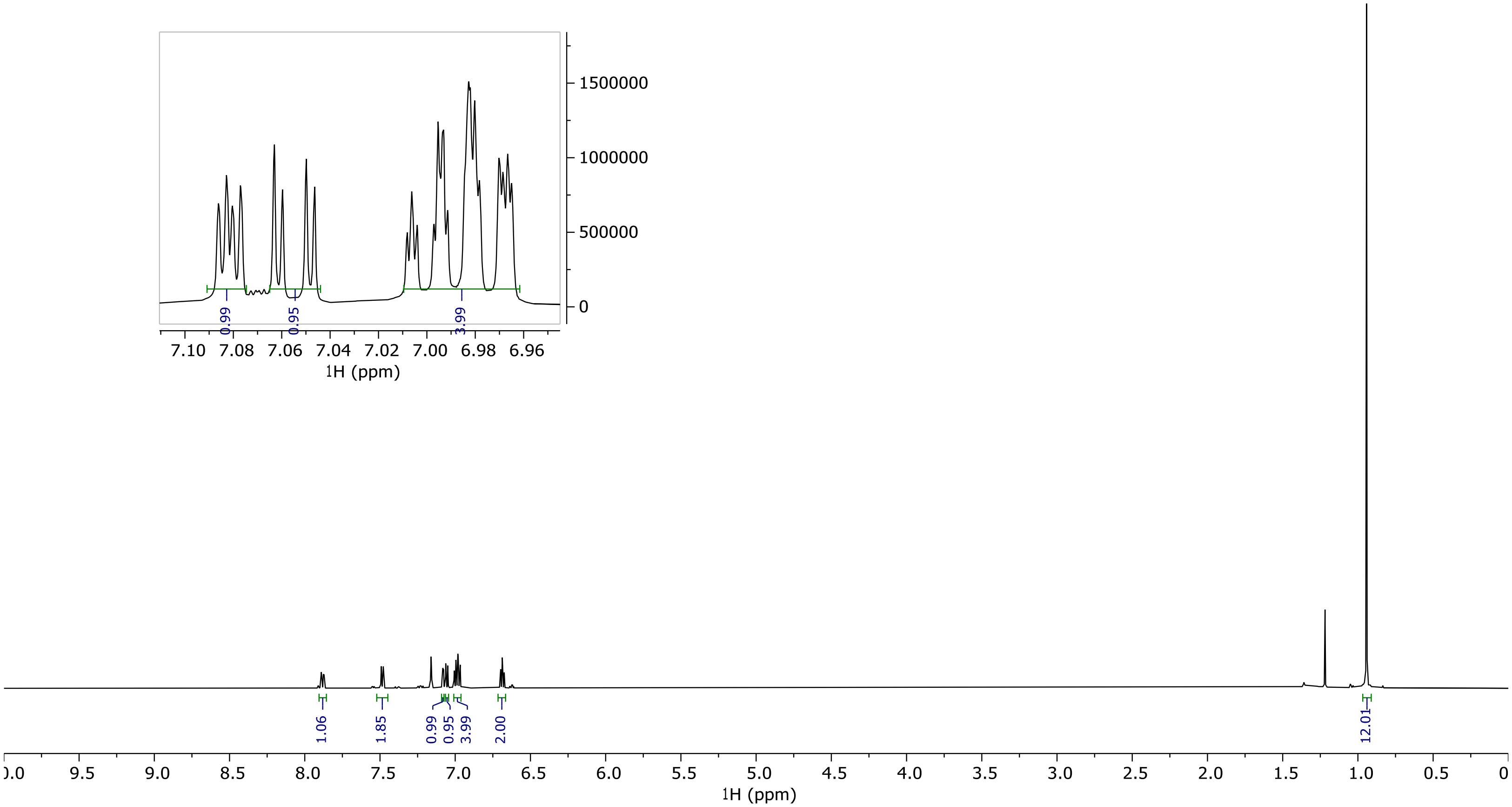

^{13}C NMR of (5-chloro-2-(4,4,5,5-tetramethyl-1,3,2-dioxaborolan-2-yl)phenyl)bis(3-chlorophenyl)phosphane (14)

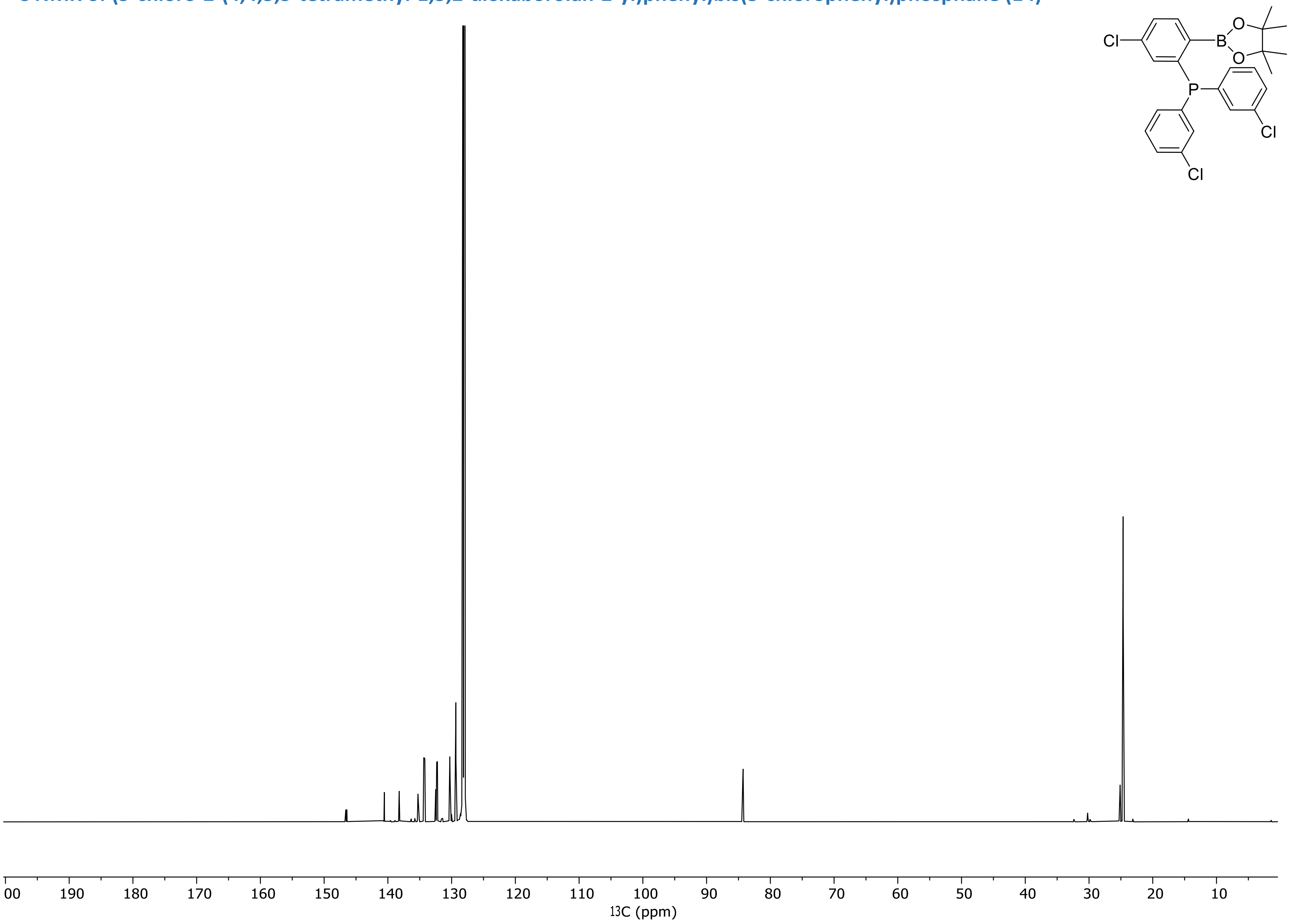

^{31}P NMR of (5-chloro-2-(4,4,5,5-tetramethyl-1,3,2-dioxaborolan-2-yl)phenyl)bis(3-chlorophenyl)phosphane (14)

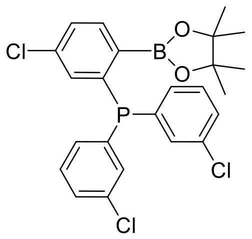

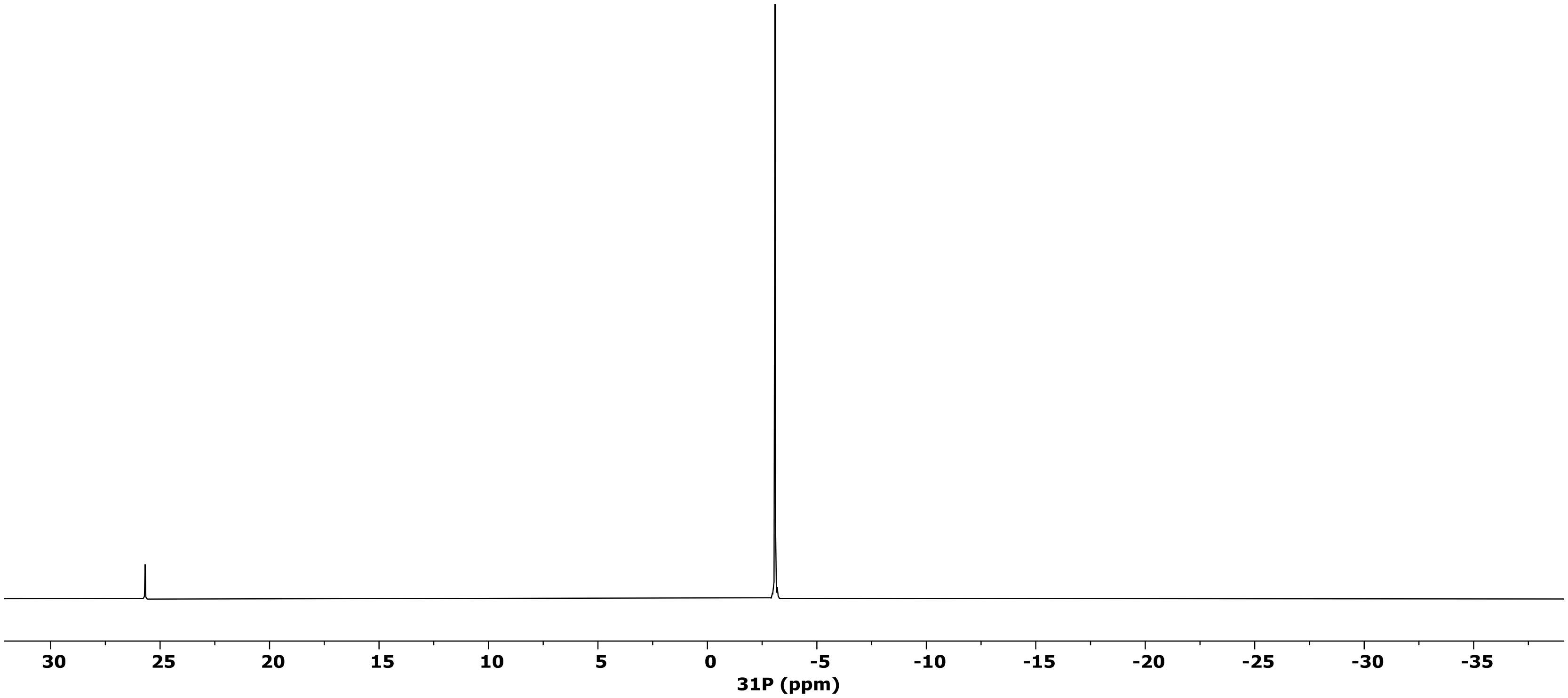

^{1}H NMR of (3-chloro-2-(4,4,5,5-tetramethyl-1,3,2-dioxaborolan-2-yl)phenyl)bis(3-chlorophenyl)phosphane (15)

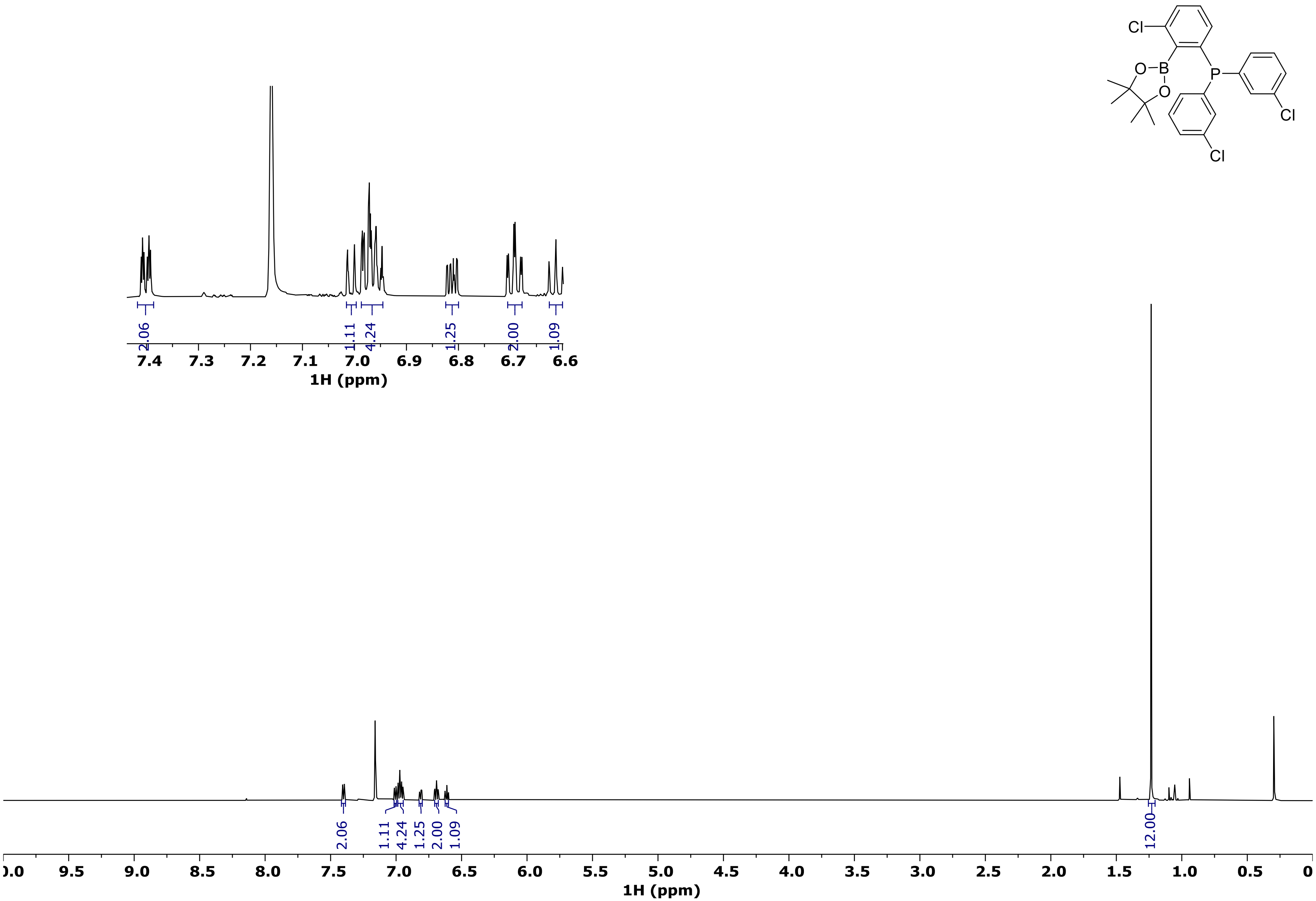

^{13}C NMR of (3-chloro-2-(4,4,5,5-tetramethyl-1,3,2-dioxaborolan-2-yl)phenyl)bis(3-chlorophenyl)phosphane (15)

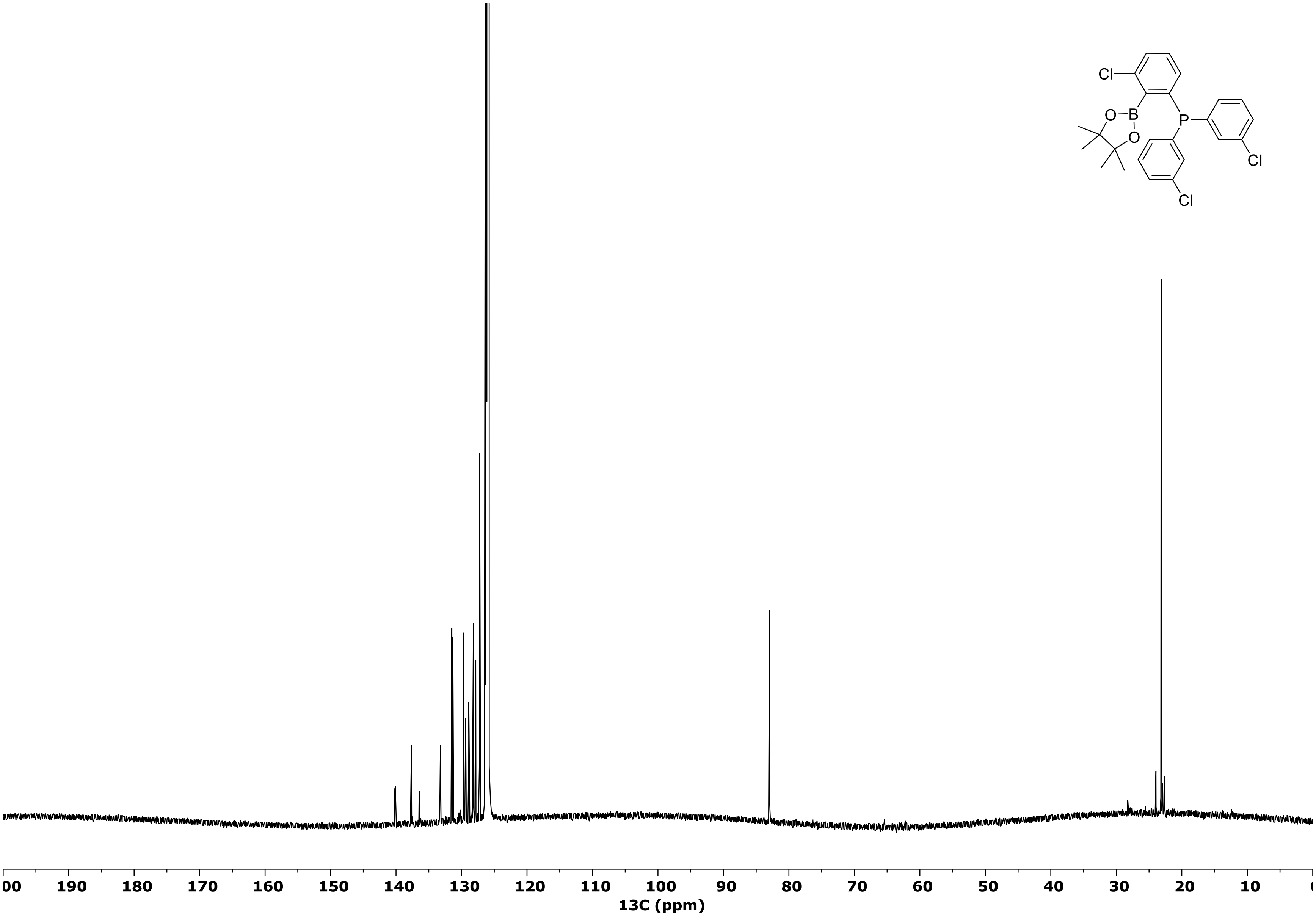

^{31}P NMR of (3-chloro-2-(4,4,5,5-tetramethyl-1,3,2-dioxaborolan-2-yl)phenyl)bis(3-chlorophenyl)phosphane (15)

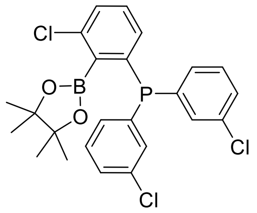

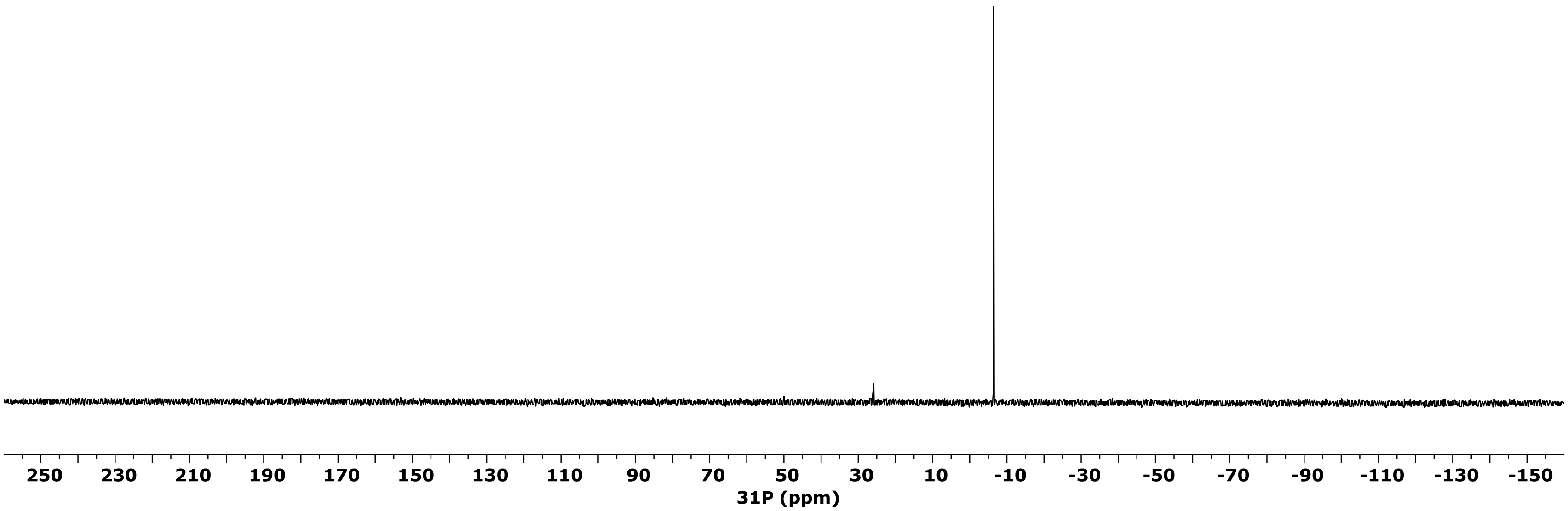

^{1}H NMR of borylated [1-(Diphenylphosphino)ethyl]ferrocene (16)

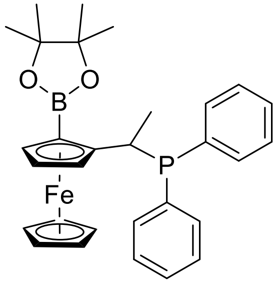

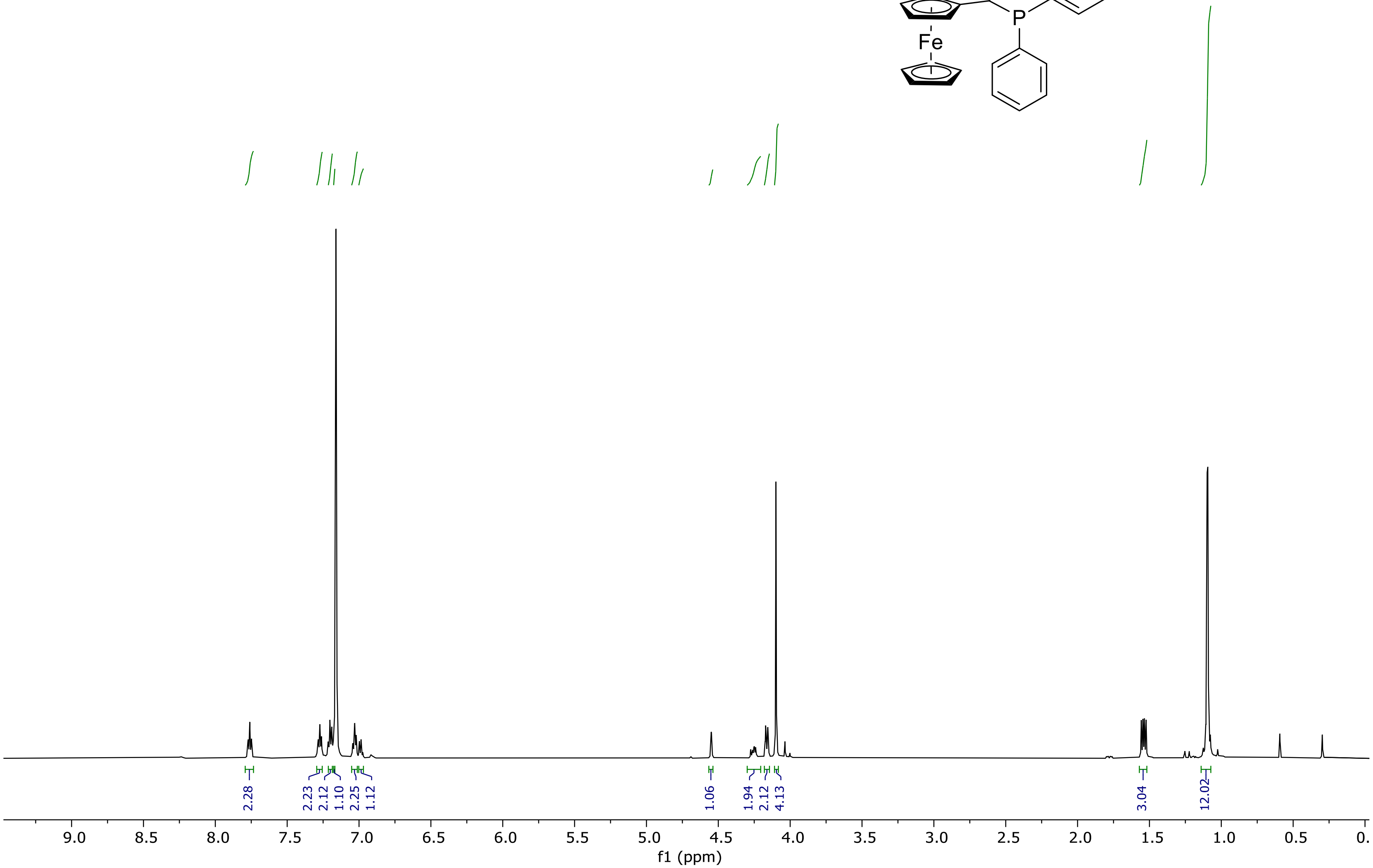

^{13}C NMR of borylated [1-(Diphenylphosphino)ethyl]ferrocene (16)

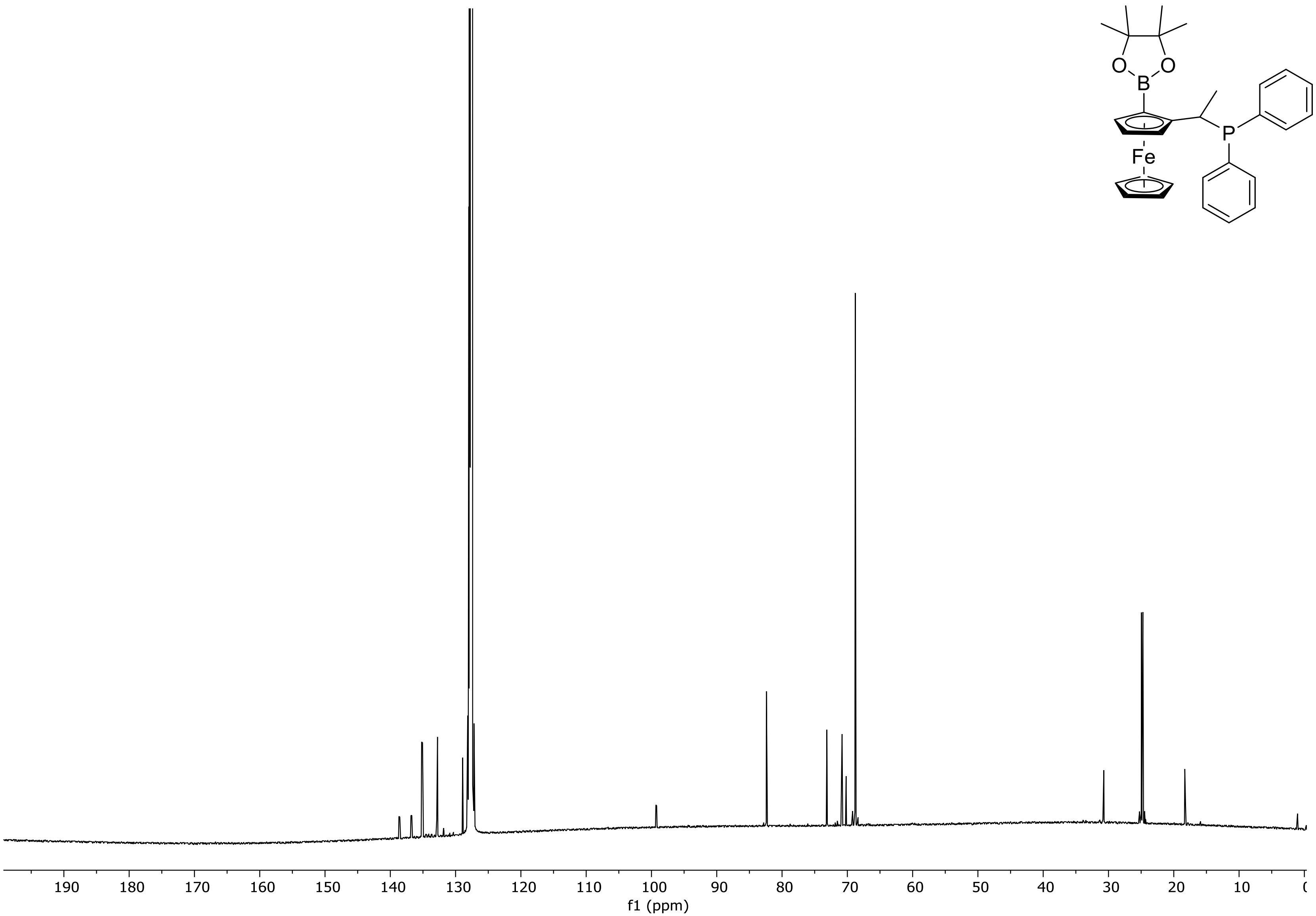

^{31}P NMR of borylated [1-(Diphenylphosphino)ethyl]ferrocene (16)

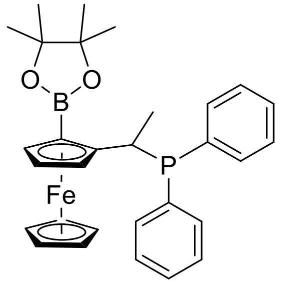

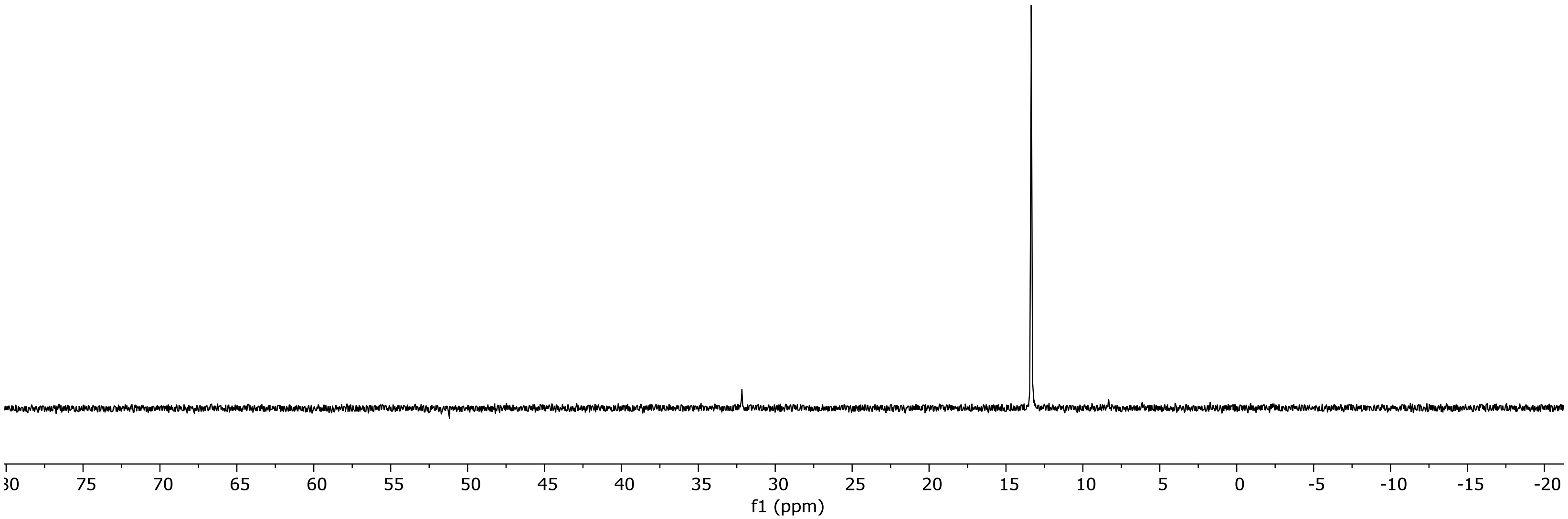

^{1}H NMR of 2-(dicyclohexylphosphaneyl)-2',4',6'-triisopropyl-[1,1'-biphenyl]-4-ol (19)

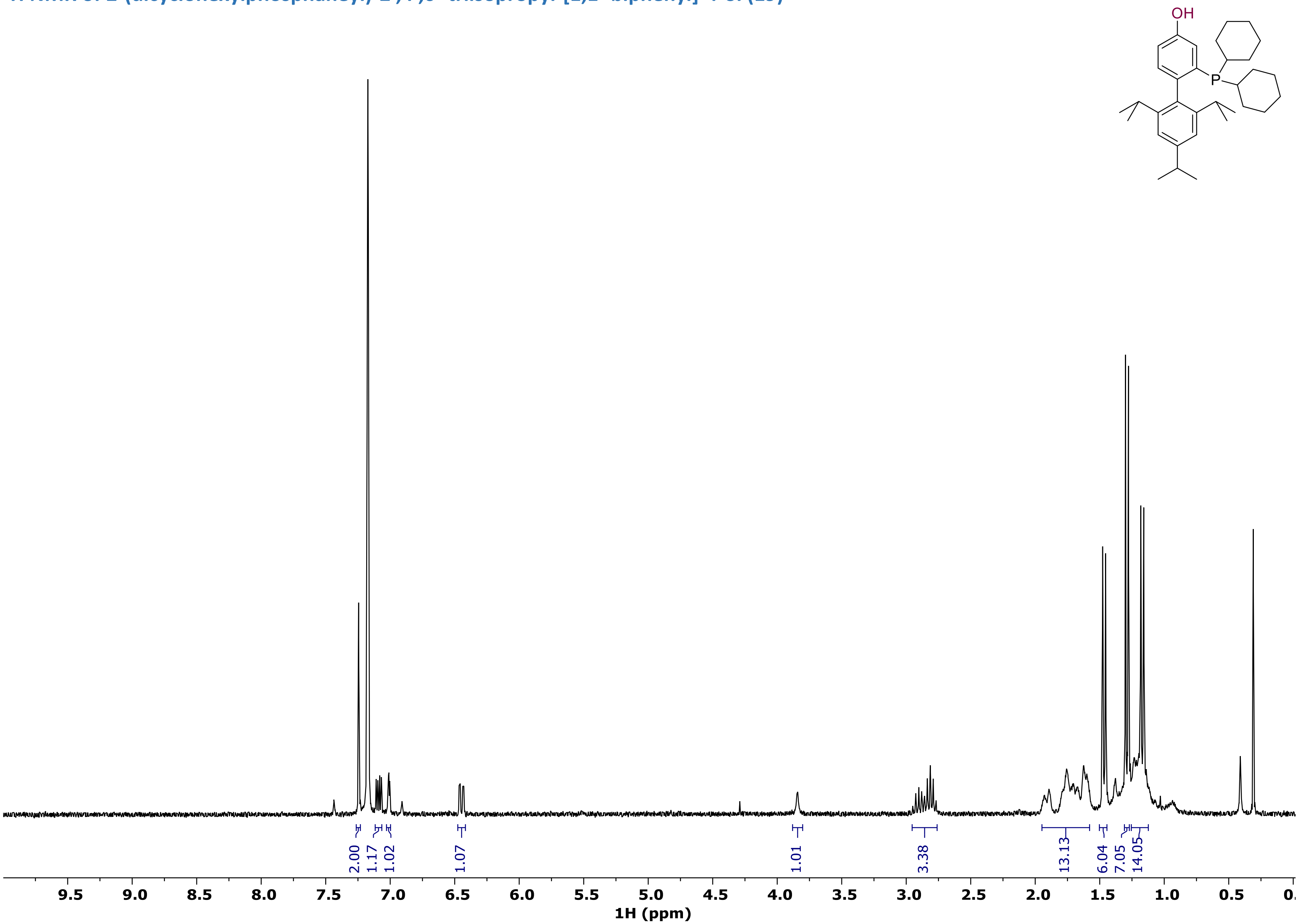

^{13}C NMR of 2-(dicyclohexylphosphaneyl)-2',4',6'-triisopropyl-[1,1'-biphenyl]-4-ol (19)

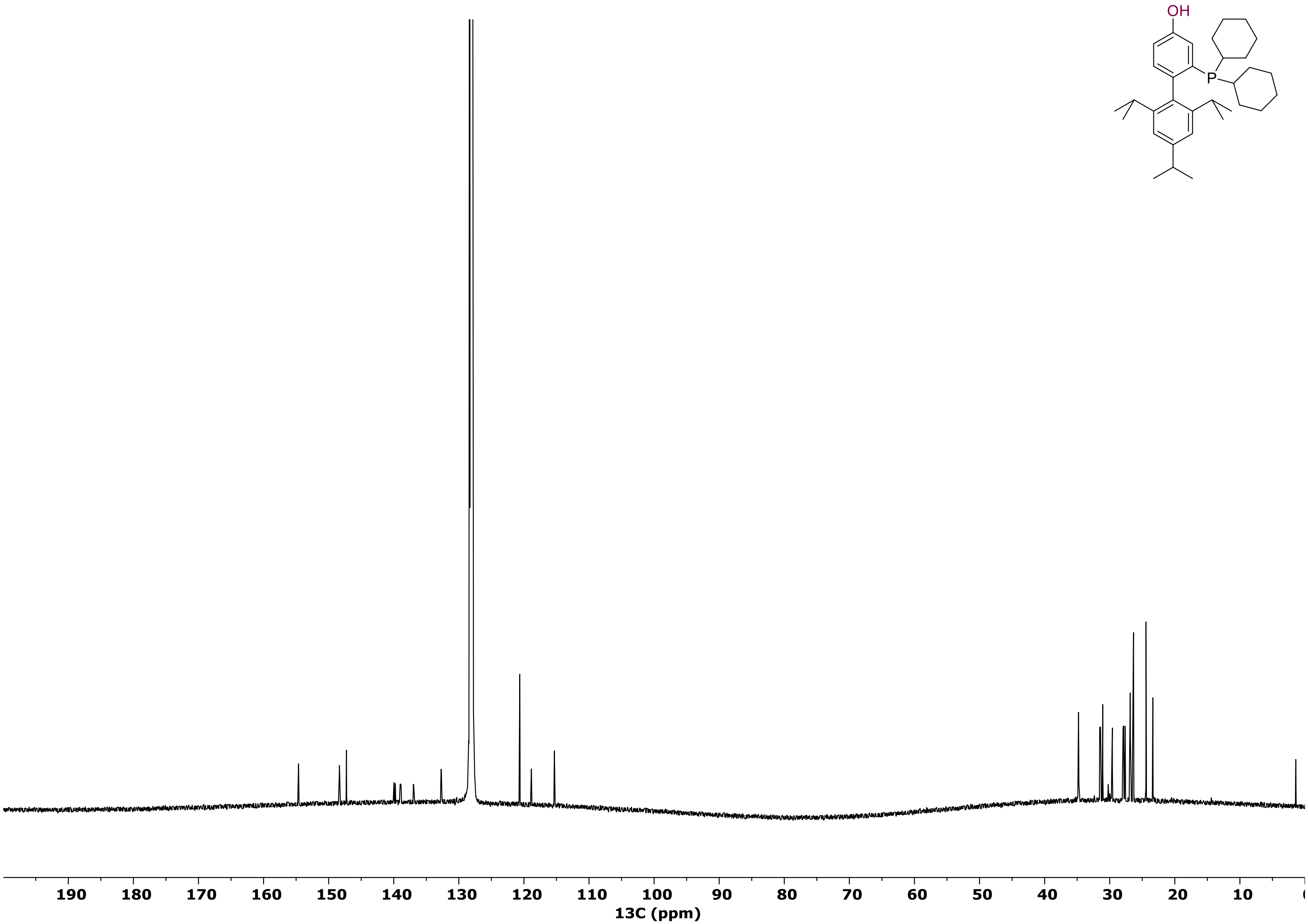

^{31}P NMR of 2-(dicyclohexylphosphaneyl)-2',4',6'-triisopropyl-[1,1'-biphenyl]-4-ol (19)

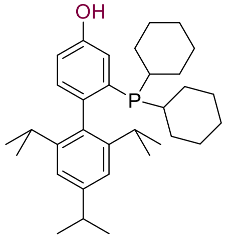

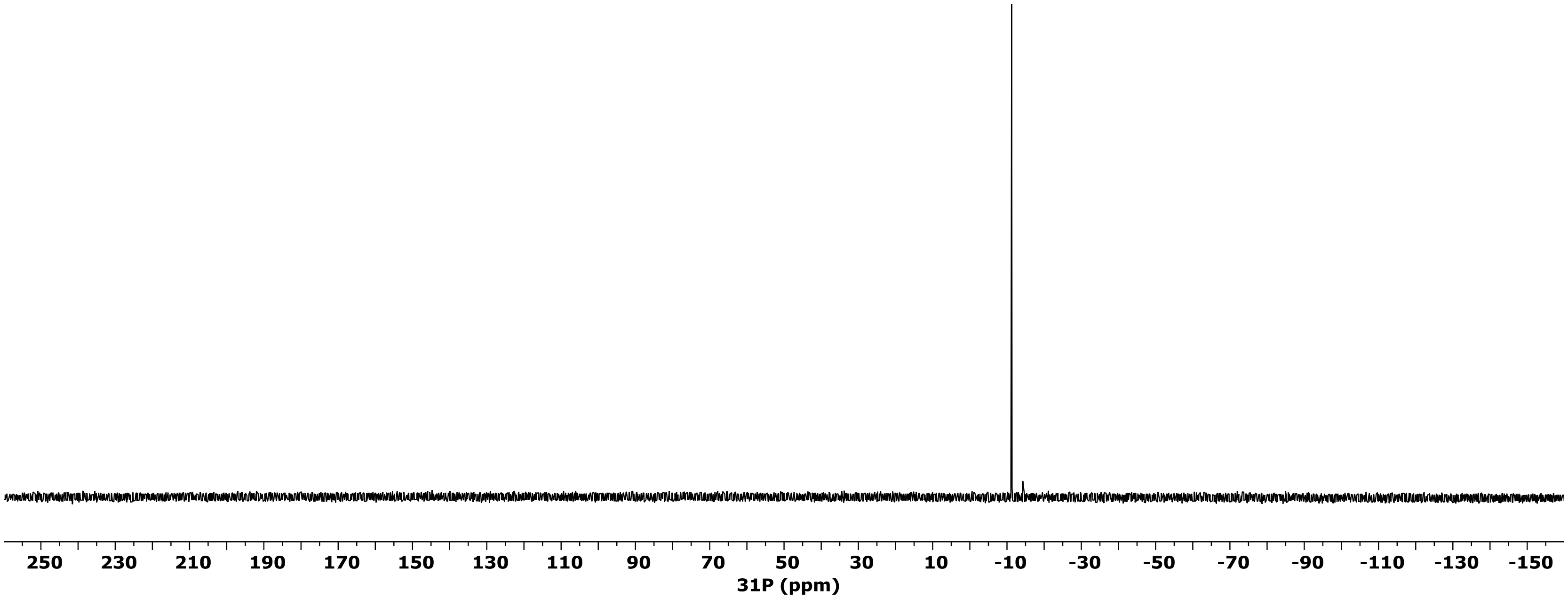

^{1}H NMR of dicyclohexyl(2',4',6'-triisopropyl-4-methoxy-[1,1'-biphenyl]-2-yl)phosphane (20)

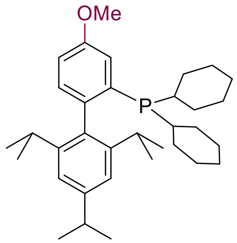

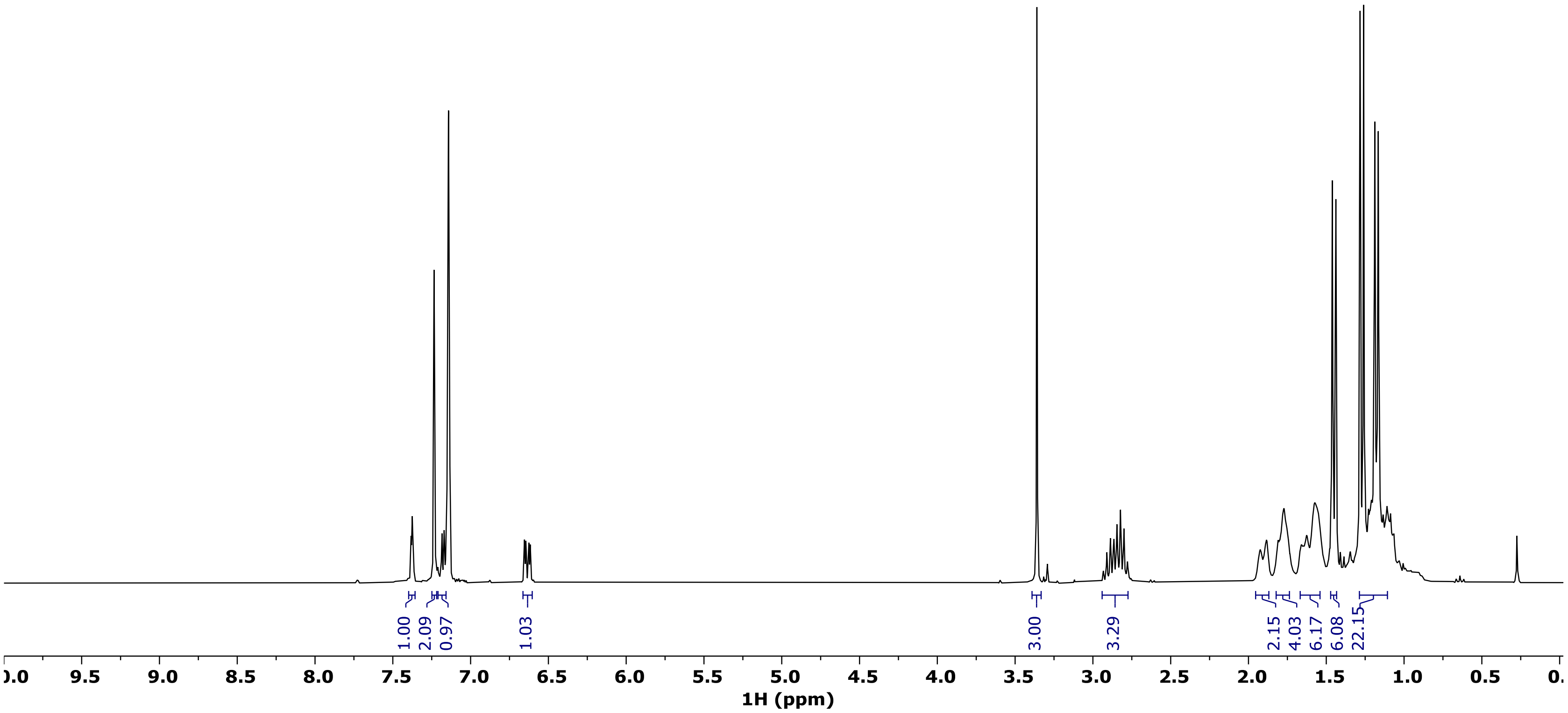

^{13}C NMR of dicyclohexyl(2',4',6'-triisopropyl-4-methoxy-[1,1'-biphenyl]-2-yl)phosphane (20)

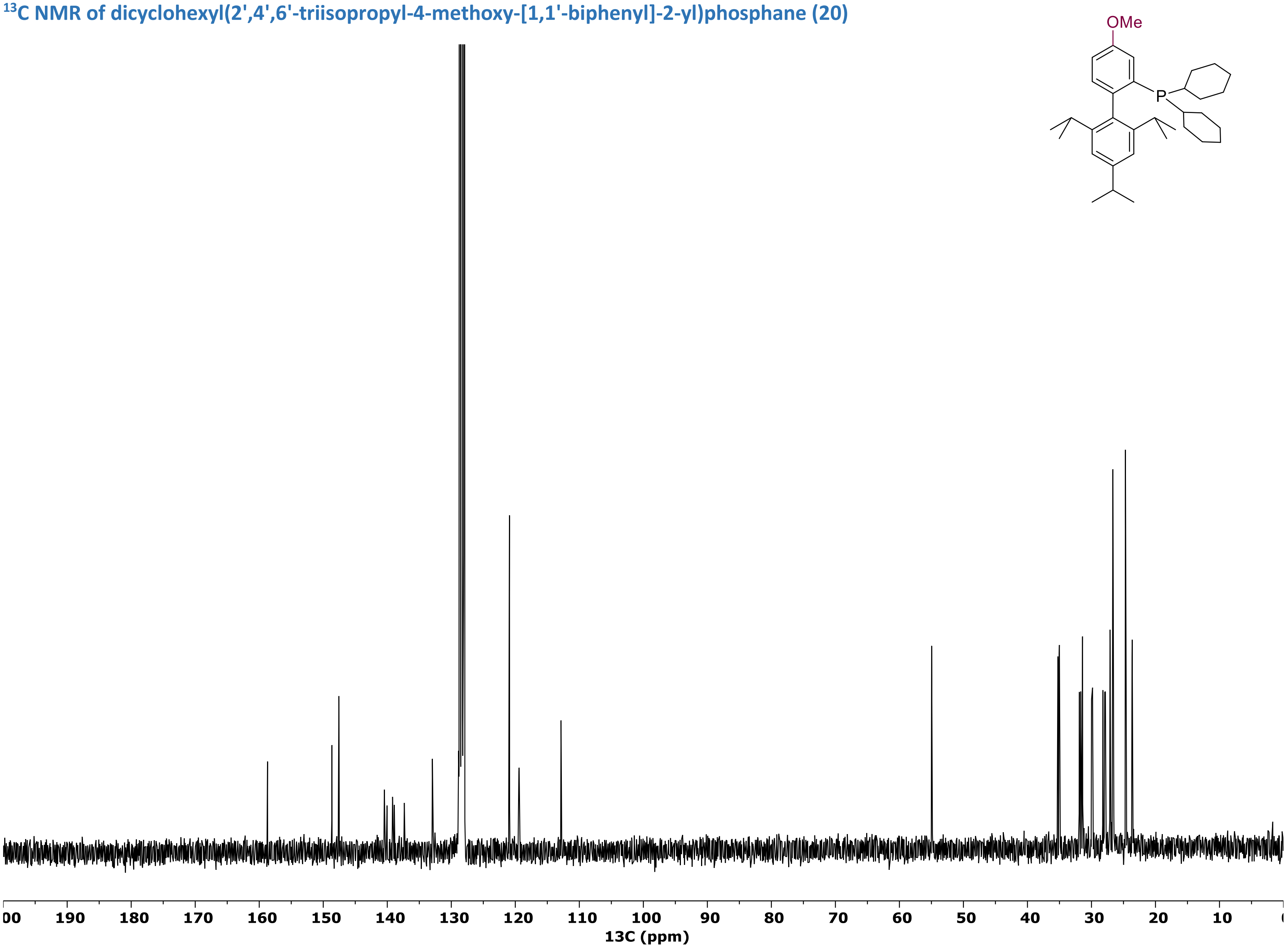

^{31}P NMR of dicyclohexyl(2',4',6'-triisopropyl-4-methoxy-[1,1'-biphenyl]-2-yl)phosphane (20)

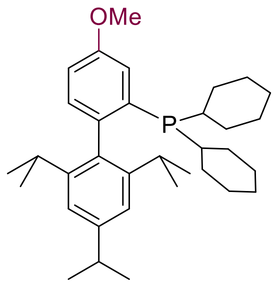

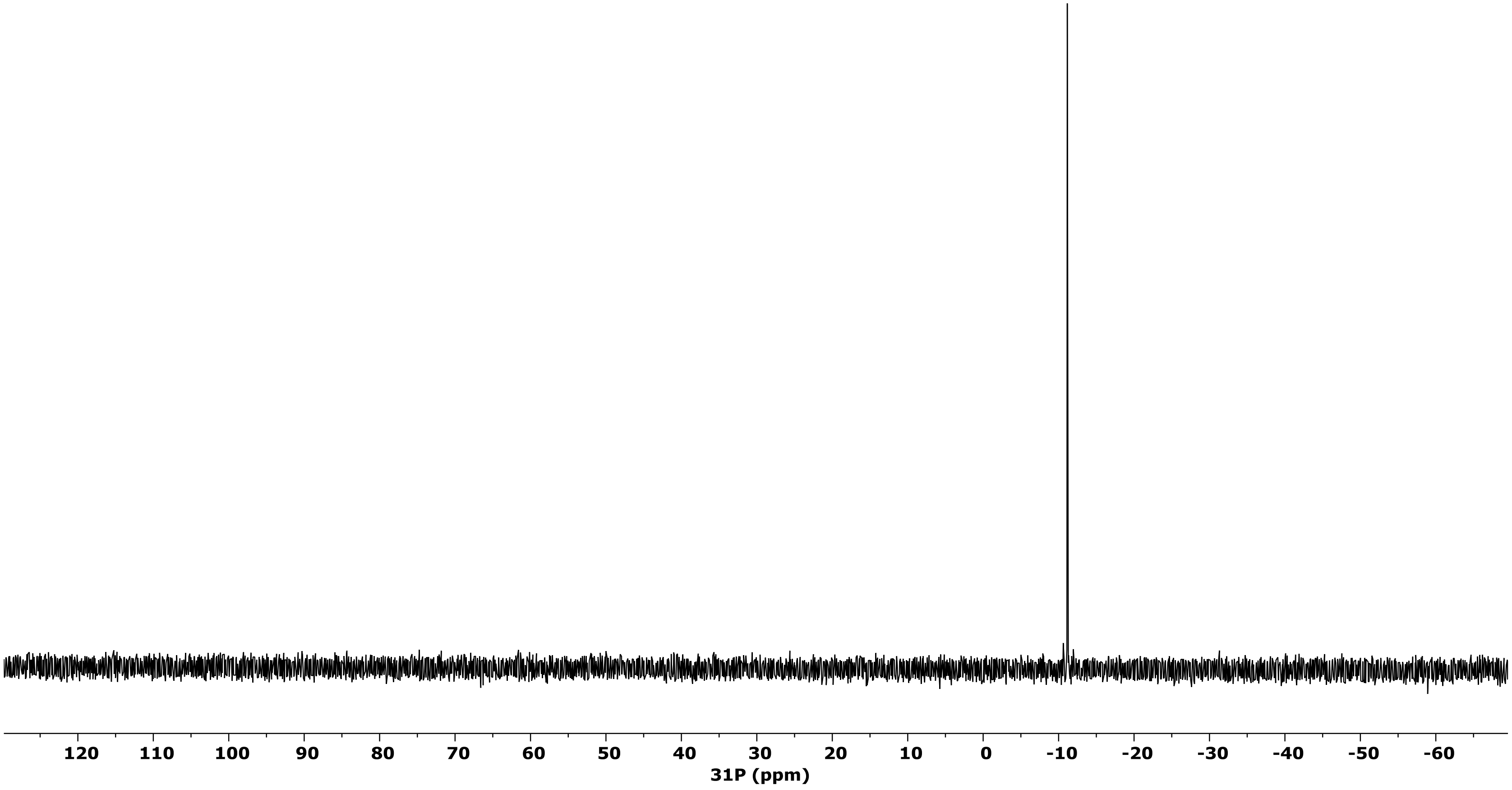

^{1}H NMR of 3'-(dicyclohexylphosphaneyl)-2'',4'',6''-triisopropyl-N,N-dimethyl-[1,1':4',1''-terphenyl]-3-amine (22)

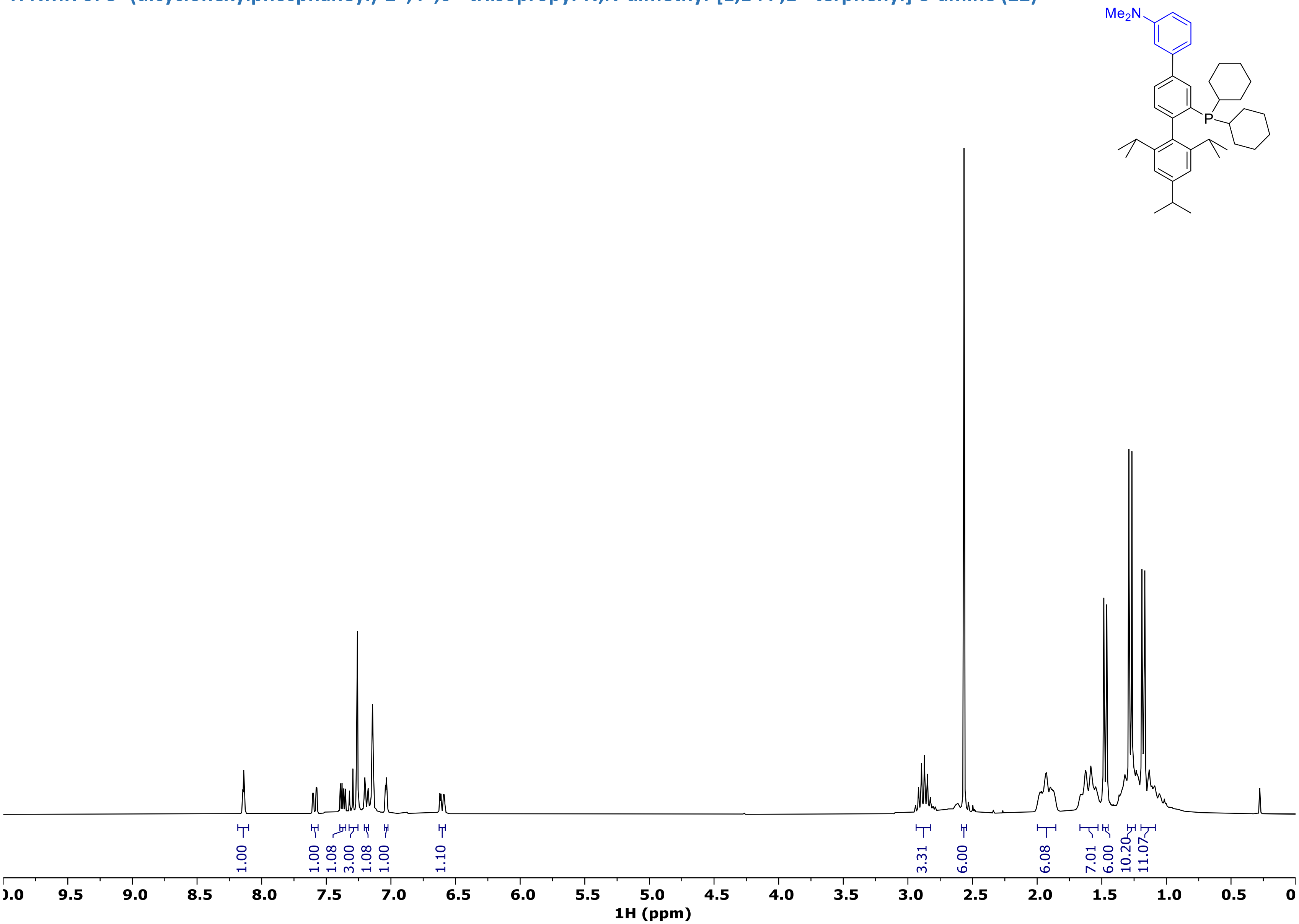

^{13}C NMR of 3'-(dicyclohexylphosphaneyl)-2'',4'',6''-triisopropyl-N,N-dimethyl-[1,1':4',1''-terphenyl]-3-amine (22)

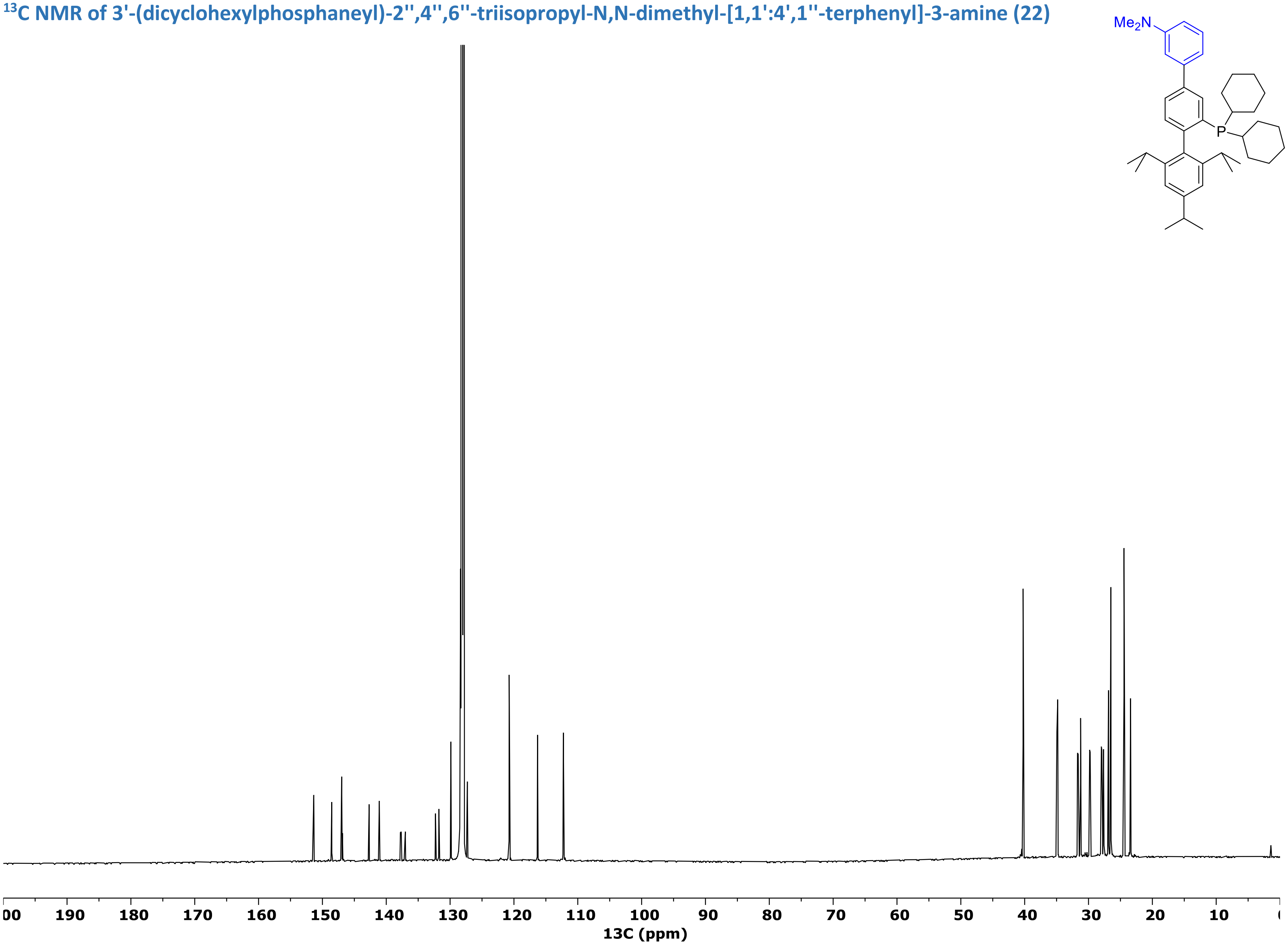

^{31}P NMR of 3'-(dicyclohexylphosphaneyl)-2'',4'',6''-triisopropyl-N,N-dimethyl-[1,1':4',1''-terphenyl]-3-amine (22)

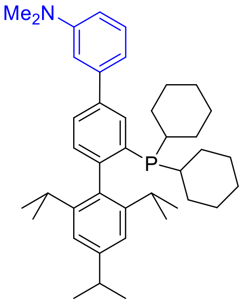

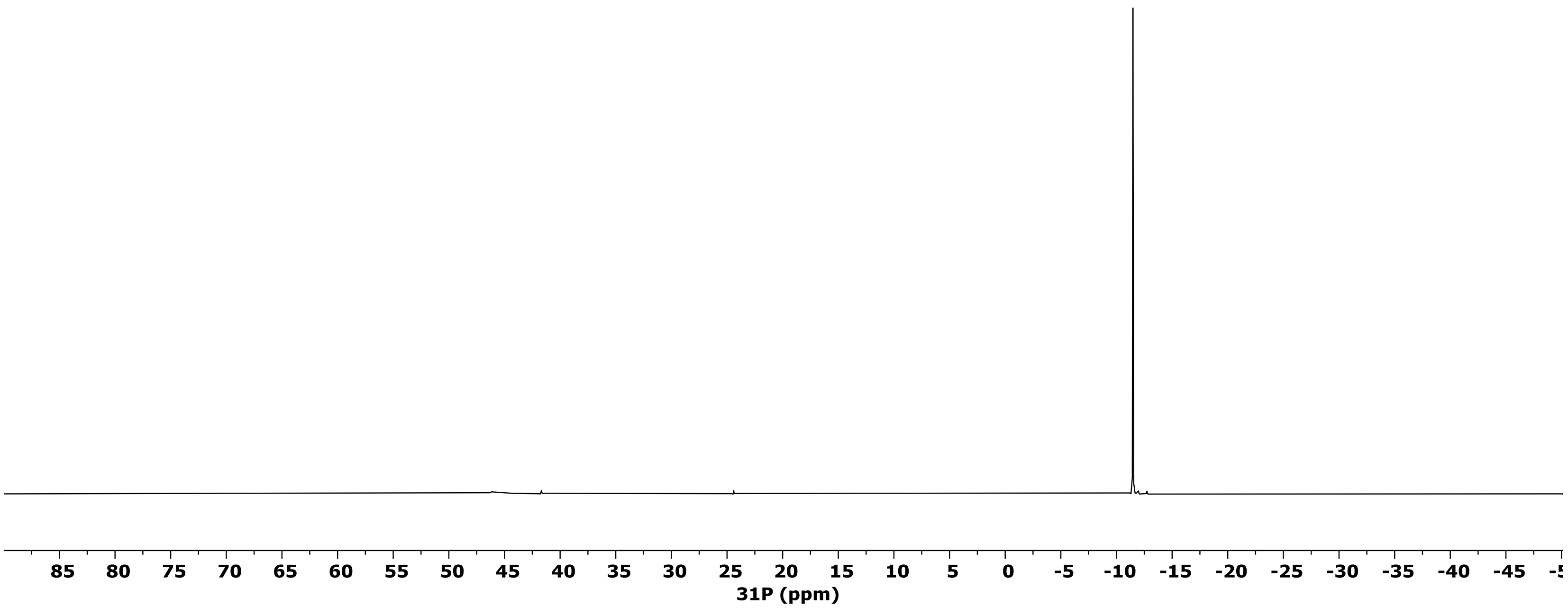

^{1}H NMR of 3-(3'-(dicyclohexylphosphaneyl)-2'',4'',6''-triisopropyl-[1,1':4',1''-terphenyl]-3-yl)-5-ethyl-1,2,4-oxadiazole (23)

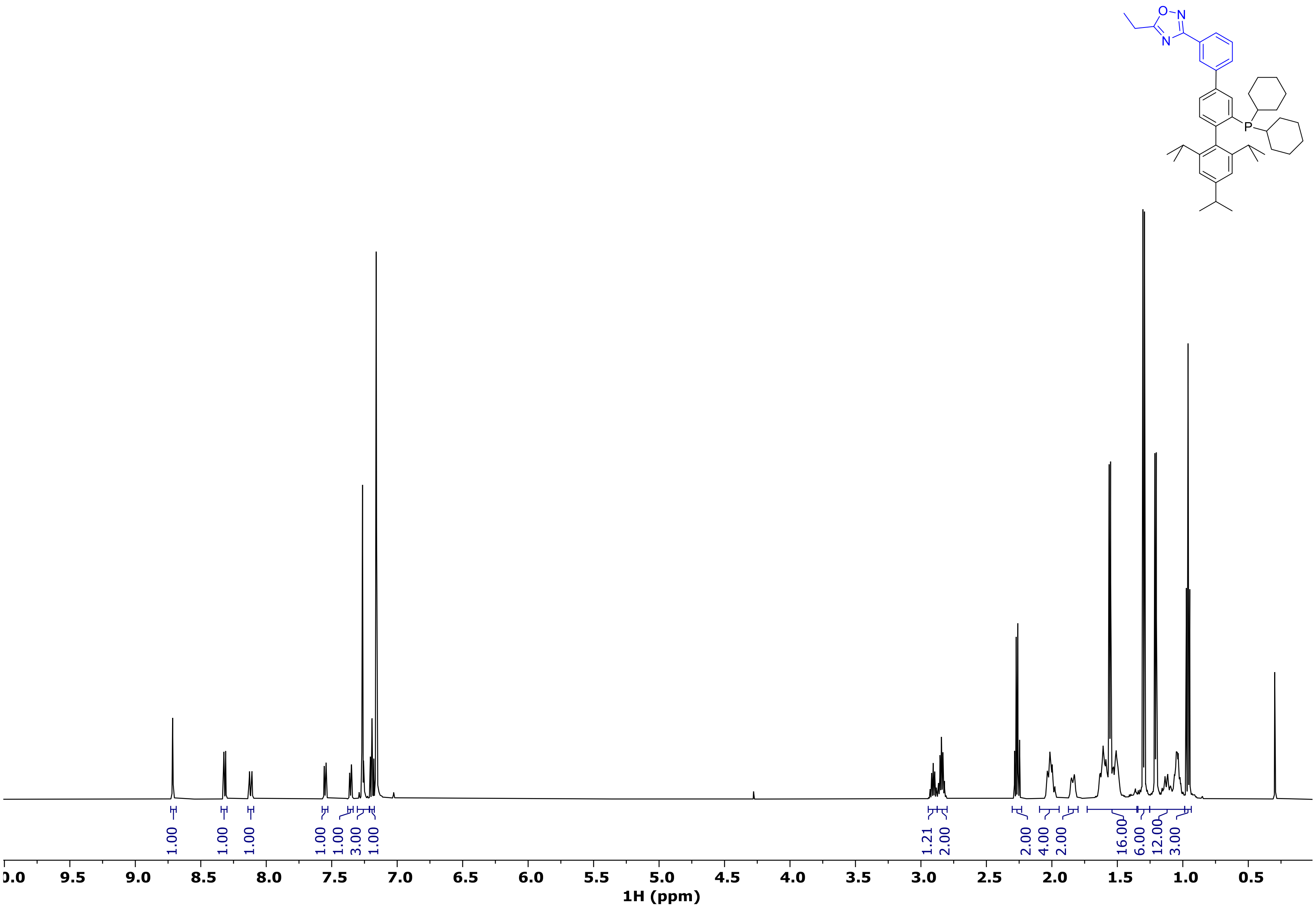

^{13}C NMR of 3-(3'-(dicyclohexylphosphaneyl)-2'',4'',6''-triisopropyl-[1,1':4',1''-terphenyl]-3-yl)-5-ethyl-1,2,4-oxadiazole (23)

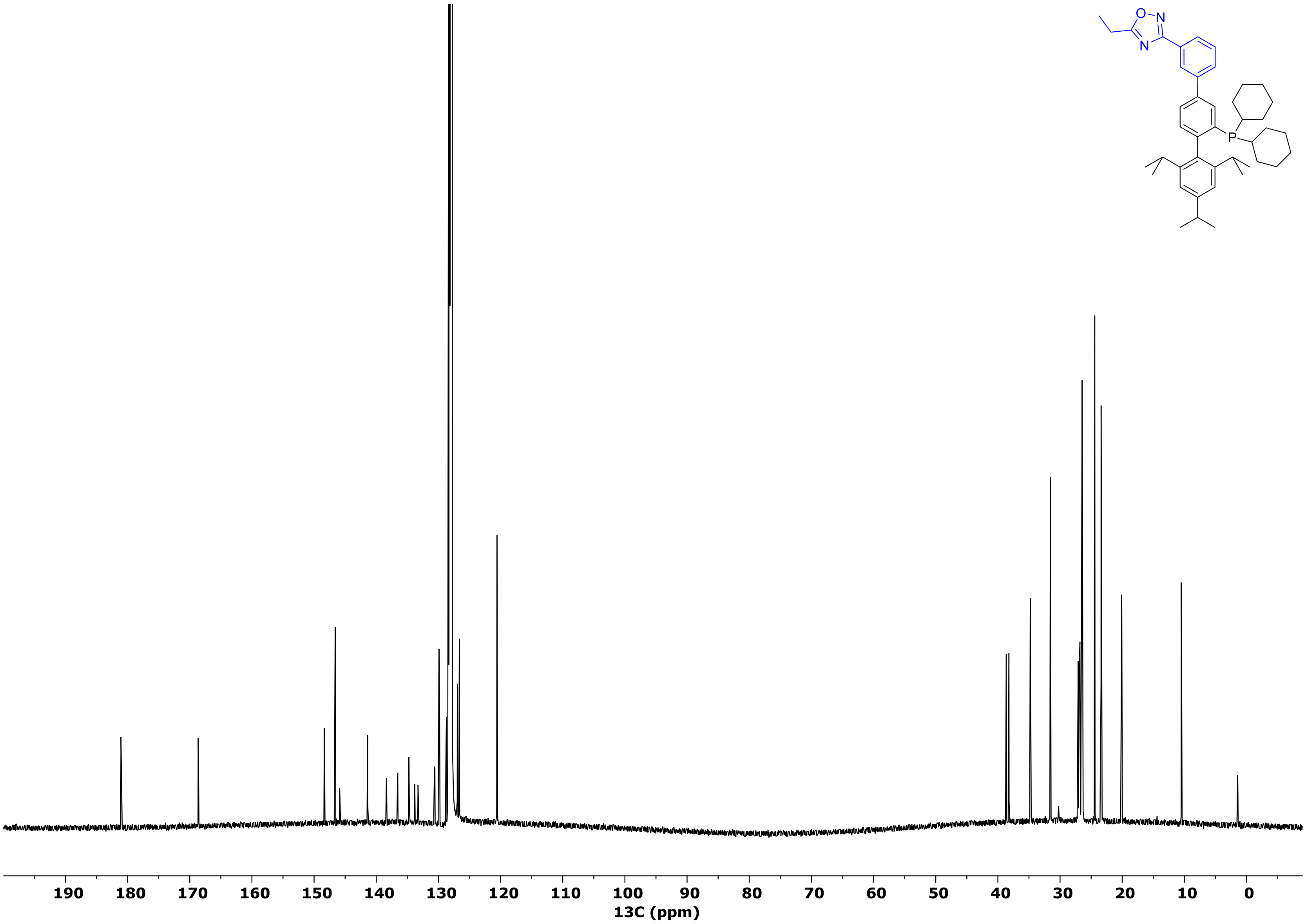

^{31}P NMR of 3-(3'-(dicyclohexylphosphaneyl)-2'',4'',6''-triisopropyl-[1,1':4',1''-terphenyl]-3-yl)-5-ethyl-1,2,4-oxadiazole (23)

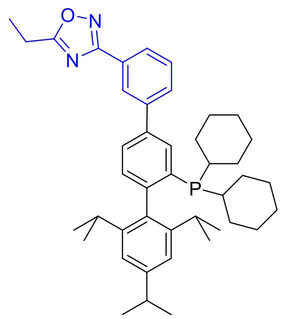

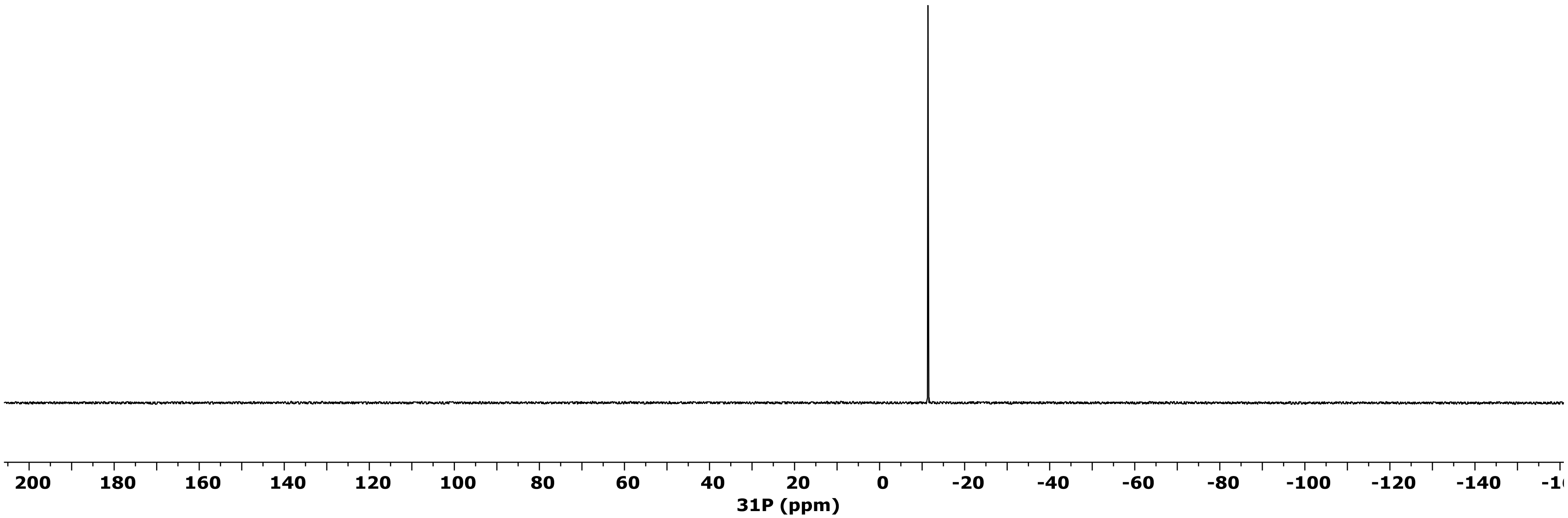

^{1}H NMR of 8-(2-(dicyclohexylphosphaneyl)-2',4',6'-triisopropyl-[1,1'-biphenyl]-4-yl)quinolone (24)

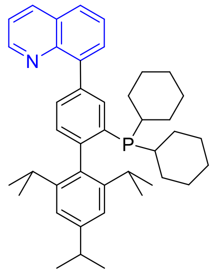

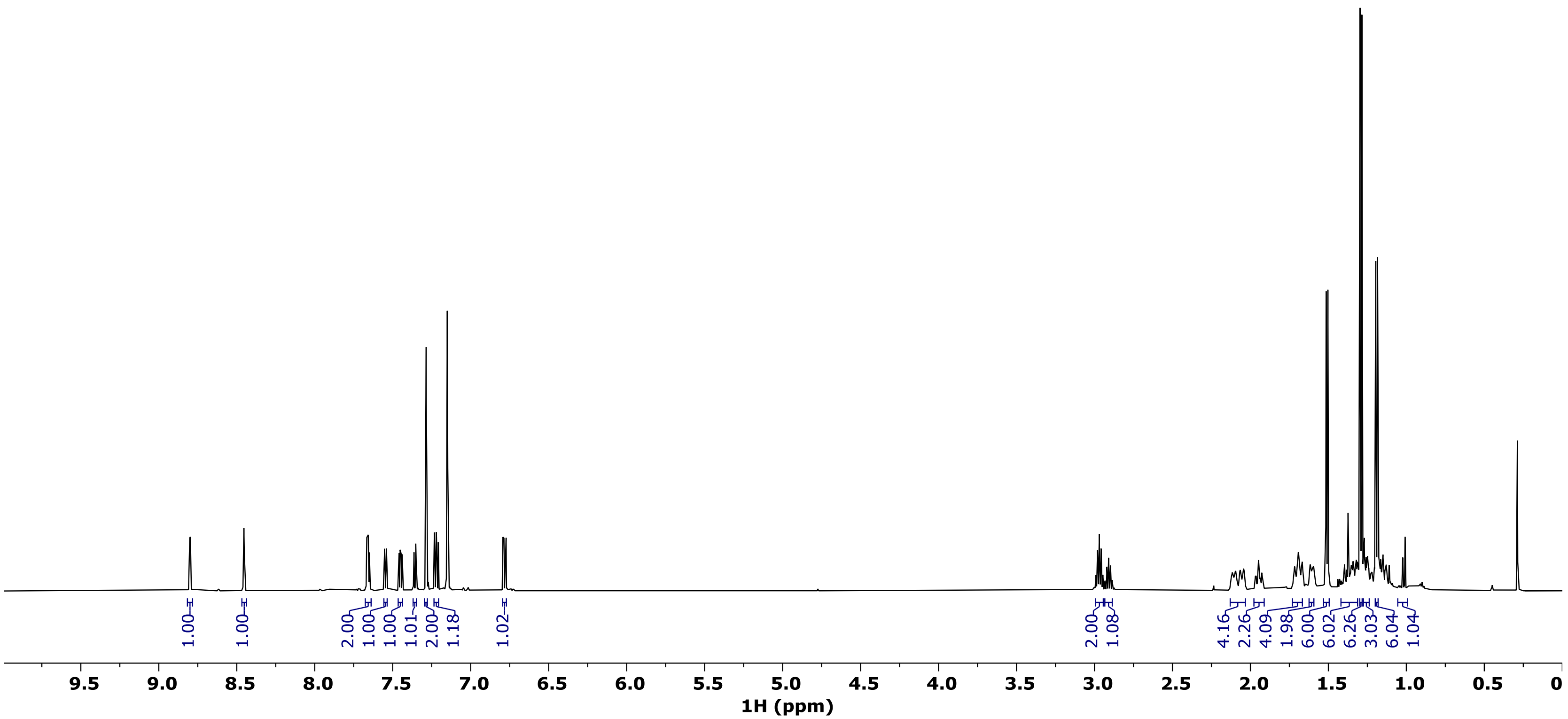

^{13}C NMR of 8-(2-(dicyclohexylphosphaneyl)-2',4',6'-triisopropyl-[1,1'-biphenyl]-4-yl)quinolone (24)

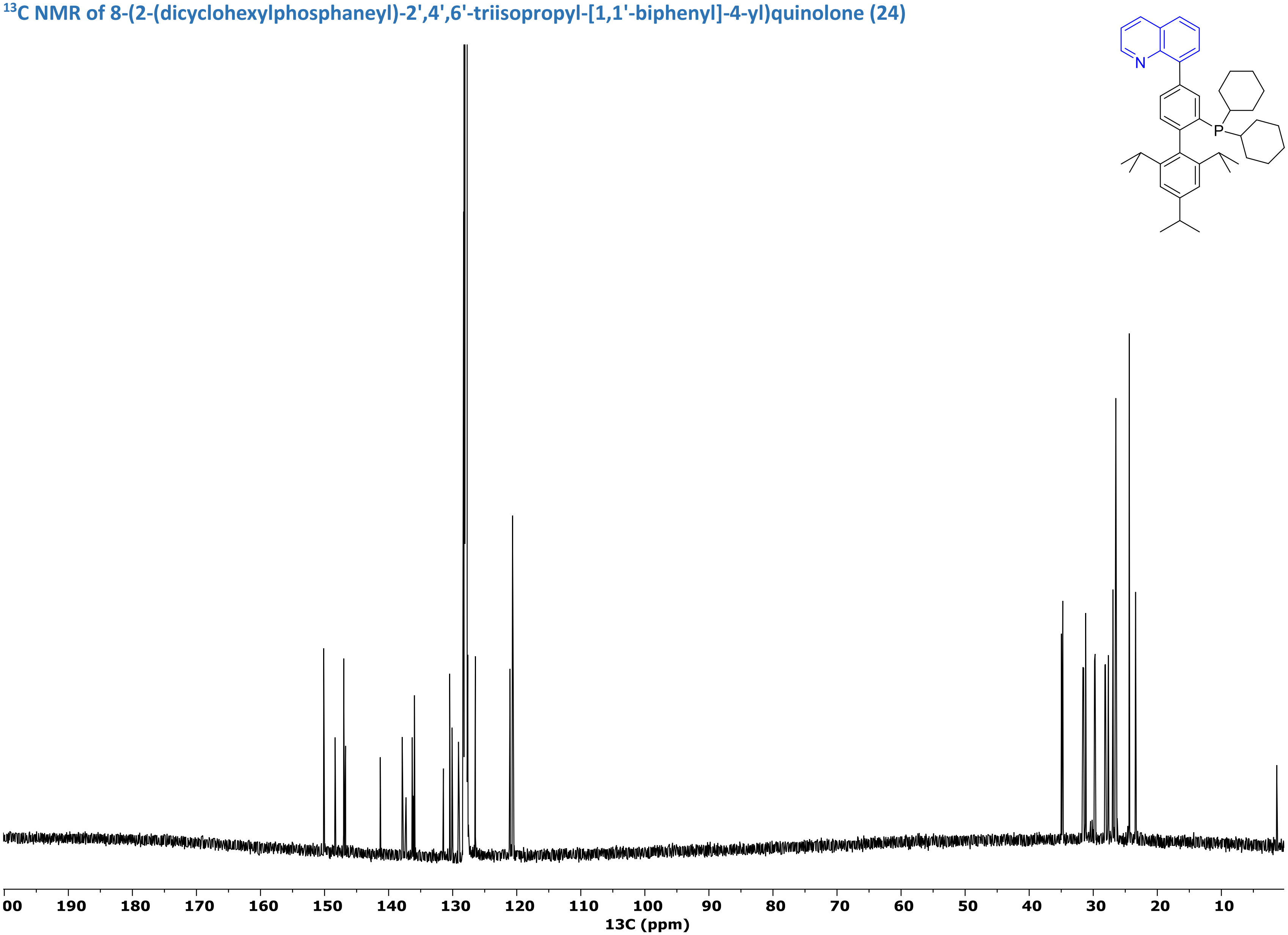

^{31}P NMR of 8-(2-(dicyclohexylphosphaneyl)-2',4',6'-triisopropyl-[1,1'-biphenyl]-4-yl)quinolone (24)

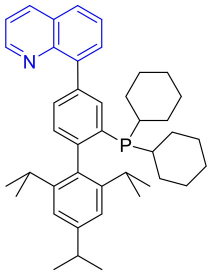

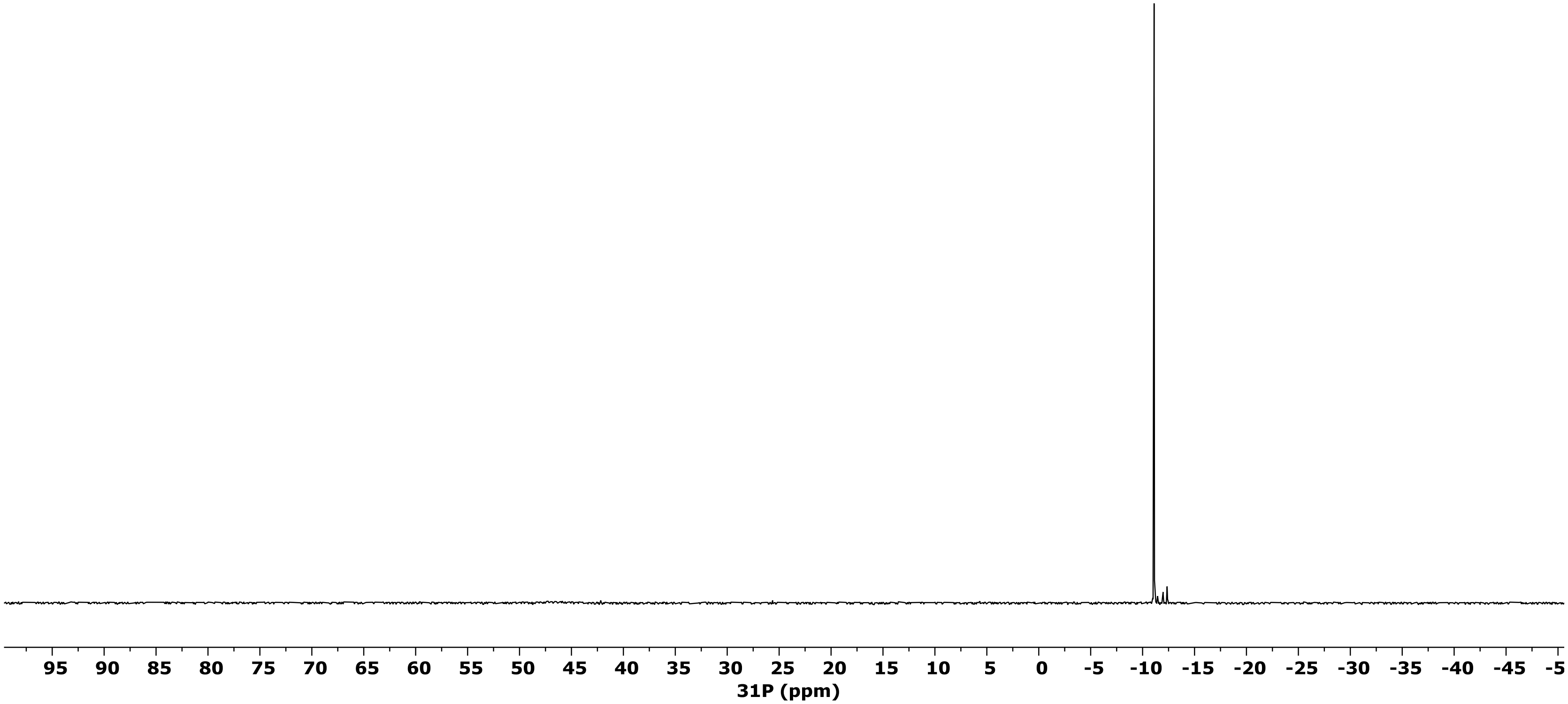

^{1}H NMR of dicyclohexyl(2'',3'',4'',5'',6''-pentafluoro-2,4,6-triisopropyl-[1,1':4',1''-terphenyl]-2'-yl)phosphane (25)

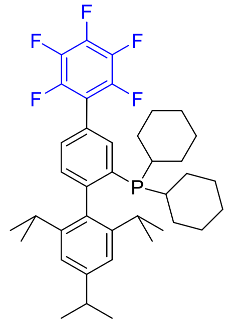

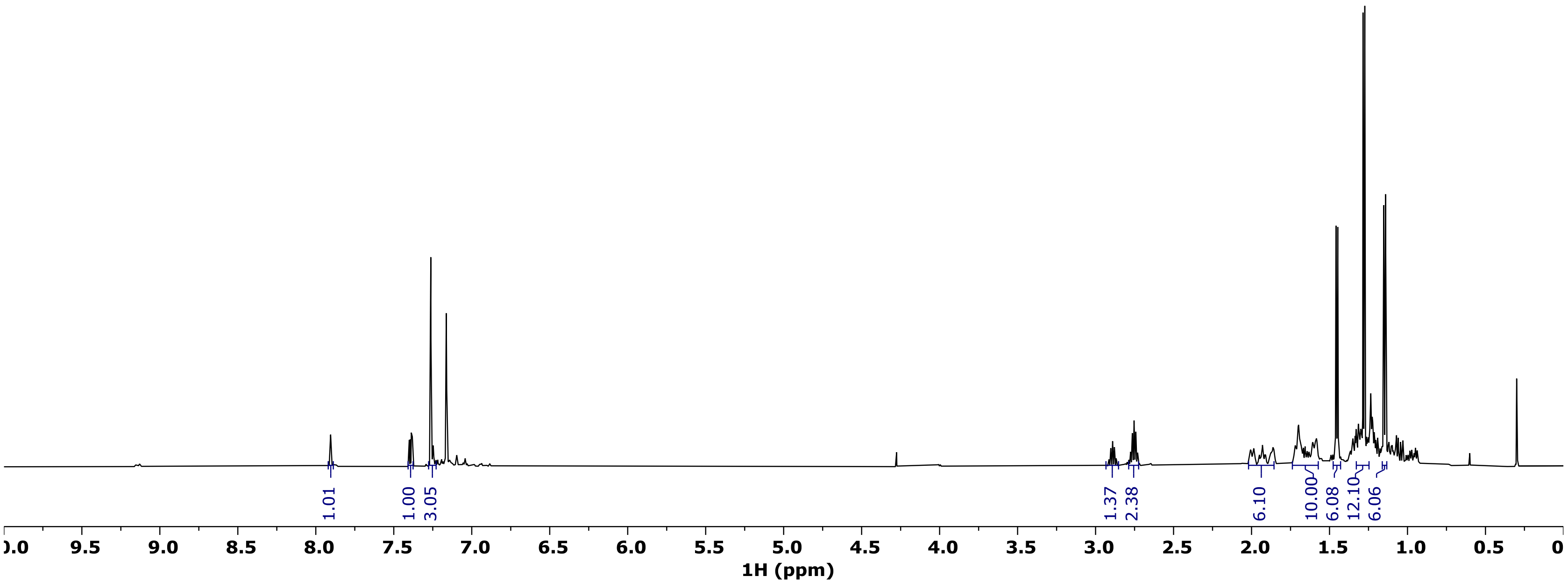

^{13}C NMR of dicyclohexyl(2'',3'',4'',5'',6''-pentafluoro-2,4,6-triisopropyl-[1,1':4',1''-terphenyl]-2'-yl)phosphane (25)

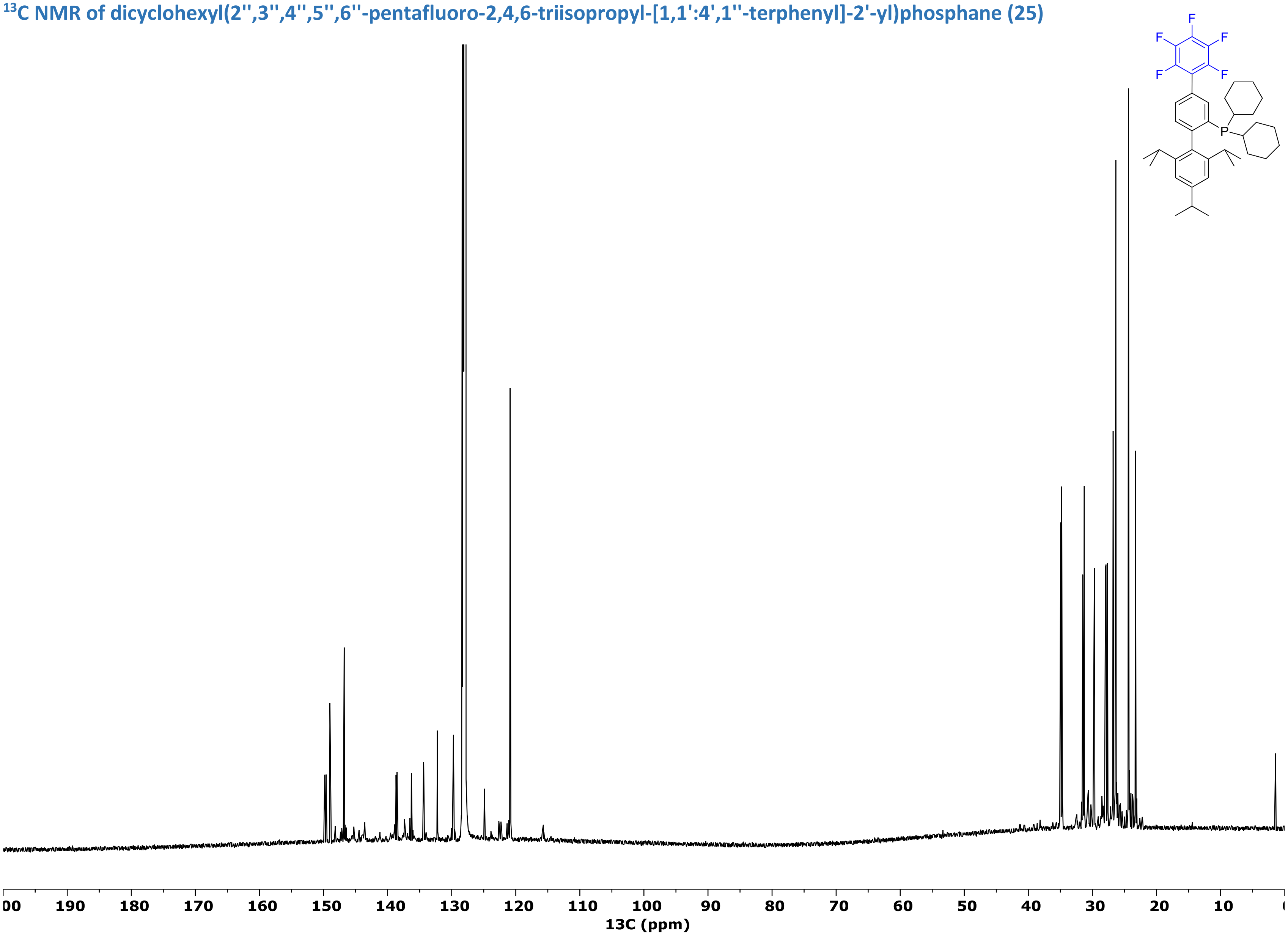

^{31}P NMR of dicyclohexyl(2'',3'',4'',5'',6''-pentafluoro-2,4,6-triisopropyl-[1,1':4',1''-terphenyl]-2'-yl)phosphane (25)

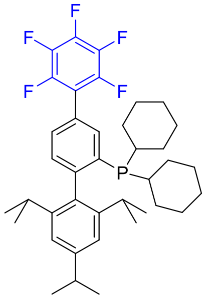

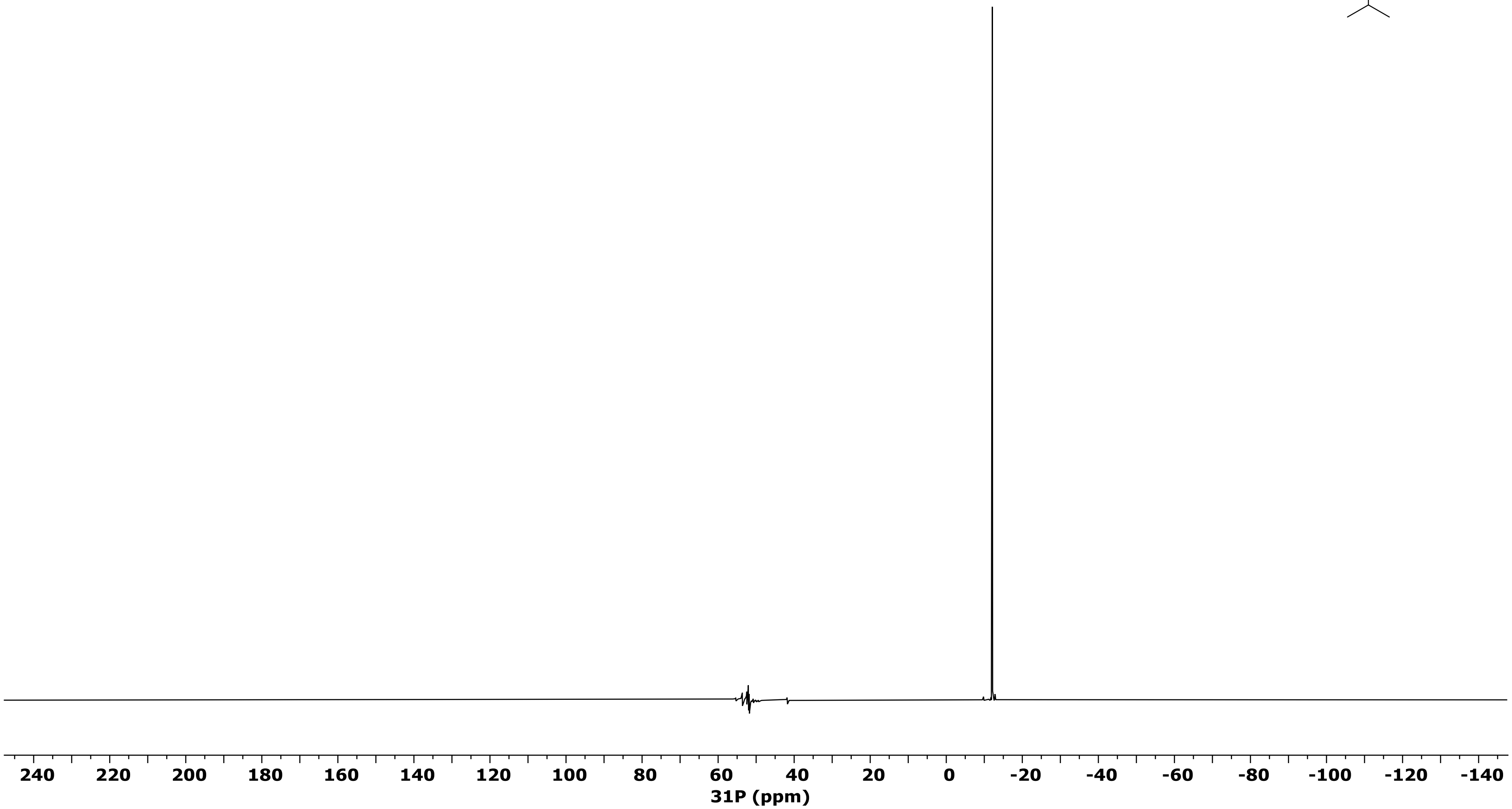

^{19}F NMR of dicyclohexyl(2'',3'',4'',5'',6''-pentafluoro-2,4,6-triisopropyl-[1,1':4',1''-terphenyl]-2'-yl)phosphane (25)

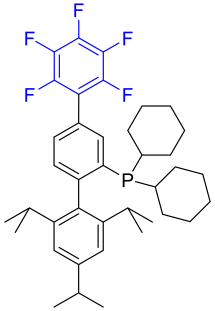

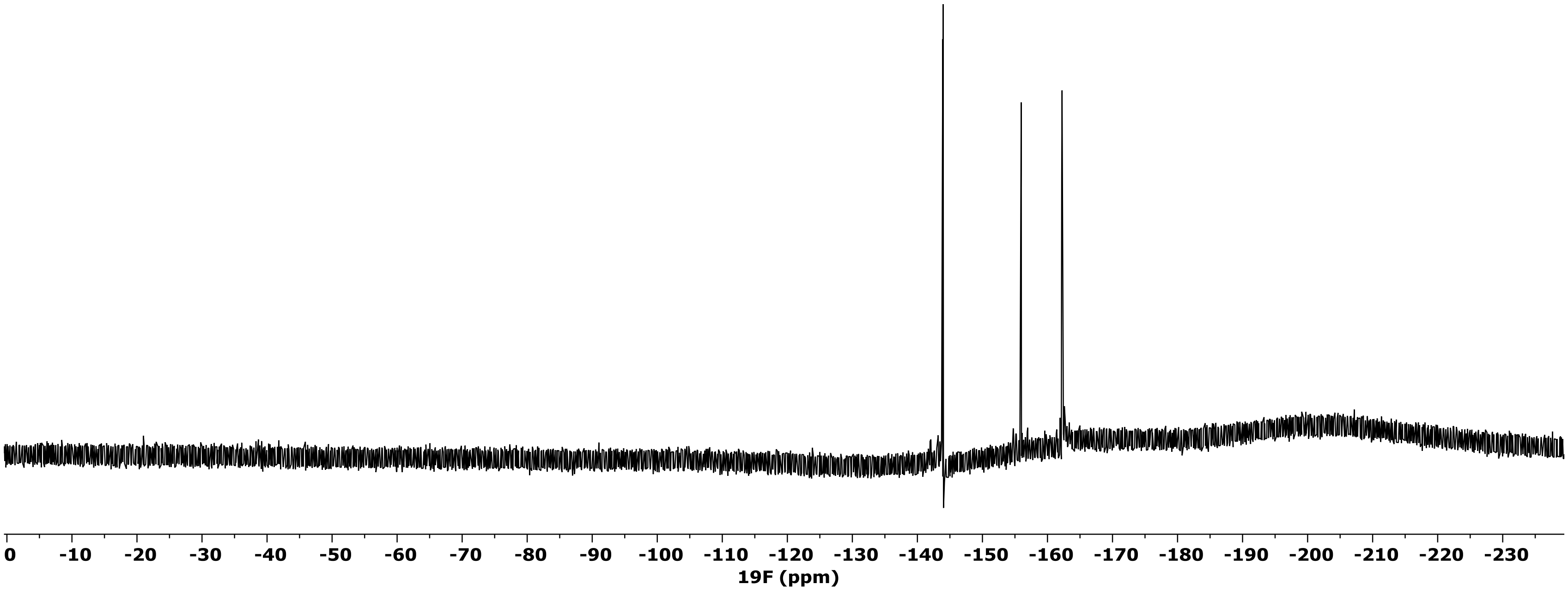

^{1}H NMR of dicyclohexyl(2',4',6'-triisopropyl-4-(thiophen-2-yl)-[1,1'-biphenyl]-2-yl)phosphane (26)

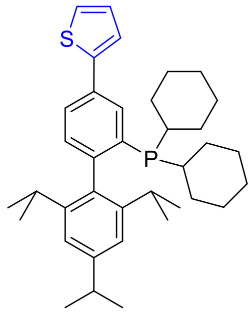

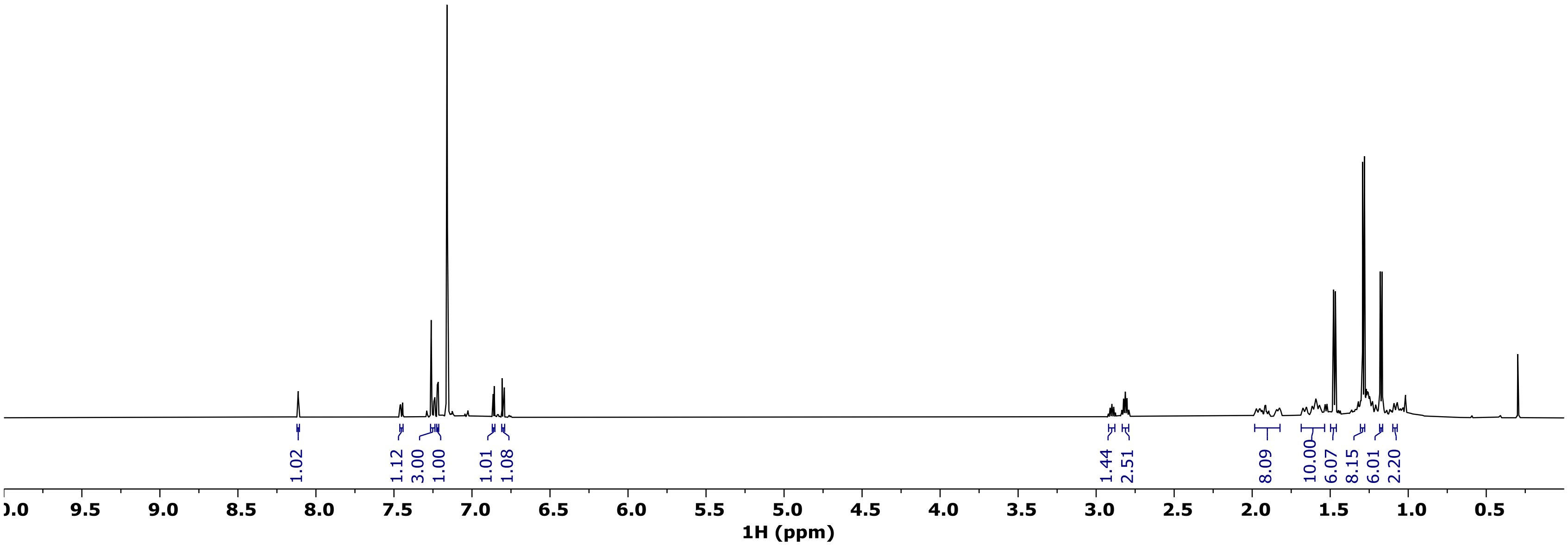

^{13}C NMR of dicyclohexyl(2',4',6'-triisopropyl-4-(thiophen-2-yl)-[1,1'-biphenyl]-2-yl)phosphane (26)

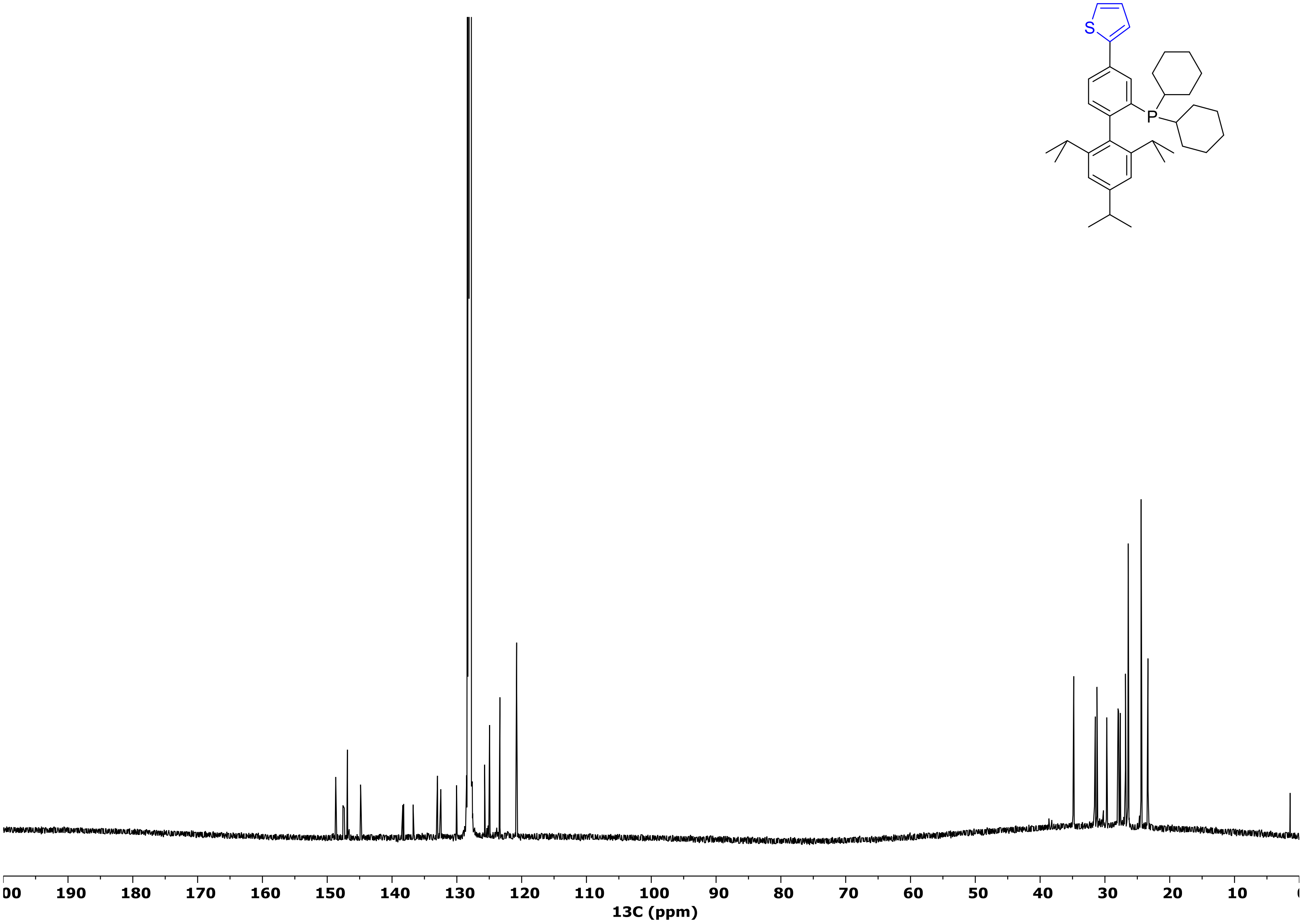

^{31}P NMR of dicyclohexyl(2',4',6'-triisopropyl-4-(thiophen-2-yl)-[1,1'-biphenyl]-2-yl)phosphane (26)

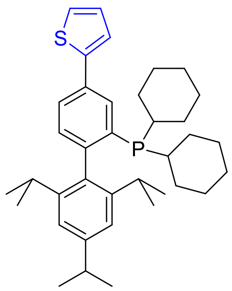

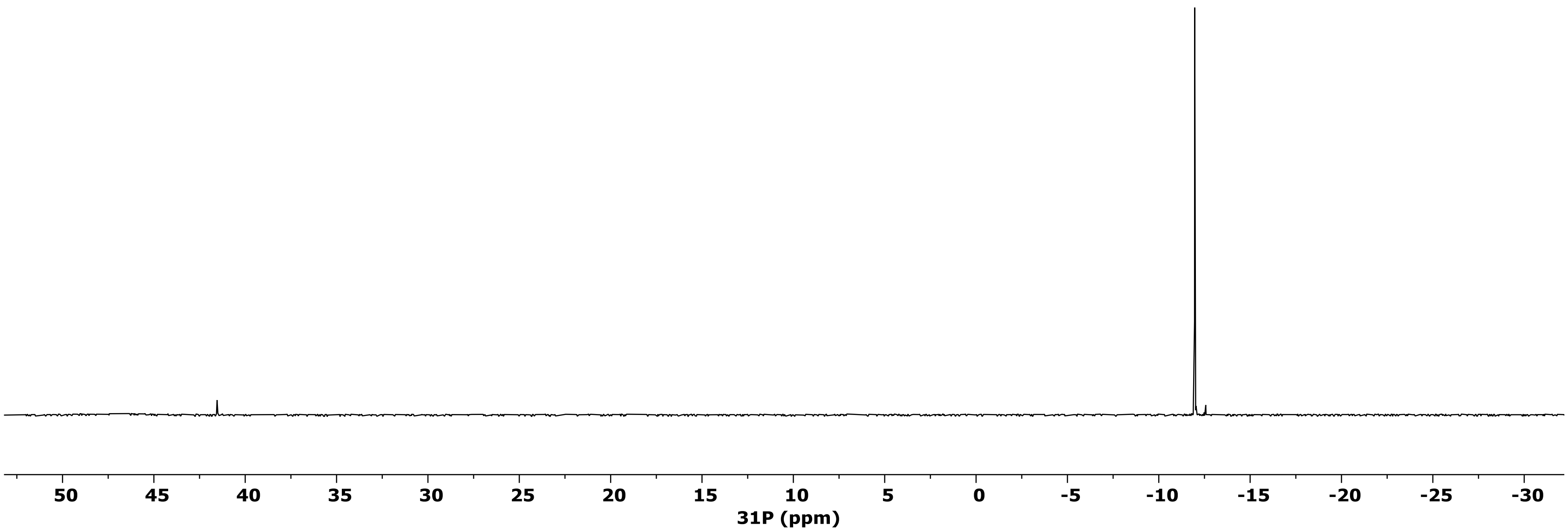

^{1}H NMR of dicyclohexyl(2,4,6-triisopropyl-3'',4''-dimethoxy-[1,1':4',1''-terphenyl]-2'-yl)phosphane (27)

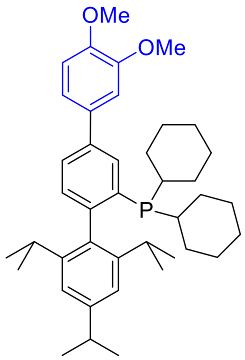

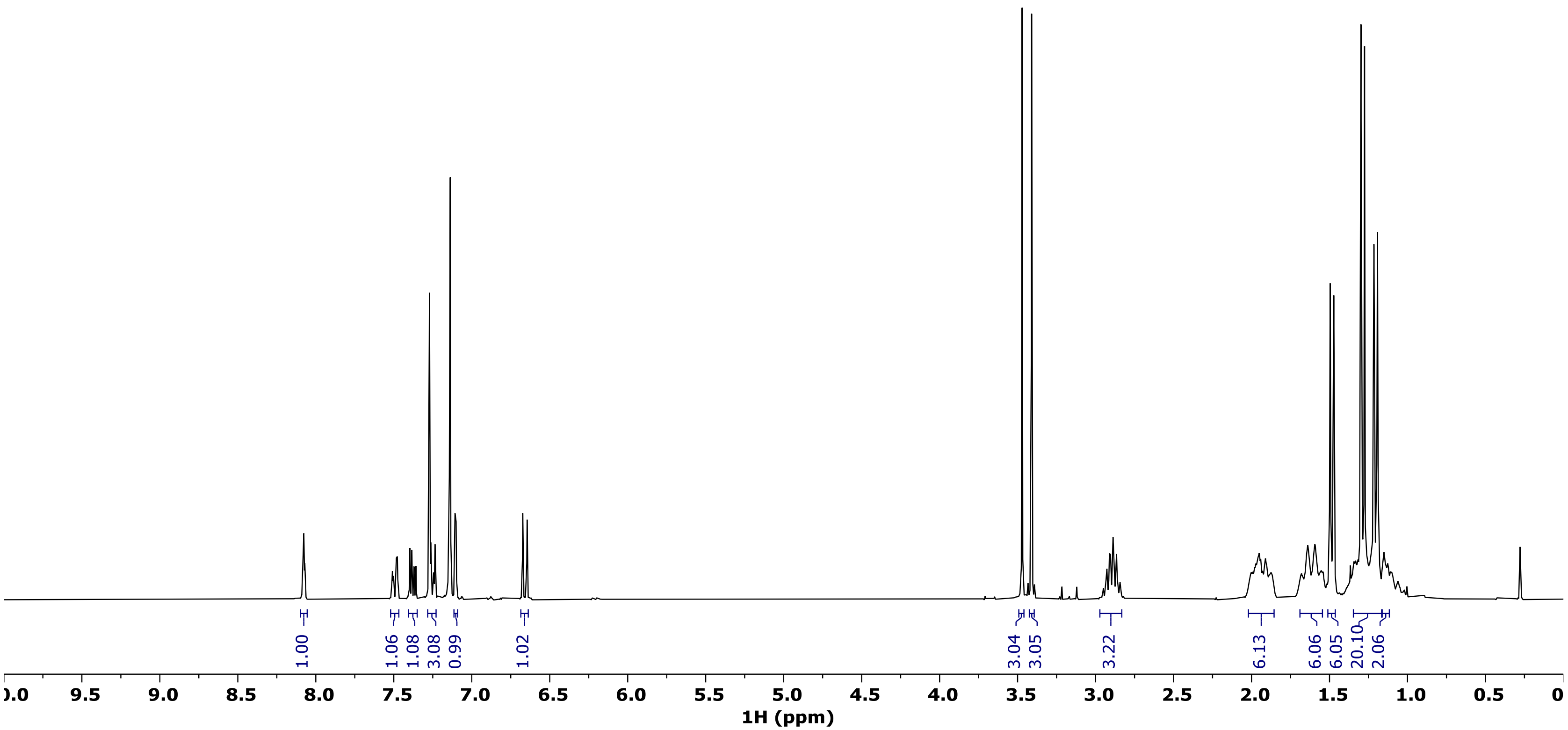

^{13}C NMR of dicyclohexyl(2,4,6-triisopropyl-3'',4''-dimethoxy-[1,1':4',1''-terphenyl]-2'-yl)phosphane (27)

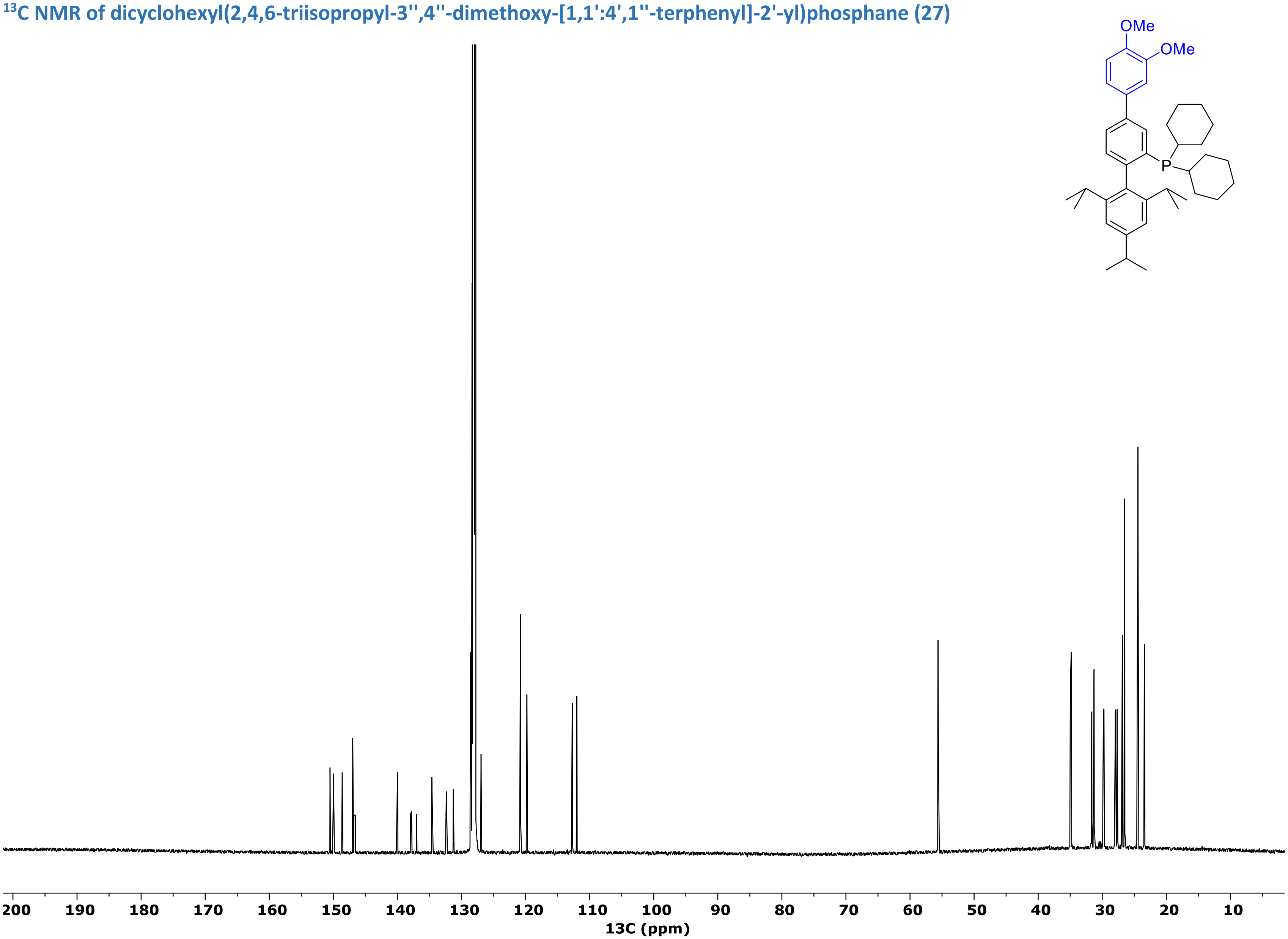

^{31}P NMR of dicyclohexyl(2,4,6-triisopropyl-3'',4''-dimethoxy-[1,1':4',1''-terphenyl]-2'-yl)phosphane (27)

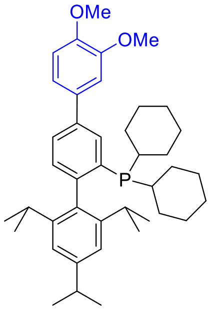

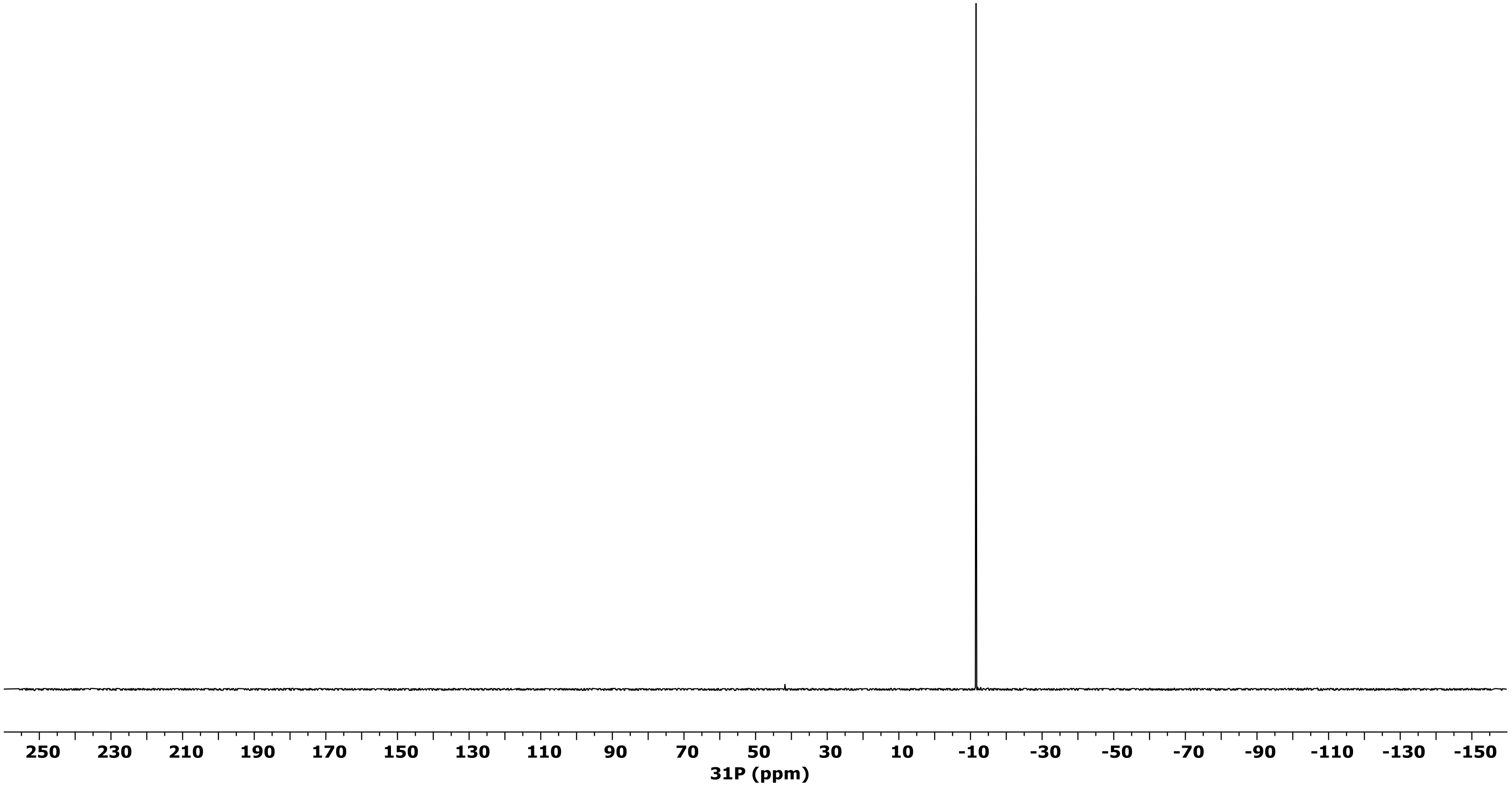

^{1}H NMR of 1,2-bis(3'-(dicyclohexylphosphaneyl)-2'',4'',6''-triisopropyl-[1,1':4',1''-terphenyl]-4-yl)ethyne(28)

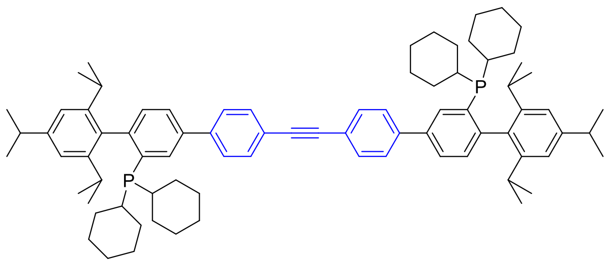

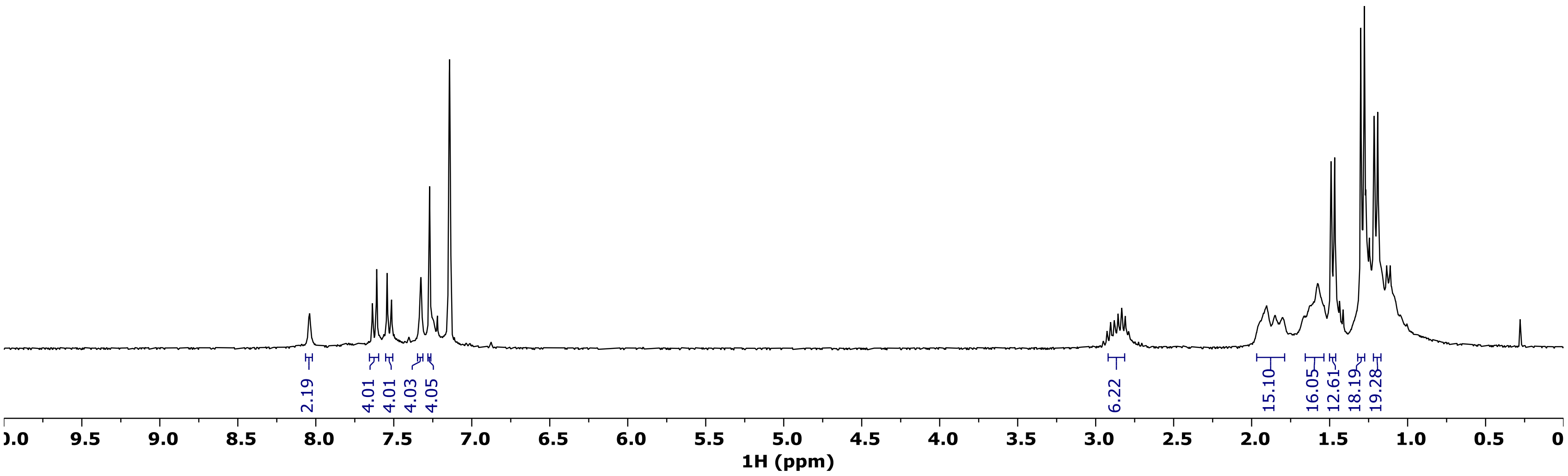

^{13}C NMR of 1,2-bis(3'-(dicyclohexylphosphaneyl)-2'',4'',6''-triisopropyl-[1,1':4',1''-terphenyl]-4-yl)ethyne(28)

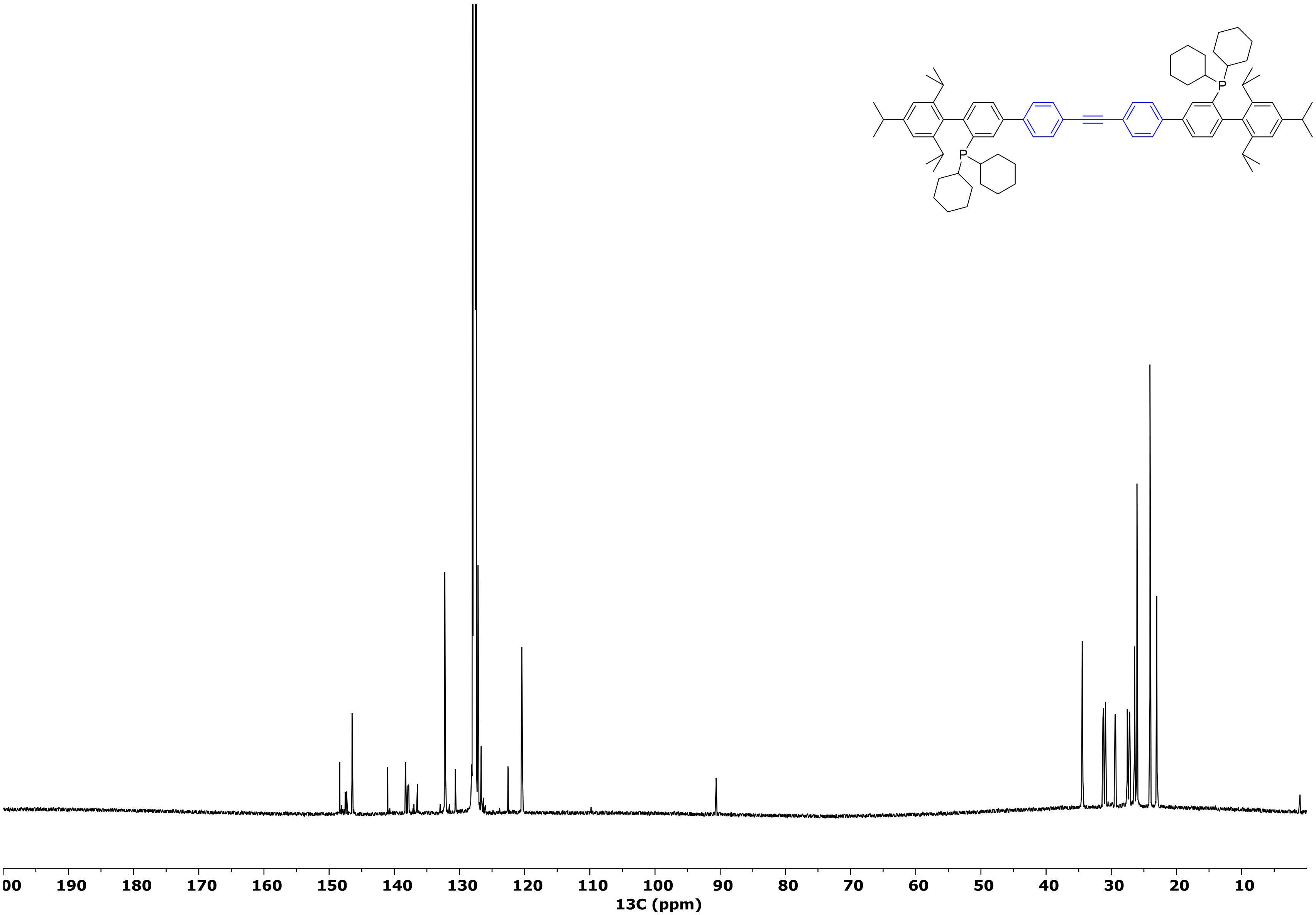

^{31}P NMR of 1,2-bis(3'-(dicyclohexylphosphaneyl)-2'',4'',6''-triisopropyl-[1,1':4',1''-terphenyl]-4-yl)ethyne(28)

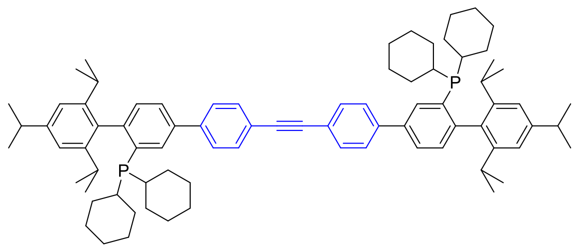

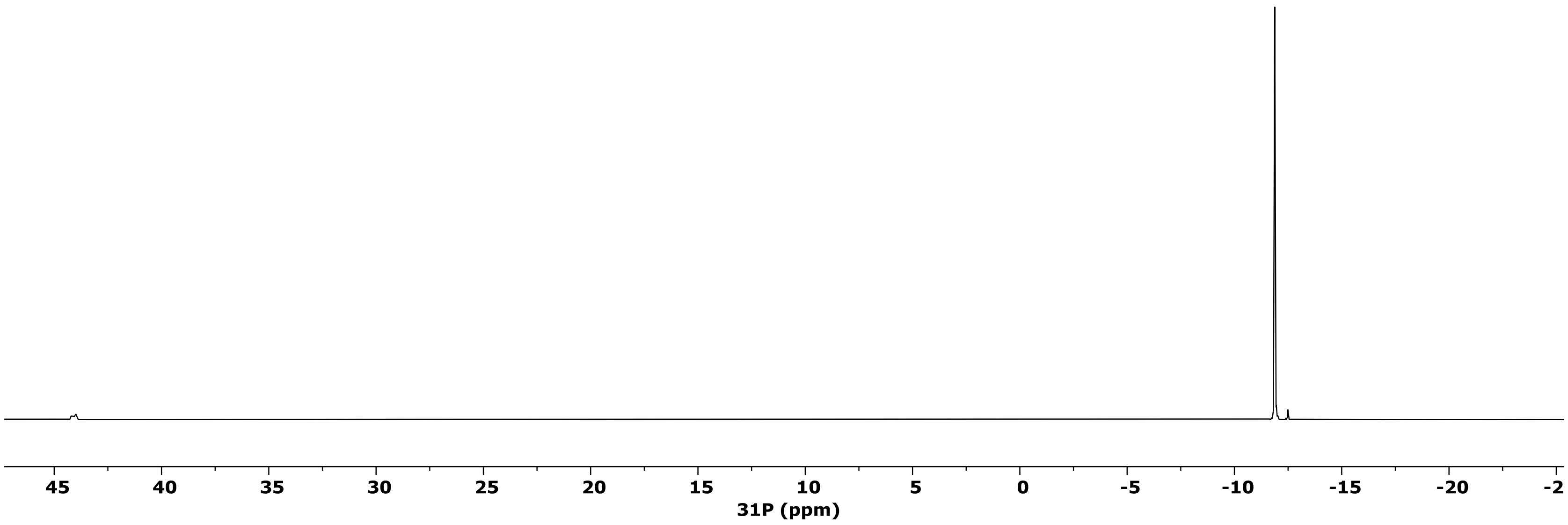

^{1}H NMR of BH_3 protected XPhos (29)

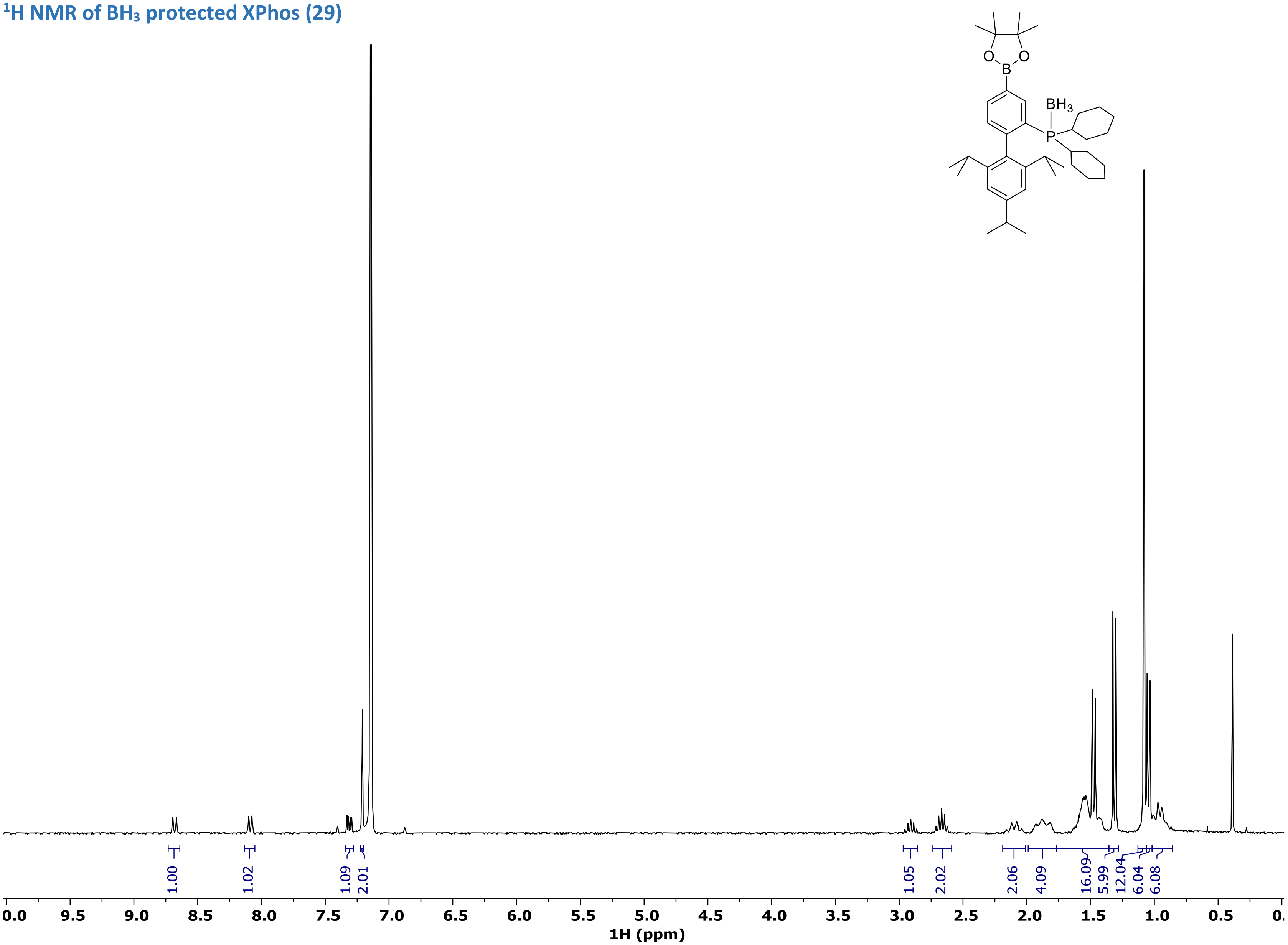

^{13}C NMR of BH_3 protected XPhos (29)

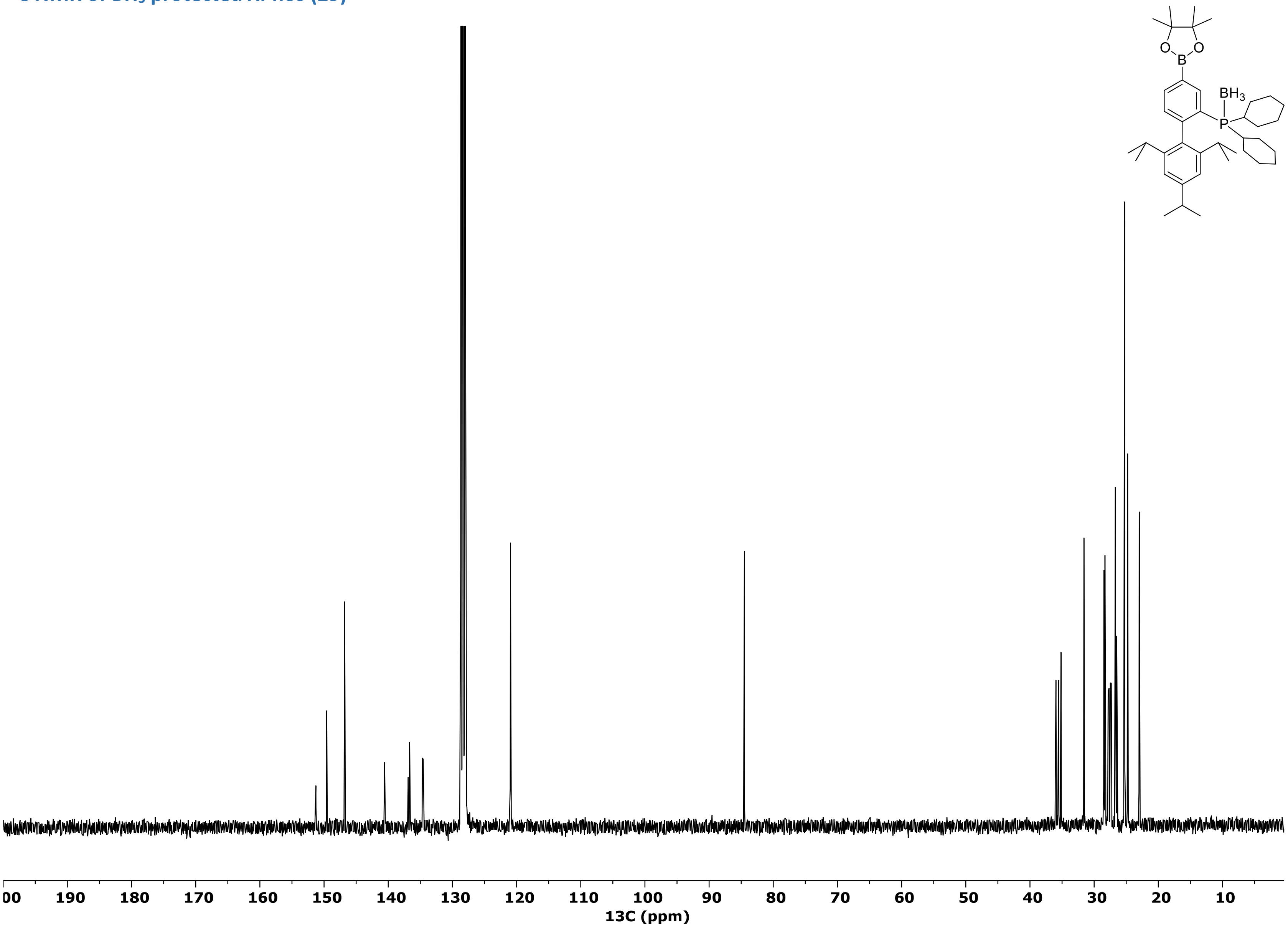

^{31}P NMR of BH_3 protected XPhos (29)

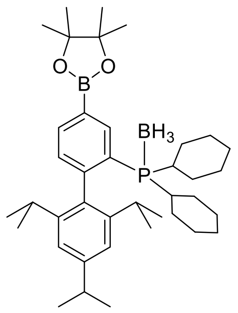

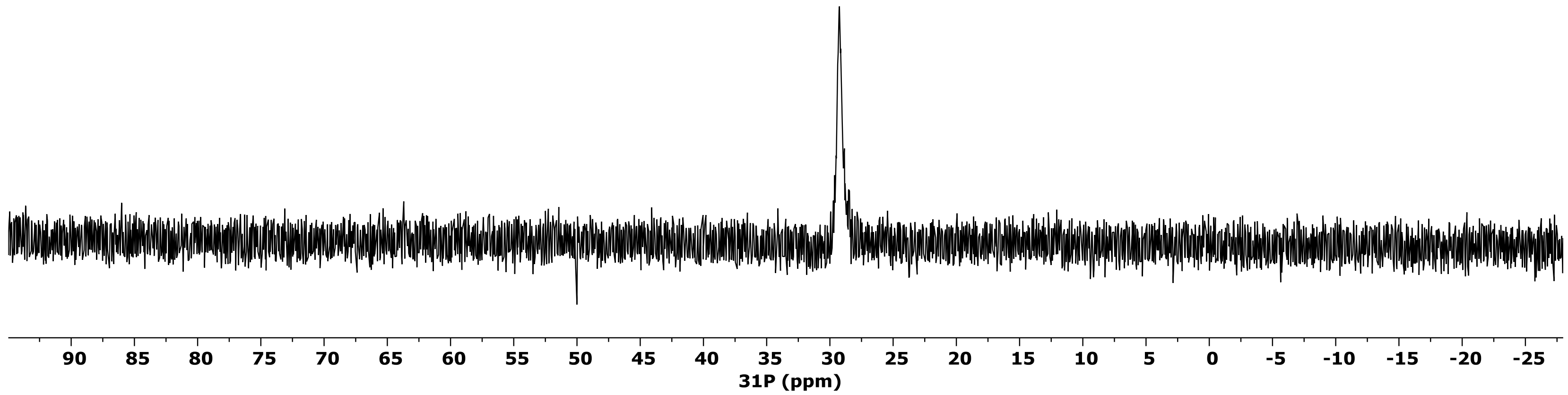

^{11}B NMR of BH_3 protected XPhos (29)

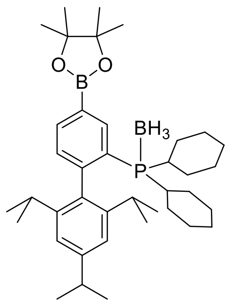

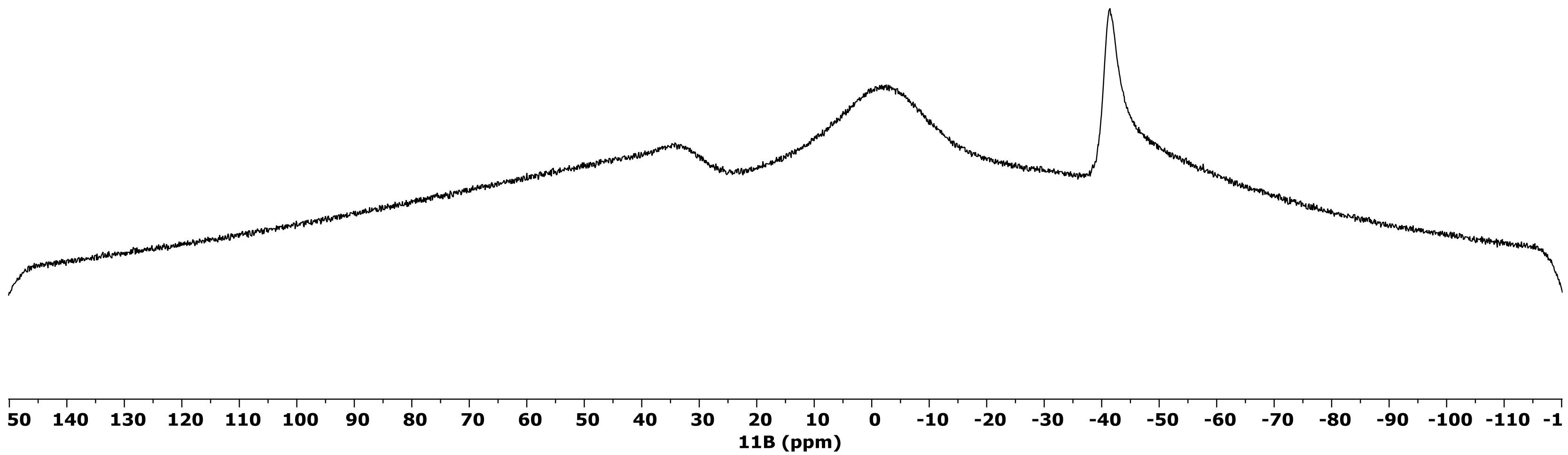

References

[24] N. C. Bruno, M. T. Tudge, S. L. Buchwald, *Chem. Sci.* **2013**, *4*, 916-920.

[25] S. D. McCann, E. C. Reichert, P. L. Arrechea, S. L. Buchwald, *J. Am. Chem. Soc.* **2020**, *142*, 15027-15037.

[26] L. M. Green, D. W. Meek, *Organometallics* **1989**, *8*, 659-666.

[27] a) X. Wang, W.-G. Liu, L.-T. Liu, X.-D. Yang, S. Niu, C.-H. Tung, L.-Z. Wu, H. Cong, *Org. Lett.* **2021**, *23*, 5485-5490; b) R.-B. Hu, H. Zhang, X.-Y. Zhang, S.-D. Yang, *Chem. Commun.* **2014**, *50*, 2193-2195.

[28] J. Wen, D. Wang, J. Qian, D. Wang, C. Zhu, Y. Zhao, Z. Shi, *Angew. Chem. Int. Ed.* **2019**, *58*, 2078-2082.

[29] K. C. Morris, S. E. Wright, G. F. Meyer, T. B. Clark, *J. Org. Chem.* **2020**, *85*, 14795-14801.

[30] K. M. Crawford, T. R. Ramseyer, C. J. A. Daley, T. B. Clark, *Angew. Chem. Int. Ed.* **2014**, *53*, 7589-7593.

[31] X. Meng, X. Li, D. Xu, *Tetrahedron: Asymmetry* **2009**, *20*, 1402-1406.

[32] O. Desponds, C. Huynh, M. Schlosser, *Synthesis* **1998**, *1998*, 983-985.
